# Supplementary material for: Rhodium-catalysed direct hydroarylation of alkenes and alkynes with phosphines through phosphorous-assisted C−H activation
Source: Nat Commun. 2019 Aug 6;10:3539. doi: 10.1038/s41467-019-11420-5 (PMC6684548; doi:10.1038/s41467-019-11420-5)
Supplement: Supplementary file 1 — Supplementary Information [file 41467_2019_11420_MOESM1_ESM.pdf]

Supplementary Information

Rhodium-Catalysed Direct Hydroarylation of Alkenes and Alkynes with Phosphines through Phosphorous-Assisted C−H Activation

Wang et al

Supplementary Methods:

General Information: All new compounds were fully characterized. Compounds were visualized by exposure to UV-light. All reactions and manipulations involving air- or moisture-sensitive compounds were performed using standard Schlenk techniques or in a glovebox. Toluene was purified using Pure Solv MD-5 solvent purification system, from Innovative Technology, Inc., by passing the solvent through two activated alumina columns after purging with argon. ^{1}H, ^{13}C, ^{31}P and ^{19}F NMR spectra were recorded on a Bruker AVANCE III 400 MHz or 500 MHz spectrometer. Chemical shifts (δ values) were reported in ppm with $CDCl_3$ (7.26 and 77.16 ppm for ^{1}H and ^{13}C respectively). Mass spectra were conducted at Agilent 6540 Ultra-High-Definition (UHD) Accurate-Mass Quadrupole Time-of-Flight (Q-TOF) liquid chromatography/mass spectrometry (LC/MS) system and Thermo Scientific TRACE 1300 ISQ LT gas chromatography/mass spectrometry (GC/MS) system. IR spectra were recorded on a Bruker FT-IR spectrometer. Chiral HPLC analyses were performed on a Thermo Scientific UltiMate 3000 liquid chromatography system. Unless otherwise noted, materials obtained from commercial suppliers were used without further purification. All reactions were carried out in flame-dried 25-mL Schlenk tubes with Teflon screw caps under argon. $[Rh(cod)Cl]_2$ were purchased from Strem. $[Rh(coe)_2Cl]_2$and **1f**[1], **D-1a**[1] were synthesized using standard procedures. Unless otherwise noted, materials obtained from commercial suppliers were used without further purification.

General Procedure for Hydroarylation of Alkenes

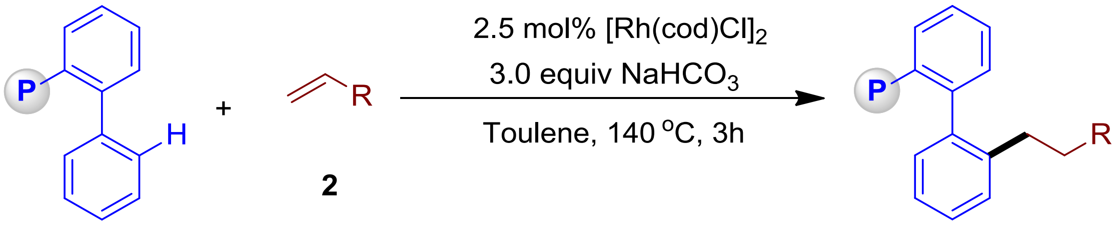

1 **2** **3**

In an oven-dried Schlenk tube, monophosphine ligands **1** (1.0 equiv, 0.20 mmol), alkenes **2** (1.0 equiv, 0.20 mmol), $[Rh(cod)Cl]_2$ (2.5 mol%, 2.5 mg, 0.005 mmol), $NaHCO_3$ (3.0 equiv, 50.4 mg, 0.60 mmol) were dissolved in toluene (1.0 mL). The mixture was stirred at 140 ℃ under argon for 3 hours. Upon the completion of the reaction, the solvent was removed. The crude mixture was directly subjected to column Chromatography on silica gel using petrol ether/ EtOAc as eluent to give the desired products **3**.

Methyl 3-(2'-(diphenylphosphanyl)-[1,1'-biphenyl]-2-yl)propanoate (3aa)

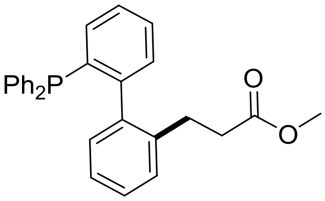

3aa

Following the general procedure, the reaction of **1a** (67.6 mg, 0.20 mmol), **2a** (17.2 mg, 0.20 mmol), $[Rh(cod)Cl]_2$ (2.5 mg, 0.005 mmol), $NaHCO_3$ (50.4 mg, 0.60 mmol) in toluene (1.0 mL) at 140 ℃ under Ar after 4 h, purification by column chromatographyon silica gel (petroleum-ether : ethyl acetate = 30:1) affording 63.5 mg (75%) of **3aa** as colorless oil. **^{1}H NMR (500 MHz, $CDCl_3$)** δ 7.42 (t, J = 7.5 Hz, 1H), 7.38 – 7.27 (m, 9 H), 7.26 – 7.17 (m, 5H), 7.14 (dd, J = 8.2, 4.1 Hz, 1H), 7.06 – 7.00 (m, 1H), 6.79 (d, J = 7.5 Hz, 1H), 3.65 (s, 3H), 2.77 (t, J = 8.1 Hz, 2H), 2.58 – 2.43 (m, 2H). **^{13}C NMR (126 MHz, $CDCl_3$)** δ 173.5, 147.0 (d, J = 31.1 Hz), 140.9 (d, J = 6.8 Hz), 138.1, 137.4 (d, J = 12.4 Hz), 137.1 (d, J = 8.7 Hz), 137.0 (d, J = 9.0 Hz), 134.1 (d, J = 14.8 Hz), 133.9 (d, J = 14.5 Hz), 133.8, 131.0 (d, J = 3.1 Hz), 130.1 (d, J = 5.2 Hz), 128.71 (d, J = 20.2 Hz), 128.5 (d, J = 3.4 Hz), 128.37 (d, J = 16.4 Hz), 128.4, 127.8 (d, J = 28.9 Hz), 125.5, 51.6, 34.8, 28.6 (d, J = 3.3 Hz). **^{31}P NMR (162 MHz, $CDCl_3$)** δ -14.00. **IR (cm^{-1}, neat)**:

3053, 2949, 1739, 1516, 1434, 745. **HRMS-ESI**: m/z calculated for $C_{28}H_{26}O_2P^+$ [M + H^+] 425.1665, found 425.1663.

Methyl 3-(2'-(diphenylphosphanyl)-6-methyl-[1,1'-biphenyl]-2-yl)propanoate (3ba)

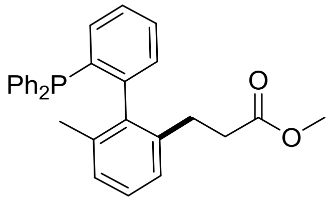

3ba

Following the modified general procedure, the reaction of **1b** (70.4 mg, 0.20 mmol), **2a** (51.7 mg, 0.60 mmol), $[Rh(cod)Cl]_2$ (5.0 mg, 0.01mmol), $NaHCO_3$ (50.4 mg, 0.60 mmol) in toluene (1.0 mL) at 150 ℃ under Ar after 24 h, purification by column chromatographyon silica gel (petroleum-ether : ethyl acetate = 30:1) affording 46.8 mg (53%) of **3ba** as colorless oil. **^{1}H NMR (500 MHz, $CDCl_3$)** δ 7.42 (t, J = 7.4 Hz, 1H), 7.36 – 7.25 (m, 8H), 7.24 – 7.16 (m, 5H), 7.16 – 7.11 (m, 1H), 7.04 (dd, J = 15.5, 7.6 Hz, 2H), 3.59 (s, 3H), 2.44 – 2.34 (m, 2H), 2.33 – 2.19 (m, 2H), 1.76 (s, 3H). **^{13}C NMR (126 MHz, $CDCl_3$)** δ 173.6, 146.3 (d, *J* = 32.8 Hz), 140.6 (d, *J* = 6.8 Hz), 138.7 (d, *J* = 2.1 Hz), 137.3 (d, *J* = 11.3 Hz), 137.0 (d, *J* = 12.1 Hz), 136.9 (d, *J* = 1.6 Hz), 136.7 (d, *J* = 11.9 Hz), 134.2 (d, *J* = 1.4 Hz), 134.1 (d, *J* = 4.5 Hz), 134.0 (d, *J* = 4.6 Hz), 130.0 (d, *J* = 5.8 Hz), 129.4, 128.6 (d, *J* = 4.5 Hz), 128.5 (d, *J* = 13.9 Hz), 128.4, 127.9 (d, *J* = 9.8 Hz), 127.6, 125.5, 51.5, 34.8, 28.6, 21.0 (d, *J* = 3.4 Hz). **^{31}P NMR (162 MHz, $CDCl_3$)** δ -14.63. **IR (cm^{-1}, neat)**: 3053, 2949, 1739, 1516, 1434, 1370, 1167, 884, 762, 697. **HRMS-ESI**: m/z calculated for $C_{29}H_{28}O_2P^+$ [M + H^+] 439.1821, found 439.1822.

Methyl3-(6'-(diphenylphosphanyl)-5-methyl-2',3'-dihydro-[1,1'-biphenyl]-2-yl)propanoate (3ca)

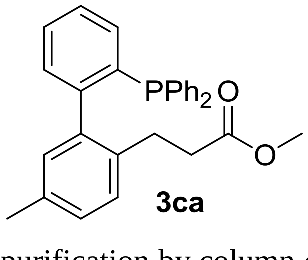

3ca

Following the modified general procedure A,the reaction of **1m** (87.7 mg, 0.20 mmol), **2a** (17.6 mg, 0.20 mmol), $[Rh(cod)Cl]_2$ (5.0 mg, 0.01mmol), $NaHCO_3$ (50.4 mg, 0.60 mmol) in toluene (1.0 mL) at 140 ℃ under Ar after 3.0h, purification by column chromatographyon silica gel (petroleum-ether : ethyl acetate = 30:1) affording 53.8 mg (61%) of **3ca** as colorless oil. **^{1}H NMR (500 MHz, $CDCl_3$)** δ

7.41 (t, J = 7.4 Hz, 1H), 7.38 – 7.30 (m, 7H), 7.28 – 7.20 (m, 5H), 7.18 (d, J = 7.8 Hz, 1H), 7.13 (dd, J = 7.7, 3.6 Hz, 1H), 7.09 (d, J = 7.8 Hz, 1H), 6.45 (s, 1H), 3.65 (s, 3H), 2.87 – 2.69 (m, 2H), 2.59 – 2.46 (m, 2H), 2.07 (s, 3H). **^{13}C NMR (126 MHz, $CDCl_3$)** δ 173.6, 146.9 (d, J = 31.0 Hz), 140.6 (d, J = 7.0 Hz), 137.5, 137.4, 137.3, 137.2, 137.1, 134.8 (d, J = 8.9 Hz), 134.3 (d, J = 20.5 Hz), 133.9 (d, J = 19.6 Hz), 133.6, 132.1 (d, J = 3.0 Hz), 129.9 (d, J = 5.1 Hz), 128.7, 128.6, 128.5, 128.4 (d, J = 2.0 Hz), 128.3, 128.3, 127.5, 51.6, 34.9 (d, J = 1.7 Hz), 28.3 (d, J = 3.3 Hz), 20.8. **^{31}P NMR (202 MHz, $CDCl_3$)** δ -13.6. **^{31}P NMR (162 MHz, Chloroform-*d*)** δ -13.6. **IR (cm^{-1}, neat)**: 3046, 2850, 2810, 1726, 1500, 1435, 1100, 742, 697. **HRMS-ESI**: m/z calculated for $C_{29}H_{27}O_2PNa^+$ [M + Na^+] 463.1797, found 463.1789.

Methyl 3-(2'-(diphenylphosphanyl)-6-methoxy-[1,1'-biphenyl]-2-yl)propanoate (3da)

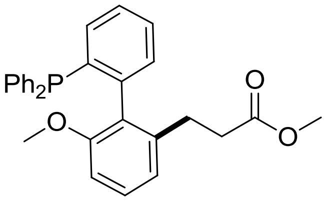

3da

Following the modified general procedure, the reaction of **1c** (73.7 mg, 0.20 mmol), **2a** (51.7 mg, 0.60 mmol), $[Rh(cod)Cl]_2$ (5.0 mg, 0.01mmol), $NaHCO_3$ (50.4 mg, 0.60 mmol) in toluene (1.0 mL) at 150 ℃ under Ar after 24 h, purification by column chromatographyon silica gel (petroleum-ether : ethyl acetate = 10:1) affording 75.6 mg (83%) of **3da** as colorless oil. **^{1}H NMR (500 MHz, $CDCl_3$)** δ 7.43 (t, J = 7.5 Hz, 1H), 7.35 – 7.26 (m, 8H), 7.25 – 7.14 (m, 6H), 6.89 (d, J = 7.7 Hz, 1H), 6.65 (d, J = 8.2 Hz, 1H), 3.62 (s, 3H), 3.23 (s, 3H), 2.61 (t, J = 8.1 Hz, 2H), 2.52 – 2.33 (m, 2H). **^{13}C NMR (126 MHz, $CDCl_3$)** δ 173.4, 157.2(d, J = 1.7 Hz), 143.5 (d, J = 33.5 Hz), 139.8, 137.9 (d, J = 12.2 Hz), 137.5 (d, J = 10.3 Hz), 137.3 (d, J = 12.1 Hz), 134.4 (d, J = 1.8 Hz), 133.9 (d, J = 15.2 Hz), 133.7 (d, J = 15.6 Hz), 130.4 (d, J = 5.9 Hz), 129.9 (d, J = 7.5 Hz), 128.97 (d, J = 32.4 Hz), 128.3, 128.2 (d, J = 27.7Hz), 128.0 (d, J = 6.9 Hz), 127.5, 120.4, 108.0, 54.7, 51.5, 34.7, 28.4 (d, J = 2.5 Hz). **^{31}P NMR (162 MHz, $CDCl_3$)** δ -14.19. **IR (cm^{-1}, neat)**: 3056, 2950, 1735, 1516, 1434, 1370, 1167, 884, 762. **HRMS-ESI**: m/z calculated for $C_{29}H_{28}O_3P^+$ [M + H^+] 455.1771, found 455.1774.

Methyl 3-(6-(dimethylamino)-2'-(diphenylphosphanyl)-[1,1'-biphenyl]-2-yl) propanoae (3ea)

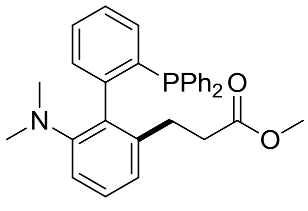

3ea

Following the modified general procedure, the reaction of **1d** (76.2 mg, 0.20 mmol), **2a** (51.7 mg, 0.60 mmol), $[Rh(cod)Cl]_2$ (5.0 mg, 0.01mmol), $NaHCO_3$ (50.4 mg, 0.60 mmol) in toluene (1.0 mL) at 150 ℃ under Ar after 24 h, purification by column chromatographyon silica gel (petroleum-ether : ethyl acetate = 10:1) affording 64.0 mg (68%) of **3ea** as light yellow oil. **^{1}H NMR (400 MHz, $CDCl_3$)** δ 7.42 (t, *J* = 7.9 Hz, 1H), 7.36 – 7.25 (m, 7H), 7.25 – 7.18 (m, 5H), 7.16 – 7.08 (m, 2H), 6.93 (dd, *J* = 7.8, 2.6 Hz, 2H), 3.58 (s, 3H), 2.53 (t, 2H), 2.47 – 2.35 (m, 1H), 2.28 – 2.17 (m, 1H), 2.15 (s, 6H). **^{13}C NMR (101 MHz, $CDCl_3$)** δ 173.5, 152.8 (d, *J* = 2.1 Hz), 145.7 (d, *J* = 34.6 Hz), 139.6, 139.4 (d, *J* = 14.3 Hz), 138.8 (d, *J* = 12.9 Hz), 137.4 (d, *J* = 10.1 Hz), 136.0 (d, *J* = 3.0 Hz), 133.8 (d, *J* = 20.4 Hz), 132.9 (d, *J* = 18.5 Hz), 131.5 (d, *J* = 6.7 Hz), 128.8 (d, *J* = 38.6 Hz), 128.3 (d, *J* = 6.4 Hz), 128.1 (d, *J* = 9.1 Hz), 128.0, 127.60, 127.2, 122.9, 116.9, 51.6, 43.6, 35.0, 28.7. **^{31}P NMR (162 MHz, $CDCl_3$)** δ -14.86. **IR (cm^{-1}, neat)**: 3053, 2947, 1734, 1577, 1169, 744, 698. **HRMS-ESI**: m/z calculated for $C_{30}H_{31}NO_2P^+$ [M + H^+] 468.2087, found 468.2082.

Methyl 3-(2'-(diphenylphosphanyl)-4-fluoro-[1,1'-biphenyl]-2-yl)propanoate (3fa)

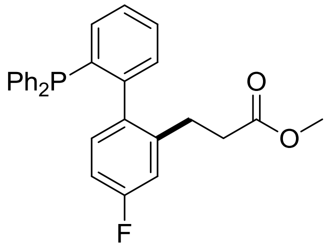

3fa

Following the general procedure, the reaction of **1e** (71.2 mg, 0.20 mmol), **2a** (51.7 mg, 0.60 mmol), $[Rh(cod)Cl]_2$ (2.5 mg, 0.005mmol), $NaHCO_3$ (50.4 mg, 0.60 mmol) in toluene (1.0 mL) at 140 ℃ under Ar after 3.5 h, purification by column chromatographyon silica gel (petroleum-ether : ethyl acetate = 30:1) affording 59.2 mg (67%) of **3fa** as colorless oil.**^{1}H NMR (400 MHz, $CDCl_3$)** δ 7.39 (t, *J* = 6.8 Hz, 1H), 7.37 – 7.24 (m, 7H), 7.25 – 7.13 (m, 5H), 7.09 (dd, *J* = 7.1, 4.1 Hz, 1H), 6.94 (d, *J* = 11.3 Hz, 1H), 6.75 – 6.64 (m, 2H), 3.64 (s, 3H), 2.79 – 2.65 (m, 2H), 2.56 – 2.39 (m,

2H). **^{13}C NMR (101 MHz, CDCl$_3$)** δ 173.1, 162.3 (d, *J* = 245.8 Hz), 145.9 (d, *J* = 30.5 Hz), 140.6 (d, *J* = 7.1 Hz), 137.5 (d, *J* = 11.9 Hz), 136.9 (d, *J* = 11.8 Hz), 136.8, 136.7 (d, *J* = 2.9 Hz), 136.69, 134.2 (d, *J* = 13.3 Hz), 134.0 (d, *J* = 13.2 Hz), 133.7, 132.4 (dd, *J* = 8.3 Hz,*J* = 3.0 Hz), 130.4 (d, *J* = 5.0 Hz), 128.8 (d, *J* = 8.2 Hz), 128.6 (d, *J* = 8.9 Hz), 128.5 (d, *J* = 5.2 Hz), 128.4, 127.9, 115.0 (d, *J* = 21.4 Hz), 112.4 (d, *J* = 21.0 Hz), 51.6, 34.3 (d, *J* = 1.9 Hz), 28.5 (dd, *J* = 3.7, 1.5 Hz). **^{31}P NMR (162 MHz, CDCl$_3$)** δ -13.67. **^{19}F NMR (376 MHz, CDCl$_3$)** δ -114.64. **IR (cm^{-1}, neat)**: 3052, 2949, 1739, 1610, 1585, 1499, 1363, 1154, 744. **HRMS-ESI**: m/z calculated for $C_{28}H_{25}FO_2P^+$ [M + H$^+$] 443.1571, found 443.1567.

Methyl 3-(3-(2-(diphenylphosphanyl)phenyl)naphthalen-2-yl)propanoate (3ga)

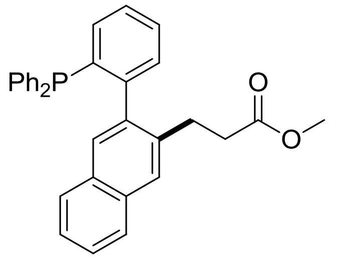

3ga

Following the general procedure, the reaction of **1f** (76.7 mg, 0.20 mmol), **2a** (51.7 mg, 0.60 mmol), [Rh(cod)Cl]$_2$ (2.5 mg, 0.005mmol), NaHCO$_3$ (50.4 mg, 0.60 mmol) in toluene (1.0 mL) at 140 ℃ under Ar after 4h, purification by column chromatographyon silica gel (petroleum-ether : ethyl acetate = 30:1) affording 43.0 mg (45%) of **3ga** as colorless oil. **^{1}H NMR (400 MHz, CDCl$_3$)** δ 7.78 (d, *J* = 8.1 Hz, 1H), 7.70 (s, 1H), 7.45 – 7.38 (m, 2H), 7.35 – 7.25 (m, 9H), 7.24 – 7.08 (m, 6H), 7.04 (s, 1H), 3.62 (s, 3H), 3.05 – 2.88 (m, 2H), 2.71 – 2.52 (m, 2H). **^{13}C NMR (101 MHz, CDCl$_3$)** δ 173.6, 146.6 (d, *J* = 31.1 Hz), 139.4 (d, *J* = 7.1 Hz), 137.6 (d, *J* = 4.5 Hz), 137.5 (d, *J* = 4.3 Hz), 136.9 (d, *J* = 11.0 Hz), 136.5, 134.4 (d, *J* = 20.4 Hz), 133.8 (d, *J* = 19.5 Hz), 133.2, 131.5, 130.4, 128.5 (d, *J* = 6.4 Hz), 128.4 (d, *J* = 4.8 Hz), 128.3, 127.9, 127.7, 127.4, 126.8, 126.0, 125.6, 51.7, 34.7 (d, *J* = 1.8 Hz), 29.0 (d, *J* = 3.6 Hz). **^{31}P NMR (162 MHz, CDCl$_3$)** δ -13.61. **IR (cm^{-1}, neat)**: 3069, 2959, 1734, 1434, 1247, 1167, 1027, 744, 698. **HRMS-ESI**: m/z calculated for $C_{32}H_{28}O_2P^+$ [M + H$^+$] 475.1821, found 475.1823.

Methyl 3-(2'-(dicyclohexylphosphanyl)-[1,1'-biphenyl]-2-yl)propanoate (3ha)

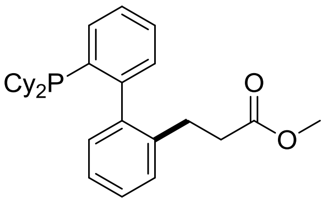

3ha

Following the modified general procedure, the reaction of **1g** (70.1 mg, 0.20 mmol), **2a** (34.4 mg, 0.40 mmol), $[Rh(cod)Cl]_2$ (2.5 mg, 0.005mmol), NaOAc (49.2 mg, 0.60 mmol) in toluene (1.0 mL) at 150 ℃ under Ar after 24 h, purification by column chromatographyon silica gel (petroleum-ether : ethyl acetate = 20:1) affording 58.6 mg (67%) of **3ha** as colorless oil. **^{1}H NMR (400 MHz, CDCl$_3$)** δ 7.68 – 7.50 (m, 1H), 7.39 – 7.33 (m, 2H), 7.30 (t, *J* = 7.5 Hz, 1H), 7.24 – 7.15 (m, 3H), 7.06 (d, *J* = 7.4 Hz, 1H), 3.57 (s, 3H), 2.80 – 2.64 (m, 2H), 2.55 – 2.35 (m, 2H), 2.12 – 1.91 (m, 1H), 1.86 – 1.56 (m, 11H), 1.35 – 0.84 (m, 10H). **^{13}C NMR (101 MHz, CDCl$_3$)** δ 173.4, 149.1 (d, *J* = 31.0 Hz), 142.3 (d, *J* = 6.0 Hz), 137.9, 134.5 (d, *J* = 19.8 Hz), 132.7, 131.3, 130.4 (d, *J* = 5.8 Hz), 128.3, 127.9, 127.6, 126.6, 125.2, 51.4, 35.5 (d, *J* = 15.5 Hz), 34.7, 33.1 (d, *J* = 12.7 Hz), 30.8 (d, *J* = 14.6 Hz), 30.0, 29.9 (d, *J* = 4.7 Hz), 29.8, 28.8 (d, *J* = 2.1 Hz), 28.76 (d, *J* = 2.7 Hz), 27.7, 27.4 (d, *J* = 21.8 Hz), 27.37 (d, *J* = 37.0 Hz), 27.1, 26.4 (d, *J* = 9.6 Hz). **^{31}P NMR (162 MHz, CDCl$_3$)** δ -11.92. **IR (cm^{-1}, neat)**: 2925, 2849, 1741, 1447, 1168, 742. **HRMS-ESI**: m/z calculated for $C_{28}H_{38}O_2P^+$[M + H$^+$] 437.2604, found437.2609.

Methyl 3-(2'-(dicyclohexylphosphanyl)-6-methoxy-[1,1'-biphenyl]-2-yl) propanoate (3ia)

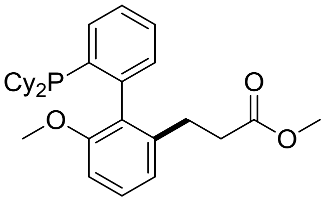

3ia

Following the modified general procedure, the reaction of **1h** (76.0 mg, 0.20 mmol), **2a** (51.7 mg, 0.60 mmol), $[Rh(cod)Cl]_2$ (2.5 mg, 0.005mmol), NaOAc (49.2 mg, 0.60 mmol) in toluene (1.0 mL) at 150 ℃ under Ar after 24 h, purification by column chromatographyon silica gel (petroleum-ether : ethyl acetate = 10:1) affording 43.6 mg (47%) of **3ia** as colorless oil. **^{1}H NMR (400 MHz, CDCl$_3$)** δ 7.57 (d, *J* = 7.0 Hz, 1H), 7.40 – 7.32 (m, 2H), 7.28 (dd, *J* = 16.1, 8.1 Hz, 1H), 7.17 – 7.08 (m, 1H), 6.87 (d, *J* = 7.6 Hz, 1H), 6.76 (dt, *J* = 8.1 Hz, 1H), 3.64 (s, 3H), 3.58 (s, 3H), 2.73 – 2.50 (m, 2H), 2.45 (t, *J* = 8.2 Hz, 2H), 2.06 – 1.85 (m, 1H), 1.76 -1.55 (m, 11H), 1.36 – 0.96 (m, 10H). **^{13}C NMR (101

MHz, $CDCl_3$) δ 173.5, 156.8, 144.7 (d, *J* = 31.4 Hz), 139.6, 135.7 (d, *J* = 18.4 Hz), 132.6 (d, *J* = 3.2 Hz), 131.0 (d, *J* = 5.9 Hz), 130.8 (d, *J* = 6.4 Hz), 128.5, 128.3, 126.5, 120.3, 107.8, 55.0, 51.5, 34.9 (d, *J* = 15.0 Hz), 34.8, 33.2 (d, *J* = 13.2 Hz), 30.3, 30.1, 29.8, 29.7 (d, *J* = 1.8 Hz), 29.4 (d, *J* = 13.1 Hz), 28.8 (d, *J* = 4.1 Hz), 27.8 (d, *J* = 3.0 Hz), 27.7, 27.3 (d, *J* = 4.3 Hz), 27.2 (d, *J* = 6.9 Hz), 26.5. **^{31}P NMR (162 MHz, $CDCl_3$)** δ -9.85. **IR (cm^{-1}, neat)**: 2924, 2849, 1740, 1579, 1468, 1436, 1256, 1169, 745. **HRMS-ESI**: m/z calculated for $C_{29}H_{40}O_3P^+$ [M + H$^+$] 467.2710, found 467.2706.

Methyl 3-(2'-(dicyclohexylphosphanyl)-6-(dimethylamino)-[1,1'-biphenyl]-2-yl) propanoate (3ja)

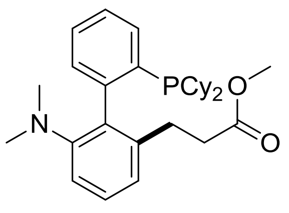

3ja

Following the modified general procedure, the reaction of **1i** (78.7 mg, 0.20 mmol), **2a** (51.6 mg, 0.60 mmol), $[Rh(cod)Cl]_2$ (2.5 mg, 0.005mmol), NaOAc (49.2 mg, 0.60 mmol) in toluene (1.0 mL) at 150 ℃ under Ar after 36 h, purification by column chromatographyon silica gel (petroleum-ether : ethyl acetate = 5:1) affording 49.8 mg (52%) of **3ja** as colorless oil. **^{1}H NMR (400 MHz, $CDCl_3$)** δ 7.59 (d, *J* = 7.4 Hz, 1H), 7.42 – 7.27 (m, 3H), 7.23 – 7.18 (m, 1H), 6.92 (dd, *J* = 2.1 Hz, *J* = 0.5 Hz 2H), 3.62 (s, 3H), 2.73 – 2.48 (m, 4H), 2.41 (s, 6H), 2.02 – 1.44 (m, 11H), 1.44 – 0.84 (m, 11H). **^{13}C NMR (101 MHz, $CDCl_3$)** δ 173.6, 152.4, 146.7 (d, *J* = 31.1 Hz), 139.5, 136.5 (d, *J* = 19.2 Hz), 136.0, 133.1 (d, *J* = 3.5 Hz), 131.8 (d, *J* = 6.0 Hz), 128.2, 127.9, 126.2, 121.9, 116.1, 51.5, 44.1, 35.5, 35.4 (d, *J* = 15.3 Hz), 34.3 (d, *J* = 14.9 Hz), 30.5 (d, *J* = 10.5 Hz), 30.4 (d, *J* = 9.2 Hz), 30.0, 29.8, 29.2 (d, *J* = 8.3 Hz), 28.9, 27.8 (d, *J* = 10.7 Hz), 27.5 (d, *J* = 1.8 Hz), 27.4 (d, *J* = 2.3 Hz), 26.7, 26.4. **^{31}P NMR (162 MHz, $CDCl_3$)** δ -9.79. **IR (cm^{-1}, neat)**: 2924, 2849, 1741, 1516, 1447, 1168, 742. **HRMS-ESI**: m/z calculated for $C_{30}H_{43}NO_2P^+$ [M + H$^+$] 480.3026, found 480.3027.

Methyl 3-(2'-(di-tert-butylphosphanyl)-[1,1'-biphenyl]-2-yl)propanoate (3ka)

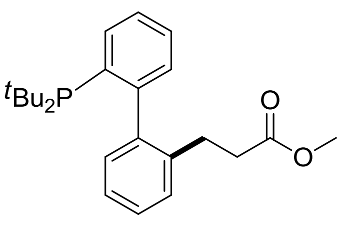

3ka

Following the modified general procedure, the reaction of **1j** (59.6 mg, 0.20 mmol), **2a** (51.6 mg, 0.60 mmol), $[Rh(cod)Cl]_2$ (2.5 mg, 0.005mmol), NaOAc (49.2 mg, 0.60 mmol) in toluene (1.0 mL) at 150 ℃ under Ar after 24 h, purification by column chromatographyon silica gel (petroleum-ether : ethyl acetate = 30:1) affording 33.6 mg (41%) of **3ka** as colorless oil. **^{1}H NMR (500 MHz, $CDCl_3$)** δ 7.88 (d, *J* = 7.0 Hz, 1H), 7.40 – 7.32 (m, 2H), 7.30 (t, *J* = 7.5 Hz, 1H), 7.23 (d, *J* = 7.6 Hz, 1H), 7.21 – 7.15 (m, 2H), 7.09 (d, *J* = 7.6 Hz, 1H), 3.60 (s, 3H), 2.87 – 2.70 (m, 2H), 2.52 (t, *J* = 8.1 Hz, 2H), 1.19 (d, *J* = 11.6 Hz, 9H), 1.12 (d, *J* = 11.4 Hz, 9H). **^{13}C NMR (126 MHz, $CDCl_3$)** δ 173.5, 149.6 (d, *J* = 34.0 Hz), 142.7 (d, *J* = 6.5 Hz), 137.8 (d,*J* = 1.3 Hz), 136.2 (d, *J* = 27.8 Hz), 135.6 (d, *J* = 2.8 Hz), 131.9 (d, *J* = 2.8 Hz), 130.9 (d, *J* = 6.6 Hz), 128.4 (d,*J* = 1.3 Hz), 127.7, 127.4, 126.0, 124.9, 51.5, 35.1, 33.3 (d, *J* = 23.9 Hz), 32.5 (d, *J* = 25.2 Hz), 31.2 (d, *J* = 15.4 Hz), 30.7 (d, *J* = 14.6 Hz), 28.7. **^{31}P NMR (162 MHz, $CDCl_3$)** δ 20.44. **IR (cm^{-1}, neat)**: 2973, 1746, 1516, 1462, 1366, 1143, 745. **HRMS-ESI**: m/z calculated for $C_{24}H_{34}O_2P^+$ [M + H^+] 385.2291, found 385.2296.

Methyl 3-(2-(2-(diphenylphosphanyl)-1H-pyrrol-1-yl)phenyl)propanoate (3la)

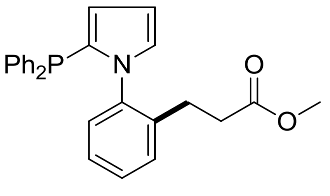

3la

Following the general procedure, the reaction of **1k** (65.5 mg, 0.20 mmol), **2a** (17.2 mg, 0.20 mmol), $[Rh(cod)Cl]_2$ (2.5 mg, 0.005mmol), $NaHCO_3$ (50.4 mg, 0.60 mmol) in toluene (1.0 mL) at 150 ℃ under Ar after 2h, purification by column chromatographyon silica gel (petroleum-ether : ethyl acetate = 30:1) affording 55.7 mg (66%) of **3la** as colorless oil. **^{1}H NMR (400 MHz, $CDCl_3$)** δ 7.37 – 7.26 (m, 12H), 7.11 (t, *J* = 8.3 Hz, 1H), 7.03 – 6.90 (m, 2H), 6.41 – 6.32 (t, *J* = 2.8 Hz, 1H), 6.15 (dd, *J* = 3.6 Hz, *J* = 1.5 Hz1H), 3.64 (s, 3H), 2.71 (t, *J* = 7.9 Hz, 2H), 2.55 – 2.34 (m, 2H). **^{13}C NMR (101 MHz, $CDCl_3$)** δ 173.2, 139.3 (d,*J* = 3.0 Hz)), 138.4, 137.5(d, *J* = 6.6 Hz), 133.5 (d, *J* = 19.8 Hz), 130.0, 129.43, 129.4 (d, *J* = 3.0 Hz), 129.0, 128.6 (d, *J* = 9.5 Hz), 128.4 (d, *J* = 2.4 Hz), 128.3 (d, *J* = 2.2 Hz), 127.1 (d, *J* = 2.3 Hz), 126.7, 118.8, 109.8 (d, *J* = 1.4 Hz), 51.7, 34.4 (d, *J* =

1.4 Hz), 26.4 (d, *J* = 3.0 Hz). **^{31}P NMR (162 MHz, $CDCl_3$)** δ -30.74. **IR (cm^{-1}, neat)**: 3069, 2950, 1739, 1579, 1468, 1436, 1256, 1169, 1082, 741. **HRMS-ESI**: m/z calculated for $C_{26}H_{25}NO_2P^+$ [M + H^+] 414.1617, found 414.1621.

Methyl 3-(2-(2-(dicyclohexylphosphanyl)-1H-pyrrol-1-yl)phenyl)propanoate (3ma)

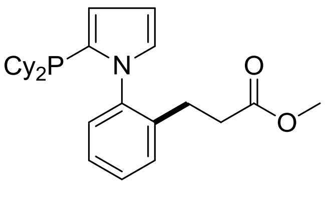

3ma

Following the modified general procedure, the reaction of **1l** (67.9 mg, 0.20 mmol), **2a** (51.6 mg, 0.60 mmol), $[Rh(cod)Cl]_2$ (2.5 mg, 0.005mmol), NaOAc (49.2 mg, 0.60 mmol) in toluene (1.0 mL) at 150 ℃ under Ar after 24 h, purification by column chromatographyon silica gel (petroleum-ether : ethyl acetate = 20:1) affording 36.5 mg (43%) of **3ma** ascolorlessoil.**^{1}H NMR (400 MHz, $CDCl_3$)** δ 7.39 – 7.33 (m, 1H), 7.31 – 7.23 (m, 2H), 7.15 (d, *J* = 8.7 Hz, 1H), 6.86 – 6.81 (m, 1H), 6.49 (dd, *J* = 3.6, 1.5 Hz, 1H), 6.38 (dd, *J* = 3.5, 2.7 Hz, 1H), 3.60 (s, 3H), 2.73 – 2.62 (m, 2H), 2.45 (t, *J* = 8.2 Hz, 2H), 2.06 – 1.87 (m, 1H), 1.87 – 1.51 (m, 11H), 1.33 – 0.84 (m, 10H). **^{13}C NMR (101 MHz, $CDCl_3$)** δ 173.1, 140.0, 138.2, 129.8 (d, *J* = 2.2 Hz), 128.9, 128.6, 128.3 (d, *J* = 7.7 Hz), 126.3, 126.2 (d, *J* = 2.0 Hz), 116.5 (d, *J* = 4.5 Hz), 109.0, 51.6, 34.7 (d, *J* = 10.4 Hz), 34.3, 33.5 (d, *J* = 5.4 Hz), 30.8 (d, *J* = 15.2 Hz), 30.0 (d, *J* = 6.4 Hz), 29.9, 28.1 (d, *J* = 1.9 Hz), 27.6, 27.5, 27.3, 27.2, 27.1, 27.0, 26.4 (d, *J* = 4.9 Hz). **^{31}P NMR (162 MHz, $CDCl_3$)** δ -26.90.**IR (cm^{-1}, neat)**: 2925, 2849, 1741, 1496, 1448, 1167, 744. **HRMS-ESI**: m/z calculated for $C_{26}H_{37}NO_2P^+$ [M + H^+] 426.2556, found 426.2559.

Butyl 3-(2'-(diphenylphosphanyl)-[1,1'-biphenyl]-2-yl)propanoate (3ab)

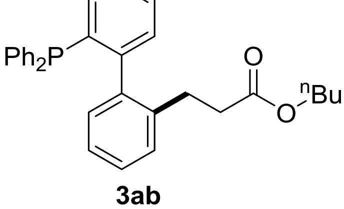

3ab

Following the general procedure, the reaction of **1a** (67.6 mg, 0.20 mmol), **2b** (17.2 mg, 0.20 mmol), $[Rh(cod)Cl]_2$ (2.5 mg, 0.005mmol), $NaHCO_3$ (50.4 mg, 0.60 mmol) in toluene (1.0 mL) at 140 ℃ under Ar after 4 h, purification by column chromatographyon silica gel (petroleum-ether : ethyl acetate = 30:1) affording 72.6 mg (77%) of **3ab** as colorless

oil. **^{1}H NMR (400 MHz, CDCl$_3$)** δ 7.38 (t, *J*= 7.4 Hz, 1H), 7.34 – 7.25 (m, 7H), 7.26 – 7.20 (m, 3H), 7.21 – 7.12 (m, 4H), 7.09 (dd, *J* = 7.6, 3.6 Hz, 1H), 7.05 – 6.93 (m, 1H), 6.74 (d, *J* = 7.5 Hz, 1H), 4.01 (t, *J* = 6.6 Hz, 2H), 2.72 (t, *J* = 8.1 Hz, 2H), 2.55 – 2.35 (m, 2H), 1.68 – 1.47 (m, 2H), 1.44 – 1.18 (m, 2H), 0.89 (t, *J* = 7.4 Hz, 3H). **^{13}C NMR (101 MHz, CDCl$_3$)** δ 173.2, 147.2 (d, *J* = 31.2 Hz), 141.1 (d, *J* = 7.1 Hz), 138.3, 137.6 (d, *J* = 12.3 Hz), 137.2 (d, *J* = 5.9 Hz), 137.1 (d, *J* = 6.4 Hz), 134.2(d, *J* = 15.0 Hz), 134.1 (d, *J* = 14.9 Hz), 133.9, 131.1 (d, *J* = 3.2 Hz), 130.3 (d, *J* = 5.2 Hz), 128.8(d, *J* = 14.7 Hz), 128.6 (d, *J* = 2.4 Hz), 128.5, 128.4 (d, *J* = 7.0 Hz), 127.9 (d, *J* = 23.4 Hz), 125.6, 64.3, 35.1 (d, *J* = 1.6 Hz), 30.8, 28.7 (d, *J* = 3.3 Hz), 13.8. **^{31}P NMR (162 MHz, CDCl$_3$)** δ -13.95. **IR (cm^{-1}, neat)**: 3069, 2950, 1738, 1584, 1434, 1247, 1167, 1027, 744. **HRMS-ESI**: m/z calculated for $C_{31}H_{32}O_2P^+$ [M + H$^+$] 467.2134, found 467.2130.

Diphenyl(2'-(2-(phenylsulfonyl)ethyl)-[1,1'-biphenyl]-2-yl)phosphane(3ac)

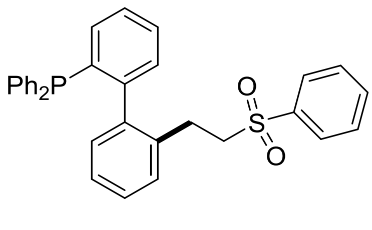

3ac

Following the modified general procedure, the reaction of **1a** (67.6 mg, 0.20 mmol), **2c** (100.9 mg, 0.60 mmol), [Rh(coe)$_2$Cl]$_2$ (3.6 mg, 0.005mmol), K_2CO_3 (83.0 mg, 0.60 mmol) in toluene (1.0 mL) at 150 ℃ under Ar after 24 h, purification by column chromatographyon silica gel (petroleum-ether : ethyl acetate = 20:1) affording 48.9 mg (45%) of **3ac** as orange-yellow oils. **^{1}H NMR (500 MHz, CDCl$_3$)** δ 7.70 (d, *J* = 7.7 Hz, 2H), 7.60 (t, *J* = 7.4 Hz, 1H), 7.46 (t, *J* = 7.7 Hz, 2H), 7.38 – 7.22 (m, 9H), 7.18 (d, *J* = 7.6 Hz, 1H), 7.08 (dt, *J* = 11.4, 6.5 Hz, 5H), 6.98 (t, *J* = 6.4 Hz, 2H), 6.66 (d, *J* = 7.5 Hz, 1H), 3.41 – 3.16 (m, 2H), 2.91 – 2.73 (m, 2H). **^{13}C NMR (126 MHz, CDCl$_3$)** δ 145.9 (d, *J* = 30.6 Hz), 140.9 (d, *J* = 6.5 Hz), 138.7, 136.9 (d, *J* = 12.2 Hz), 136.8 (d, *J* = 11.6 Hz), 136.5 (d, *J* = 10.4 Hz), 135.1, 134.1 (d, *J* = 12.0 Hz), 134.0 (d, *J* = 12.1 Hz), 133.6 (d, *J* = 5.3 Hz), 131.3 (d, *J* = 3.2 Hz), 130.0(d, *J* = 5.0 Hz), 128.9 (d, *J* = 9.6 Hz), 128.8 (d, *J* = 3.5 Hz), 128.6 (d, *J* = 6.7 Hz), 128.4 (d, *J* = 7.4 Hz), 128.3 (d, *J* = 35.6 Hz), 128.2,127.8, 126.3, 56.3, 27.3 (d, *J* = 4.2 Hz). **^{31}P NMR (162 MHz, CDCl$_3$)** δ -13.51. **IR (cm^{-1}, neat)**: 3069, 1613, 1516, 1447, 1307, 1141, 1086,

807, 745, 688. **HRMS-ESI**: m/z calculated for $C_{32}H_{28}O_2PS^+$ [M + H^+] 507.1542, found 507.1544.

(2'-phenethyl-[1,1'-biphenyl]-2-yl)diphenylphosphane (3ad)

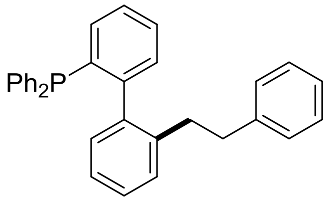

3ad

Following the modified general procedure,the reaction of **1a** (67.6 mg, 0.20 mmol), **2d** (62.4 mg, 0.60 mmol), $[Rh(coe)_2Cl]_2$ (7.2 mg, 0.01mmol), K_2CO_3 (83.0 mg, 0.60 mmol) in toluene (1.0 mL) at 150 ℃ under Ar after 24 h, purification by column chromatographyon silica gel (petroleum-ether : ethyl acetate = 500:1) affording 72.8 mg (82%) of **3ad** as colorless oil. **^{1}H NMR (500 MHz, $CDCl_3$)** δ 7.42 (t, *J* = 7.4 Hz, 1H), 7.38 – 7.29 (m, 9H), 7.28 – 7.20 (m, 8H), 7.18 (dd, *J* = 7.7, 4.7 Hz, 1H), 7.07 – 7.03 (m, 1H), 7.01 (d, *J* = 7.0 Hz, 2H), 6.81 (d, *J* = 7.5 Hz, 1H), 2.90 – 2.81 (m, 2H), 2.82 – 2.69 (m, 2H). **^{13}C NMR (126 MHz, $CDCl_3$)** δ 147.2 (d, *J* = 31.2 Hz), 142.2, 141.0 (d, *J* = 6.9 Hz), 139.4, 137.6 (d, *J* = 12.3 Hz), 137.3 (d, *J* = 11.3 Hz), 137.0 (d, *J* = 11.6 Hz), 134.1(d, *J* = 20.1 Hz), 133.9 (d, *J* = 19.7 Hz), 130.9 (d, *J* = 3.1 Hz), 130.2 (d, *J* = 5.3 Hz), 130.0 (d, *J* = 4.7 Hz), 128.8, 128.7 (d, *J* = 8.3 Hz), 128.5, 128.46 (d, *J* = 3.2 Hz), 128.4, 128.3, 128.35, 127.7 (d, *J* = 27.4 Hz), 125.9, 125.2, 37.1, 35.7 (d, *J* = 3.1 Hz). **^{31}P NMR (202 MHz, $CDCl_3$)** δ -13.97. **IR (cm^{-1}, neat)**: 3053, 2925, 1584, 1434, 910, 1144, 743. **HRMS-ESI**: m/z calculated for $C_{32}H_{28}P^+$ [M + H^+] 443.1923, found 443.1920.

Diphenyl(2'-(4-(trifluoromethyl)phenethyl)-[1,1'-biphenyl]-2-yl)phosphane (3ae)

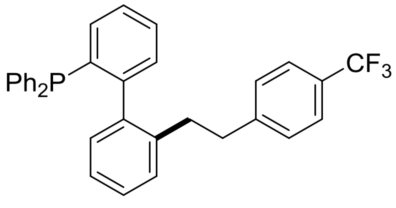

3ae

Following the modified general procedure, the reaction of **1a** (67.6 mg, 0.20 mmol), **2e** (103.3 mg, 0.60 mmol), $[Rh(coe)_2Cl]_2$ (7.2 mg, 0.01mmol), K_2CO_3 (83.0 mg, 0.60 mmol) in toluene (1.0 mL) at 150 ℃ under Ar after 24 h, purification by column chromatographyon silica gel (petroleum-ether : ethyl acetate = 500:1) affording 41.6 mg (41%) of **3ae** as colorless oil. **^{1}H NMR (500 MHz, $CDCl_3$)** δ 7.44 (d, *J* = 8.0 Hz,

2H), 7.38 – 7.19 (m, 10H), 7.19 – 7.08 (m, 7H), 7.00 (d, *J* = 7.8 Hz, 2H), 6.74 (d, *J* = 7.3 Hz, 1H), 2.87 – 2.76 (m, 2H), 2.76 – 2.62 (m, 2H). **^{13}C NMR (126 MHz, $CDCl_3$)** δ 147.1 (d, *J* = 31.1 Hz), 146.1, 141.0 (d, *J* = 6.9 Hz), 138.7, 137.5 (d, *J* = 12.2 Hz), 137.2 (d, *J* = 7.6 Hz), 137.1 (d, *J* = 8.4 Hz), 134.4 (d, *J* = 31.2 Hz), 134.1, 134.0 (d, *J* = 1.4 Hz), 133.8 (d, *J* = 4.6 Hz), 131.0 (d, *J* = 3.0 Hz), 130.2 (d, *J* = 5.3 Hz), 128.8, 128.75 (d, *J* = 3.2 Hz),128.7, 128.6 (d, *J* = 3.3 Hz), 128.5, 128.4 (d, *J* = 7.0 Hz), 127.8 (d, *J* = 24.3 Hz), 126.5 (q, *J*=272.2 Hz), 125.3, 125.2 (q, *J* = 3.8 Hz), 36.7, 35.4 (d, *J* = 3.4 Hz). **^{19}F NMR (471 MHz, $CDCl_3$)** δ -62.25. **IR (cm^{-1}, neat)**: 3053, 2930, 1516, 1439, 1237, 1165, 743. **HRMS-ESI**: m/z calculated for $C_{33}H_{27}F_3P^+$ [M + H$^+$] 511.1797, found 511.1799.

3-(2'-(Diphenylphosphanyl)-[1,1'-biphenyl]-2-yl)propanenitrile (3af)

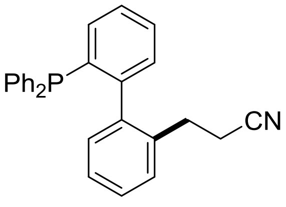

3af

Following the modified general procedure, the reaction of **1a** (67.6 mg, 0.20 mmol), **2f** (31.8 mg, 0.60 mmol), $[Rh(cod)Cl]_2$ (5.0 mg, 0.01mmol), $NaHCO_3$ (50.4 mg, 0.60 mmol) in toluene (1.0 mL) at 140 ℃ under Ar after 4 h, purification by column chromatographyon silica gel (petroleum-ether : ethyl acetate = 30:1) affording 62.3 mg (80%) of **3af** as white solids. **^{1}H NMR (500 MHz, $CDCl_3$)** δ 7.44 (td, *J* = 7.5, 1.3 Hz, 1H), 7.38 – 7.28 (m, 9H), 7.25 – 7.19 (m, 3H), 7.19 – 7.12 (m, 3H), 7.08 (t, *J* = 7.4 Hz, 1H), 6.82 (d, *J* = 7.1 Hz, 1H), 2.85 – 2.67 (m, 2H), 2.54 – 2.41 (m, 1H), 2.41 – 2.32 (m, 1H). **^{13}C NMR (126 MHz, $CDCl_3$)** δ 146.2 (d, *J*= 30.4 Hz), 140.8 (d, *J* = 6.5 Hz), 137.2 (d, *J* = 12.3 Hz), 136.7 (d, *J* = 6.4 Hz), 136.6 (d, *J* = 5.6 Hz), 135.8, 134.1 (d, *J* = 7.4 Hz), 134.0 (d, *J* = 7.1 Hz), 133.7, 131.3 (d, *J* = 2.9 Hz), 130.1 (d, *J* = 5.0 Hz), 128.9 (d, *J* = 8.7 Hz), 128.7 (d, *J* = 6.8 Hz), 128.6 (d, *J* = 28.0 Hz), 128.4, 128.2 (d, *J* = 25.1 Hz), 126.4, 119.6, 29.3 (d, *J* = 3.9 Hz), 18.1 (d, *J* = 2.4 Hz). **^{31}P NMR (202 MHz, $CDCl_3$)** δ -13.74.**IR (cm^{-1}, neat)**: 3053, 2949, 1601, 1434, 1090, 745, 697. **HRMS-ESI**: m/z calculated for $C_{27}H_{23}NP^+$ [M + H$^+$] 392.1563, found 392.1560.

Methyl 3-(2'-(diphenylphosphanyl)-6-methyl-[1,1'-biphenyl]-2-yl)butanoate(3bg)

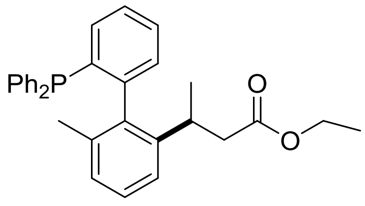

3bg

Following the modified general procedure, the reaction of **1b** (70.4mg, 0.20 mmol), **2g** (68.5 mg, 0.60 mmol), $[Rh(cod)Cl]_2$ (5.0 mg, 0.01mmol), $NaHCO_3$ (83.0 mg, 0.60 mmol) in toluene (1.0 mL) at 150 ℃ under Ar after 24 h, purification by column chromatographyon silica gel (petroleum-ether : ethyl acetate = 30:1) affording 55.6 mg (60%) of **3bg** as colorless oil. **^{1}H NMR (500 MHz, $CDCl_3$)** δ 7.43 (t, *J* = 7.4 Hz, 1H), 7.38 – 7.31 (m, 2H), 7.31 – 7.25 (m, 7H), 7.25 – 7.17 (m, 4H), 7.15 (dd, *J* = 8.1, 3.7 Hz, 1H), 7.09 (d, *J* = 7.7 Hz, 1H), 7.02 (d, *J* = 7.4 Hz, 1H), 4.19 – 3.99 (m, 2H), 3.04 – 2.93 (m, 1H), 2.46 (dd, *J* = 15.7, 12.0 Hz, 1H), 2.28 (d, *J* = 14.9 Hz, 1H), 1.63 (s, 3H), 1.24 (t, *J* = 7.1 Hz, 3H), 1.08 (d, *J* = 6.7 Hz, 3H). ^{13}C NMR (126 MHz, $CDCl_3$) δ 172.7, 146.2 (d, *J* = 33.2 Hz), 144.4, 139.6 (d, *J* = 7.3 Hz), 137.3 (d, *J* = 3.6 Hz), 137.2 (d, *J* = 2.7 Hz), 136.9 (d, *J* = 1.8 Hz), 136.6 (d, *J* = 12.4 Hz), 134.4 (d, *J* = 21.2 Hz), 134.3 (d, *J* = 1.8 Hz), 133.3 (d, *J* = 19.0 Hz), 129.9 (d, *J* = 5.8 Hz), 129.2, 128.7, 128.4 (d, *J* = 6.0 Hz), 128.3 (d, *J* = 7.3 Hz), 128.0 (d, *J* = 5.8 Hz), 127.5 (d, *J* = 1.6 Hz), 122.9, 60.0, 40.9 (d, *J* = 3.8 Hz), 32.5 (d, *J* = 1.8 Hz), 22.5, 20.9 (d, *J* = 2.9 Hz), 14.3. **^{31}P NMR (202 MHz, $CDCl_3$)** δ -15.11.**IR (cm^{-1}, neat)**: 3056, 2975, 1736, 1516, 1434, 1370, 1167, 745. **HRMS-ESI**: m/z calculated for $C_{31}H_{32}O_2P^+$ [M + H^+] 467.2134, found 467.2138.

General Procedure for Hydroarylation of Alkynes Phosphine Ligands

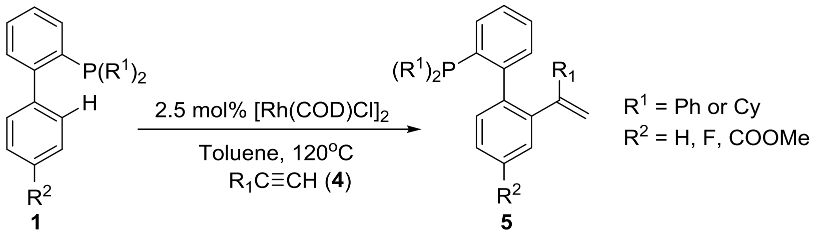

To a 25 mL Schlenk tube was added monophosphine ligands **1** (0.2 mmol, 1.0 equiv), Alkynes **4** (0.4 – 0.8 mmol, 2.0 – 4.0 equiv), $[Rh(cod)Cl]_2$ (0.005 mmol, 2.5%). The tube was purged with Ar three times, followed by addition of toluene (1.0 mL). The mixture was stirred at 120°C for 12 h to 48 h. The solution was then cooled to room temperature and the solvent was removed under vacuum directly. The crude

product was purified by column chromatography on silica gel affording the pure products **5**.

(2'-(1-(4-Methoxyphenyl)vinyl)-[1,1'-biphenyl]-2-yl)diphenylphosphane (5aa)

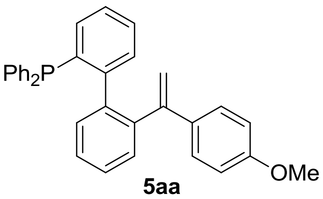

5aa

The reaction of **1a** (67.6 mg, 0.20 mmol), **4a** (52.8 mg, 0.40 mmol), $[Rh(cod)Cl]_2$ (2.5 mg, 0.005 mmol) in toluene (0.5 mL) at 120 ℃ under Ar after 12 h, purification by column chromatography on silica gel (petroleum-ether : ethyl acetate = 200:1) affording 79.9 mg (85%) of **5aa** as a colorless liquid. **^{1}H NMR (400 MHz, $CDCl_3$) δ** 7.40-7.35 (m, 2H), 7.33-7.31 (m, 1H), 7.27-7.24 (m, 2H), 7.21-7.19 (m, 3H), 7.12-7.10 (m, 4H), 7.05-7.02 (m, 2H), 6.99-6.96 (m, 3H), 6.90-6.86 (m, 2H),6.76-6.74 (m, 1H), 6.68-6.66 (m, 2H), 5.42 (d, J = 1.6 Hz, 1H), 5.18 (d, J = 1.6 Hz, 1H), 3.74 (s, 3H). **^{13}C NMR (101 MHz, $CDCl_3$) δ** 158.9 , 149.1 , 147.4 (d, J = 32.4 Hz), 141.1, 140.4, 139.0 (d, J = 13.5 Hz), 137.9 (d, J = 12.5 Hz), 134.1 (d, J = 20.3 Hz), 133.0 (d, J = 18.2 Hz), 131.4 (d, J = 4.8 Hz), 130.6 (d, J = 5.8 Hz), 130.2 , 128.5 , 128.4 (d, J = 7.1 Hz), 128.2 (d, J = 1.7 Hz), 128.0 (d, J = 5.6 Hz), 127.8 (d, J = 14.2 Hz), 127.4, 126.9, 126.3, 115.2, 113.2, 55.2. **^{31}P NMR (162 MHz, $CDCl_3$) δ** -14.2. ATR-FTIR (cm-1): 3028, 3010, 2860, 1700, 1590, 1453, 1155, 668. HRMS (ESI) m/z calcd for $C_{33}H_{28}OP$ $(M + H)^+$ 471.1872, found 471.1879.

(2'-(1-(4-methoxyphenyl)vinyl)-5'-methyl-[1,1'-biphenyl]-2-yl)diphenylphosphine (5ca)

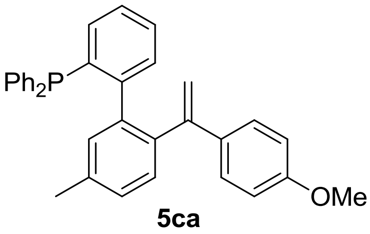

5ca

Following the modified general procedure, the reaction of **8** (87.7 mg, 0.20 mmol), **2a** (87.6 mg, 0.60 mmol), $[Rh(COD)Cl]_2$ (2.5 mg, 0.005 mmol), $NaHCO_3$ (50.4 mg, 0.60 mmol) in toluene (1.0 mL) at 120 ℃ under Ar after 24h, purification by column chromatographyon silica gel (petroleum-ether : ethyl acetate = 250:1) affording 63.3 mg (58%) of **5ca** as colorless oil. **^{1}H NMR (500 MHz, $CDCl_3$)** δ 7.38 – 7.27 (m, 6H), 7.24 – 7.18 (m, 3H), 7.15 – 7.03 (m, 6H), 6.97 (dd, *J* = 4.9, 2.1 Hz, 1H), 6.91 – 6.85

(m, 2H), 6.70 – 6.65 (m, 2H), 6.44 (d, *J* = 1.7 Hz, 1H), 5.39 (d, *J* = 1.6 Hz, 1H), 5.17 (d, *J* = 1.7 Hz, 1H), 3.74 (s, 3H), 2.06 (s, 3H). **^{13}C NMR (126 MHz, $CDCl_3$)** δ 158.8, 149.0, 147.4 (d, *J* = 32.0 Hz), 140.0 (d, *J* = 7.2 Hz), 138.9 (d, *J* = 13.2 Hz), 138.1, 138.0, 137.9, 136.0 (d, *J* = 12.6 Hz), 135.8, 134.5, 134.4 (d, *J* = 20.7 Hz), 133.9 (d, *J* = 2.2 Hz), 133.0 (d, *J* = 18.0 Hz), 132.6 (d, *J* = 4.7 Hz), 130.5 (d, *J* = 5.6 Hz), 130.1, 128.7, 128.4 (d, *J* = 7.4 Hz), 128.3 (d, *J* = 1.8 Hz), 128.0, 127.96 , 127.7 (d, *J* = 16.2 Hz), 126.8, 114.9 , 113.2, 55.2, 20.8.**^{31}P NMR (162 MHz, Chloroform-*d*)** δ -13.6. **IR (cm^{-1}, neat)**: 3046, 2850, 2810, 1726, 1500, 1435, 1100, 742, 697. **HRMS-ESI**: m/z calculated for $C_{34}H_{30}OP^+$ [M + H^+] 485.2029, found 485.2032.

(4'-Fluoro-2'-(1-(4-methoxyphenyl)vinyl)-[1,1'-biphenyl]-2-yl)diphenylphospha (5fa)

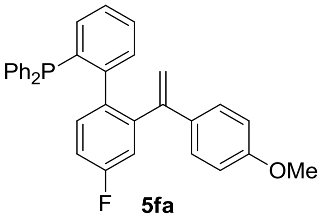

5fa

The reaction of **1e** (71.3 mg, 0.20 mmol), **4a** (105.6 mg, 0.80 mmol), $[Rh(cod)Cl]_2$ (2.5 mg, 0.005 mmol) in toluene (0.5 mL) at 120 ℃ under Ar after 12 h, purification by column chromatography on silica gel (petroleum-ether : ethyl acetate = 200:1) affording 51.8 mg (53%) of **5fa** as a yellow liquid. **^{1}H NMR (400 MHz, $CDCl_3$) δ** 7.35 – 7.28 (m, 3H), 7.26-7.20 (m, 5H), 7.12 – 7.06 (m, 3H), 7.06 – 6.99 (m, 3H), 6.98 – 6.93 (m, 1H), 6.91 – 6.87 (m, 2H), 6.80– 6.75 (m, 1H), 6.69 – 6.67 (m, 3H), 5.43 (d, J = 1.4 Hz, 1H), 5.20 (d, J = 1.4 Hz, 1H), 3.74 (s, 3H).**^{13}C NMR (101 MHz, $CDCl_3$) δ** 162.0 (d, J = 242.4 Hz), 159.1, 148.2, 146.3 (d, J = 32.1 Hz), 143.2 (d, J = 7.7 Hz), 138.7 (d, J = 13.2 Hz), 137.6 (d, J = 12.3 Hz), 136.2 (d, J = 12.5 Hz), 134.2 (d, J = 11.9 Hz), 134.0, 133.1 (d, J = 18.4 Hz), 132.8, 130.8 (d, J = 5.9 Hz), 128.7, 128.5 (d, J = 7.2 Hz), 128.3, 127.9 (d, J = 9.8 Hz), 127.2, 117.0 (d, J = 21.4 Hz), 115.7, 113.3, 113.1 (d, J = 20.8 Hz), 55.2. **^{31}P NMR (162 MHz, $CDCl_3$) δ** -15.5. **^{19}F NMR (376 MHz, $CDCl_3$) δ** -115.35. ATR-FTIR (cm-1): 3053, 3001, 2955, 2836, 2248, 1540, 1034, 698.HRMS (ESI) m/z calcd for $C_{33}H_{27}FOP$ (M + $H)^+$ 489.1778, found 489.1785.

(2-(3-(1-(4-methoxyphenyl)vinyl)naphthalen-2-yl)phenyl)diphenylphosphane (5ga)

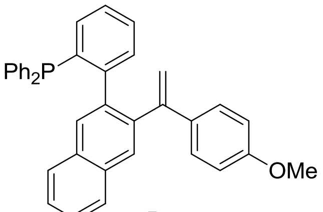

5ga

The reaction of **1f** (77.6 mg, 0.20 mmol), **4a** (105.6 mg, 0.80 mmol), [Rh(cod)Cl]$_2$ (2.5 mg, 0.005 mmol) in toluene (0.5 mL) at 120 ℃ under Ar after 12 h, purification by column chromatography on silica gel (petroleum-ether : ethyl acetate = 200:1) affording 75.0 mg (72%) of **5ga** as a white solid. **^{1}H NMR (400 MHz, CDCl$_3$) δ** 7.91 – 7.85 (m, 2H), 7.48 – 7.35 (m, 5H), 7.29 –7.25 (m, 3H), 7.21 – 7.19 (m, 3H), 7.16 – 7.10 (m, 3H), 7.04 – 6.96 (m, 4H), 6.85 –6.84 (m, 2H), 6.68 – 6.63 (m, 2H), 5.48 (d, J = 1.7 Hz, 1H), 5.36 (d, J = 1.7 Hz, 1H), 3.74 (s, 3H). **^{13}C NMR (101 MHz, CDCl$_3$) δ** 158.8, 149.6, 139.8, 139.0, 134.6, 134.5, 134.3, 133.8 (d, J = 1.9 Hz), 133.0, 132. 9, 132. 7, 131.8, 130.9, 128.9, 128.8, 128.5 (d, J = 7.6 Hz), 128.3 (d, J = 1.8 Hz), 128.0 (d, J = 5.3 Hz), 127.8 , 127.7 (d, J = 3.1 Hz), 127.6, 127.0, 126.1, 126.0, 115.4, 113.2, 55.2. **^{31}P NMR (162 MHz, CDCl$_3$) δ**-14.2.ATR-FTIR (cm-1): 3001, 2927, 2834, 1734, 1247, 1437, 744, 668. HRMS (ESI) m/z calcd for $C_{37}H_{30}OP$ $(M + H)^+$ 521.2029, found 521.2024.

Dicyclohexyl(2'-(1-(4-methoxyphenyl)vinyl)-[1,1'-biphenyl]-2-yl)phosphane (5ha)

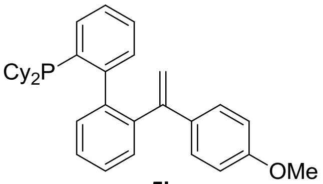

5ha

The reaction of **1h** (70.1 mg, 0.20 mmol) and [Rh(cod)Cl]$_2$ (2.5 mg, 0.005 mmol) in toluene (0.5 mL) at 120 ℃ under Ar. Then **4a** (4 × 1 equiv/2h) was batch-wise added. After 24 h, purification by column chromatography on silica gel (petroleum-ether : ethyl acetate = 200:1) affording 74.4 mg (77%) of **5ha** as a colorless liquid. **^{1}H NMR (400 MHz, CDCl$_3$) δ** 7.31 – 7.20 (m, 4H), 7.07 (m, 2H), 6.93 (d, J = 8.6 Hz, 3H), 6.80 (m, 1H), 6.62 – 6.57 (m, 2H), 5.22 (d, J = 1.6 Hz, 1H), 4.95 (s, 1H), 3.67 (s, 3H), 1.69 – 1.49 (m, 11H), 1.16 – 0.89 (m, 10H), 0.81 – 0.75 (m, 1H). **^{13}C NMR (101 MHz, CDCl$_3$) δ** 158.9, 149.1 (d, J = 30.4 Hz), 148.1, 141.4, 134.9, 134.5 (d, J = 20.2 Hz), 132.2 (d, J = 3.1 Hz), 131.9 (d, J = 1.8 Hz), 130.9 (d, J = 6.1 Hz), 130.7 (d, J = 4.0 Hz), 130.0, 128.6, 127.4, 127.0, 126.2, 125.9, 115.9, 113.1, 55.3, 36.7 (d, J = 16.3 Hz), 33.3 (d, J = 13.1 Hz), 30.9 (d, J = 14.2 Hz), 30.3 (d, J = 18.5 Hz), 29.6 (d, J = 12.4 Hz), 29.1 (d, J = 5.7 Hz), 27.8 (d, J = 4.4 Hz), 27.7, 27.4 (d, J = 9.4 Hz), 27.2 (d, J = 11.1 Hz), 26.5 (d, J = 10.1 Hz). **^{31}P NMR (162 MHz, CDCl$_3$)**

δ -14.0. ATR-FTIR (cm-1): 2924, 2830, 2590, 1760, 1700, 1653, 1509, 809. HRMS (ESI) m/z calcd for $C_{33}H_{39}OPNa$ (M + Na)$^+$ 505.2631, found 505.2631.

2-(Diphenylphosphanyl)-1-(2-(1-(4-methoxyphenyl)vinyl)phenyl)-1H-pyrrole (5la)

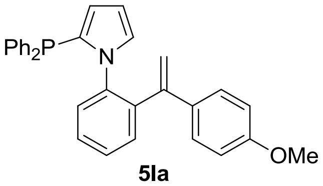

5la

The reaction of **1k** (65.4 mg, 0.20 mmol) and $[Rh(cod)Cl]_2$ (2.5 mg, 0.005 mmol) in toluene (0.5 mL) at 120 ℃ under Ar. Then **4a** (4 × 1 equiv/2h) was batch-wise added. After 24 h, purification by column chromatography on silica gel (petroleum-ether : ethyl acetate = 200:1) affording 50.6 mg (55%) of **5la** as a colorless liquid. **^{1}H NMR (400 MHz, $CDCl_3$) δ** 7.37 – 7.27 (m, 3H), 7.26 – 7.19 (m, 9H), 7.15 –7.14 (m, 3H), 7.06 – 7.02 (m, 2H), 6.80 (d, J = 9.5 Hz, 1H), 6.74-6.72 (m, 1H), 6.64 (d, J = 8.8 Hz, 2H), 6.04 –6.02 (m, 1H), 5.88 –5.57 (m, 1H), 5.37 (d, J = 1.3 Hz, 1H), 5.09 (d, J = 1.3 Hz, 1H), 3.67 (s, 3H). **^{13}C NMR (101 MHz, $CDCl_3$) δ** 158.1 , 145.8 , 138.6 , 137.6 , 132.1 , 130.2 , 127.9 (d, J = 4.8 Hz), 127.7, 127.6, 127.5, 127.2, 127.1, 127.0, 126.9, 126.8 (d, J = 1.8 Hz), 126.6 (d, J = 2.3 Hz), 117.8, 113.9, 112.2, 108.0, 54.1. **^{31}P NMR (162 MHz, $CDCl_3$) δ** -31.0. ATR-FTIR (cm-1): 2925, 2850, 1607, 1510, 1449, 1248, 1178, 835. HRMS (ESI) m/z calcd for $C_{31}H_{27}NOP$ (M + H)$^+$ 460.1825, found 460.1829.

2-(Dicyclohexylphosphanyl)-1-(2-(1-(4-methoxyphenyl)vinyl)phenyl)-1H-pyrrole (5ma)

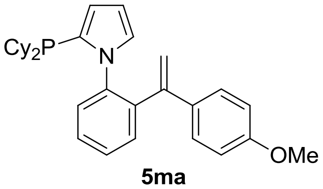

5ma

The reaction of **1l** (68.0 mg, 0.20 mmol) and $[Rh(cod)Cl]_2$ (2.5 mg, 0.005 mmol) in toluene (0.5 mL) at 120 ℃ under Ar. Then **4a** (4 × 1 equiv/2h) was batch-wise added. After 24 h, purification by column chromatography on silica gel (petroleum-ether : ethyl acetate = 300:1) affording 54.8 mg (58%) of **5ma** as a colorless liquid. **^{1}H NMR (400 MHz, $CDCl_3$) δ** 7.42 – 7.37 (m, 2H), 7.36 – 7.32 (m, 1H), 7.28 – 7.26 (m, 1H), 7.18 – 7.14 (m, 2H), 6.80 – 6.74 (m, 2H), 6.64– 6.61 (m, 1H), 6.39– 6.38 (m, 1H), 6.13– 6.12 (m, 1H), 5.39 (d, J = 1.3 Hz, 1H), 5.04 (d, J = 1.3 Hz, 1H), 3.81 (s, 3H), 1.69 (dd, J = 39.4, 10.9 Hz, 11H), 1.18 (dq, J = 35.5, 12.6 Hz, 10H), 0.90 (dd, J = 13.4, 6.2 Hz, 1H). **^{13}C NMR (101 MHz, $CDCl_3$) δ** 159.1, 145.6,

140.2, 139.3 (d, J = 1.8 Hz), 134.0, 131.0, 130.3 (d, J = 3.4 Hz), 128.3 (d, J = 1.7 Hz), 127.9, 127.8, 127.3, 126.7 (d, J = 1.8 Hz), 116.1 (d, J = 4.6 Hz), 115.1, 113.2, 108.1, 55.2, 34.4 (d, J = 8.3 Hz), 30.3 (d, J = 16.2 Hz), 29.0 (d, J = 7.2 Hz), 27.4 (d, J = 8.3 Hz), 27.2 (d, J = 2.9 Hz), 27.1, 26.9, 26.5. **^{31}P NMR (162 MHz, CDCl$_3$) δ** -27.6.ATR-FTIR (cm-1): 2924, 2830, 2590, 1760, 1700, 1653, 1509, 809.HRMS (ESI) m/z calcd for $C_{31}H_{38}NNaOP$ $(M + Na)^+$ 494.2583, found 494.2591.

Diphenyl(2'-(1-phenylvinyl)-[1,1'-biphenyl]-2-yl)phosphane (5ab)

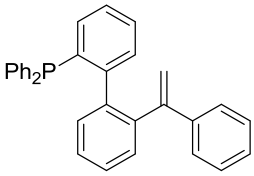

5ab

The reaction of **1a** (67.6 mg, 0.20 mmol) and [Rh(cod)Cl]$_2$ (2.5 mg, 0.005 mmol) in toluene (0.5 mL) at 120 ℃ under Ar. Then **4b** (4 × 1 equiv/2h) was batch-wise added. After 24 h, purification by column chromatography on silica gel (petroleum-ether) affording 70.5 mg (80%) of **5ab** as a colorless oil. **^{1}H NMR (400 MHz, CDCl$_3$) δ** 7.34 – 7.28 (m, 2H), 7.25 – 7.23 (m, 3H), 7.18 – 7.12 (m, 5H), 7.09 – 6.99 (m, 8H), 6.96 – 6.92 (m, 1H), 6.90 – 6.82 (m, 3H), 6.70 – 6.67 (m, 1H), 5.42 (d, J = 1.5 Hz, 1H), 5.21 (d, J = 1.5 Hz, 1H). **^{13}C NMR (101 MHz, CDCl$_3$) δ** 148.6, 140.7, 139.8, 137.8 (d, J = 13.3 Hz), 136.8 (d, J = 12.5 Hz), 135.0 (d, J = 12.6 Hz), 133.0 (d, J = 2.0 Hz), 132.9, 132.2, 132.0, 130.3 (d, J = 4.6 Hz), 129.5 (d, J = 5.8 Hz), 129.3, 127.4 (d, J = 10.6 Hz), 127.3, 127.0 (d, J = 5.6 Hz), 126.8, 126.8, 126.4, 126.0 (d, J = 3.2 Hz), 125.4, 115.8 (d, J = 2.2 Hz). **^{31}P NMR (162 MHz, CDCl$_3$) δ** -14.1. ATR-FTIR (cm-1): 2964, 2246, 1955, 1261, 1089, 1027, 743. HRMS (ESI) m/z calcd for $C_{32}H_{26}P$ $(M + H)^+$ 441.1767, found 441.1762.

Diphenyl(2'-(1-(p-tolyl)vinyl)-[1,1'-biphenyl]-2-yl)phosphane (5ac)

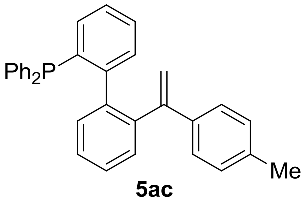

5ac

The reaction of **1a** (67.6 mg, 0.20 mmol) and [Rh(cod)Cl]$_2$ (2.5 mg, 0.005 mmol) in toluene (0.5 mL) at 120 ℃ under Ar. Then **4c** (4 × 1 equiv/2h) was batch-wise added. After 24 h, purification by column chromatography on silica gel (petroleum-ether) affording 74.4 mg (82%) of **5ac** as a colorless oil. **^{1}H NMR (400 MHz, CDCl$_3$) δ** 7.40 – 7.37 (m, 1H), 7.33 – 7.27 (m, 5H), 7.22 – 7.18 (m, 4H), 7.12 –

7.06 (m, 4H), 7.01– 6.99 (m, 2H), 6.97 – 6.91 (m, 3H), 6.89 – 6.85 (m, 2H), 6.75 – 6.73 (m, 1H), 5.46 (d, J = 1.6 Hz, 1H), 5.22 (d, J = 1.6 Hz, 1H), 2.29 (s, 3H). **^{13}C NMR (101 MHz, $CDCl_3$) δ** 149.5, 141.0, 138.8, 136.8, 134.2 (d, J = 3.7 Hz), 134.1, 134.0 (d, J = 2.7 Hz), 133.8, 133.1 (d, J = 18.2 Hz), 131.4 (d, J = 4.7 Hz), 130.7 (d, J = 5.9 Hz), 130.3, 129.7 (d, J = 3.9 Hz), 128.6, 128.4 (d, J = 4.8 Hz), 128.3 (d, J = 4.2 Hz), 128.02 (d, J = 5.5 Hz), 127.7, 127.4, 126.9 (d, J = 1.4 Hz), 126.3, 115.9, 21.1. **^{31}P NMR (162 MHz, $CDCl_3$) δ** -13.4. ATR-FTIR (cm-1): 3022, 2920, 2862, 2361, 1734, 1457, 909, 503. HRMS (ESI) m/z calcd for $C_{33}H_{28}P$ $(M + H)^+$ 455.1923, found 455.1928.

(2'-(1-([1,1'-biphenyl]-4-yl)vinyl)-[1,1'-biphenyl]-2-yl)diphenylphosphane (5ad)

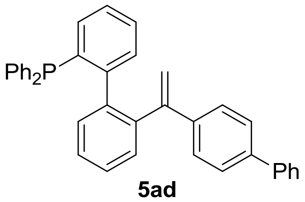

5ad

The reaction of **1a** (67.6 mg, 0.20 mmol), **4d** (106.8 mg, 0.60 mmol), $[Rh(cod)Cl]_2$ (2.5 mg, 0.005 mmol) in toluene (0.5 mL) at 120 ℃ under Ar after 12 h, purification by column chromatography on silica gel (petroleum-ether) affording 87.7 mg (85%) of **5ad** as a white solid. **^{1}H NMR (400 MHz, $CDCl_3$) δ** 7.57 – 7.53 (m, 2H), 7.46 – 7.38 (m, 6H), 7.33 – 7.31 (m, 4H), 7.24 – 7.22 (m, 2H), 7.17 – 7.15 (m, 3H), 7.12 – 7.04 (m, 6H), 6.93 – 6.90 (m, 1H), 6.88 – 6.82 (m, 2H), 6.75 – 6.73 (m, 1H), 5.55 (d, J = 1.5 Hz, 1H), 5.32 (d, J = 1.5 Hz, 1H). **^{13}C NMR (101 MHz, $CDCl_3$) δ** 149.5, 140.7, 140.4 (d, J = 7.3 Hz), 139.7, 138.8 (d, J = 13.5 Hz), 134.2, 134.0 (d, J = 2.2 Hz), 134.0, 133.0 (d, J = 18.2 Hz), 131.4 (d, J = 4.3 Hz), 130. 8 (d, J = 5.9 Hz), 130.3, 128.7, 128.6, 128.4, 128.4, 128.1 (d, J = 5.7 Hz), 127.9, 127.68, 127.5, 127.5, 127.2, 127.0, 126.9, 126. 5, 116.7. **^{31}P NMR (162 MHz, $CDCl_3$) δ** -14.2. ATR-FTIR (cm-1): 3001, 2836, 2361, 1734, 1204, 1154, 743, 668. HRMS (ESI) m/z calcd for $C_{38}H_{30}P$ $(M + H)^+$ 517.2080, found 517.2086.

(2'-(1-(3-chloro-4-methoxyphenyl)vinyl)-[1,1'-biphenyl]-2-yl)diphenylphosphane (5ae)

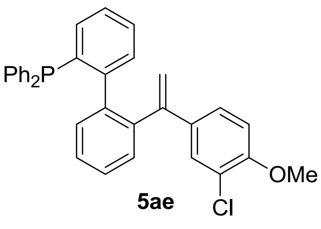

5ae

The reaction of **1a** (67.6 mg, 0.20 mmol), **4e** (99.6 mg,

0.60 mmol), [Rh(cod)Cl]$_2$ (2.5 mg, 0.005 mmol) in toluene (0.5 mL) at 120 ℃ under Ar after 12 h, purification by column chromatography on silica gel (petroleum-ether : ethyl acetate = 200:1) affording 58.4 mg (58%) of **5ae** as a colorless oil. **^{1}H NMR (400 MHz, CDCl$_3$) δ** 7.37 – 7.34 (m, 2H), 7.33 – 7.27 (m, 3H), 7.25 – 7.21 (m, 5H), 7.13–7.11 (m, 4H), 7.04 – 6.97 (m, 2H), 6.95 – 6.87 (m, 3H), 6.77 – 6.75 (m, 1H), 6.65 (d, J = 8.6 Hz, 1H), 5.41 (d, J = 1.4 Hz, 1H), 5.23 (d, J = 1.4 Hz, 1H), 3.81 (s, 3H). **^{13}C NMR (101 MHz, CDCl$_3$) δ** 154.1 , 148.1 , 147.2 (d, J = 32.0 Hz), 140.5, 138.7 (d, J = 13.4 Hz), 137.9 (d, J = 12.9 Hz), 136.0 (d, J = 12.6 Hz), 135.3, 134.1, 134.0, 133.0 (d, J = 18.5 Hz), 131.4 (d, J = 4.5 Hz), 130.6 (d, J = 5.8 Hz), 130.1, 128.6(d, J = 4.1 Hz), 128.4 (d, J = 7.1 Hz), 128.1 (d, J = 5.5 Hz), 127.9 (d, J = 10.4 Hz), 127.6, 127.1, 126.6, 121.8, 116.3, 111.2, 56.1.**^{31}P NMR (162 MHz, CDCl$_3$) δ** -14.0. ATR-FTIR (cm-1): 2360, 1734, 1700, 1576, 1521, 1507, 1457, 749. HRMS (ESI) m/z calcd for $C_{33}H_{27}ClOP$ $(M + H)^+$ 505.1483, found 505.1502.

Diphenyl(2'-(1-(thiophen-3-yl)vinyl)-[1,1'-biphenyl]-2-yl)phosphane (5af)

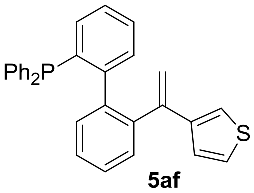

5af

The reaction of **1a** (67.6 mg, 0.20 mmol), **4f** (64.8 mg, 0.60 mmol), [Rh(cod)Cl]$_2$ (2.5 mg, 0.005 mmol) in toluene (0.5 mL) at 120 ℃ under Ar after 12 h, purification by column chromatography on silica gel (petroleum-ether) affording 54.4 mg (61%) of **5af** as a yellow oil. **^{1}H NMR (400 MHz, CDCl$_3$) δ** 7.43 – 7.32 (m, 5H), 7.29 – 7.25 (m, 5H), 7.18 – 7.10 (m, 5H), 7.07 – 7.03 (m, 4H), 6.98 (dd, J = 3.0, 1.2 Hz, 1H), 6.84 – 6.82 (m, 1H), 5.54 (d, J = 1.4 Hz, 1H), 5.18 (d, J = 1.4 Hz, 1H). **^{13}C NMR (101 MHz, CDCl$_3$) δ** 147.4 (d, J = 32.4 Hz), 143.7, 143.3, 140.6, 140.3 (d, J = 6.9 Hz), 138.7 (d, J = 13.3 Hz), 137.9 (d, J = 12.1 Hz), 136.0 (d, J = 12.2 Hz), 134.3 (d, J = 2.3 Hz), 134.0 (d, J = 19.9 Hz), 133.2 (d, J = 18.8 Hz), 131.4 (d, J = 4.6 Hz), 130.4 (d, J = 5.8 Hz), 129.8, 128.4 (d, J = 7.1 Hz), 128.1 (d, J = 5.8 Hz), 127.9 (d, J = 1.7 Hz), 127.3 (d, J = 44.0 Hz), 126.2 (d, J = 33.0 Hz), 125.0, 123.0 (d, J = 3.6 Hz), 115.6 (d, J = 2.3 Hz). **^{31}P NMR (162 MHz, CDCl$_3$) δ** -14.5. ATR-FTIR (cm-1): 2932, 2863, 1631, 1584, 1433, 1300, 1104, 742. HRMS (ESI) m/z calcd for $C_{30}H_{24}PS$ $(M + H)^+$ 447.1331, found 447.1337.

Diphenyl(2'-(4-phenylbut-1-en-2-yl)-[1,1'-biphenyl]-2-yl)phosphane (5ag)

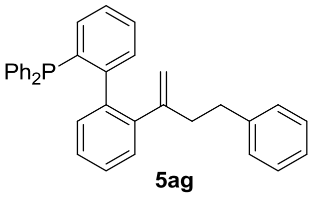

5ag

The reaction of **1a** (67.6 mg, 0.20 mmol) and $[Rh(cod)Cl]_2$ (2.5 mg, 0.005 mmol) in toluene (0.3 mL) at 120 ℃ under Ar. Then **4g** (4 × 1 equiv/2h) was batch-wise added. After 48 h, purification by column chromatography on silica gel (petroleum-ether) affording 48.6 mg (52%) of **5ag** as a yellow oil. **^{1}H NMR (400 MHz, $CDCl_3$) δ** 7.28 – 7.21 (m, 6H), 7.20 – 7.15 (m, 4H), 7.14 – 7.03 (m, 7H), 6.96 – 6.85 (m, 5H), 6.67 – 6.63 (m, 1H), 5.02 – 5.00 (m, 2H), 2.62 – 2.54 (m, 1H), 2.50 – 2.42 (m, 1H), 2.30 – 2.14 (m, 2H). **^{13}C NMR (101 MHz, $CDCl_3$) δ** 150.1, 147.8 (d, J = 32.5 Hz), 141.9, 139.1 (d, J = 7.2 Hz), 138.5 (d, J = 12.8 Hz), 137.6 (d, J = 12.1 Hz), 136.4 (d, J = 12.7 Hz), 134.6 (d, J = 2.1 Hz), 134.1, 133.9, 133.3, 133.2, 131.2 (d, J = 5.0 Hz), 130.7 (d, J = 5.8 Hz), 129.4, 128.4 (d, J = 6.8 Hz), 128.2 (d, J = 5.2 Hz), 127.9, 127.5 (d, J = 25.3 Hz), 126.0, 125.6, 115.7, 37.9, 34.5. **^{31}P NMR (162 MHz, $CDCl_3$) δ** -14.7. ATR-FTIR (cm-1): 2923, 2853, 1721, 1201, 1116, 746, 720, 543. HRMS (ESI) m/z calcd for $C_{34}H_{30}P$ $(M + H)^+$ 469.2080, found 469.2077.

Diphenyl(2'-(4-phenylbut-1-en-2-yl)-[1,1'-biphenyl]-2-yl)phosphane(5ah)

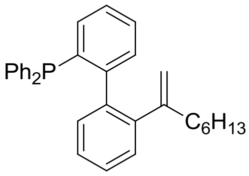

5ah

The reaction of **1a** (67.6 mg, 0.20 mmol) and $[Rh(cod)Cl]_2$ (2.5 mg, 0.005 mmol) in toluene (0.3 mL) at 120 ℃ under Ar. Then **4h** (4 × 1 equiv/2h) was batch-wise added. After 48 h, purification by column chromatography on silica gel (petroleum-ether) affording 51.1 mg (57%) of **5ah** as a colorless oil.**^{1}H NMR (400 MHz, $CDCl_3$) δ** 7.34 – 7.31 (m, 4H), 7.30 – 7.26 (m, 8H), 7.17 – 7.10 (m, 4H), 7.04 – 7.01 (m, 1H), 6.74 – 6.73 (m, 1H), 5.05 – 4.97 (m, 2H), 2.00 – 1.90 (m, 2H), 1.22 – 1.11 (m, 8H), 0.82 (t, J = 3.6 Hz, 3H). **^{13}C NMR (101 MHz, $CDCl_3$) δ** 150.7 , 142.3 , 139.1 (d, J = 7.2 Hz), 136.2 (d, J = 12.6 Hz), 134.6 (d, J = 2.1 Hz), 133.9 (d, J = 19.9 Hz), 133.3 (d, J = 18.9 Hz), 131.1 (d, J = 4.6 Hz), 130.6 (d, J = 5.6 Hz), 129.2, 128.5, 128.4 (d, J = 6.9 Hz), 128.2, 128.2, 128.0, 127.3 (d, J = 16.4 Hz), 125.7, 115.1, 36.5, 31.7, 28.9, 27.9, 22.7, 14.1. **^{31}P NMR (162 MHz, $CDCl_3$) δ** -14.9.ATR-FTIR (cm-1): 2961, 2864, 1631, 1478, 1433, 1384, 907, 742.HRMS (ESI) m/z calcd for $C_{32}H_{34}P$ (M

+ H)$^+$ 449.2393, found 449.2394.

(2'-(3,3-dimethylbut-1-en-2-yl)-[1,1'-biphenyl]-2-yl)diphenylphosphane(5ai)

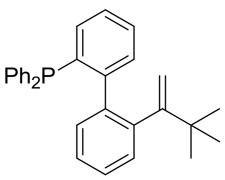

5ai

The reaction of **1a** (67.6 mg, 0.20 mmol) and $[Rh(cod)Cl]_2$ (2.5 mg, 0.005 mmol) in toluene (0.3 mL) at 120 ℃under Ar. Then **4i** (4 × 1 equiv/2h) was batch-wise added. After 48 h, purification by column chromatography on silica gel (petroleum-ether) affording 31.9 mg (38%) of **5ai**as a colorless oil. **^{1}H NMR (400 MHz, $CDCl_3$) δ** 7.40 – 7.28 (m, 7H), 7.26 – 7.19 (m, 6H), 7.17 – 7.14 (m, 1H), 7.11 – 7.04 (m, 2H), 7.01 – 6.95 (m, 1H), 6.75 – 6.70 (m, 1H), 5.30 (s, 1H), 5.14 (s, 1H), 0.98 (s, 9H). **^{13}C NMR (101 MHz, $CDCl_3$) δ** 134.8, 134.1 (d, J = 20.0 Hz), 133.2 (d, J = 18.4 Hz), 131.9, 130.6, 130.4, 128.6, 128.5, 128.4, 128.2 (d, J = 6.9 Hz), 127.9, 127.7, 127.0, 126.6, 125.06, 115.3, 31.5, 30.9. **^{31}P NMR (162 MHz, $CDCl_3$) δ** -14.7. ATR-FTIR (cm-1): 2961, 2864, 1631, 1478, 1433, 1384, 907, 742. HRMS (ESI) m/z calcd for $C_{30}H_{30}P$ (M + H)$^+$ 421.2080, found 421.2087.

(E)-diphenyl(2'-(2-(triisopropylsilyl)vinyl)-[1,1'-biphenyl]-2-yl)phosphane(7)

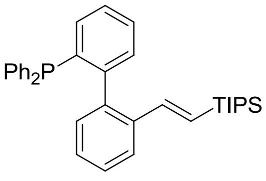

7

The reaction of **1a** (67.6 mg, 0.20 mmol), **6** (109.2 mg, 0.60 mmol), $[Rh(cod)Cl]_2$ (2.5 mg, 0.005 mmol) in toluene (0.5 mL) at 120 ℃ under Ar after 24 h, purification by column chromatography on silica gel (petroleum-ether) affording 62.4 mg (61%) of **7** as a colorless oil. **^{1}H NMR (400 MHz, $CDCl_3$) δ** 7.63 – 7.61 (m, 1H), 7.37 – 7.28 (m, 6H), 7.24 – 7.21 (m, 5H), 7.16 – 6.99 (m, 5H), 6.86 – 6.82 (m, 1H), 6.65 (d, J = 19.5 Hz, 1H), 6.23 (d, J = 19.4 Hz, 1H), 1.10– 1.08 (m, 3H), 0.97– 0.95 (m, 16H), 0.91– 0.87 (m, 2H). **^{13}C NMR (101 MHz, $CDCl_3$) δ** 144.6, 137.6, 137.4, 136.9, 134.1, 133.9 (d, J = 5.1 Hz), 133.6 (d, J = 5.3 Hz), 130.9 (d, J = 4.8 Hz), 130.7 (d, J = 3.8 Hz), 128.5, 128.4, 128.3, 128.2, 128.2, 127.7, 127.5, 126.4, 125.0 (d, J = 7.0 Hz), 18.7, 11.0. **^{31}P NMR (162 MHz, $CDCl_3$) δ** -13.8. ATR-FTIR (cm-1): 2940, 2964, 2349, 1463, 1004, 882, 741, 696. HRMS (ESI) m/z calcd for $C_{35}H_{42}PSi$ (M + H)$^+$ 521.2788, found 521.2802.

General Procedure for Di-Selective Direct Hydroarylation of Phosphine Ligands.

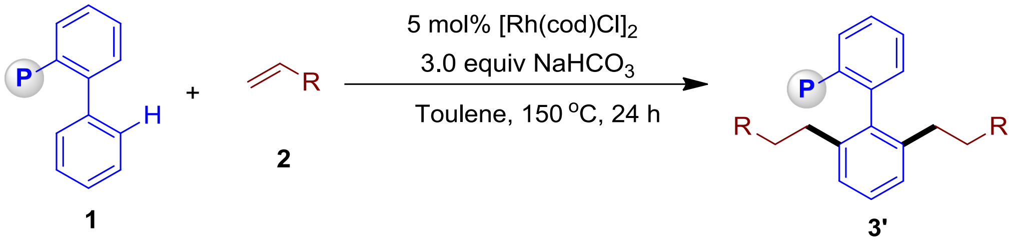

In an oven-dried Schlenk tube, monophosphine ligands **1** (1.0 equiv, 0.20 mmol), alkenes **2** (3.0 equiv, 0.60 mmol), $[Rh(cod)Cl]_2$ (5.0 mol%, 5.0 mg, 0.01 mmol), $NaHCO_3$ (3.0 equiv, 50.4 mg, 0.60 mmol) were dissolved in freshly distilled toluene (1.0 mL). The mixture was stirred at 150 ℃ under argon for 24 hours. Upon the completion of the reaction, the solvent was removed. The crude mixture was directly subjected to column Chromatography on silica gel using petrol ether/ EtOAc as eluent to give the desired products **3'**.

Dimethyl 3,3'-(2'-(diphenylphosphanyl)-[1,1'-biphenyl]-2,6-diyl)dipropionate (3aa')

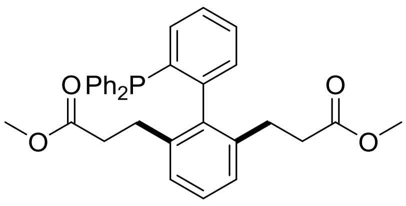

3aa'

Following the general procedure, the reaction of **1a** (67.6 mg, 0.20 mmol), **2a** (51.6 mg, 0.60 mmol), $[Rh(cod)Cl]_2$ (5.0 mg, 0.01mmol), $NaHCO_3$ (50.4 mg, 0.60 mmol) in toluene (1.0 mL) at 150 ℃ under Ar after 24 h, purification by column chromatographyon silica gel (petroleum-ether : ethyl acetate = 10:1) affording 79.6 mg (78%) of **3aa'** as colorless oil. **^{1}H NMR (500 MHz, $CDCl_3$)** δ 7.44 (t, J = 7.4 Hz, 1H), 7.36 (t, J = 7.6 Hz, 1H), 7.34 – 7.27 (m, 8H), 7.21 (td, J = 7.6, 1.7 Hz, 4H), 7.17 (dd, J = 8.5, 4.3 Hz, 1H), 7.08 (d, J = 7.7 Hz, 2H), 3.61 (s, 6H), 2.45 – 2.35 (m, 4H), 2.35 – 2.22 (m, 4H). **^{13}C NMR (126 MHz, $CDCl_3$)** δ 173.4, 145.3 (d, J = 32.8 Hz), 140.2 (d, J = 6.8 Hz), 139.0, 137.3 (d, J = 11.6 Hz), 136.5 (d, J = 11.9 Hz), 134.2, 134.0 (d, J = 20.2 Hz), 130.2 (d, J = 5.7 Hz), 129.2, 128.6, 128.4 (d, J = 6.8 Hz), 128.1, 127.8, 126.0, 51.5, 34.6, 28.6 (d, J = 2.8 Hz). **^{31}P NMR (202 MHz, $CDCl_3$)** δ -15.02.**IR (cm^{-1}, neat)**: 3053, 2955, 1730, 1518, 1463, 1376, 1145, 741. **HRMS-ESI**: m/z calculated for $C_{32}H_{32}O_4P^+$ [M + Na^+] 511.2033, found 511.2039.

Dibutyl 3,3'-(2'-(diphenylphosphanyl)-[1,1'-biphenyl]-2,6-diyl)dipropionate (3ab')

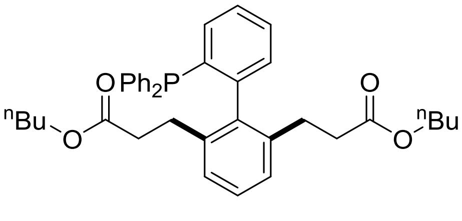

3ab'

Following the general procedure, the reaction of **1a** (67.6 mg, 0.20 mmol), **2b** (51.6 mg, 0.60 mmol), $[Rh(cod)Cl]_2$ (5.0 mg, 0.01mmol), $NaHCO_3$ (50.4 mg, 0.60 mmol) in toluene (1.0 mL) at 150 ℃ under Ar after 24 h, purification by column chromatographyon silica gel (petroleum-ether : ethyl acetate = 10:1) affording 85.6 mg (72%) of **3bi** as colorless oil. **^{1}H NMR (500 MHz, $CDCl_3$)** δ 7.40 (t, *J* = 7.4 Hz, 1H), 7.31 (t, *J* = 7.5 Hz, 1H), 7.29 – 7.22 (m, 8H), 7.21 – 7.12 (m, 5H), 7.04 (d, *J* = 7.7 Hz, 2H), 3.97 (t, *J* = 6.7 Hz, 4H), 2.40 – 2.31 (m, 4H), 2.30 – 2.16 (m, 4H), 1.61 – 1.44 (m, 4H), 1.40 – 1.18 (m, 4H), 0.89 (t, *J* = 7.4 Hz, 6H). **^{13}C NMR (126 MHz, $CDCl_3$)** δ 173.1, 145.4 (d, *J* = 32.8 Hz), 140.2 (d, *J* = 6.9 Hz), 139.1 (d, *J* = 1.7 Hz), 137.4 (d, *J* = 11.8 Hz), 136.6 (d, *J* = 12.0 Hz), 134.1, 134.0, 130.2 (d, *J* = 5.8 Hz), 129.2, 128.6, 128.4 (d, *J* = 6.9 Hz), 128.1, 127.8, 126.1, 64.2, 35.0, 30.7, 28.8 (d, *J* = 2.8 Hz), 19.2, 13.8. **^{31}P NMR (202 MHz, $CDCl_3$)** δ -14.88. **IR (cm^{-1}, neat)**: 3069, 2958, 1741, 1556, 1432, 1380, 1167, 745. **HRMS-ESI**: m/z calculated for$C_{38}H_{43}NaO_4P^+$ [M + Na^+] 617.2791, found 617.2795.

Dimethyl3,3'-(2-(2-(diphenylphosphanyl)-1H-pyrrol-1-yl)-1,3-phenylene)dipropionate (3ka')

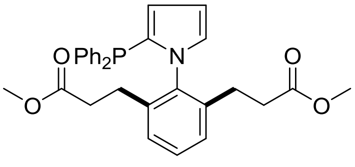

3ka'

Following the general procedure, the reaction of **1k** (65.5mg, 0.20 mmol), **2a** (51.6 mg, 0.60 mmol), $[Rh(cod)Cl]_2$ (5.0 mg, 0.01mmol), $NaHCO_3$ (50.4 mg, 0.60 mmol) in toluene (1.0 mL) at 150 ℃ under Ar after 24 h, purification by column chromatographyon silica gel (petroleum-ether : ethyl acetate = 10:1) affording 81.9 mg (82%) of **3ka'** as colorless oil.**^{1}H NMR (500 MHz, $CDCl_3$)** δ 7.40 – 7.29 (m, 11H), 7.15 (d, *J* = 7.7 Hz, 2H), 6.87 – 6.84 (m, 1H), 6.43 (dd, *J* = 3.6, 2.6 Hz, 1H), 6.33 (dd, *J* = 3.6, 1.5 Hz, 1H), 3.63 (s, 6H), 2.72 – 2.40 (m, 4H), 2.40 – 2.24 (m, 4H).**^{13}C NMR (126 MHz, $CDCl_3$)** δ 173.1, 139.4, 138.0, 137.1,

133.4 (d, *J* = 19.9 Hz), 129.7 (d, *J* = 3.2 Hz), 129.1, 128.5, 128.3 (d, *J* = 7.0 Hz), 126.8, 126.81 (d, *J* = 3.0 Hz), 118.9 (d, *J* = 2.2 Hz), 110.3, 51.6, 34.4, 25.9 (d, *J* = 2.8 Hz). **^{31}P NMR (202 MHz, $CDCl_3$)** δ -32.32. **IR (cm^{-1}, neat)**: 2925, 2849, 1726, 1516, 1468, 1433, 1363, 727. **HRMS-ESI**: m/z calculated for $C_{30}H_{31}NO_4P^+$ [M + Na^+] 500.1985, found 500.1989.

(2'-(4-methoxyphenethyl)-[1,1'-binaphthalen]-2-yl)diphenylphosphane (9)

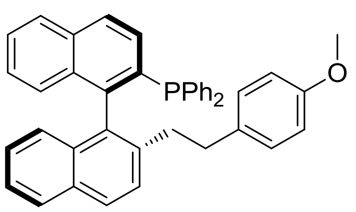

Following the modified general procedure, the reaction of **8** (87.7 mg, 0.20 mmol), **2h** (80.5 mg, 0.60 mmol), $[Rh(coe)_2Cl]_2$ (7.2 mg, 0.01mmol), K_2CO_3 (83.0 mg, 0.60 mmol) in toluene (1.0 mL) at 150 ℃ under Ar after 48h, purification by column chromatographyon silica gel (petroleum-ether : ethyl acetate = 300:1) affording 45.9 mg (39%) of **9** in 99% ee as colorless oil. (Chiralcel IA column, Hexanes/^{i}PrOH = 99.5/0.5, flow: 0.5mL/min, T = 25 ^{o}C). [α] ^{20} D 2.3 (c 2.4, $CHCl_3$). **^{1}H NMR (500 MHz, $CDCl_3$)** δ 7.98 (d, *J* = 8.5 Hz, 1H), 7.93 (d, *J* = 8.6 Hz, 2H), 7.89 (d, *J* = 8.1 Hz, 1H), 7.60 – 7.48 (m, 3H), 7.39 – 7.25 (m, 7H), 7.22 (d, *J* = 6.0 Hz, 2H), 7.16 (t, *J* = 7.3 Hz, 2H), 7.06 (t, *J* = 7.4 Hz, 2H), 7.00 (t, *J* = 7.6 Hz, 1H), 6.82 (d, *J* = 8.5 Hz, 1H), 6.70 (s, 4H), 3.77 (s, 3H), 2.76 – 2.62 (m, 2H), 2.58 – 2.48 (m, 2H). **^{13}C NMR (126 MHz, $CDCl_3$)** δ 157.7, 144.7 (d, *J* = 34.4 Hz), 138.5 (d, *J* = 2.2 Hz), 137.9, 137.8, 137.7, 135.6 (d, *J* = 11.1 Hz), 135.3 (d, *J* = 8.7 Hz), 134.1, 133.9, 133.8, 133.7, 133.5, 133.49 (d, *J* = 2.3 Hz), 133.3 (d, *J* = 7.4 Hz), 132.0, 130.5 (d, *J* = 1.6 Hz), 129.2, 128.5 (d, *J* = 6.1 Hz), 128.4, 128.3, 128.2 (d, *J* = 6.8 Hz), 128.0 (d, *J* = 4.6 Hz), 127.8, 127.1 (d, *J* = 2.7 Hz), 127.0 (d, *J* = 10.6 Hz), 126.6 (d, *J* = 25.7 Hz), 125.8, 125.0, 113.6, 55.3, 35.9 (d,*J* = 1.26 Hz), 35.3. **IR (cm^{-1}, neat)**: 3053, 2956, 2834, 1511, 1434,1435, 1211, 1169, 1028,742, 697. **HRMS-ESI**: m/z calculated for$C_{41}H_{34}OP^+$ [M + H^+] 573.2342, found 573.2346.

(2'-(2-(2,3-dihydrobenzofuran-4-yl)ethyl)-[1,1'-binaphthalen]-2-yl)diphenylphosphine (10)

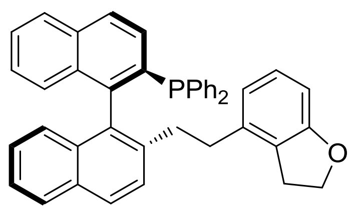

Following the modified general procedure, the reaction of **8** (87.7 mg, 0.20 mmol), **2i** (122.4 mg, 0.60 mmol), $[Rh(coe)_2Cl]_2$ (7.2 mg, 0.01mmol), K_2CO_3 (83.0 mg, 0.60 mmol) in toluene (1.0 mL) at 150 ℃ under Ar after 60h, purification by column chromatographyon silica gel (petroleum-ether : ethyl acetate = 100:1) affording 72.4 mg (62%) of **10** in > 99% ee as colorless oil. (Chiralcel OD-H column, Hexanes/^{i}PrOH = 99/1, flow: 0.5mL/min, T = 25 °C). $[\alpha]^{20}_D$ 21.6 (c 0.75, $CHCl_3$). **^{1}H NMR (500 MHz, $CDCl_3$)** δ 7.94 (d, *J* = 8.5 Hz, 1H), 7.85 (dd, *J* = 14.9, 8.3 Hz, 3H), 7.56 – 7.42 (m, 3H), 7.30 – 7.07 (m, 11H), 7.03 – 6.86 (m, 4H), 6.76 (d, *J* = 8.4 Hz, 1H), 6.52 (d, *J* = 7.9 Hz, 1H), 6.32 (d, *J* = 7.6 Hz, 1H), 4.31 – 4.16 (m, 2H), 2.69 – 2.62 (m, 1H), 2.55 – 2.33 (m, 3H), 2.30 – 2.18 (m, 2H). **^{13}C NMR (126 MHz, $CDCl_3$)** δ 159.7, 144.4 (d, *J* = 34.1 Hz), 138.6, 138.3 (d, *J* = 2.3 Hz), 137.5 (d, *J* = 13.1 Hz), 137.3 (d, *J* = 13.1 Hz), 135.8 (d, *J* = 11.4 Hz), 135.1 (d, *J* = 8.6 Hz), 133.8, 133.7, 133.64, 133.61, 133.5, 133.4 (d, *J* = 2.3 Hz), 133.3 (d, *J* = 7.3 Hz), 131.9, 130.4 (d, *J* = 2.0 Hz), 128.4, 128.35, 128.32, 128.24, 128.21, 128.1, 127.9 (d, *J* = 2.4 Hz), 127.8 (d, *J* = 5.8 Hz), 127.1, 127.0, 126.9, 126.6 (d, *J* = 13.6 Hz), 125.8, 125.4, 125.0, 120.1, 106.8, 70.8, 34.4, 34.2, 27.7. ^{31}P NMR (162 MHz, Chloroform-*d*) δ -14.8. **IR (cm^{-1}, neat)**: 2886, 2215, 2020, 1830, 1647, 1455, 1376, 682. **HRMS-ESI**: m/z calculated for $C_{42}H_{34}OP^+$ [M + H$^+$] 585.2342, found 585.2350.

(2'-(2-(anthracen-9-yl)ethyl)-[1,1'-binaphthalen]-2-yl)diphenylphosphine (11)

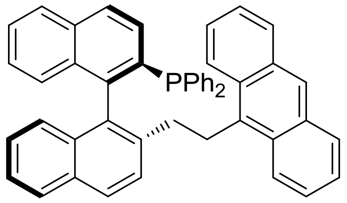

Following the modified general procedure, the reaction of **8** (87.7 mg, 0.20 mmol), **2j** (87.6 mg, 0.60 mmol), $[Rh(coe)_2Cl]_2$ (7.2 mg, 0.01mmol), K_2CO_3 (83.0 mg, 0.60 mmol) in toluene (1.0 mL) at 150 ℃ under Ar after 60h, purification by column chromatographyon silica gel (petroleum-ether : ethyl acetate = 500:1) affording 33.4 mg (26%) of **11** in > 99% ee as colorless oil. (Chiralcel IB column, Hexanes/^{i}PrOH = 99.8/0.2, flow: 0.5mL/min, T = 25 °C). **^{1}H NMR (500**

MHz, $CDCl_3$) δ 8.21 (s, 1H), 8.14 – 7.97 (m, 3H), 7.87 (dd, *J* = 16.7, 8.2 Hz, 4H), 7.57 – 7.43 (m, 3H), 7.39 – 7.26 (m, 6H), 7.21 – 6.93 (m, 11H), 6.90 – 6.75 (m, 3H), 3.74 (td, *J* = 13.3, 4.8 Hz, 1H), 3.33 (td, *J* = 13.4, 5.3 Hz, 1H), 2.82 – 2.56 (m, 2H). **^{13}C NMR (126 MHz, $CDCl_3$)** δ 144.3 (d, *J* = 33.7 Hz), 138.8 (d, *J* = 2.5 Hz), 137.2 (d, *J* = 2.7 Hz), 137.0 (d, *J* = 2.7 Hz), 136.1 (d, *J* = 11.8 Hz), 135.2 (d, *J* = 8.4 Hz), 133.99, 133.93 (d, *J* = 2.7 Hz), 133.8, 133.7 (d, *J* = 6.9 Hz), 133.3 (d, *J* = 19.3 Hz), 132.1, 131.4, 130.5 (d, *J* = 1.8 Hz), 129.3, 128.9, 128.7, 128.3, 128.1 (d, *J* = 2.2 Hz), 128.1, 128.04 (d, *J* = 1.9 Hz), 128.00, 127.9, 127.7 (d, *J* = 2.2 Hz), 127.17, 127.15 , 126.8 (d, *J* = 7.0 Hz), 125.7 (d, *J* = 21.4 Hz), 125.3, 125.0, 124.7, 123.9, 36.0 (d, *J* = 2.3 Hz), 29.8. **^{31}P NMR (162 MHz, Chloroform-*d*)** δ -14.5. **IR (cm^{-1}, neat)**: 3080, 2881, 1923, 1524, 1444, 1349, 1116, 883. **HRMS-ESI**: m/z calculated for $C_{48}H_{36}P^+$ [M + H^+] 643.2549, found 643.2547.

(S)-1-benzyl-3-hydroxy-3-phenylindolin-2-one (14a)

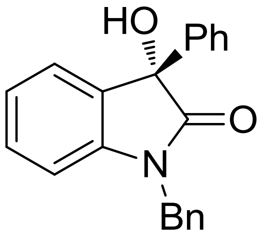

A solution of $[RhCl(C_2H_4)_2]_2$ (1.9 mg, 9.8 μmol Rh) and **11** (20 μmol) in THF (1.0 mL) was stirred for 10 min at room temperature. KOH (0.10 mL, 30 μmol; 0.3 M aqueous), isatin **12a** (0.20 mmol), and $PhB(OH)_2$ (0.40 mmol) were added successively with additional THF (1.0 mL). The resulting mixture was stirred for 48 h at 40 ℃, and this was directly passed through a pad of silica gel with Et_2O. After removing the solvent under vacuum, the residue was purified by column chromatographyon silica gel (petroleum-ether : ethyl acetate = 3:1) affording 49 mg (78%) of **14** in 84% ee as a white solid. (Chiralcel AS-H column, Hexanes/^{i}PrOH = 50/50, flow: 0.5mL/min, T = 25 ^{o}C). $[\alpha]^{20}$ D –12.0 (c 0.25, $CHCl_3$). **^{1}H NMR (500 MHz, $CDCl_3$)** δ ^{1}H NMR (400 MHz, Chloroform-*d*) δ 7.42 (dd, *J* = 8.1, 1.4 Hz, 2H), 7.38 – 7.27 (m, 9H), 7.23 (td, *J* = 7.8, 1.2 Hz, 1H), 7.05 (t, *J* = 7.9 Hz, 1H), 6.79 (d, *J* = 7.9 Hz, 1H), 5.05 (d, *J* = 15.7 Hz, 1H), 4.84 (d, *J* = 15.7 Hz, 1H), 3.29 (s, 1H). **^{13}C NMR (126 MHz, $CDCl_3$)** δ 177.6, 142.7, 140.1, 135.4, 131.5, 129.8, 128.9, 128.7, 128.4, 127.8, 127.3, 125.2, 125.0 , 123.6, 109.8, 96.0, 44.1.

(S)-1-benzyl-5-chloro-3-hydroxy-3-phenylindolin-2-one (14b)

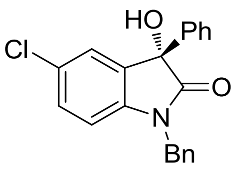

A solution of $[RhCl(C_2H_4)_2]_2$ (1.9 mg, 9.8 μmol Rh) and **9** (20 μmol) in THF (1.0 mL) was stirred for 10 min at room temperature. KOH (0.10 mL, 30 μmol; 0.3 M aqueous), isatin **12b** (0.20 mmol), and $PhB(OH)_2$ (0.40 mmol) were added successively with additional THF (1.0 mL). The resulting mixture was stirred for 48 h at 40 ℃, and this was directly passed through a pad of silica gel with Et_2O. After removing the solvent under vacuum, the residue was purified by column chromatographyon silica gel (petroleum-ether : ethyl acetate = 3:1) affording 61.4 mg (88%) of **14b** in 90% ee as a white solid. (Chiralcel AS-H column, Hexanes/^{i}PrOH = 50/50, flow: 0.5mL/min, T = 25 ^{o}C). $[\alpha]^{20}$ D –4.5 (c 0.75, $CHCl_3$). **^{1}H NMR (500 MHz, $CDCl_3$)** δ ^{1}H NMR (400 MHz, Chloroform-*d*) δ 7.38 – 7.23 (m, 11H), 7.17 (dd, *J* = 8.3, 2.2 Hz, 1H), 6.68 (d, *J* = 8.4 Hz, 1H), 5.01 (d, *J* = 15.7 Hz, 1H), 4.80 (d, *J* = 15.7 Hz, 1H), 3.94 (s, 1H).**^{13}C NMR (126 MHz, $CDCl_3$)** δ 177.4, 141.0, 139.6, 134.9, 133.4, 129.7, 129.1, 128.9, 128.8, 128.6, 127.9, 127.2, 125.5, 125.2, 110.8, 78.0, 44.2.

KIE Experiments

([1,1'-biphenyl]-2-yl-2',3',4',5',6'-d5)diphenylphosphane (D-1a)

Following the general procedure, **D-1a**[2] was prepared from (2-bromophenyl)diphenylphosphane and (phenyl-d5) boronic acid.

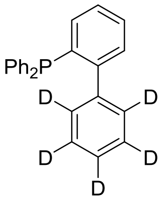

D-1a

General operation procedure for synthesis of D-3aa: The reaction of **D-1a** (0.20 mmol), **2a** (0.20 mmol), $NaHCO_3$ (0.60 mmol) and $[Rh(cod)Cl]_2$ (0.025 mmol) in toluene (0.5 mL) at 140 ℃ under Ar in 3 h, purification by column chromatography on silica gel affording the target product **D-3aa**. And its NMR spectra and deuterated ratio were obtained over time using 400MHz ^{1}H NMR spectrometer.

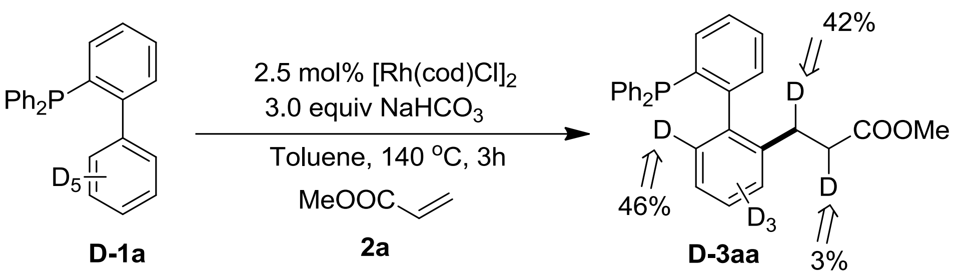

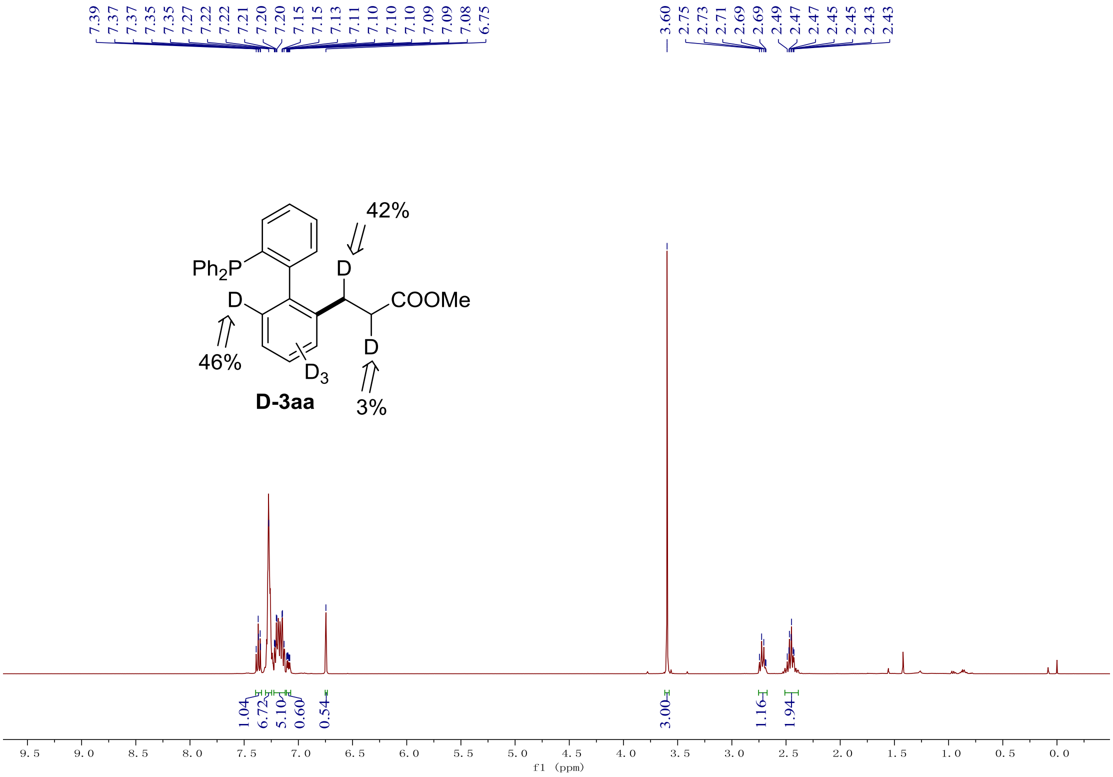

Supplementary Figure 1. ^{1}H NMR of compound D-**3aa.**

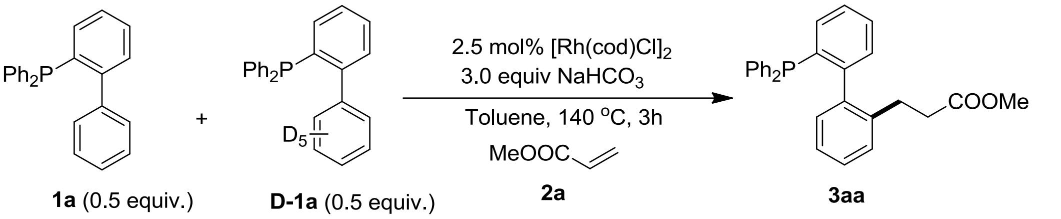

The reaction of **D-1a** (0.20 mmol) or **1a** (0.20 mmol), **2a** (0.20 mmol), $NaHCO_3$ (0.60 mmol) and $[Rh(cod)Cl]_2$ (0.025mmol) in toluene (0.5 mL) at 140 ℃ under Ar in 10 min. After cooled to rom temeperature it was passed through a short pad of silica and the filtrate was concentrated in vacuo. The yield was determined by 1H NMR analysis of the crude product using dibromomethane as the internal standard. The result was analyzed by ^{1}HNMR spectrum (relaxation delays = 10s). The KIE value of

the C-H activation process was 2.5 (deuteron products: 29% and non-deuteron products: 71%), revealing that the C-H cleavage is slow and involved as a rate-determining step.

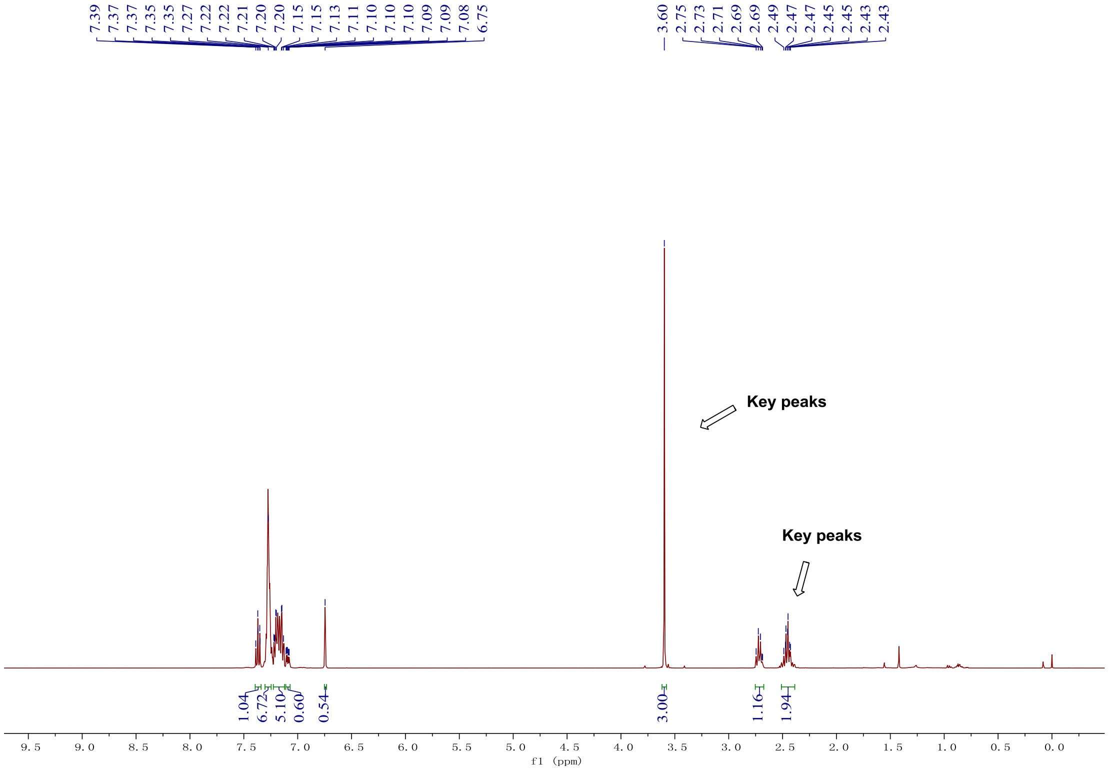

Supplementary Figure 2. ^{1}H NMR of compound **3aa** and **D-3aa.**

General operation procedure for synthesis of D-5aa: The reaction of **D-1a** (0.20 mmol), **4a** (0.40 mmol) and $[Rh(cod)Cl]_2$(0.025 mmol) in toluene (0.5 mL) at 120 ℃ under Arin 12 h. Purification by column chromatography on silica gel affording the target product **D-5aa**. And its NMR spectra and deuterated ratio were obtained over time using 400MHz ^{1}H NMR spectrometer.

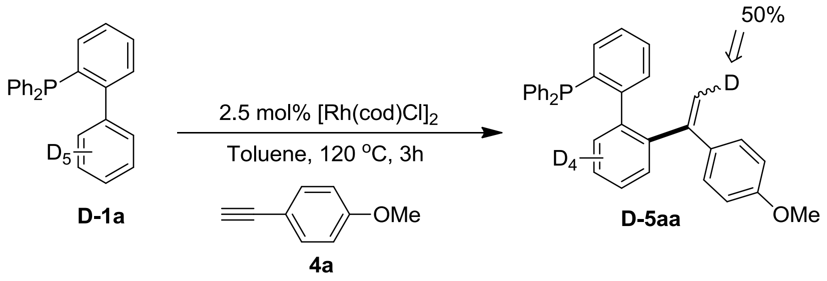

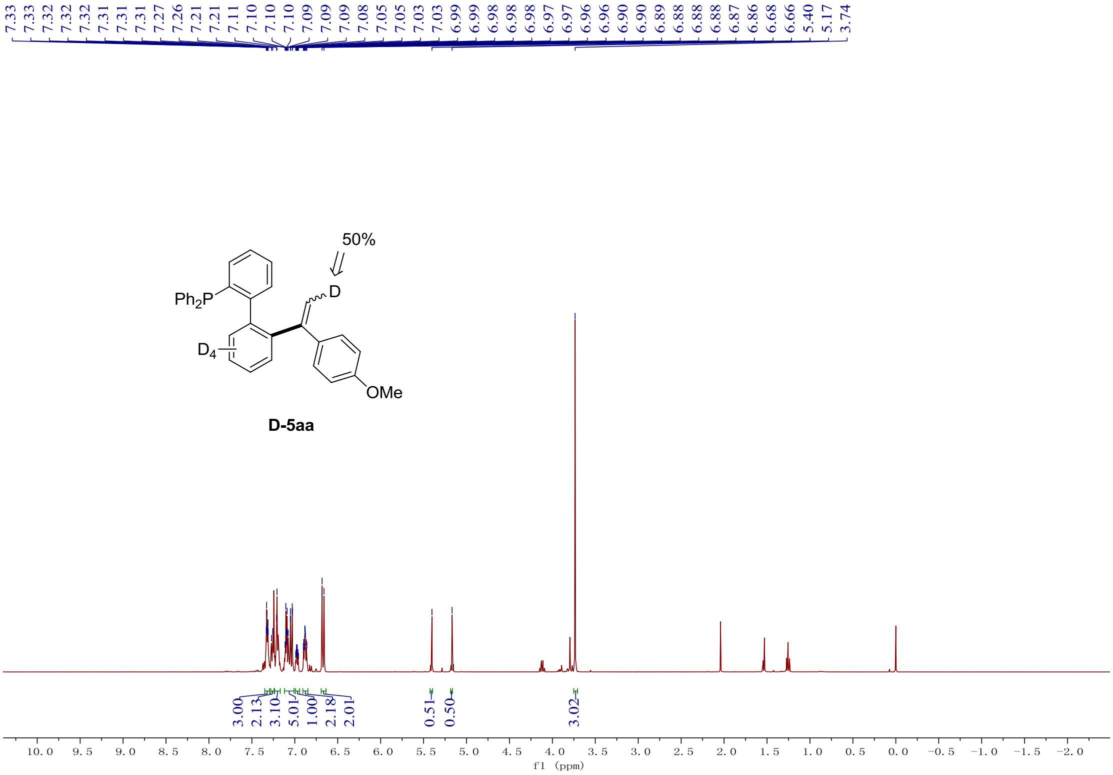

Supplementary Figure 3. ^{1}H NMR of compound D-**5aa.**

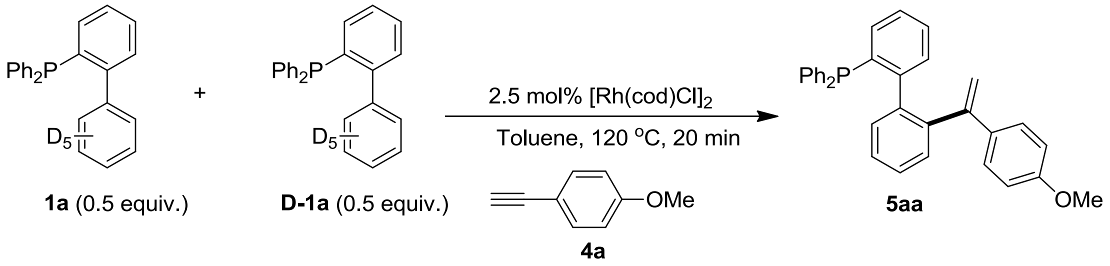

The reaction of **D-1a** (0.10 mmol), **1a** (0.1 mmol), **4a** (0.20 mmol) and $[Rh(cod)Cl]_2$ (0.025 mmol) in toluene (0.5 mL) at 120 ℃ under Ar after 20 min. Purification by column chromatography on silica gel affording the target product **5aa**. The result was analyzed by ^{1}HNMR spectrum (relaxation delays = 10s). The KIE value of the C-H activation process was 3.5(deuteron products: 22% and non-deuteron products: 78%), revealing that the C-H cleavage is slow and involved as a rate-determining step.

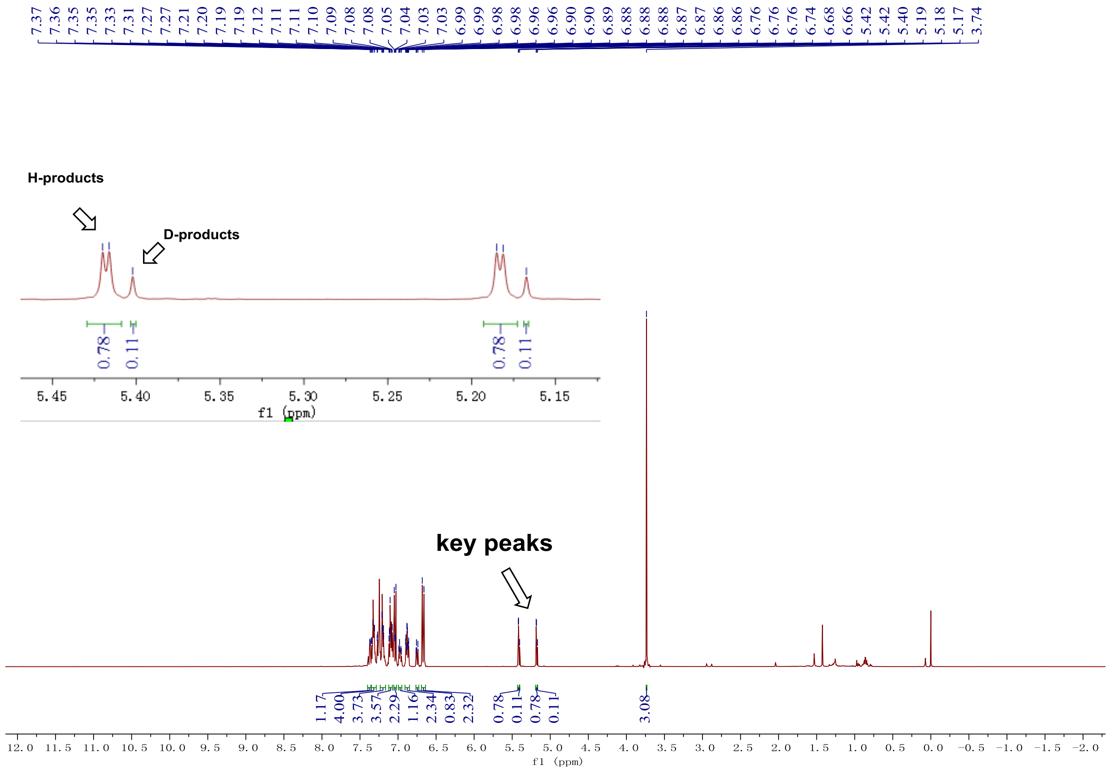

Supplementary Figure 4. ^{1}H NMR of compound **5aa** and **D-5aa.**

Complexation of [Rh(cod)Cl]$_2$ with different BiarylPhosphines.

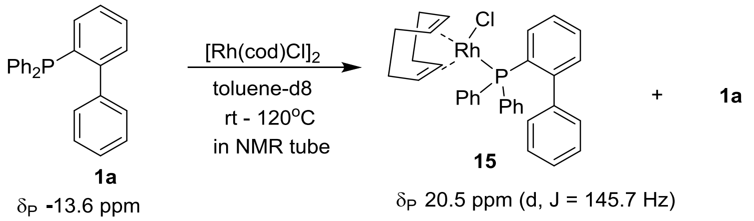

The reaction of **1a** (0.10 mmol) and [Rh(cod)Cl]$_2$ (0.10mmol) in toluene-d8 (0.5 mL) at RT-100 ℃ under Ar in NMR tube. ^{31}P NMR of the reaction was tested with in a period of ten minutes at RT to 120 ℃. And its ^{31}P NMR spectra were obtained using 400MHz ^{1}H NMR spectrometer shown below indicated formation of complexes **14** and compound **1a** as a mixture. The results show that C-H activation of the reaction do not occur without alkenes or alkynes.

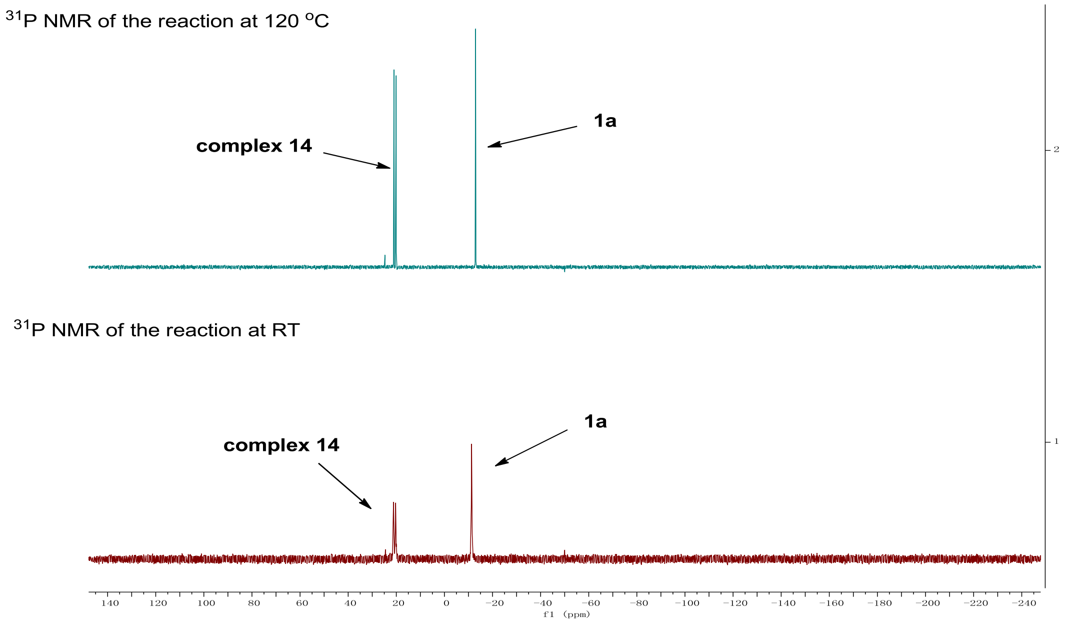

Supplementary Figure 5. ^{31}P NMR of complex **14.**

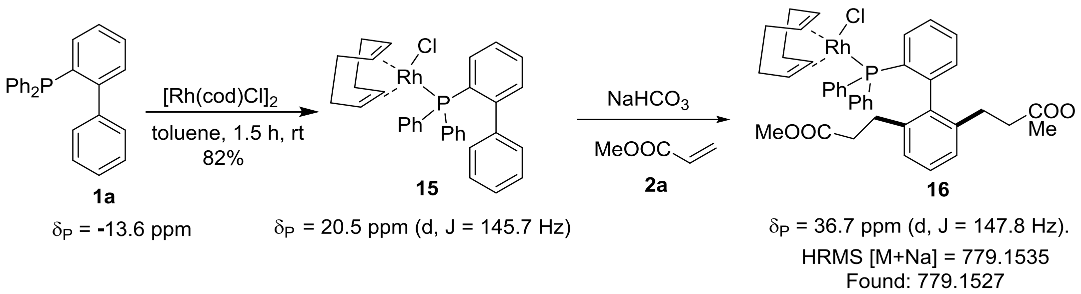

The reaction of **15** (0.10 mmol), **2a** (0.30 mmol) and $NaHCO_3$ (0.3 mmol) in toluene (0.5 mL) under Ar in Schlenk tube. The mixture was stirred at 140 ℃ for 12 h. Then the reaction was cooled to room temperature. When the reaction was completed under the standard condition, we took a few samples from the reaction mixture and directly analyzed by ESI-MS in positive ion mode. The target peak with m/z signals characterized for cationic intermediates were trapped. And purification by column chromatography on silica gel affording the target complexes. And its ^{31}P NMR spectra were obtained using 400MHz ^{1}H NMR spectrometershown belowindicated formation of complex **16**.

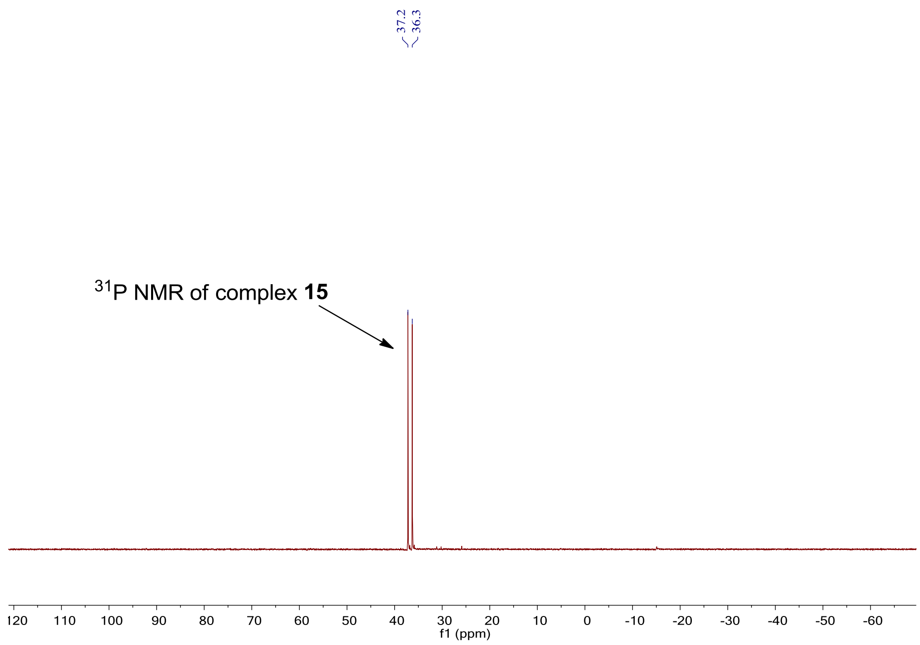

Supplementary Figure 6. ^{31}P NMR of complex **15.**

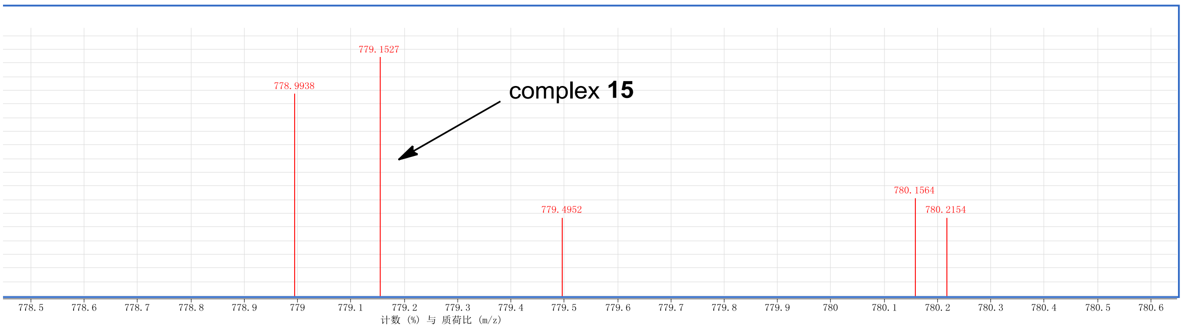

Supplementary Figure 7. high resolution mass spectrometry of compound **15.**

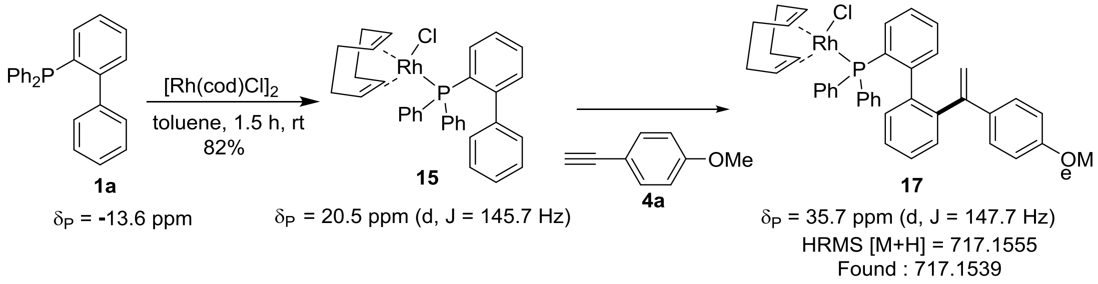

The reaction of **15** (0.10 mmol) and **4a** (0.2 mmol) in toluene (0.5 mL) under Ar in Schlenk tube. The mixture was stirred at 120 ℃ for 12 h. Then the reaction was cooled down to room temperature. When the reaction was completed under the standard condition, we took a few samples from the reaction mixture and directly analyzed by ESI-MS in positive ion mode. The target peak with m/z signals characterized for cationic intermediates were trapped. And purification by column

chromatography on silica gel affording the target complexes. And its ^{31}P NMR spectra were obtained using 400MHz ^{1}H NMR spectrometer shown below indicated formation of complex **17**.

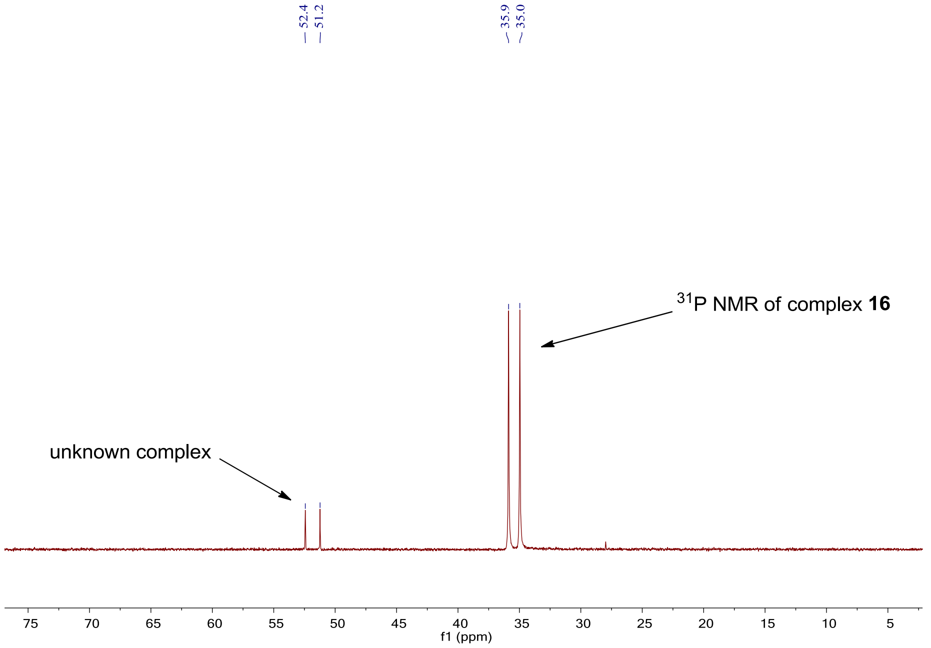

Supplementary Figure 8. ^{31}P NMR of complex **16.**

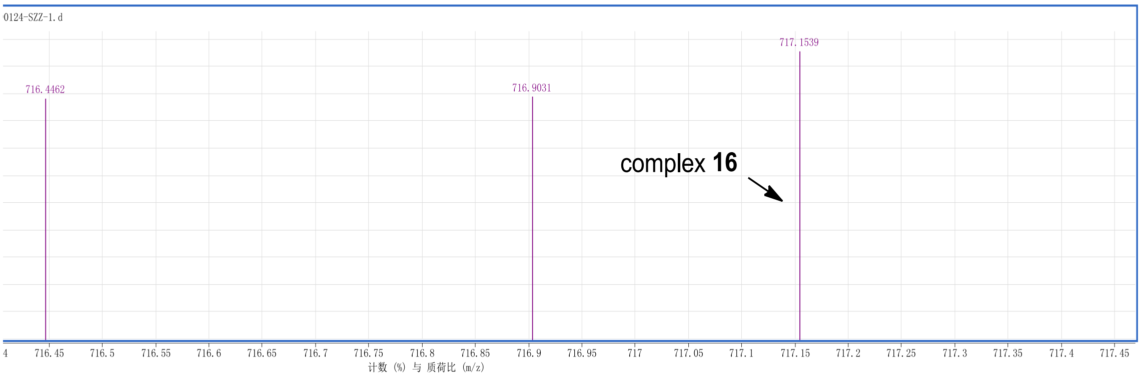

Supplementary Figure 9. high resolution mass spectrometry of complex **16.**

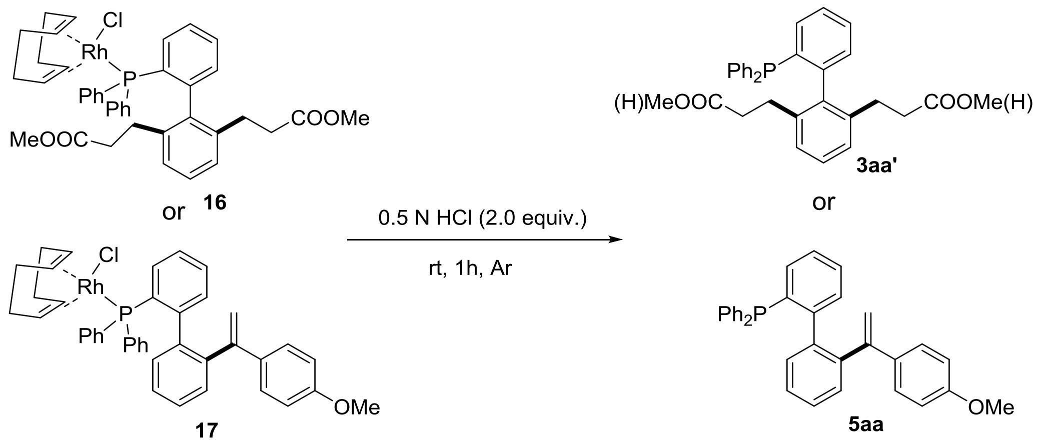

HCl (0.5N HCl, 2.0 equiv.) was added to a solution of **16** or **17** (0.1 mmol) in THF (0.5 mL) at 0 ℃ and the resulting mixture was stirred for 1 h at room temperature under Ar. And the reaction mixture was diluted with DCM and washed with water. The organic layer was dried over $MgSO_4$, filtered, and concentrated under vacuum to afford **3aa'** or **5aa**.

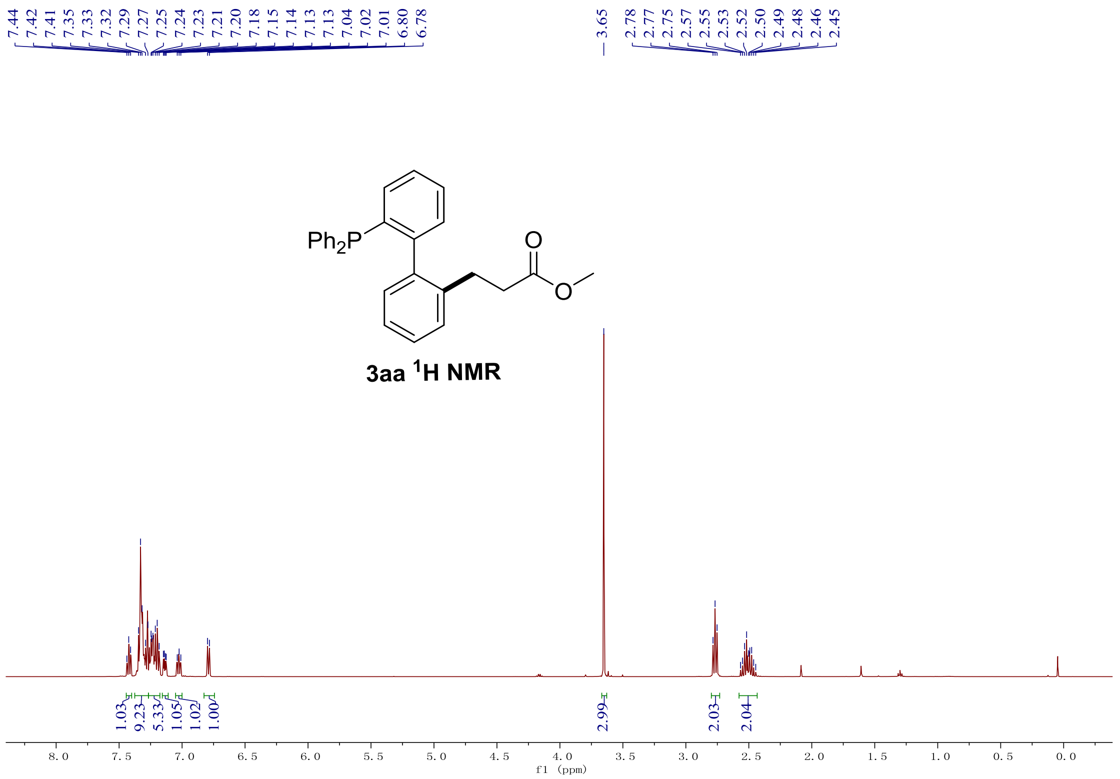

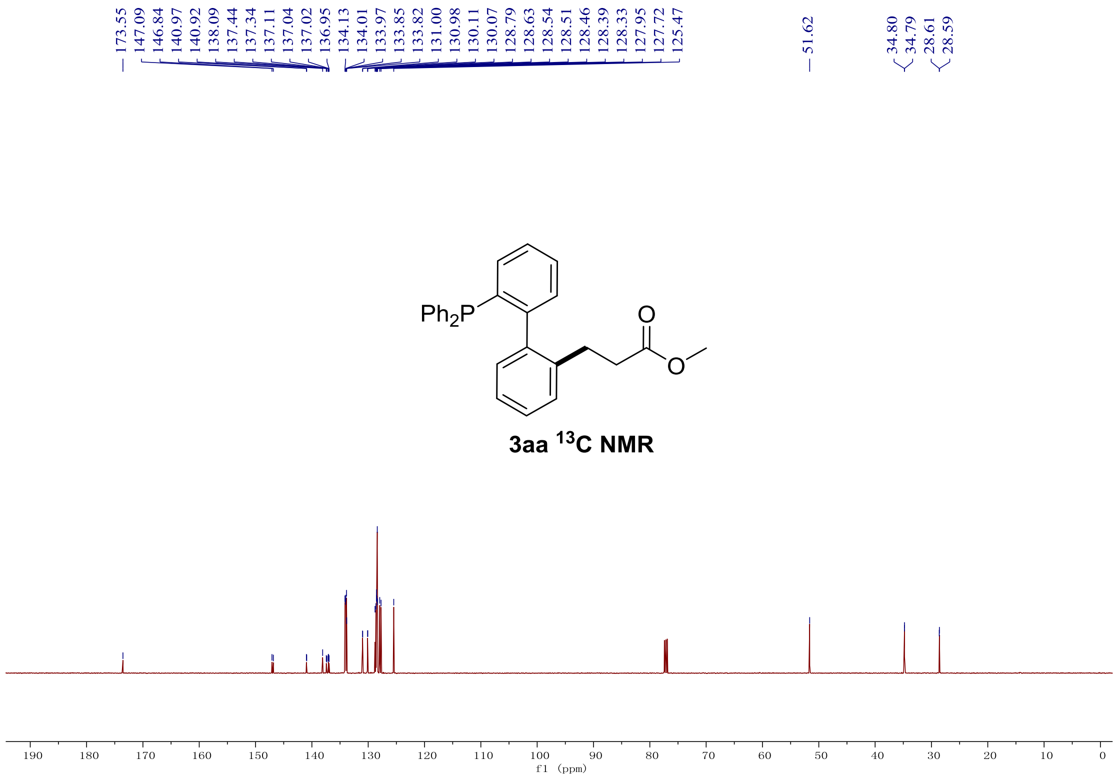

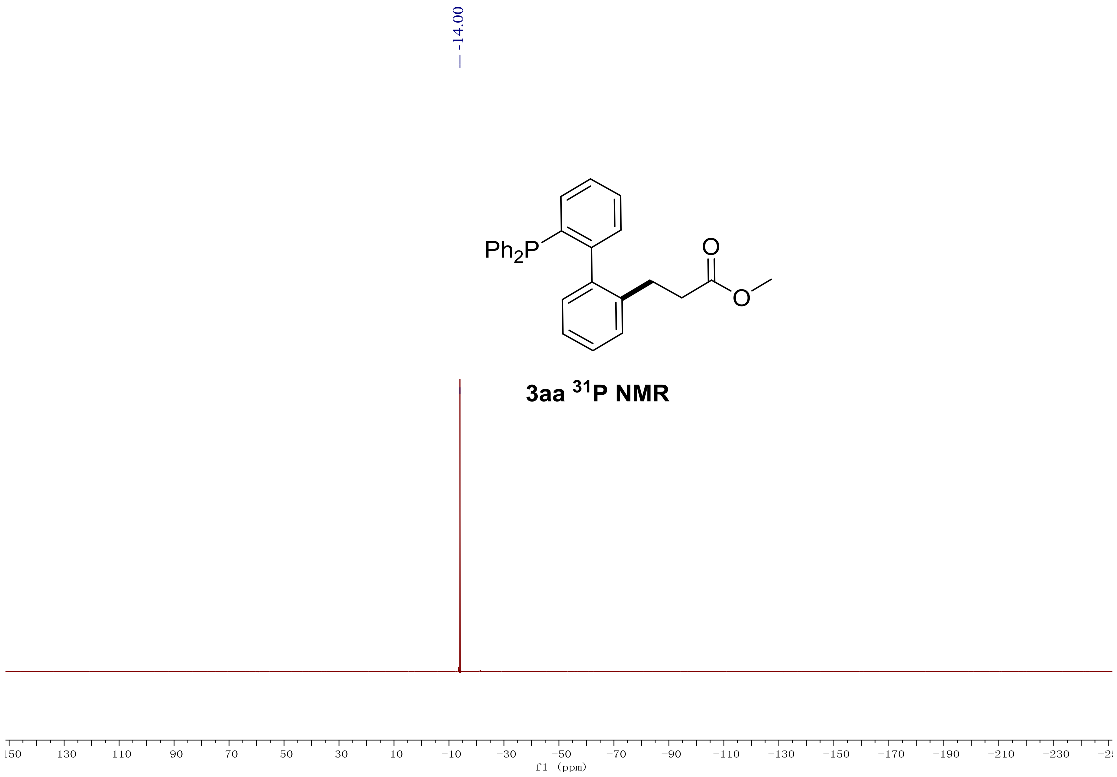

Supplementary Figure 10. ^{1}H, ^{13}C and ^{31}P NMR of compound **3aa.**

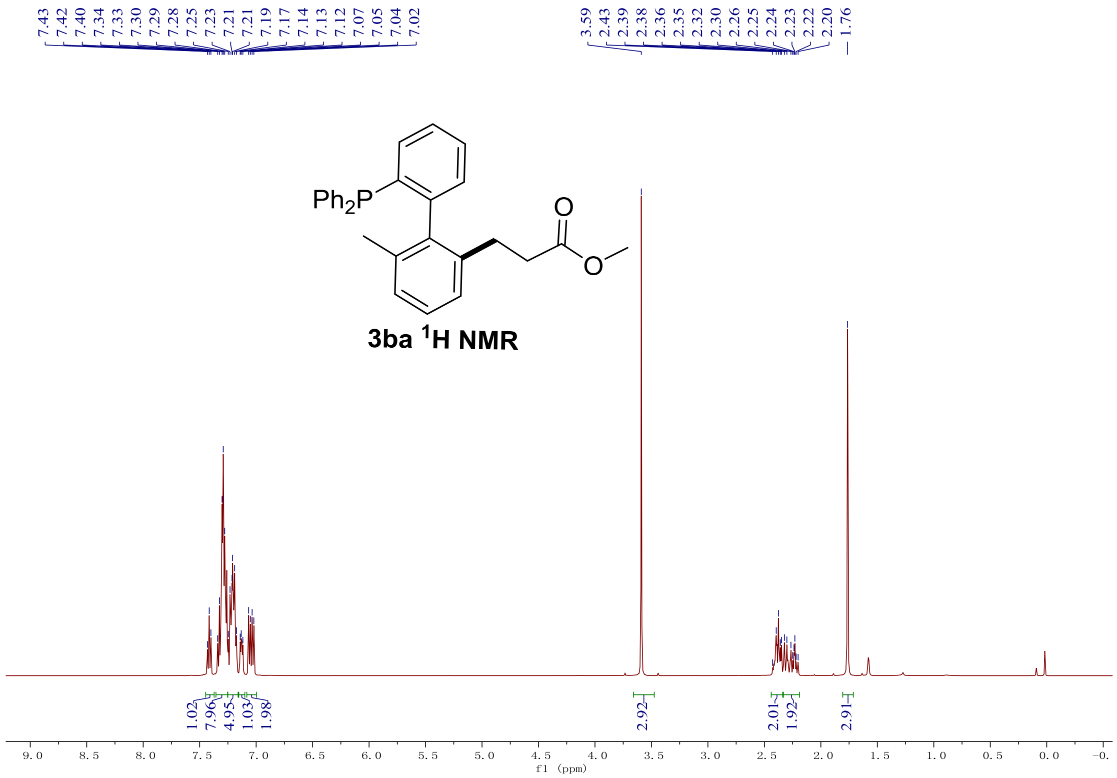
7.43 7.42 7.40 7.34 7.33 7.30 7.29 7.28 7.25 7.23 7.21 7.21 7.19 7.17 7.14 7.13 7.12 7.07 7.05 7.04 7.02
3.59 2.43 2.39 2.38 2.36 2.35 2.32 2.30 2.26 2.25 2.24 2.23 2.22 2.20 1.76
Ph2P
O
O
3ba 1H NMR
1.02 7.96 4.95 1.03 1.98
2.92
2.01 1.92
2.91
f1 (ppm)

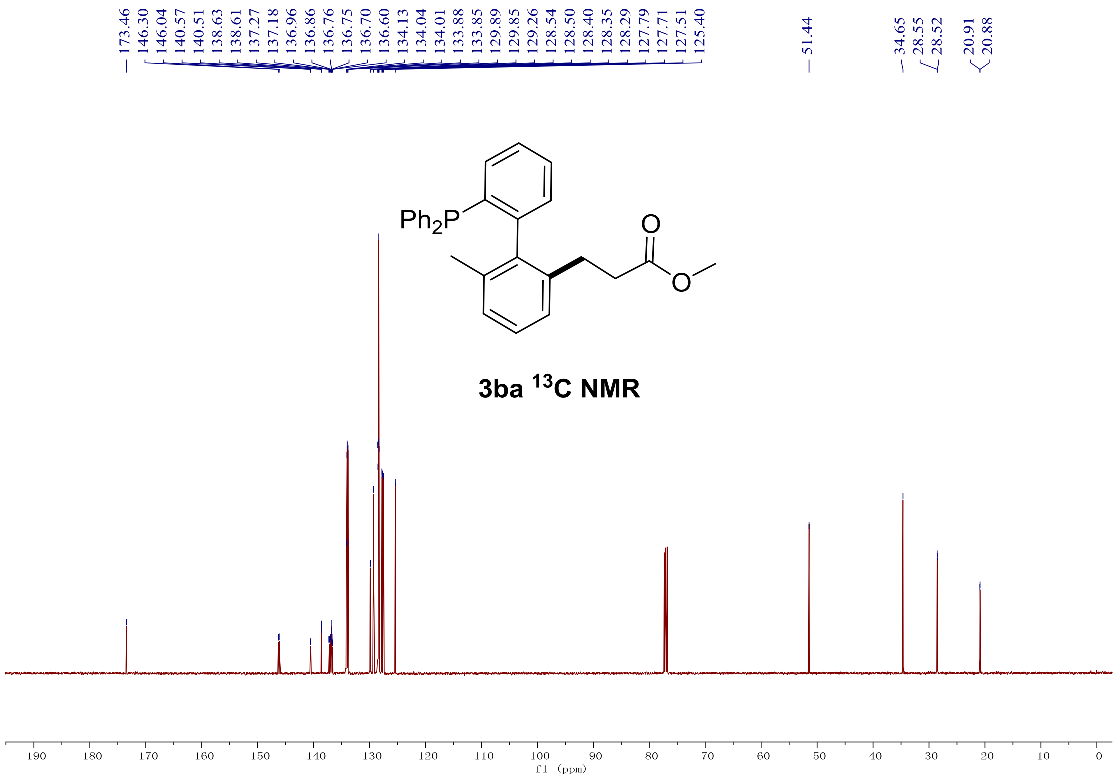
173.46 146.30 146.04 140.57 140.51 138.63 138.61 137.27 137.18 136.96 136.86 136.76 136.75 136.70 136.60 134.13 134.04 134.01 133.88 133.85 129.89 129.85 129.26 128.54 128.50 128.40 128.35 128.29 127.79 127.71 127.51 125.40
51.44
34.65 28.55 28.52 20.91 20.88
Ph2P
O
O
3ba 13C NMR
f1 (ppm)

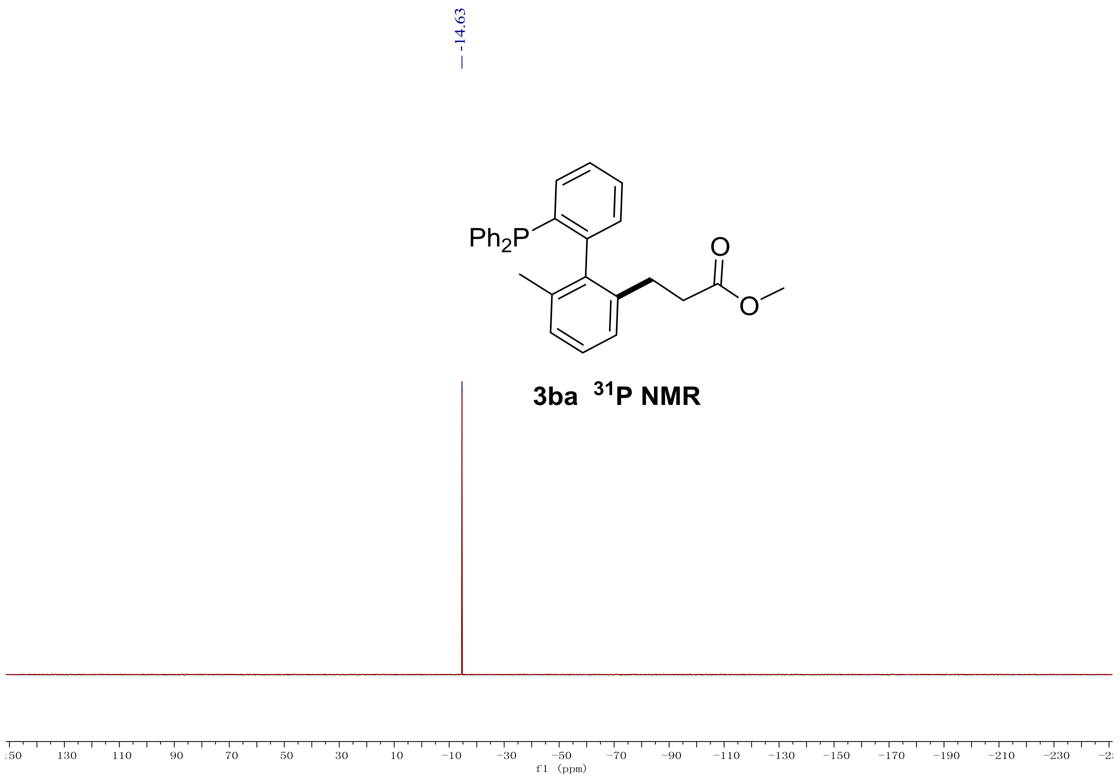

Supplementary Figure 11. ^{1}H, ^{13}C and ^{31}P NMR of compound **3ba.**

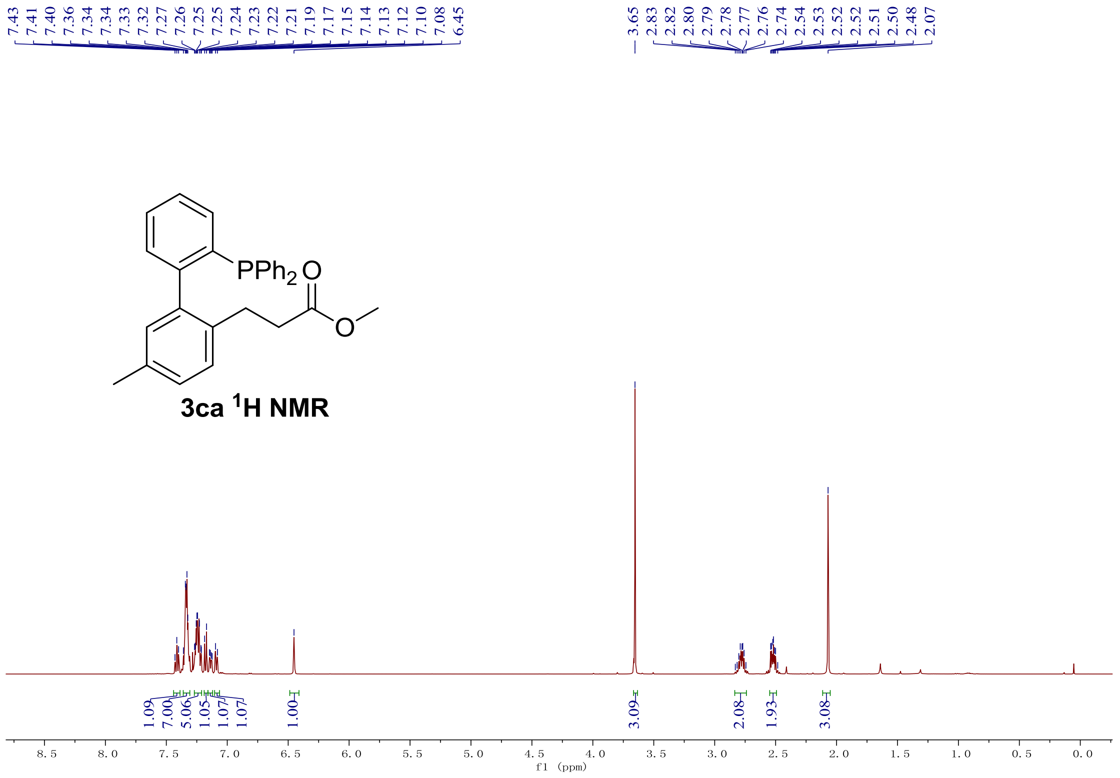

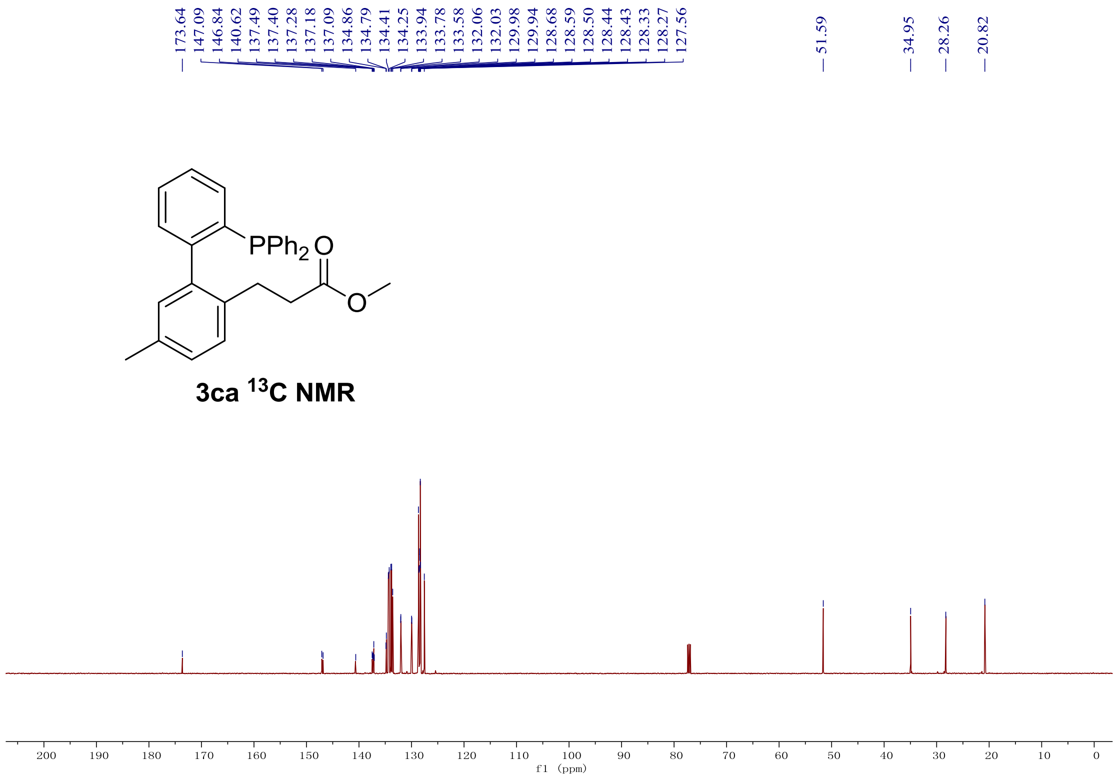

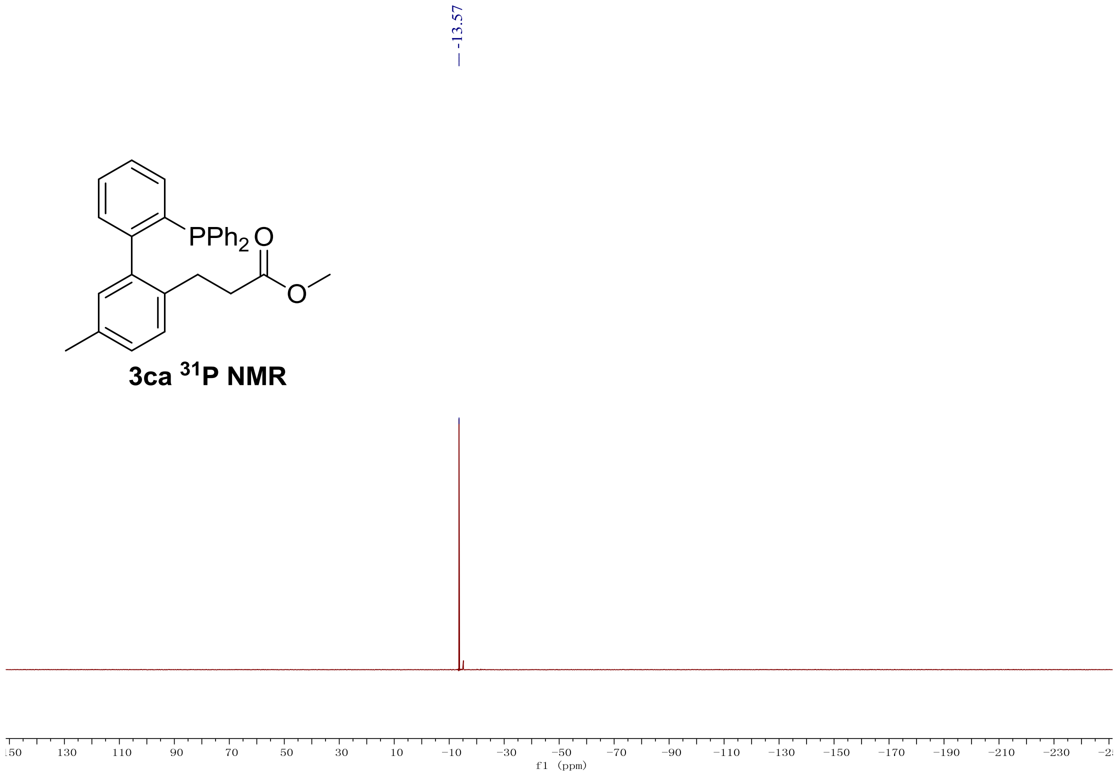

Supplementary Figure 12. ^{1}H, ^{13}C and ^{31}P NMR of compound **3ca.**

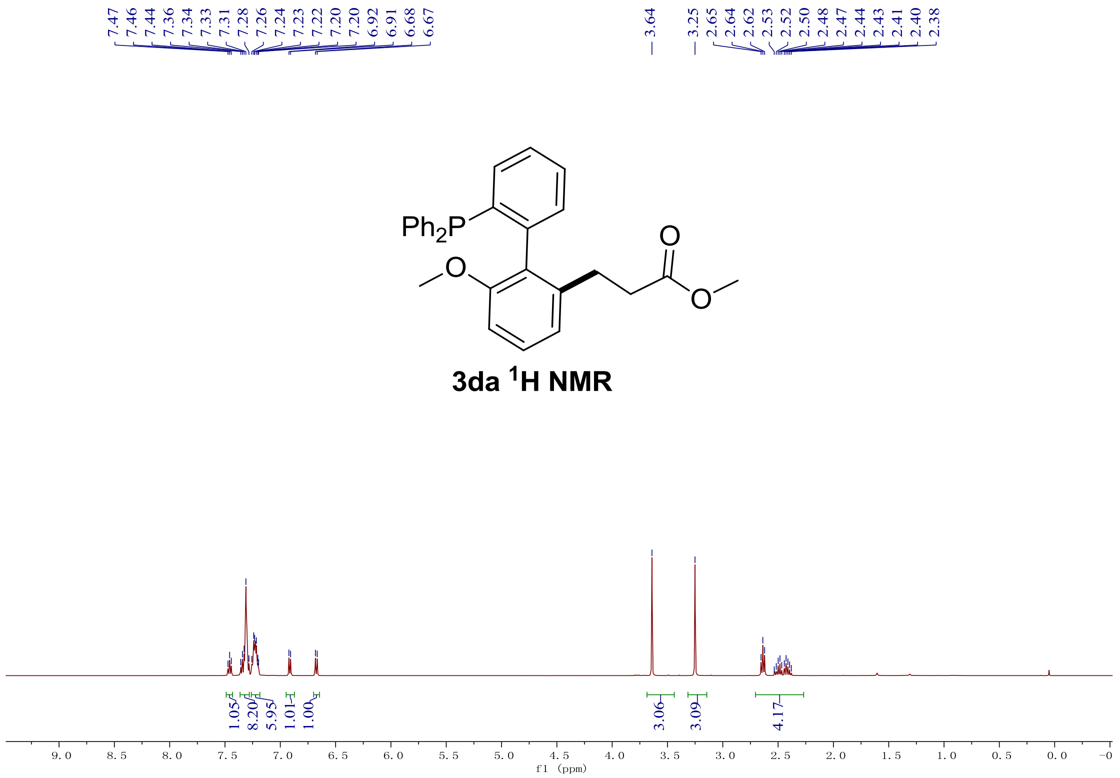
7.47
7.46
7.44
7.36
7.34
7.33
7.31
7.28
7.26
7.24
7.23
7.22
7.20
7.20
6.92
6.91
6.68
6.67
3.64
3.25
2.65
2.64
2.62
2.53
2.52
2.50
2.48
2.47
2.44
2.43
2.41
2.40
2.38
Ph2P
O
O
O
3da 1H NMR
1.05
8.20
5.95
1.01
1.00
3.06
3.09
4.17
f1 (ppm)

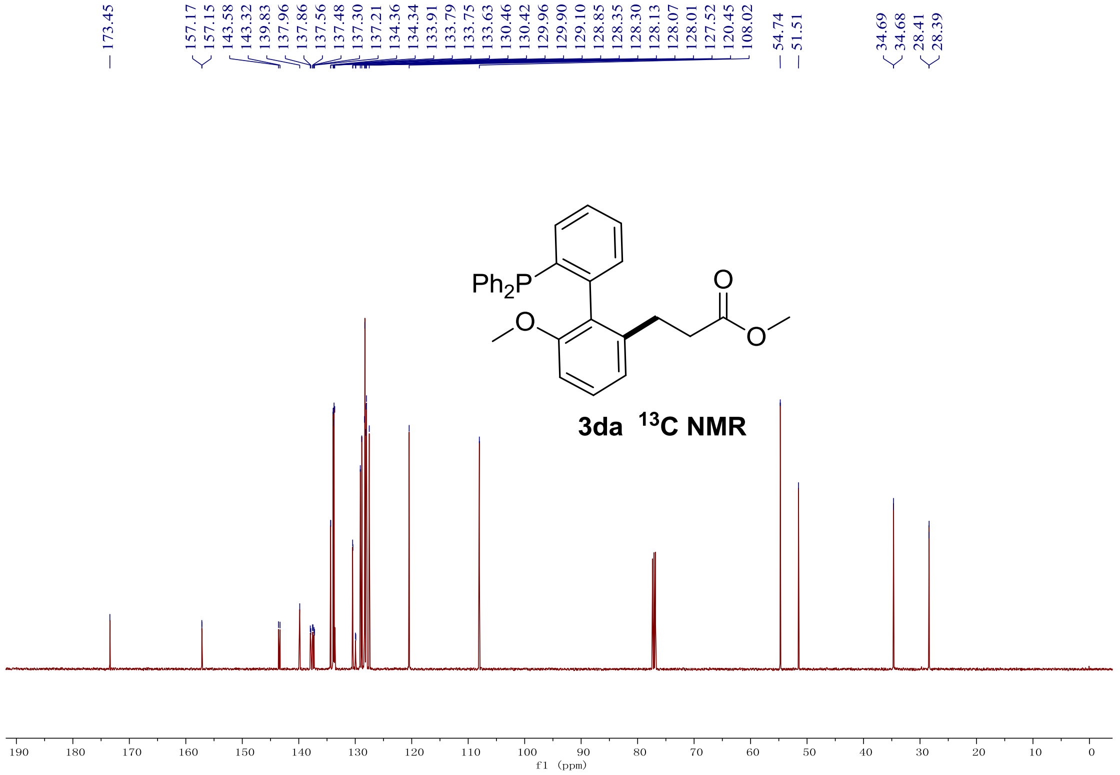
173.45
157.17
157.15
143.58
143.32
139.83
137.96
137.86
137.56
137.48
137.30
137.21
134.36
134.34
133.91
133.79
133.75
133.63
130.46
130.42
129.96
129.90
129.10
128.85
128.35
128.30
128.13
128.07
128.01
127.52
120.45
108.02
54.74
51.51
34.69
34.68
28.41
28.39
Ph2P
O
O
O
3da 13C NMR
f1 (ppm)

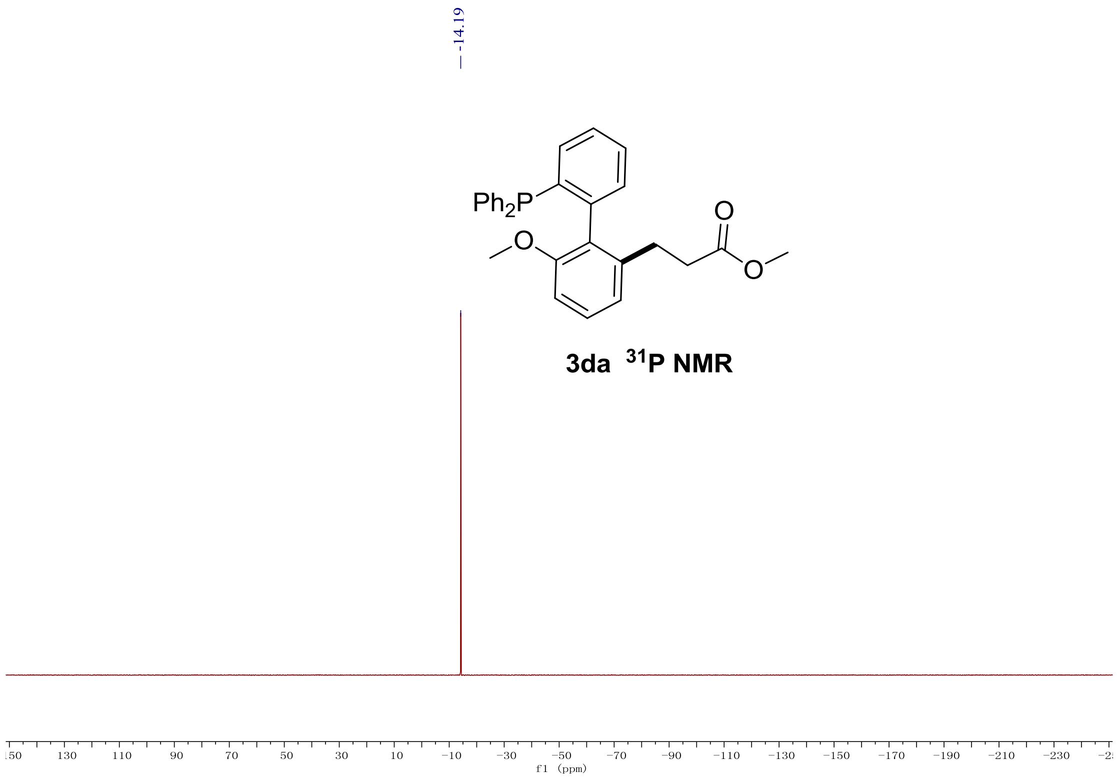

Supplementary Figure 13. ^{1}H, ^{13}C and ^{31}P NMR of compound **3da.**

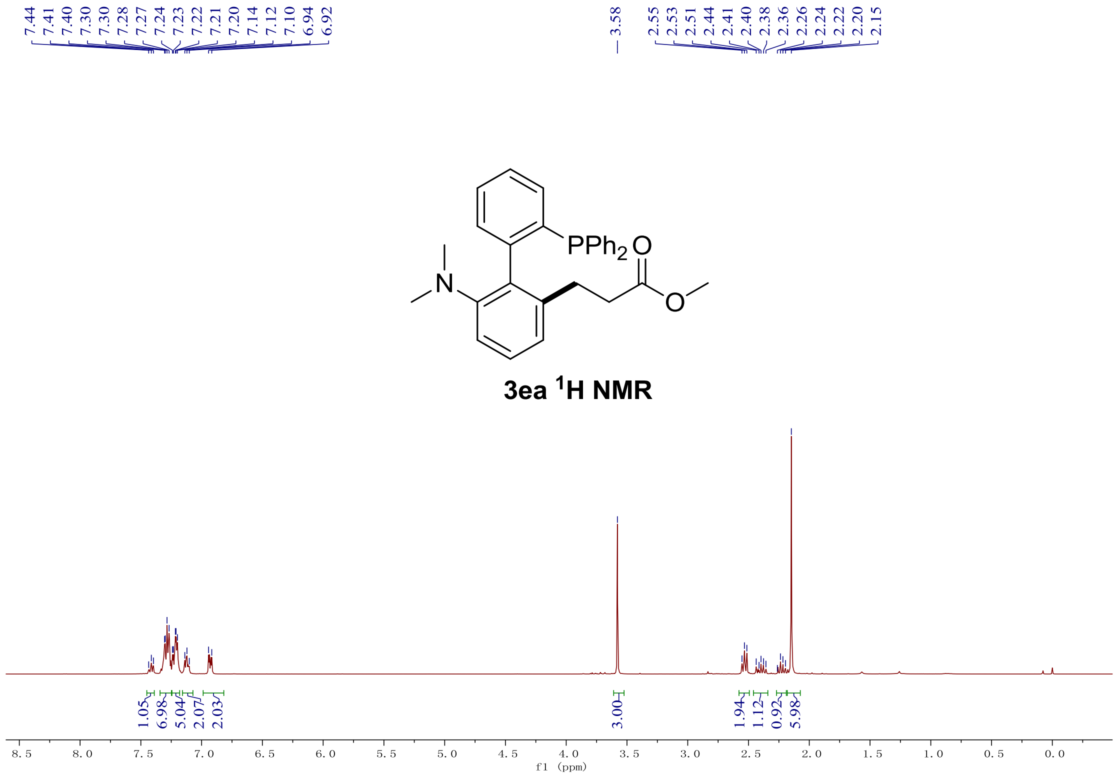

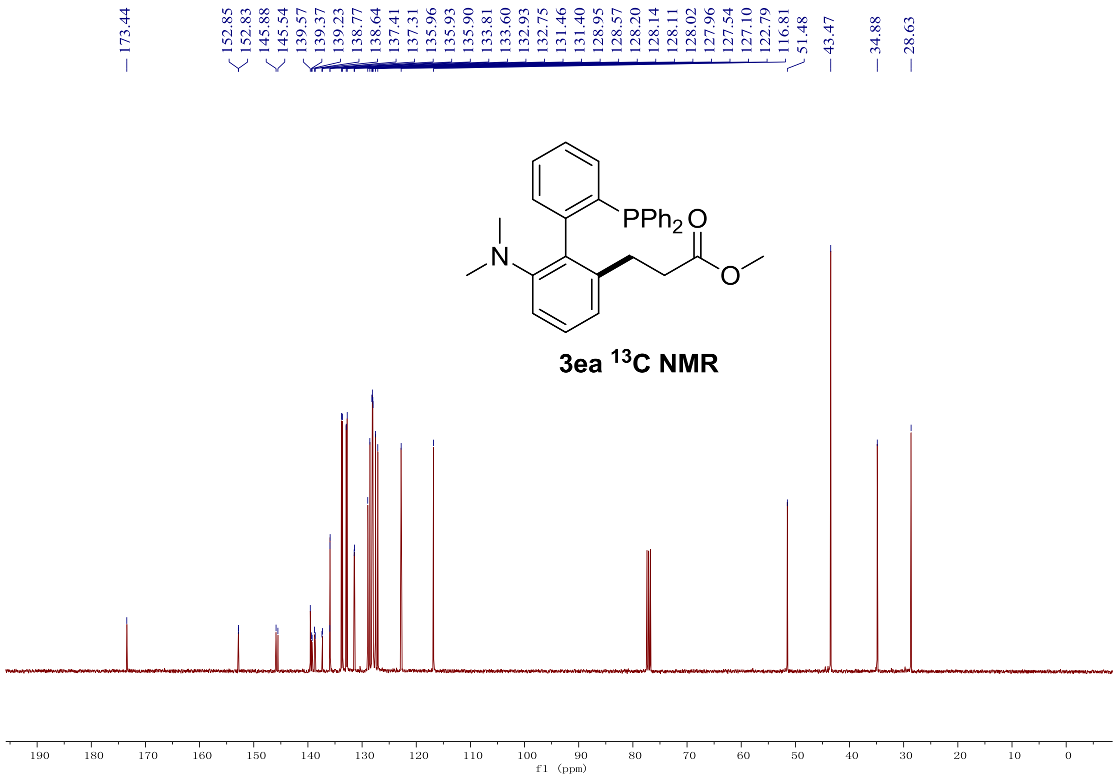

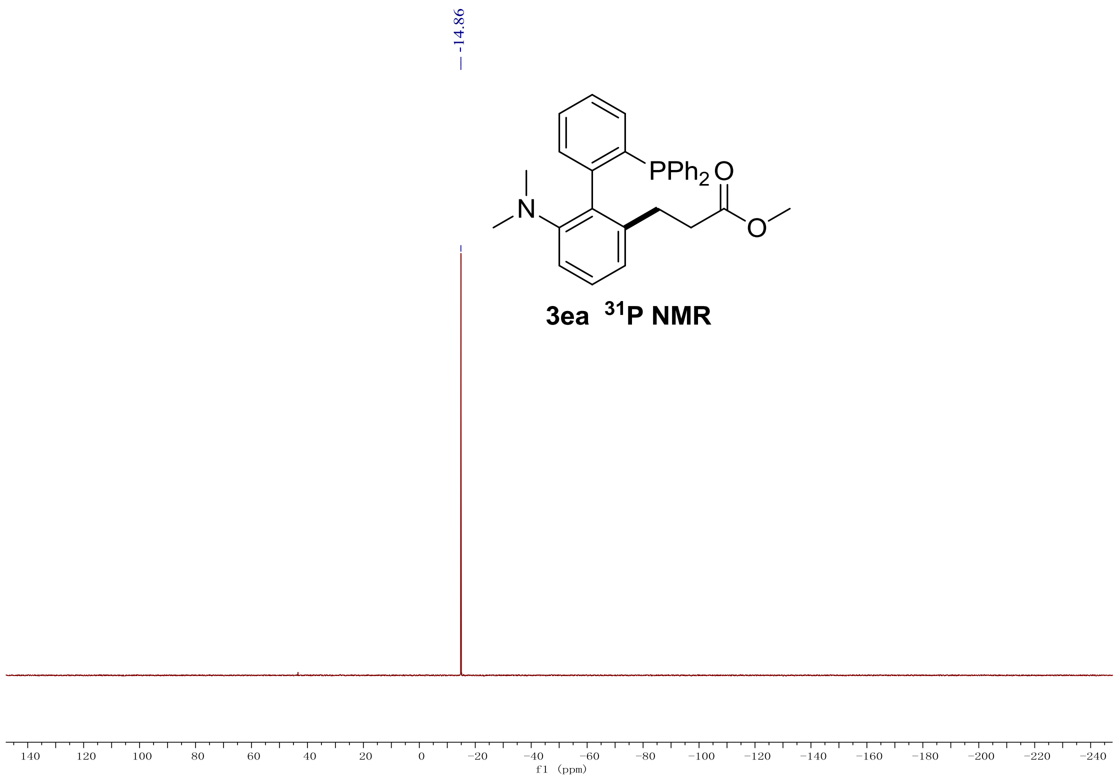

Supplementary Figure 14. ^{1}H, ^{13}C and ^{31}P NMR of compound **3ea.**

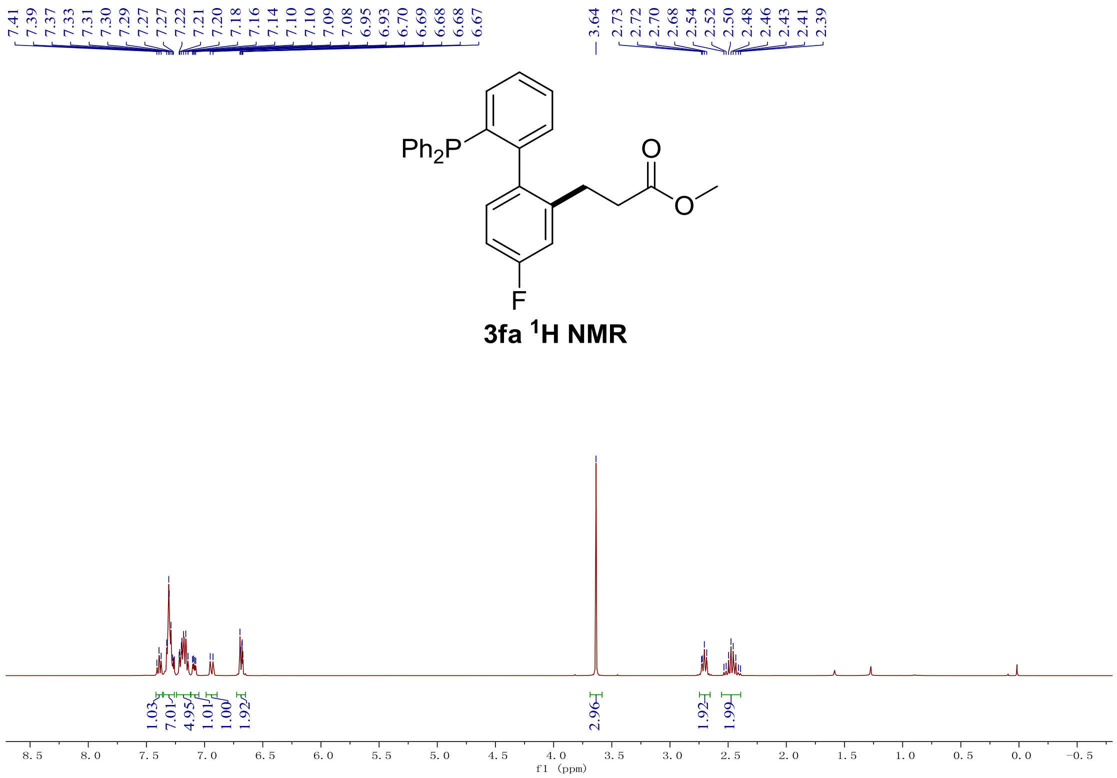

3fa ^{1}H NMR

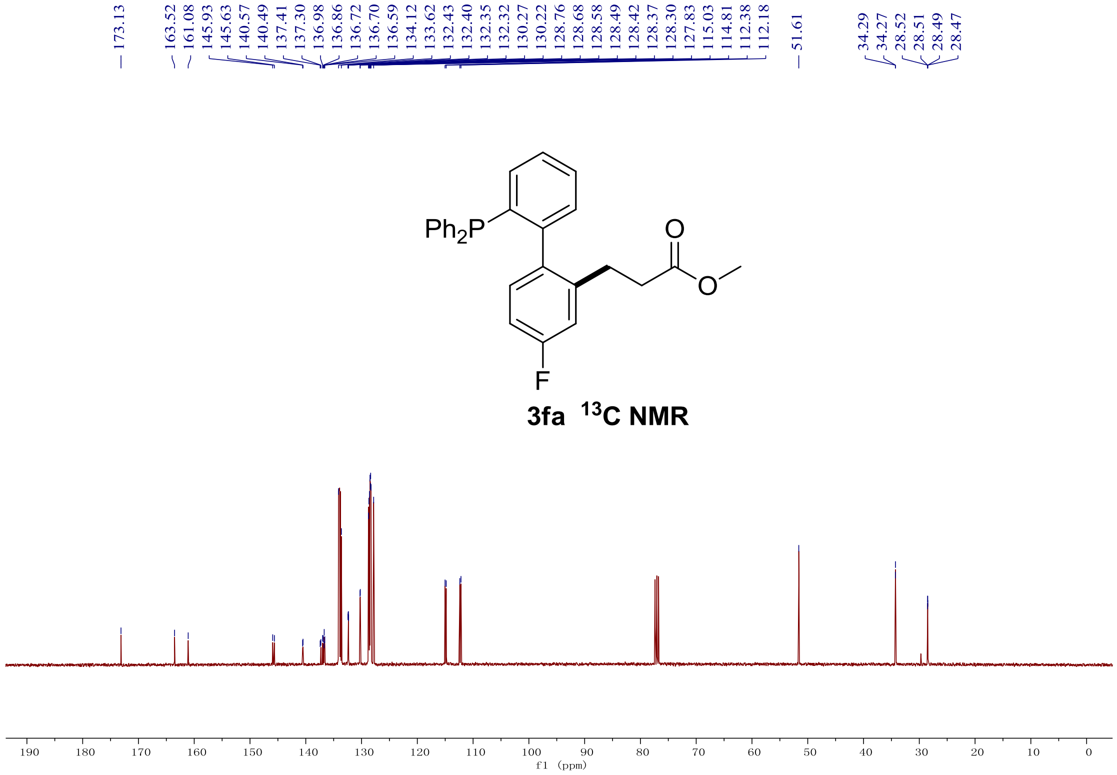

3fa ^{13}C NMR

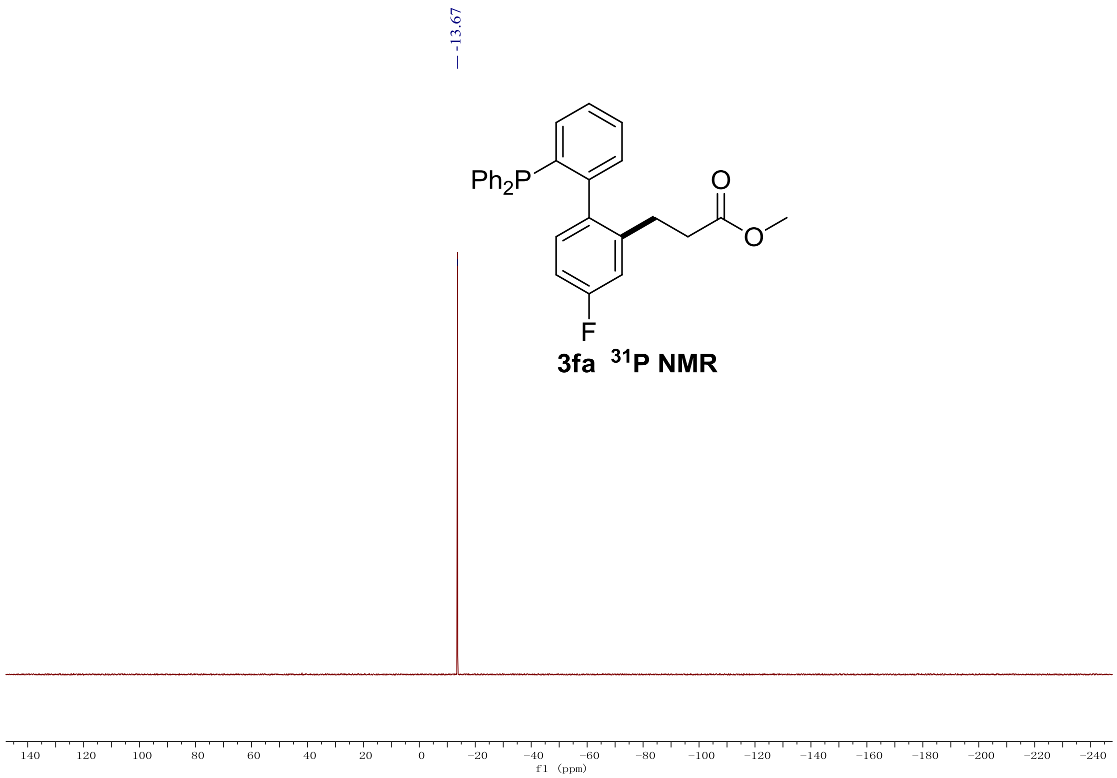

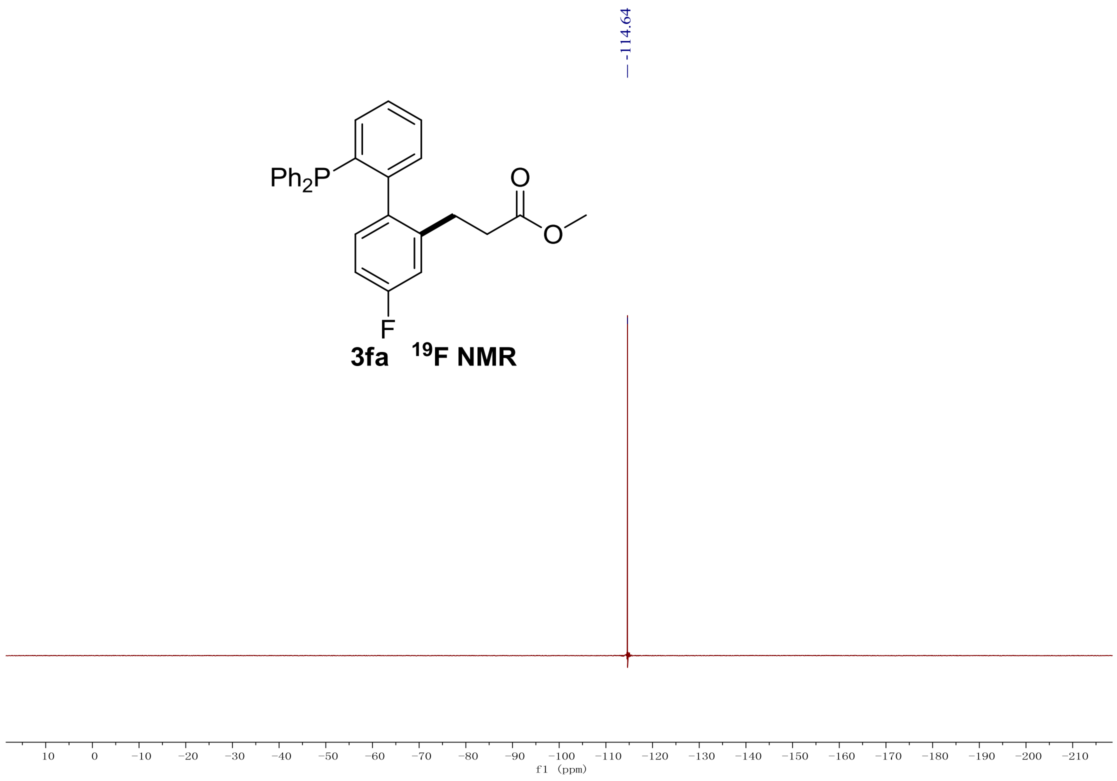

Supplementary Figure 15. ^{1}H, ^{13}C, ^{31}P and ^{19}F NMR of compound **3fa.**

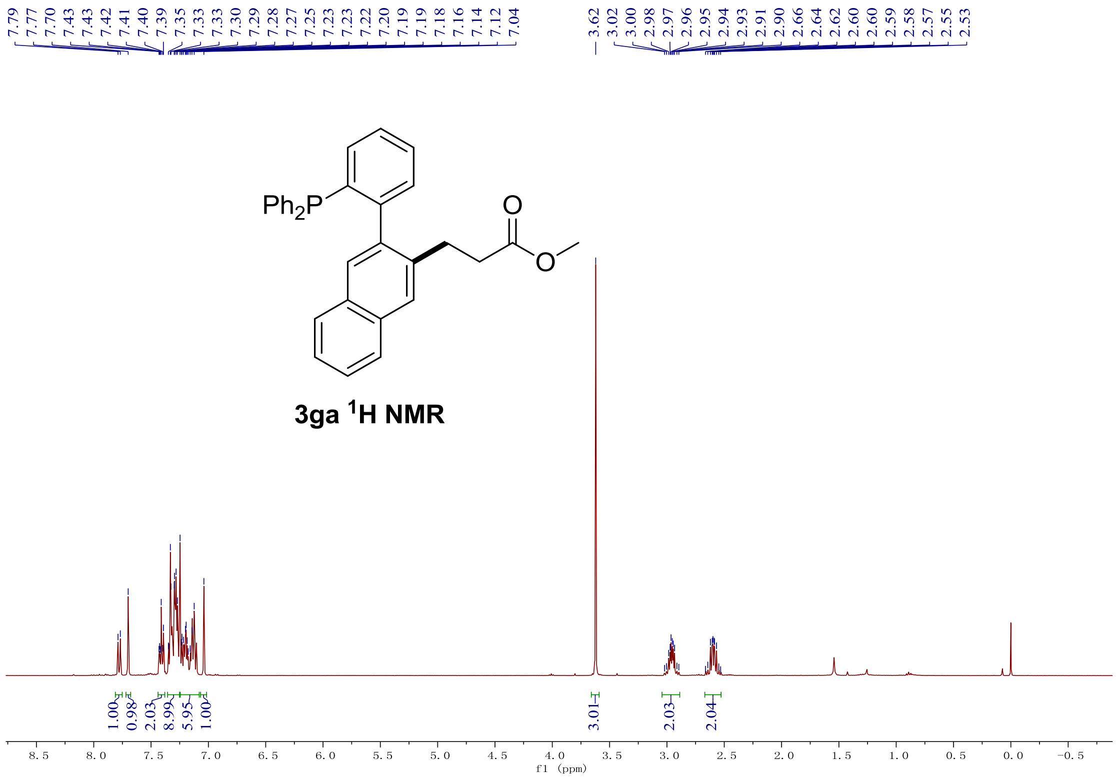

3ga ^{1}H NMR

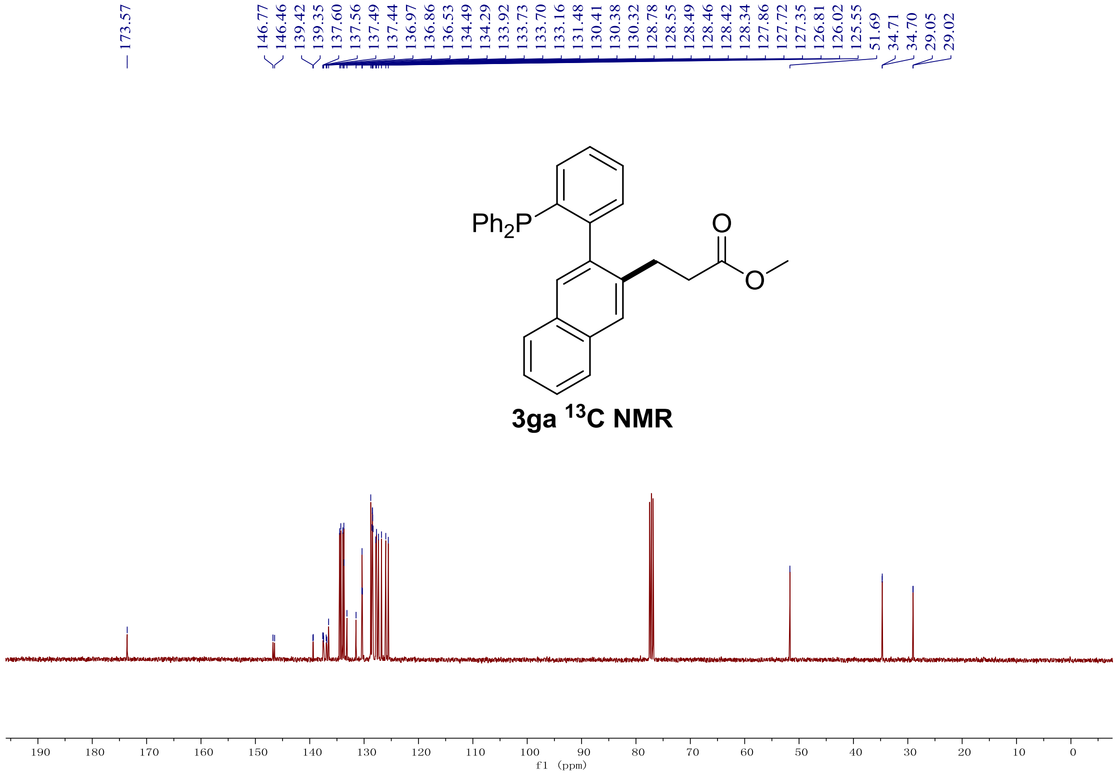

3ga ^{13}C NMR

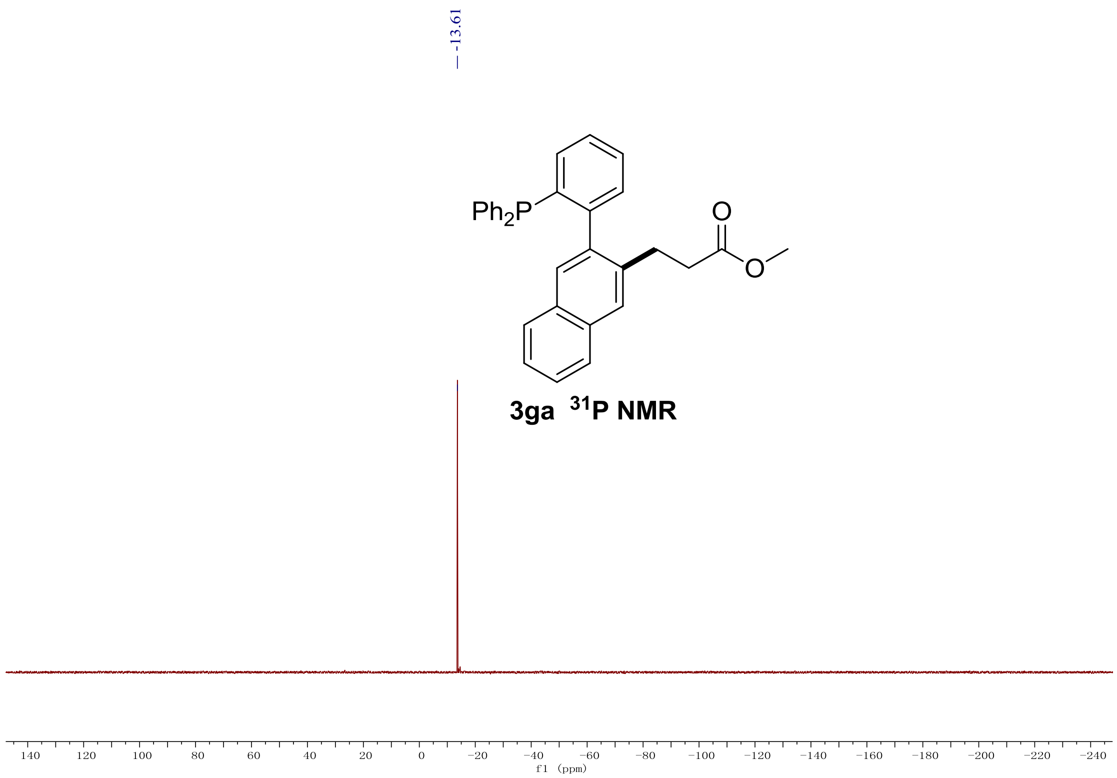

Supplementary Figure 16. ^{1}H, ^{13}C and ^{31}P NMR of compound **3ga.**

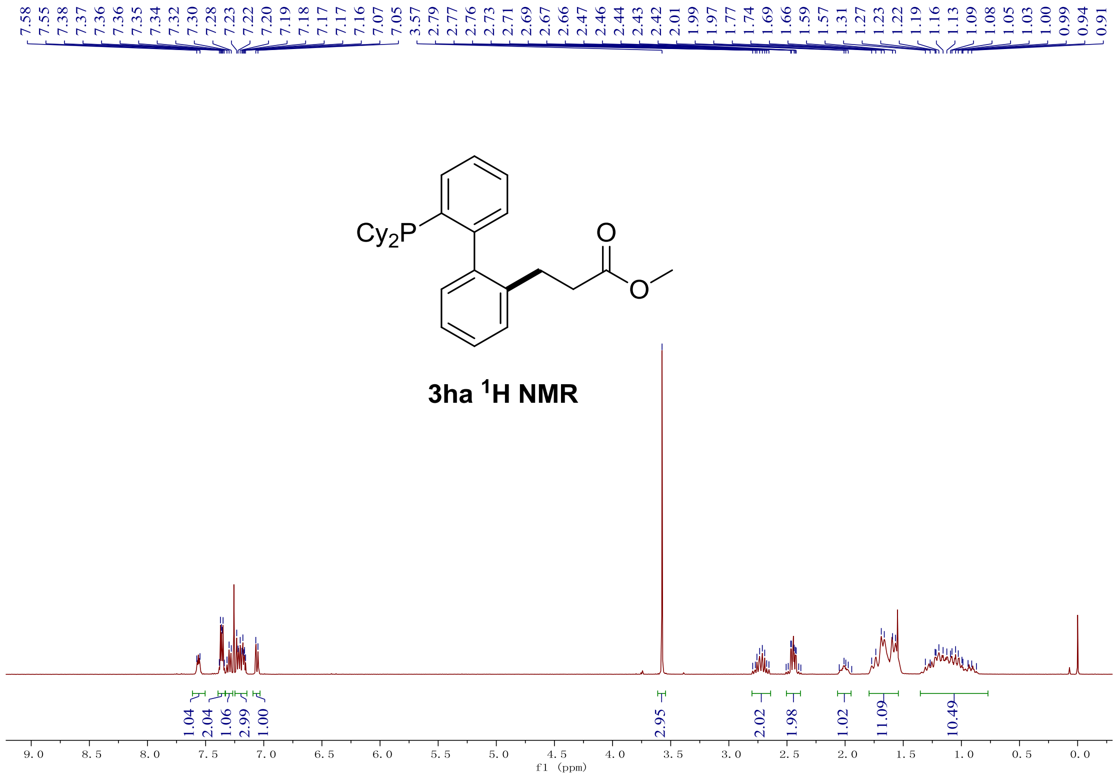

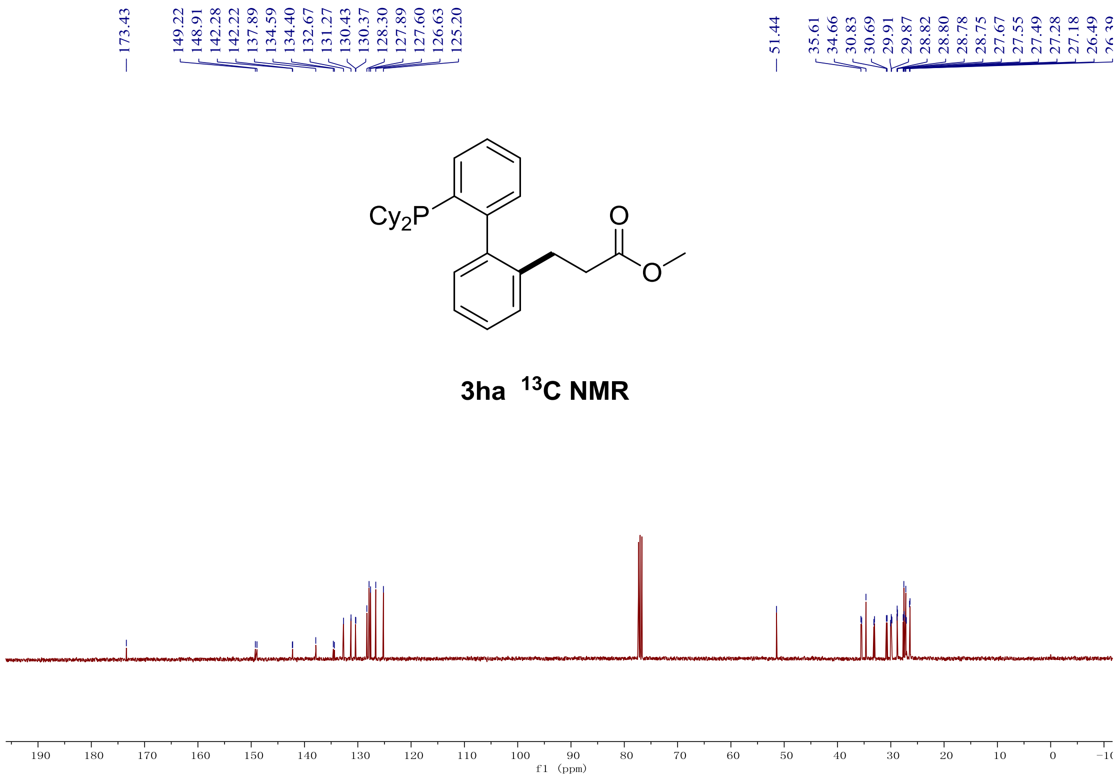

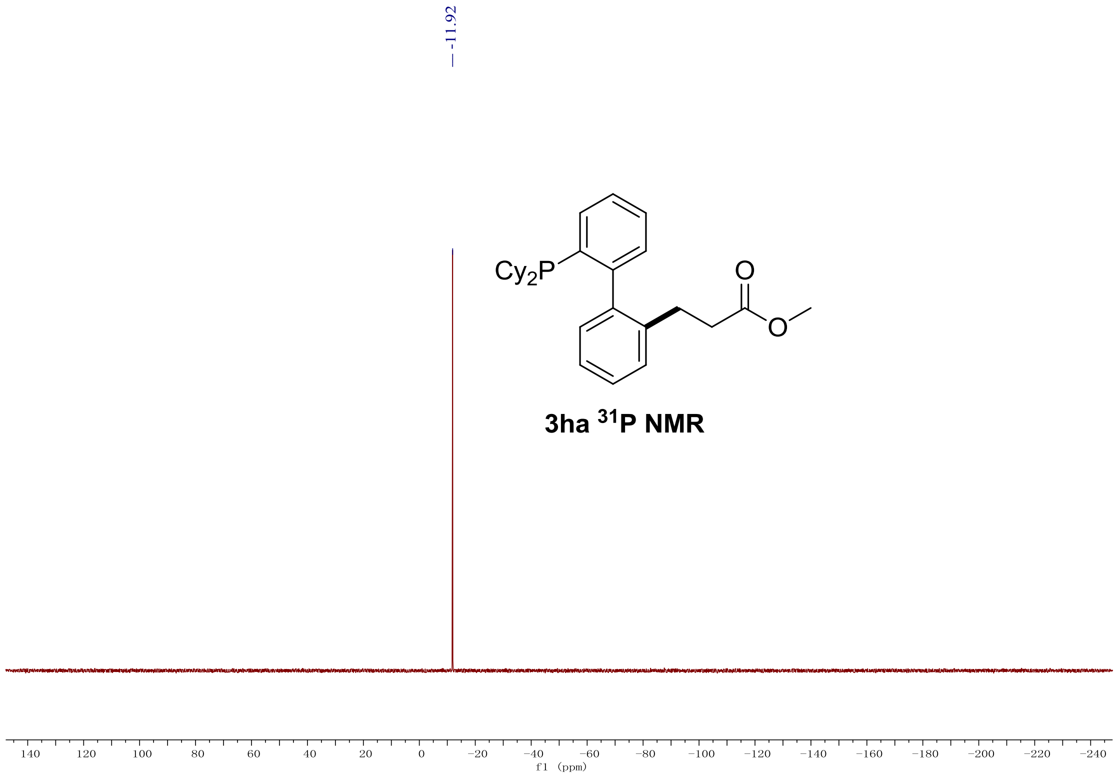

Supplementary Figure 17. ^{1}H, ^{13}C and ^{31}P NMR of compound **3ha.**

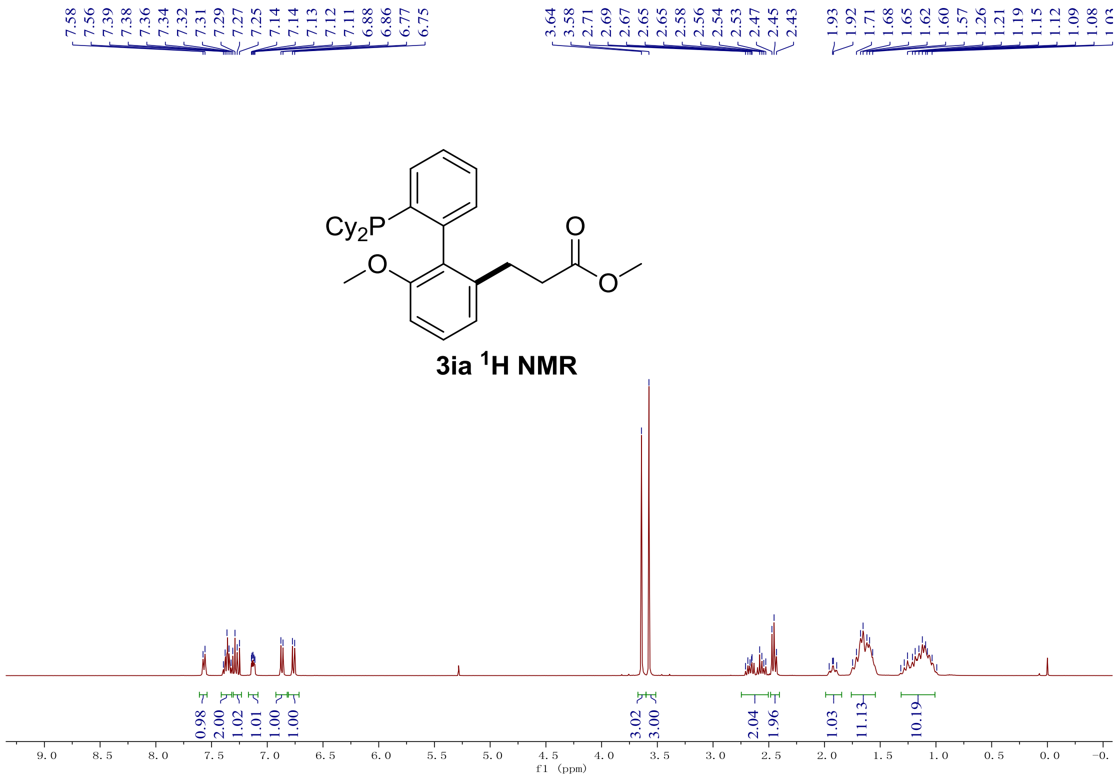
3ia ^{1}H NMR
f1 (ppm)

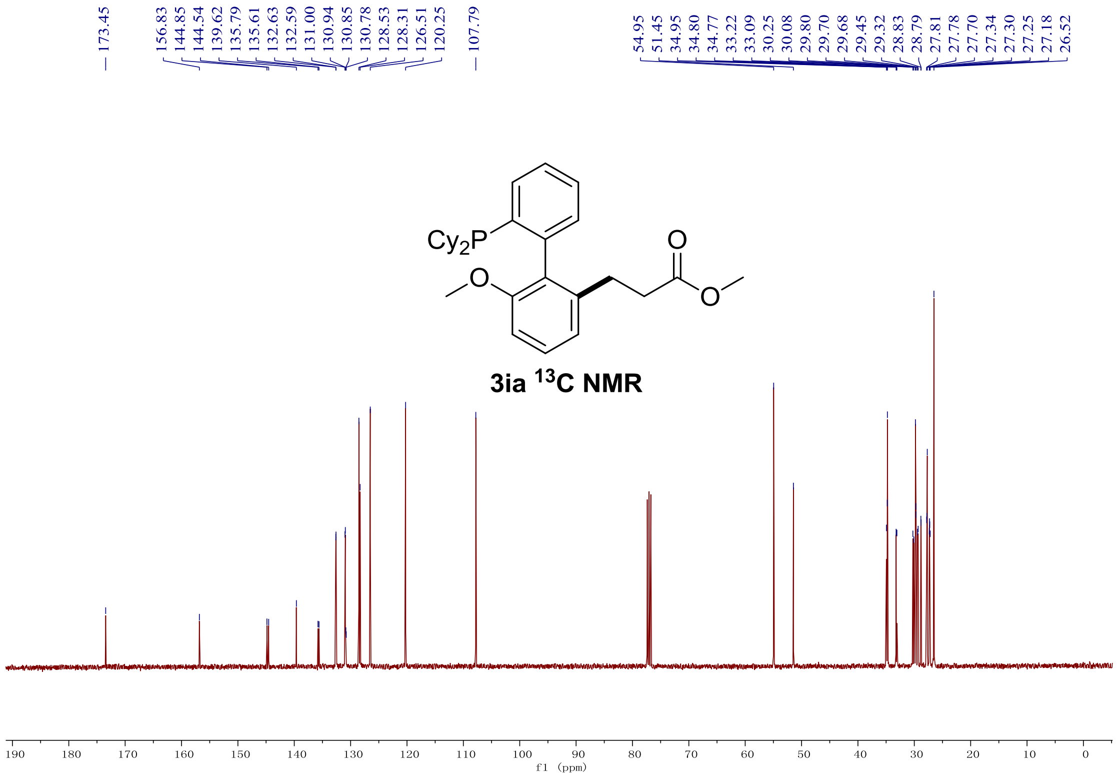
3ia ^{13}C NMR
f1 (ppm)

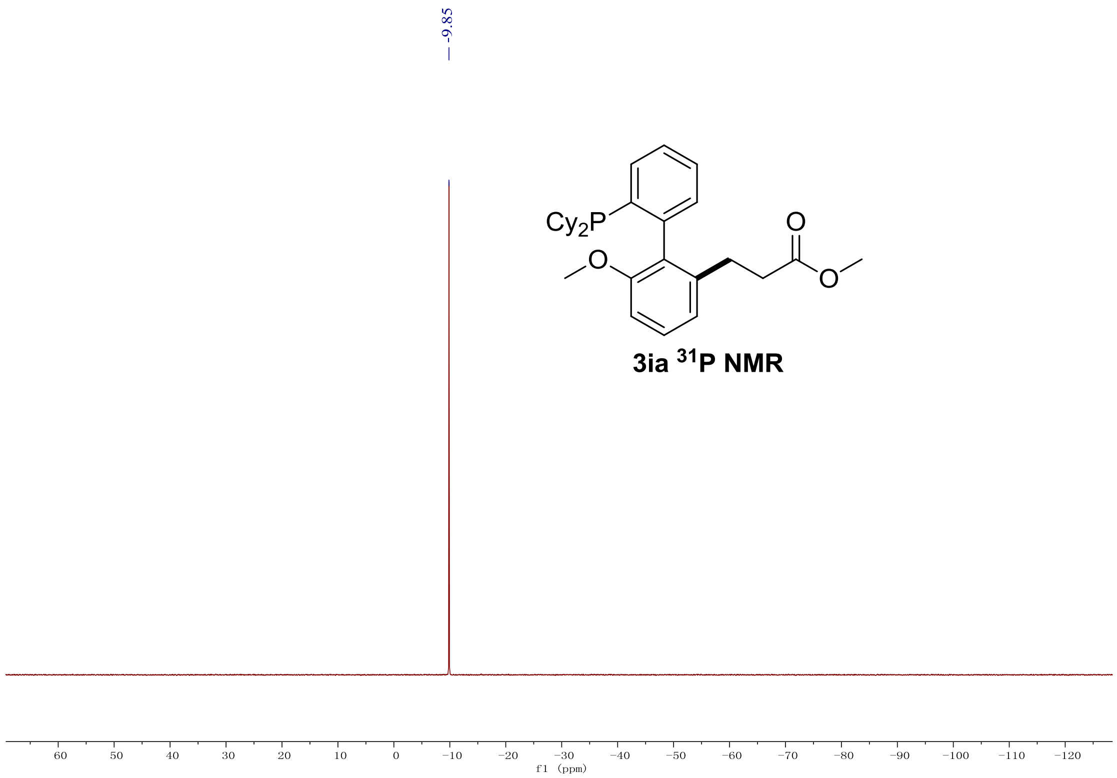

Supplementary Figure 18. ^{1}H, ^{13}C and ^{31}P NMR of compound **3ia.**

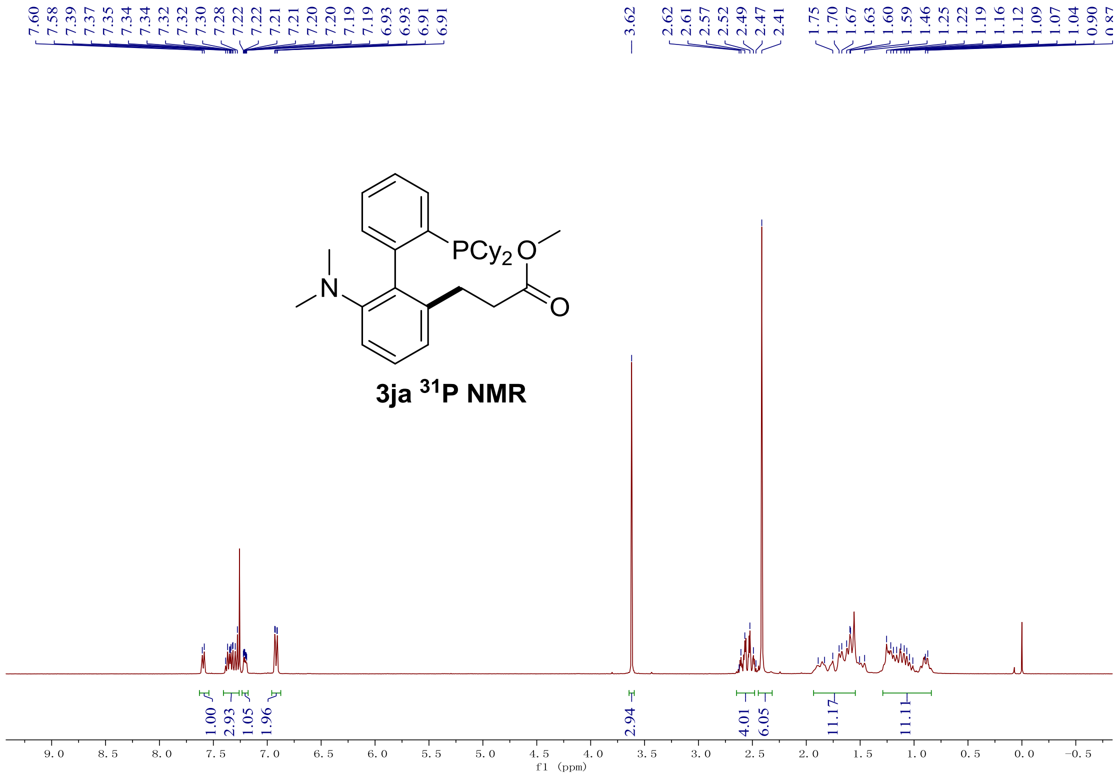

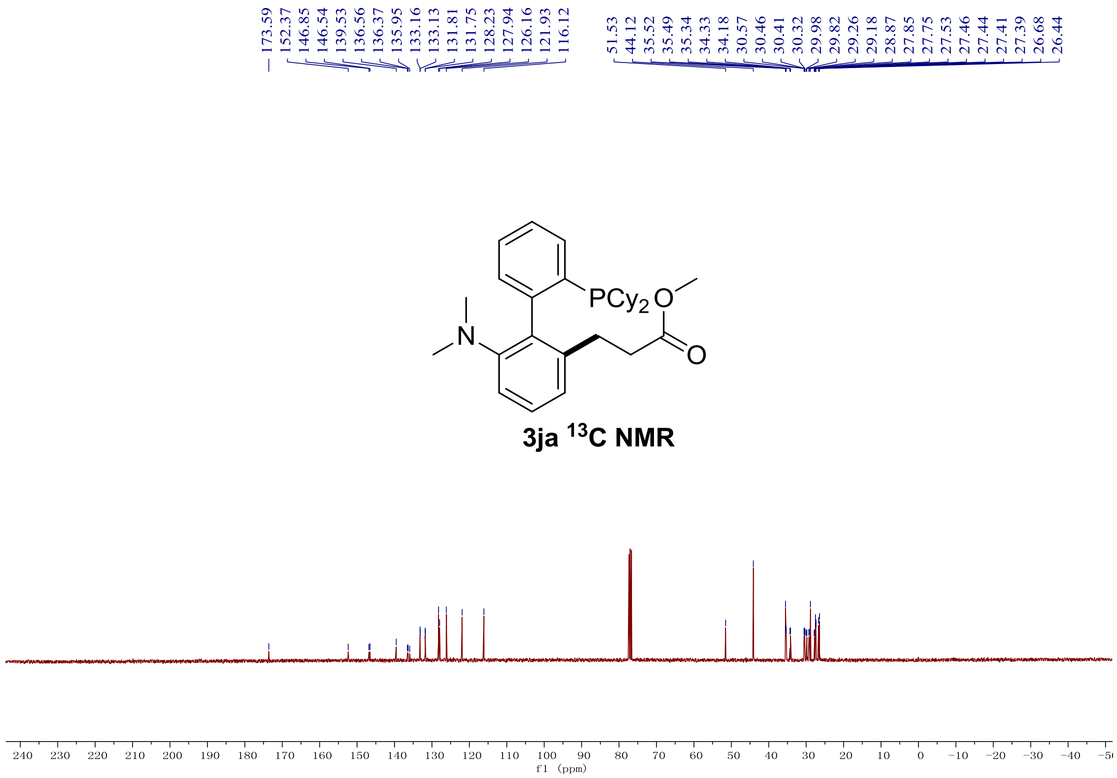

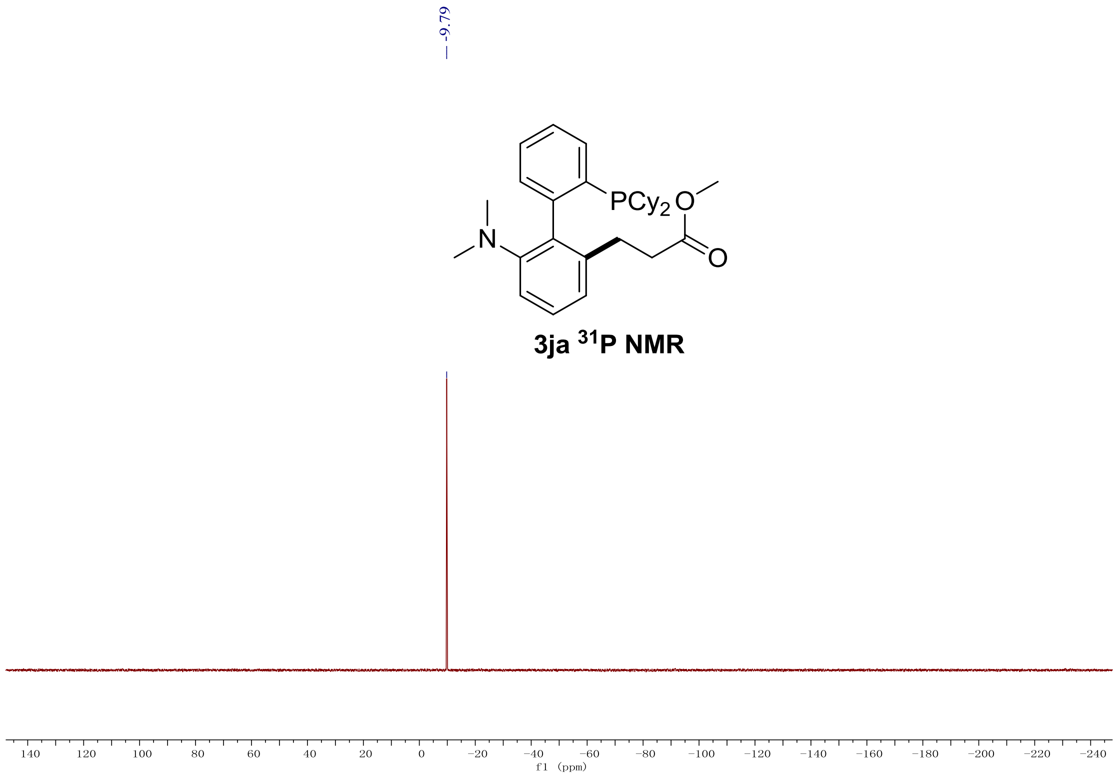

Supplementary Figure 19. ^{1}H, ^{13}C and ^{31}P NMR of compound **3ja.**

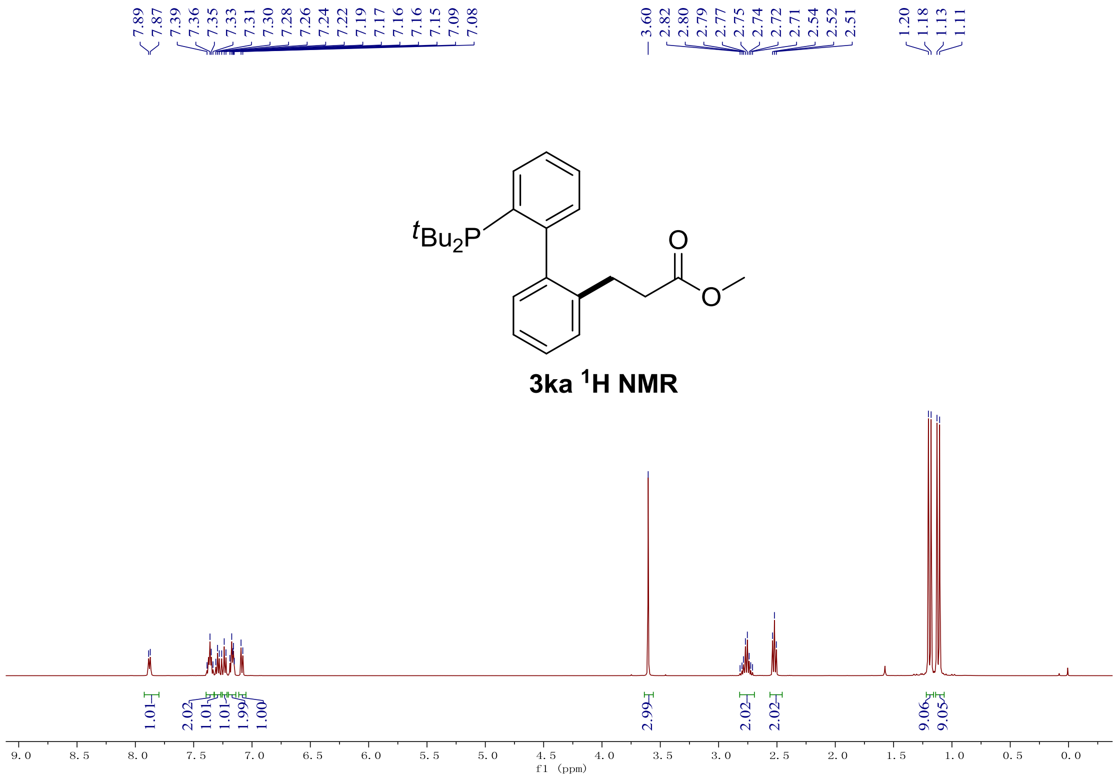
7.89
7.87
7.39
7.36
7.35
7.33
7.31
7.30
7.28
7.26
7.24
7.22
7.19
7.17
7.16
7.16
7.15
7.09
7.08
3.60
2.82
2.80
2.79
2.77
2.75
2.74
2.72
2.71
2.54
2.52
2.51
1.20
1.18
1.13
1.11
tBu2P
O
O
3ka 1H NMR
1.01
2.02
1.01
1.01
1.99
1.00
2.99
2.02
2.02
9.06
9.05
9.0
8.5
8.0
7.5
7.0
6.5
6.0
5.5
5.0
4.5
4.0
3.5
3.0
2.5
2.0
1.5
1.0
0.5
0.0
f1 (ppm)

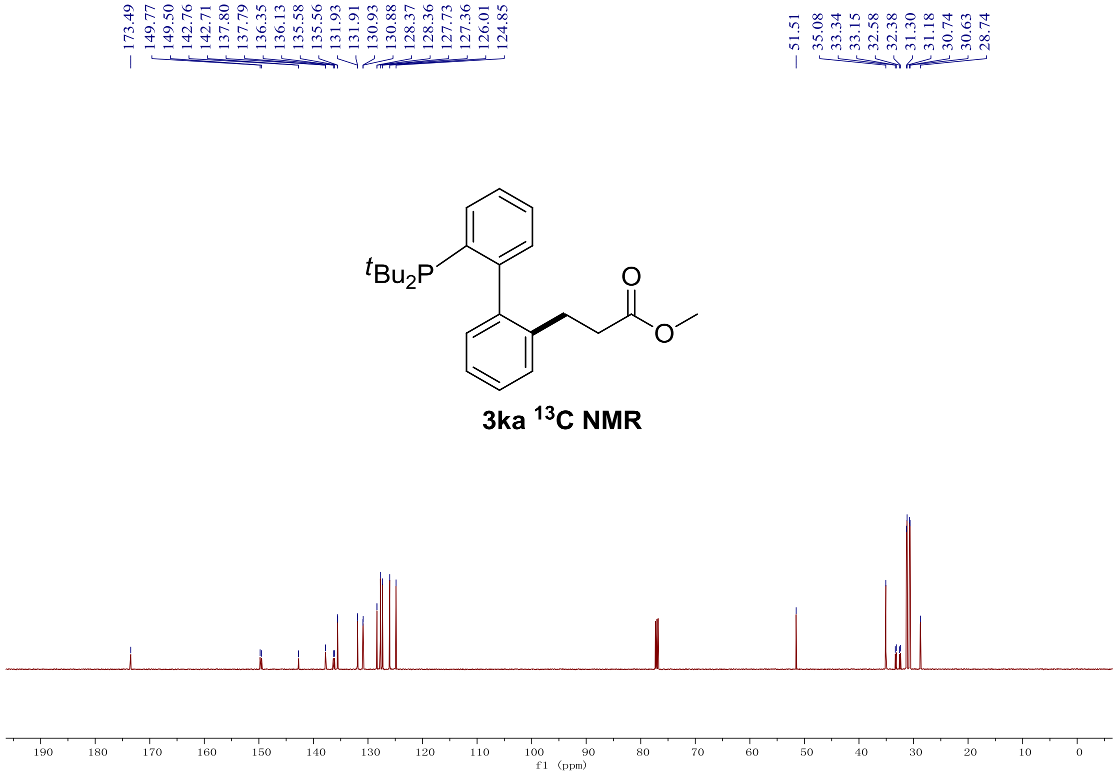
173.49
149.77
149.50
142.76
142.71
137.80
137.79
136.35
136.13
135.58
135.56
131.93
131.91
130.93
130.88
128.37
128.36
127.73
127.36
126.01
124.85
51.51
35.08
33.34
33.15
32.58
32.38
31.30
31.18
30.74
30.63
28.74
tBu2P
O
O
3ka 13C NMR
190
180
170
160
150
140
130
120
110
100
90
80
70
60
50
40
30
20
10
0
f1 (ppm)

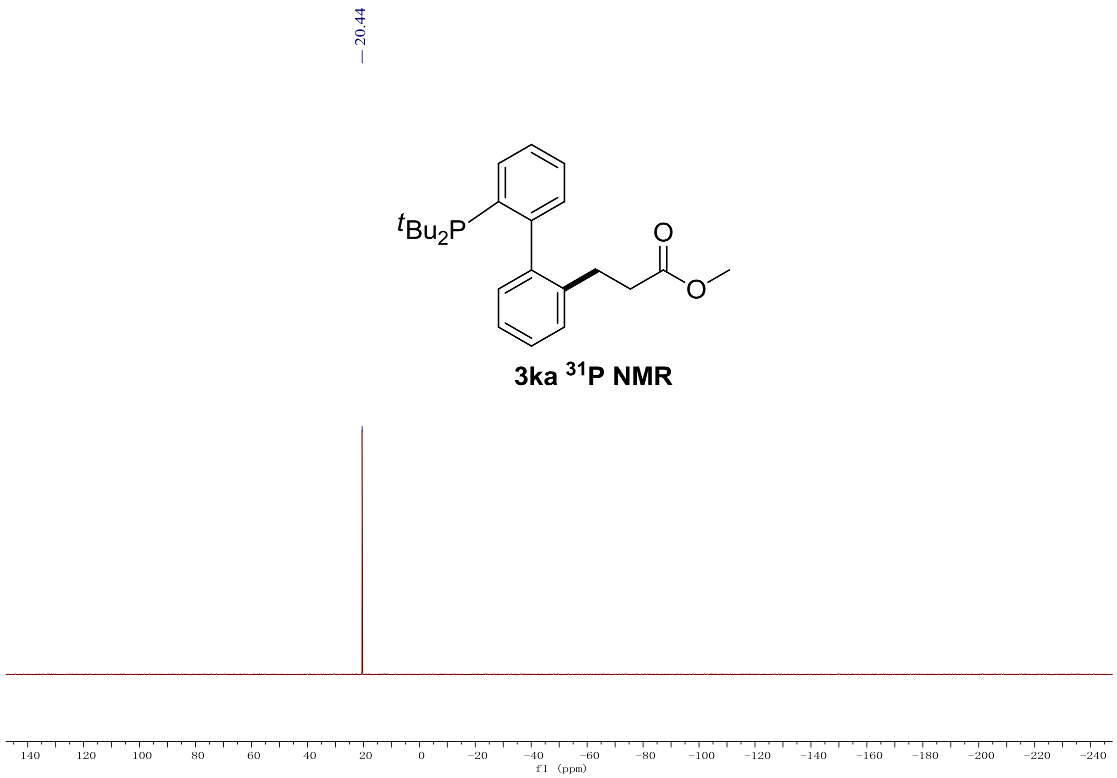

Supplementary Figure 20. ^{1}H, ^{13}C and ^{31}P NMR of compound **3ka.**

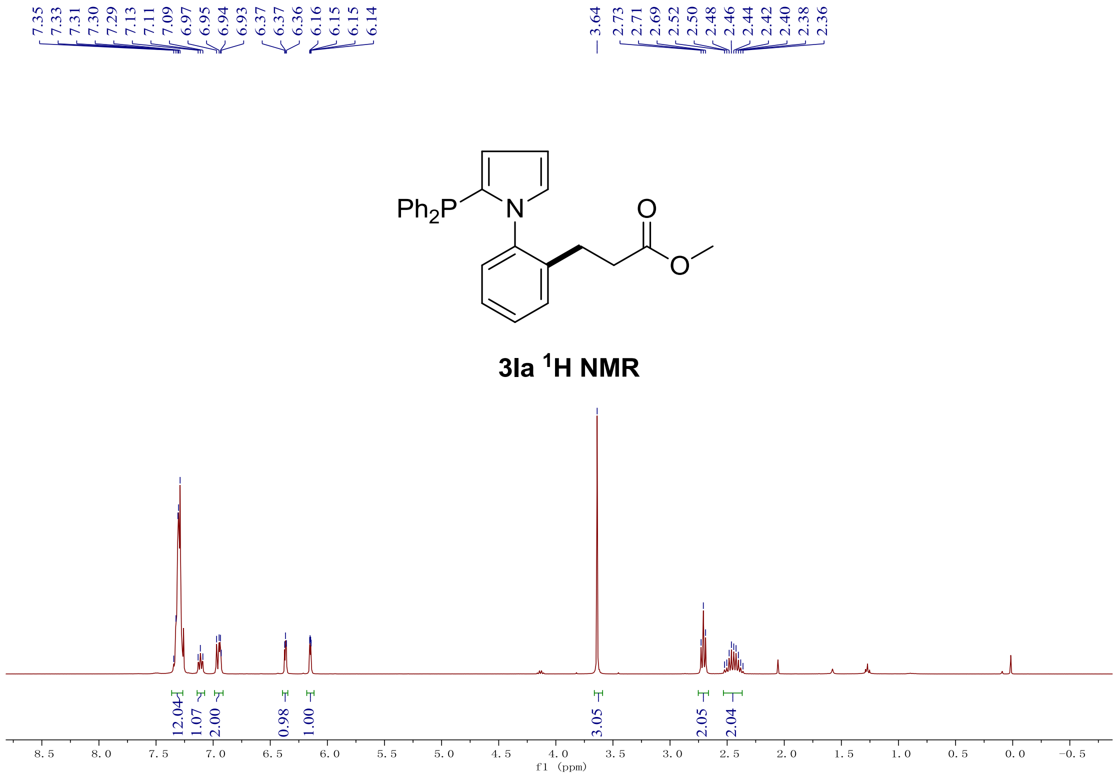

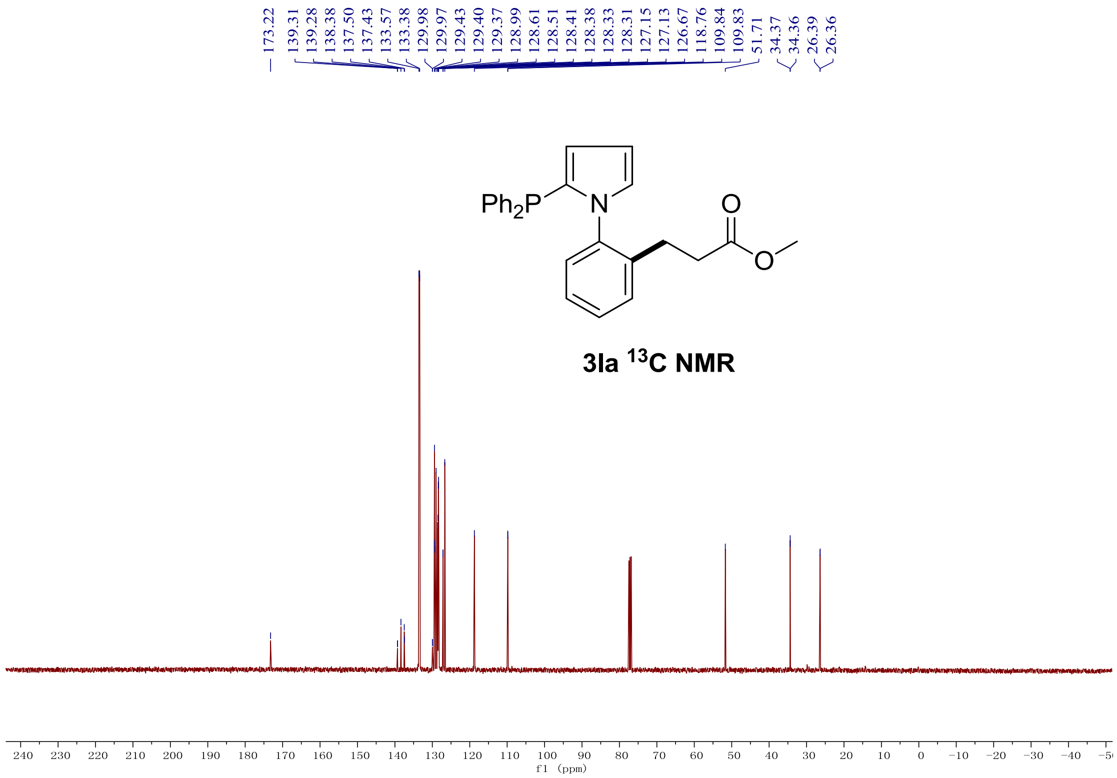

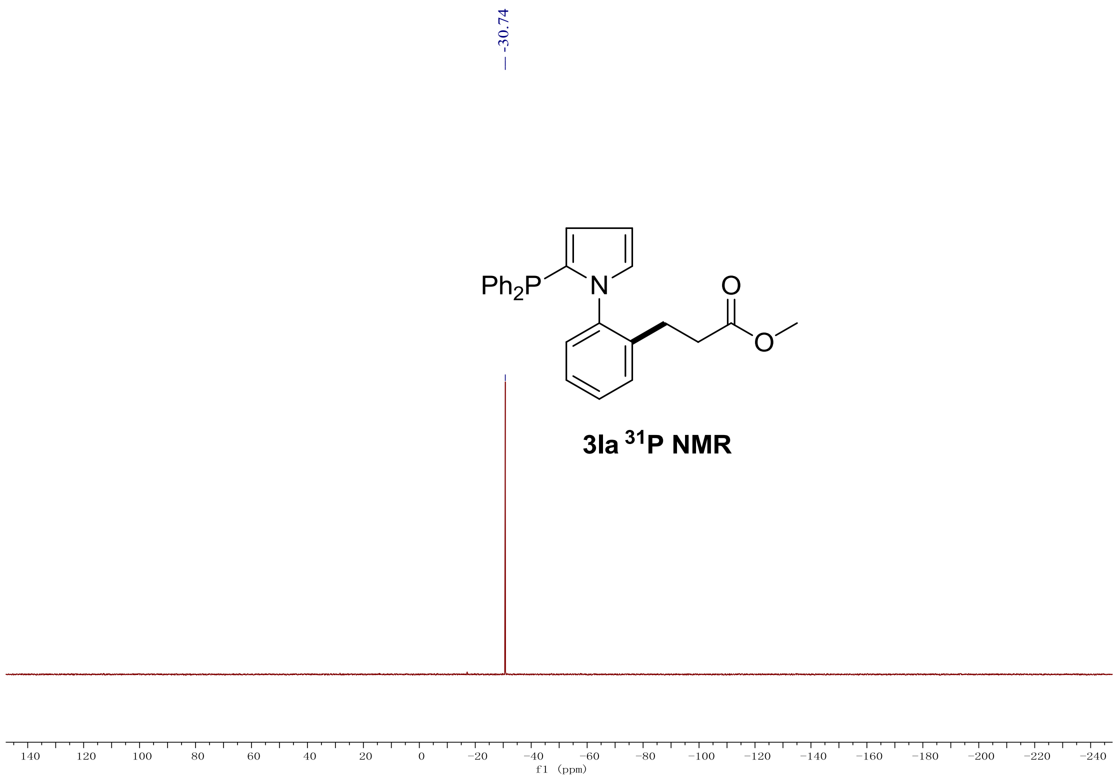

Supplementary Figure 21. ^{1}H, ^{13}C and ^{31}P NMR of compound **3la.**

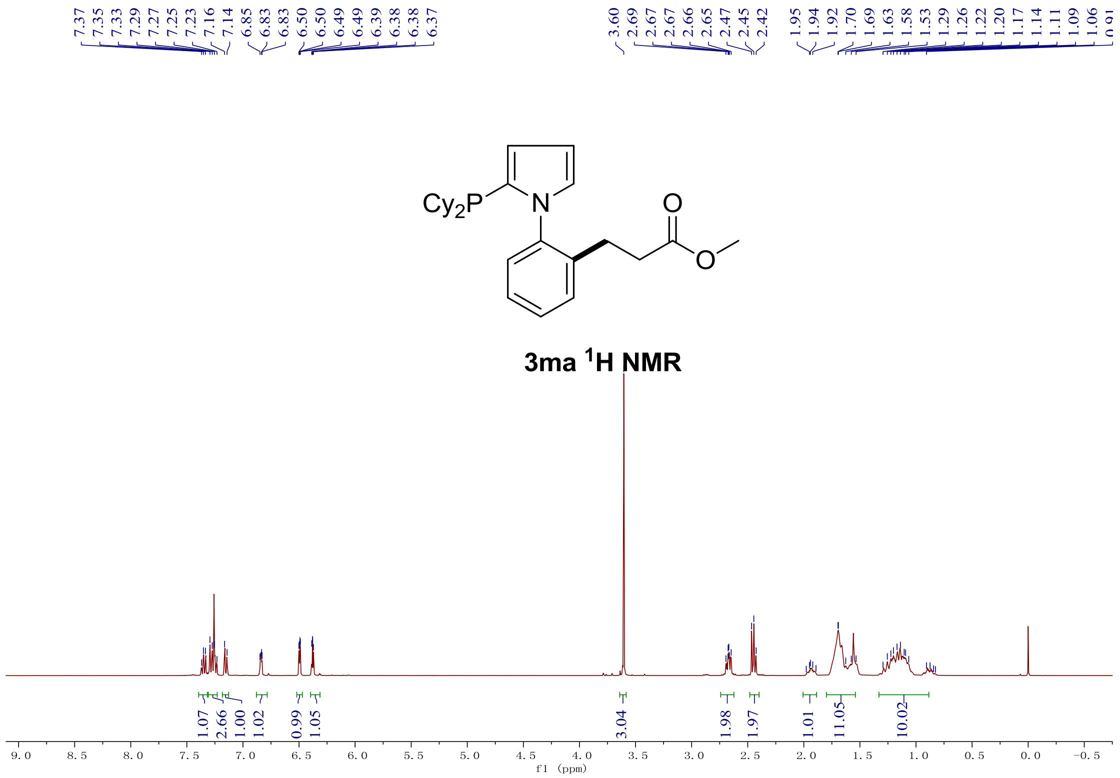
7.37 7.35 7.33 7.29 7.27 7.25 7.23 7.16 7.14 6.85 6.83 6.83 6.50 6.50 6.49 6.49 6.39 6.38 6.38 6.37
3.60 2.69 2.67 2.67 2.66 2.65 2.47 2.45 2.42 1.95 1.94 1.92 1.70 1.69 1.63 1.58 1.53 1.29 1.26 1.22 1.20 1.17 1.14 1.11 1.09 1.06
Cy2P
N
O
O
3ma 1H NMR
1.07 2.66 1.00 1.02 0.99 1.05 3.04 1.98 1.97 1.01 11.05 10.02
9.0 8.5 8.0 7.5 7.0 6.5 6.0 5.5 5.0 4.5 4.0 3.5 3.0 2.5 2.0 1.5 1.0 0.5 0.0 -0.5
f1 (ppm)

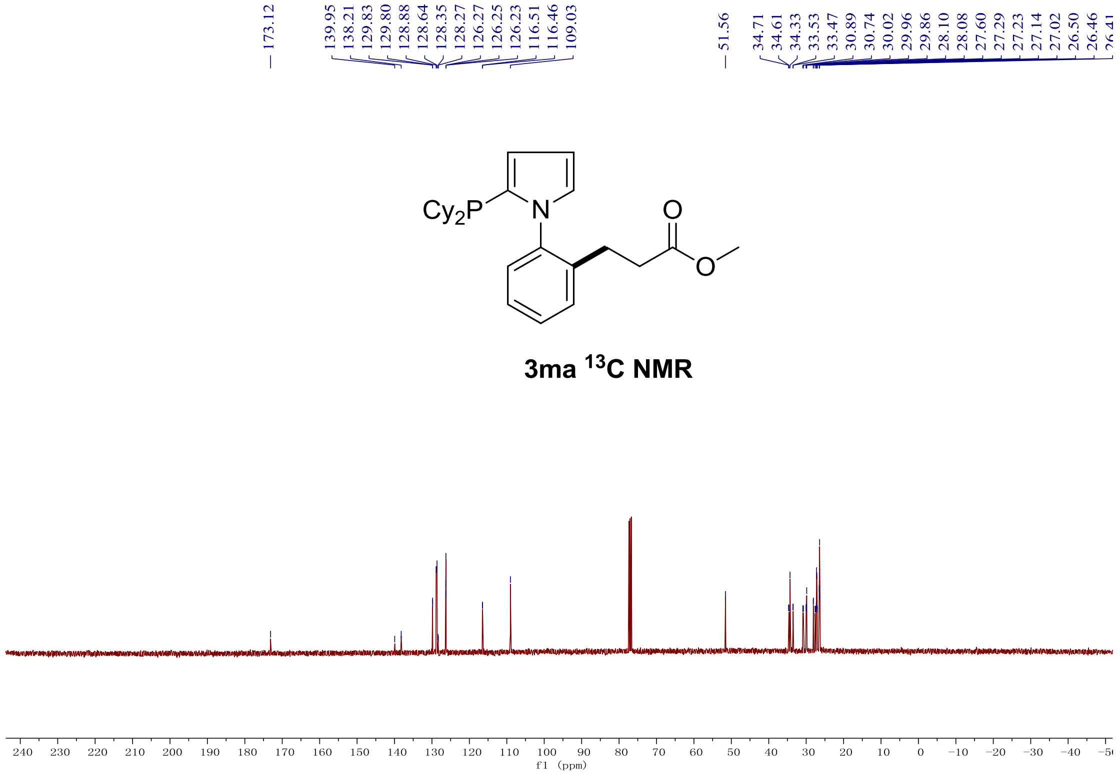
173.12
139.95 138.21 129.83 129.80 128.88 128.64 128.35 128.27 126.27 126.25 126.23 116.51 116.46 109.03
51.56 34.71 34.61 34.33 33.53 33.47 30.89 30.74 30.02 29.96 29.86 28.10 28.08 27.60 27.29 27.23 27.14 27.02 26.50 26.46
Cy2P
N
O
O
3ma 13C NMR
240 230 220 210 200 190 180 170 160 150 140 130 120 110 100 90 80 70 60 50 40 30 20 10 0 -10 -20 -30 -40
f1 (ppm)

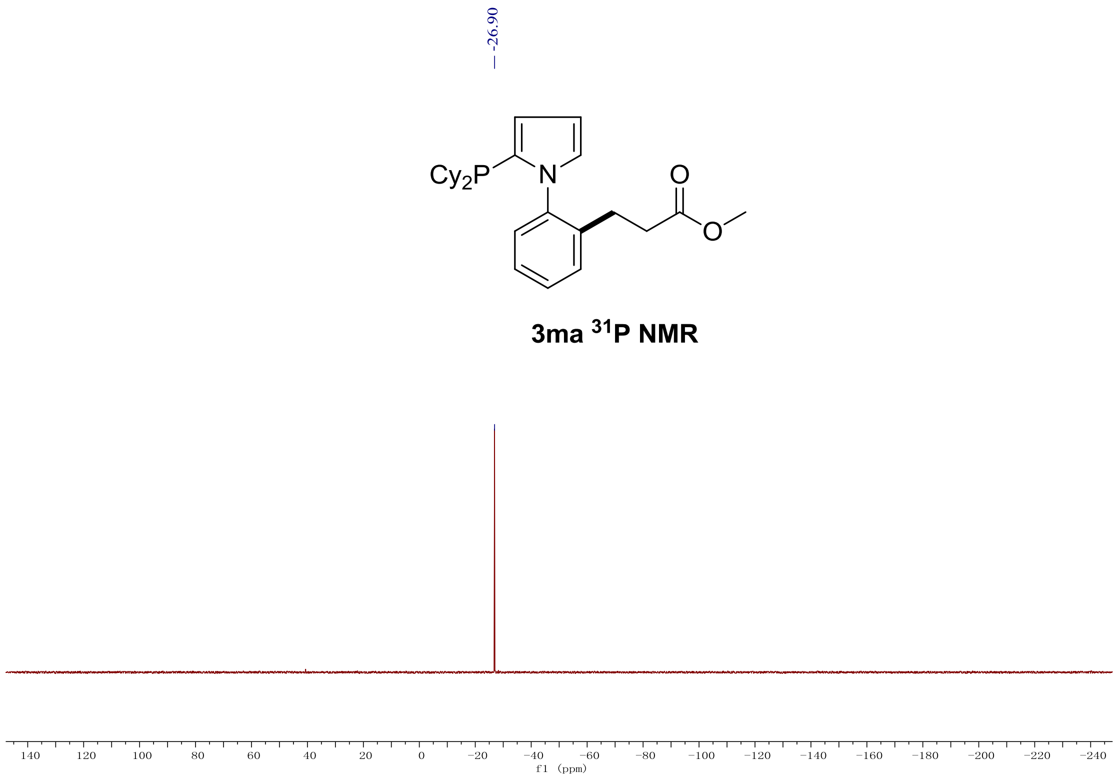

3ma ^{31}P NMR

Supplementary Figure 22. ^{1}H, ^{13}C and ^{31}P NMR of compound **3ma.**

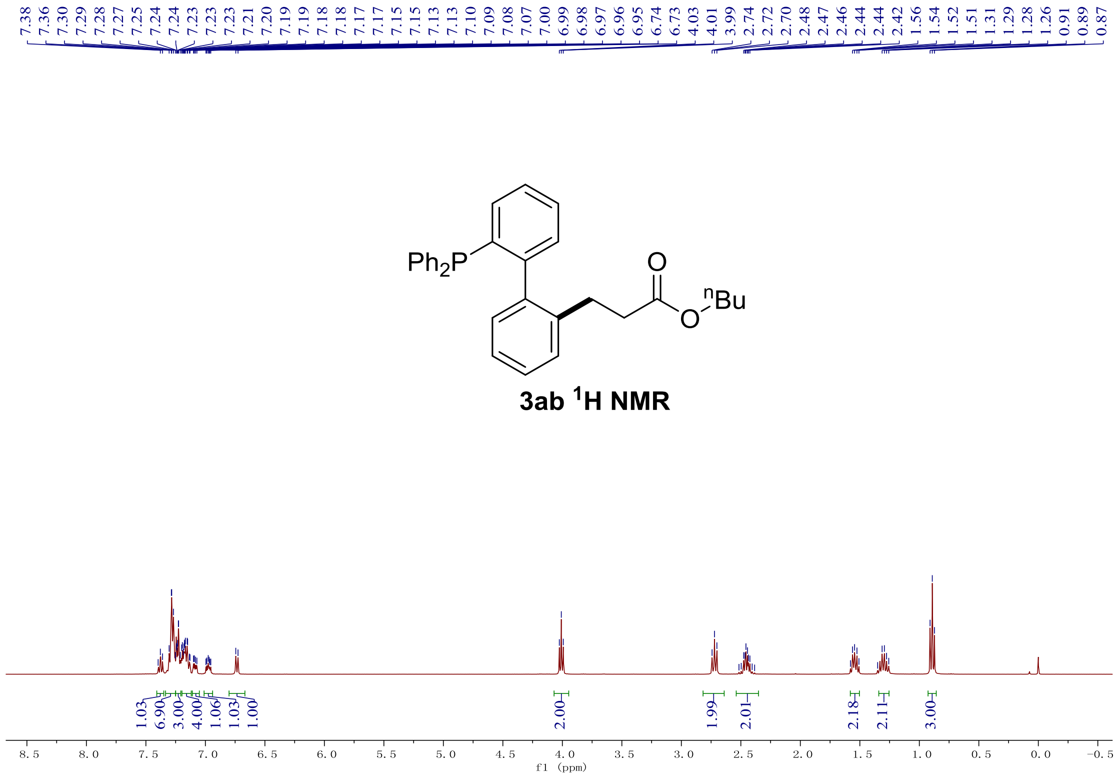

3ab ^{1}H NMR

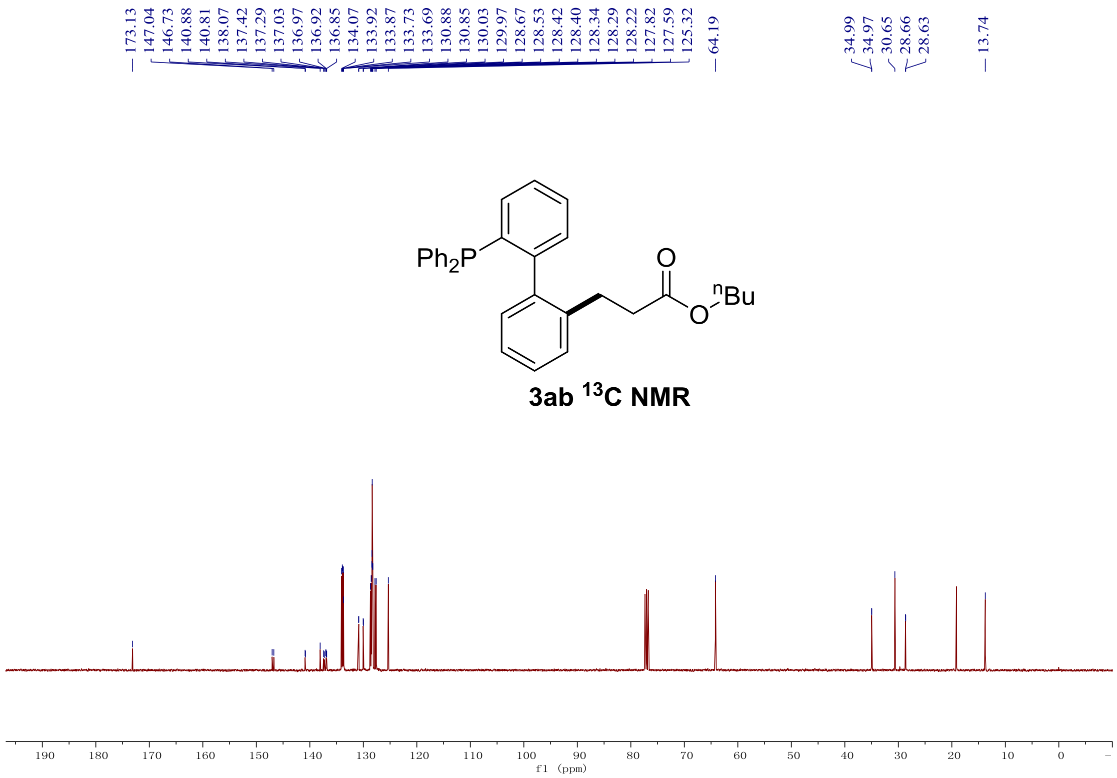

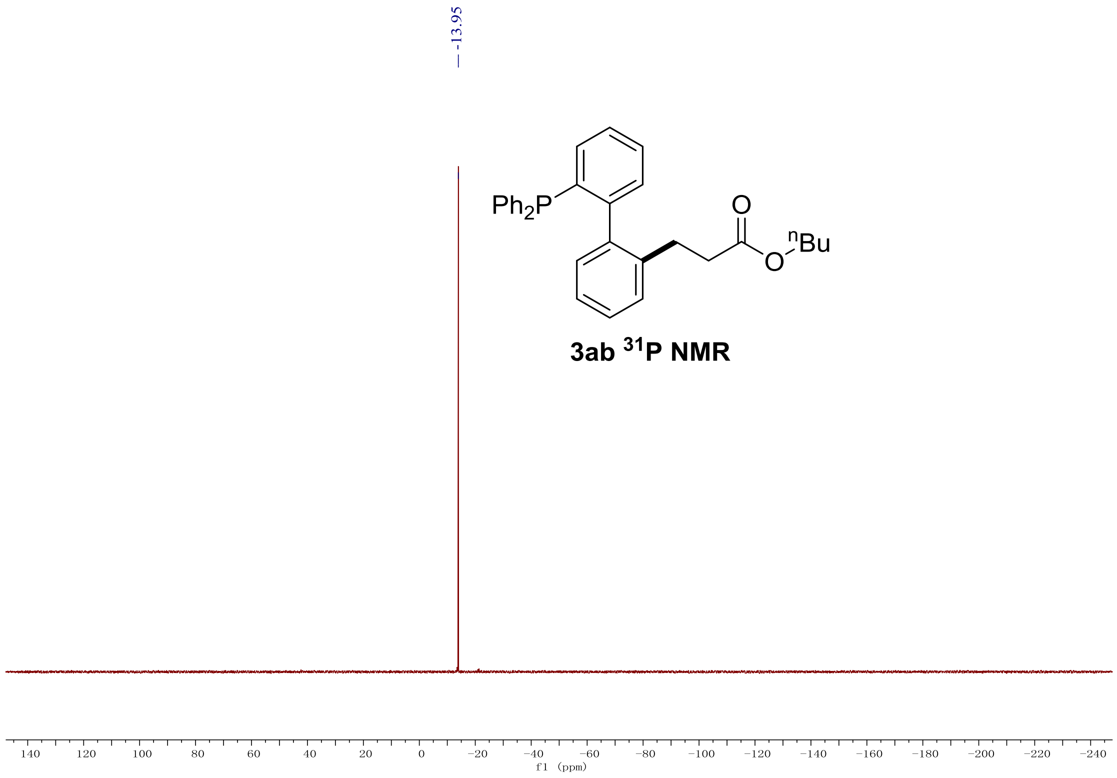

Supplementary Figure 23. ^{1}H, ^{13}C and ^{31}P NMR of compound **3ab.**

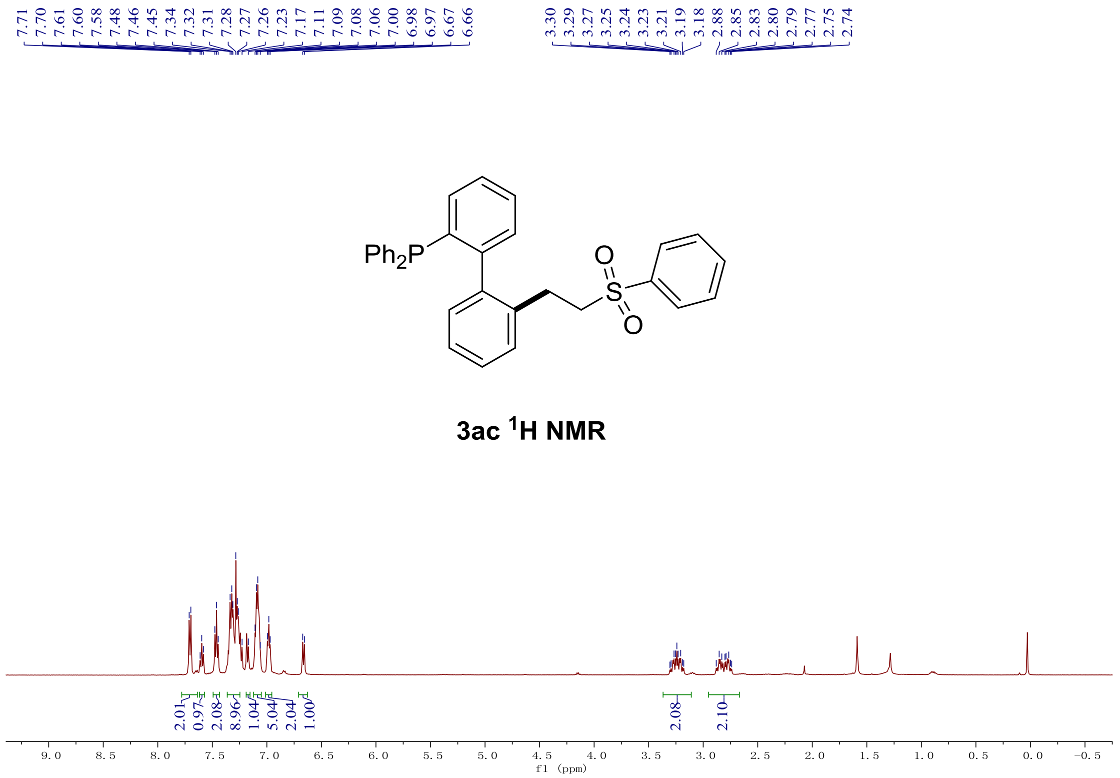

3ac ^{1}H NMR

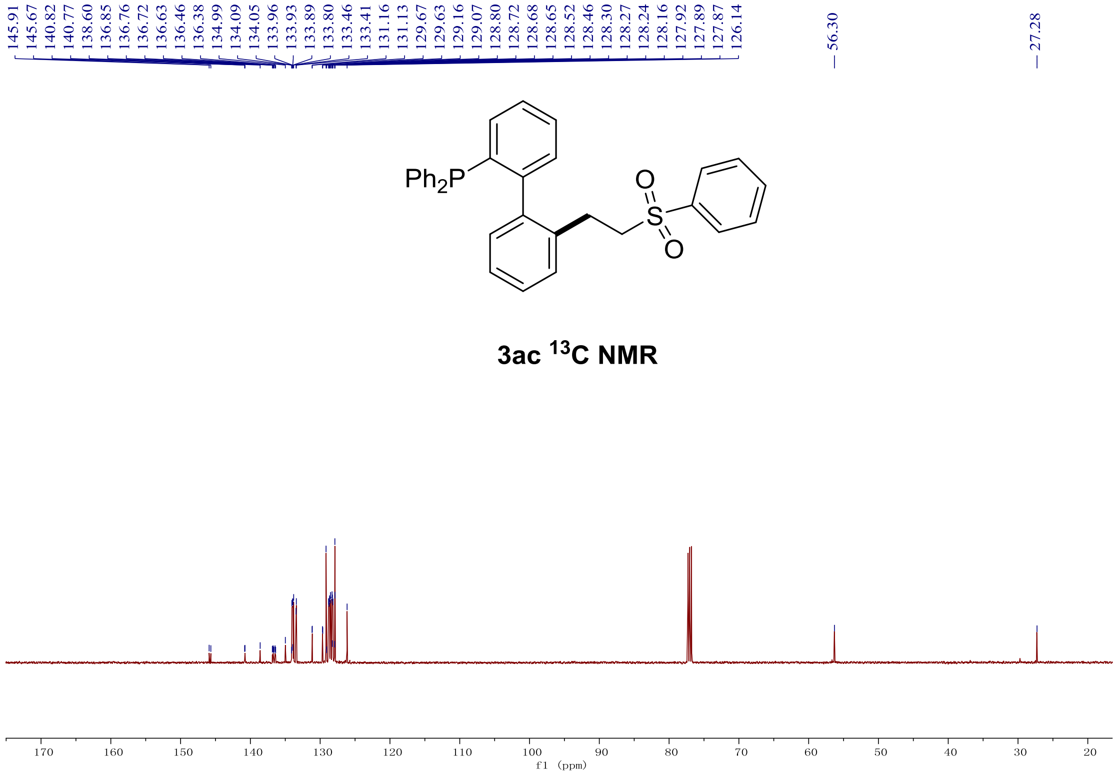

3ac ^{13}C NMR

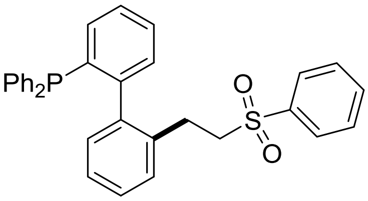

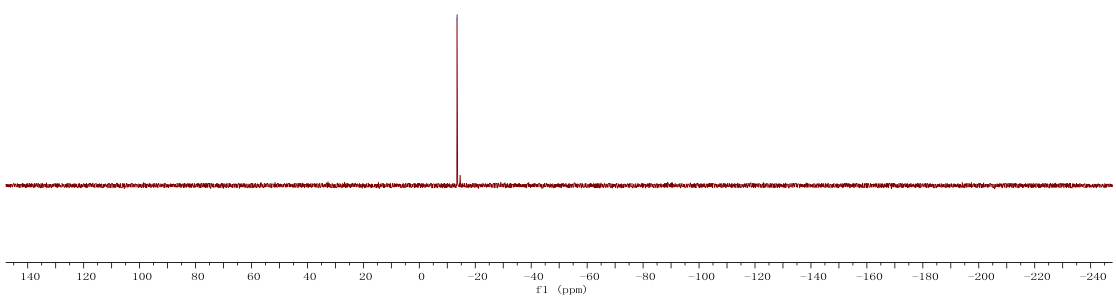

3ac ^{31}P NMR

Supplementary Figure 24. ^{1}H, ^{13}C and ^{31}P NMR of compound **3ac.**

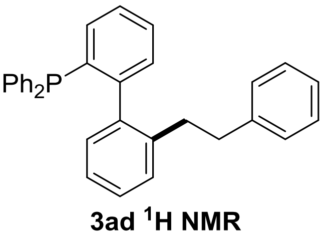

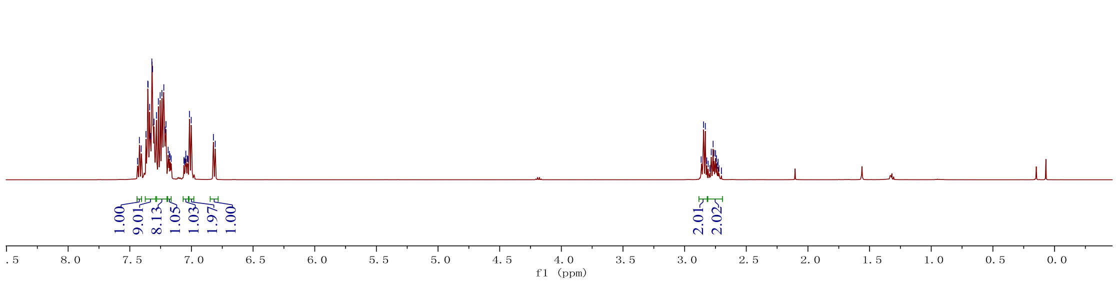

3ad ^{1}H NMR

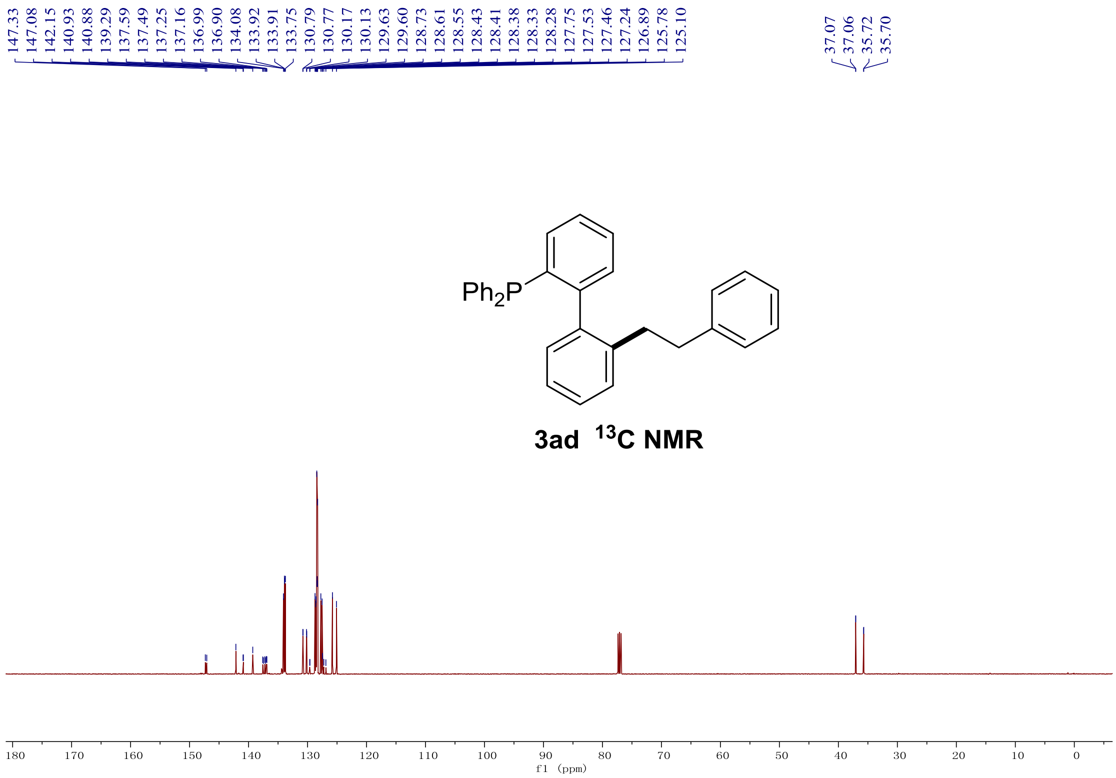

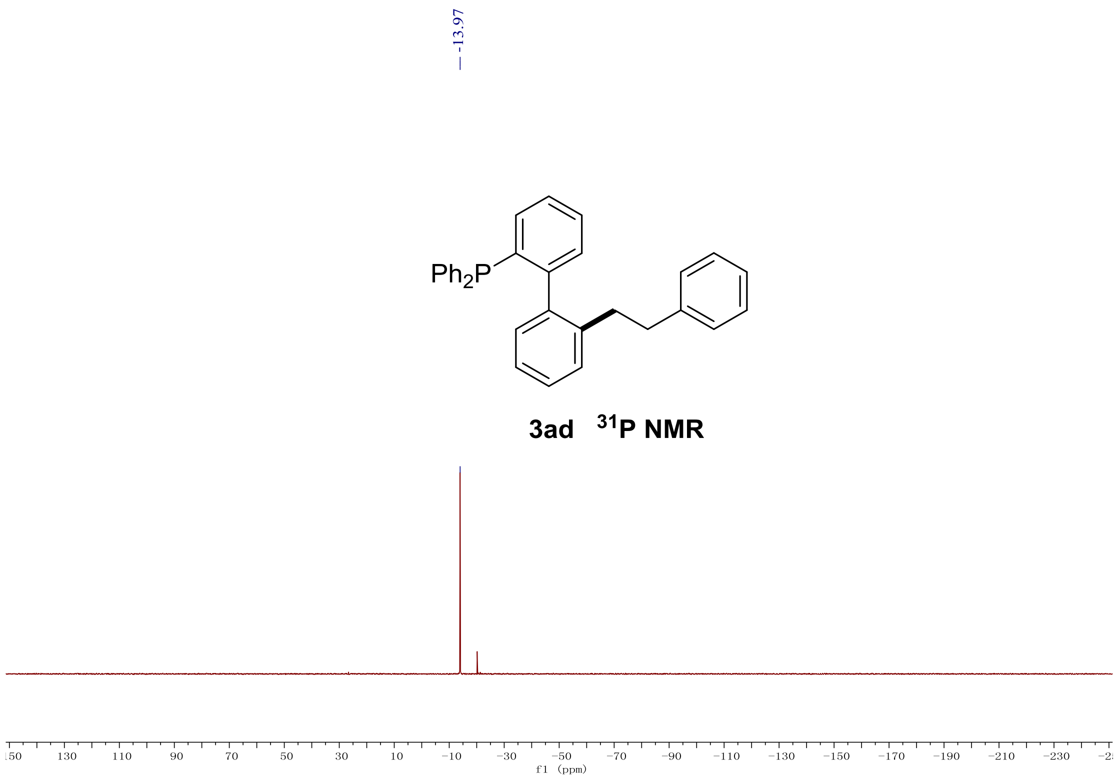

Supplementary Figure 25. ^{1}H, ^{13}C and ^{31}P NMR of compound **3ad.**

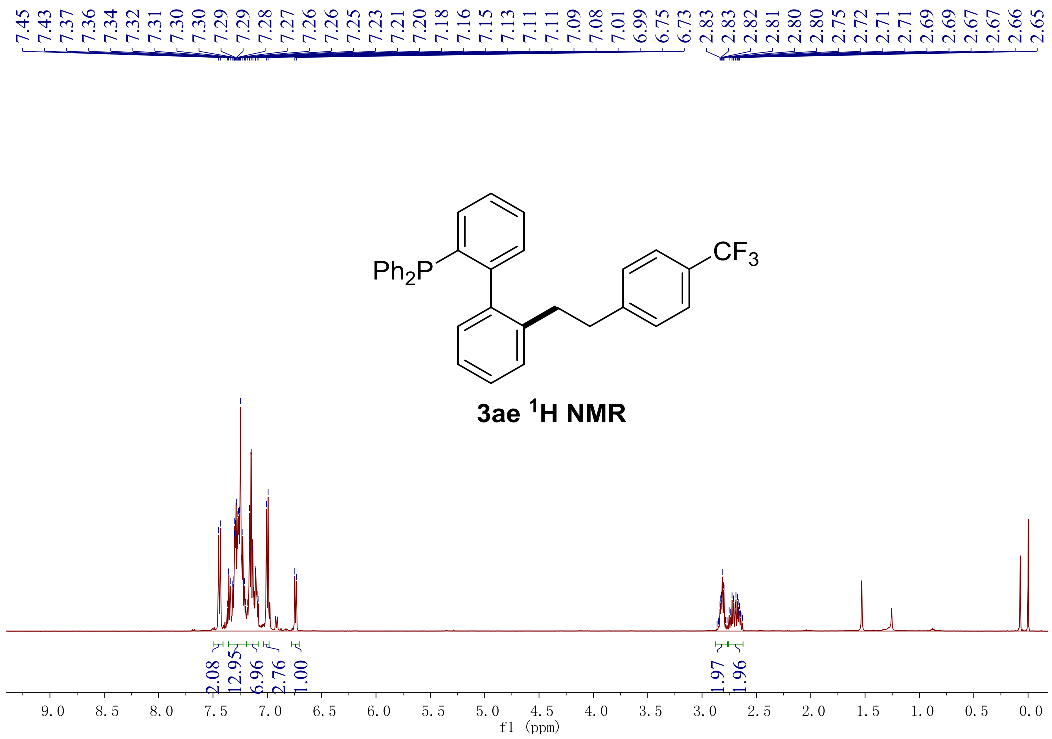
7.45 7.43 7.37 7.36 7.34 7.32 7.31 7.30 7.29 7.29 7.28 7.27 7.26 7.26 7.25 7.23 7.21 7.20 7.18 7.16 7.15 7.13 7.11 7.11 7.09 7.08 7.01 6.99 6.75 6.73 2.83 2.83 2.82 2.81 2.80 2.80 2.75 2.72 2.71 2.71 2.69 2.69 2.67 2.67 2.66 2.65
Ph2P
CF3
3ae 1H NMR
2.08 12.95 6.96 2.76 1.00
1.97 1.96
9.0 8.5 8.0 7.5 7.0 6.5 6.0 5.5 5.0 4.5 4.0 3.5 3.0 2.5 2.0 1.5 1.0 0.5 0.0
f1 (ppm)

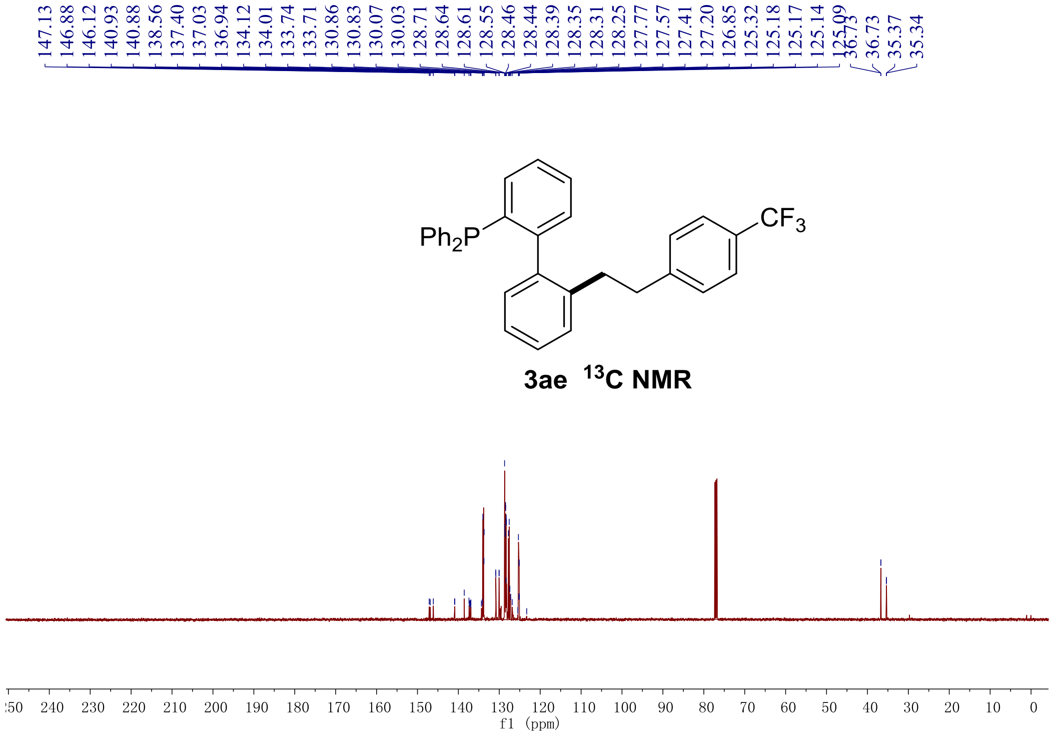
147.13 146.88 146.12 140.93 140.88 138.56 137.40 137.03 136.94 134.12 134.01 133.74 133.71 130.86 130.83 130.07 130.03 128.71 128.64 128.61 128.55 128.46 128.44 128.39 128.35 128.31 128.25 127.77 127.57 127.41 127.20 126.85 125.32 125.18 125.17 125.14 125.09 36.73 36.73 35.37 35.34
Ph2P
CF3
3ae 13C NMR
250 240 230 220 210 200 190 180 170 160 150 140 130 120 110 100 90 80 70 60 50 40 30 20 10 0
f1 (ppm)

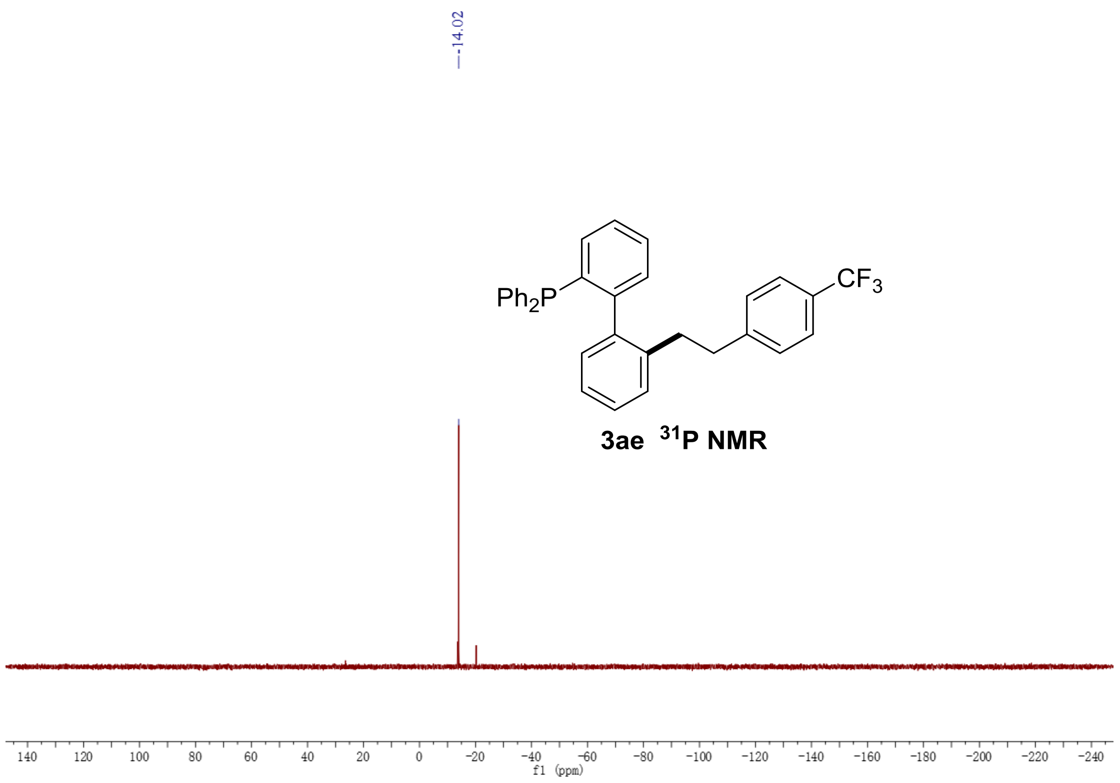

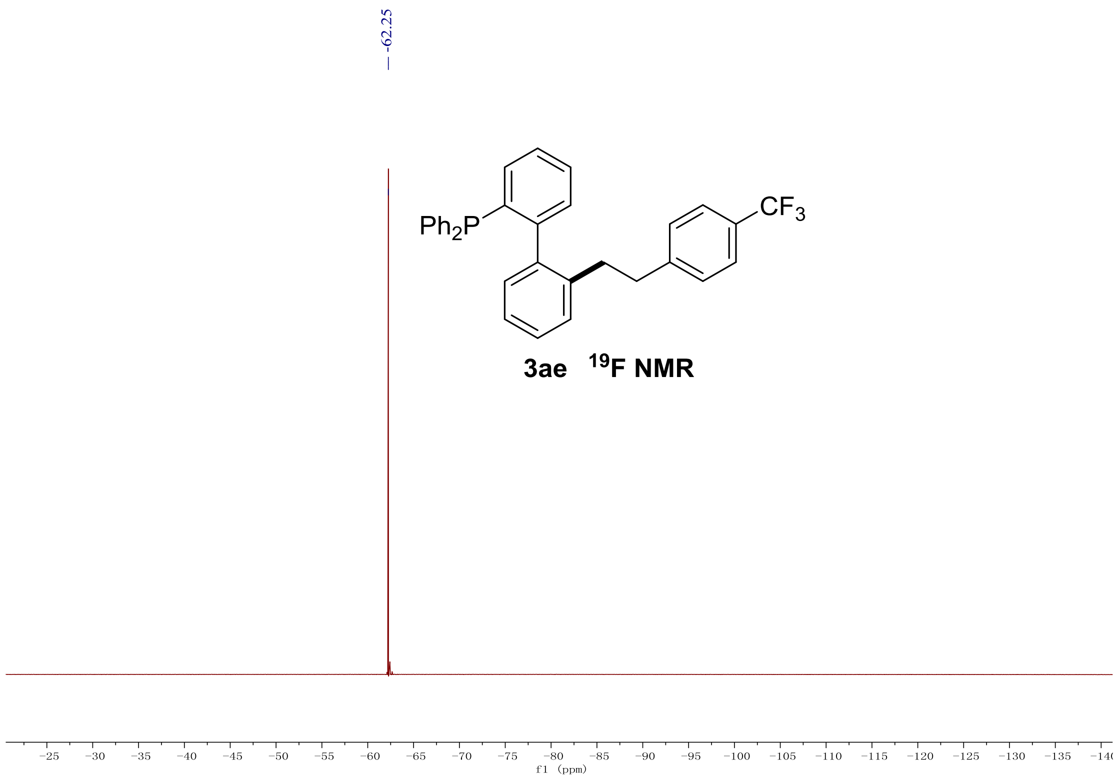

Supplementary Figure 26. ^{1}H, ^{13}C and ^{31}P NMR of compound **3ae.**

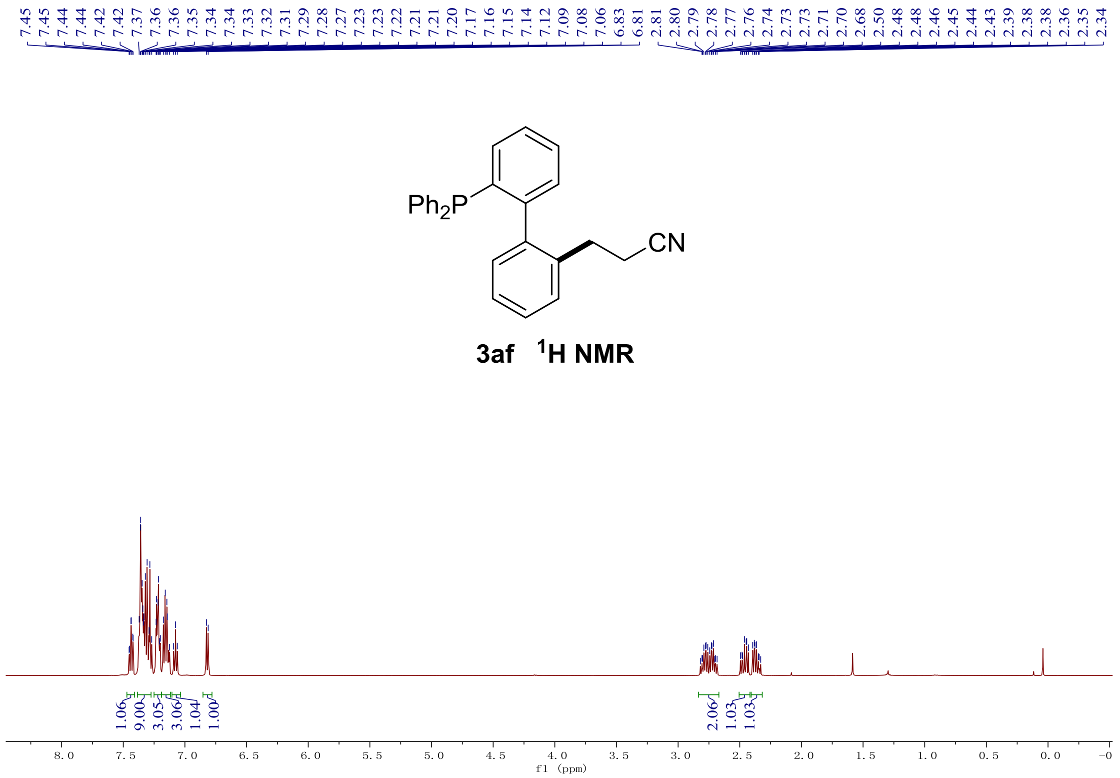
7.45 7.45 7.44 7.44 7.42 7.42 7.37 7.36 7.36 7.35 7.34 7.34 7.33 7.32 7.31 7.29 7.28 7.27 7.23 7.23 7.22 7.21 7.21 7.20 7.17 7.16 7.15 7.14 7.12 7.09 7.08 7.06 6.83 6.81 2.81 2.80 2.79 2.78 2.77 2.76 2.74 2.73 2.73 2.71 2.70 2.68 2.50 2.48 2.48 2.46 2.45 2.44 2.43 2.39 2.38 2.38 2.36 2.35 2.34
Ph2P
CN
3af 1H NMR
1.06 9.00 3.05 3.06 1.04 1.00
2.06 1.03 1.03
8.0 7.5 7.0 6.5 6.0 5.5 5.0 4.5 4.0 3.5 3.0 2.5 2.0 1.5 1.0 0.5 0.0 -0
f1 (ppm)

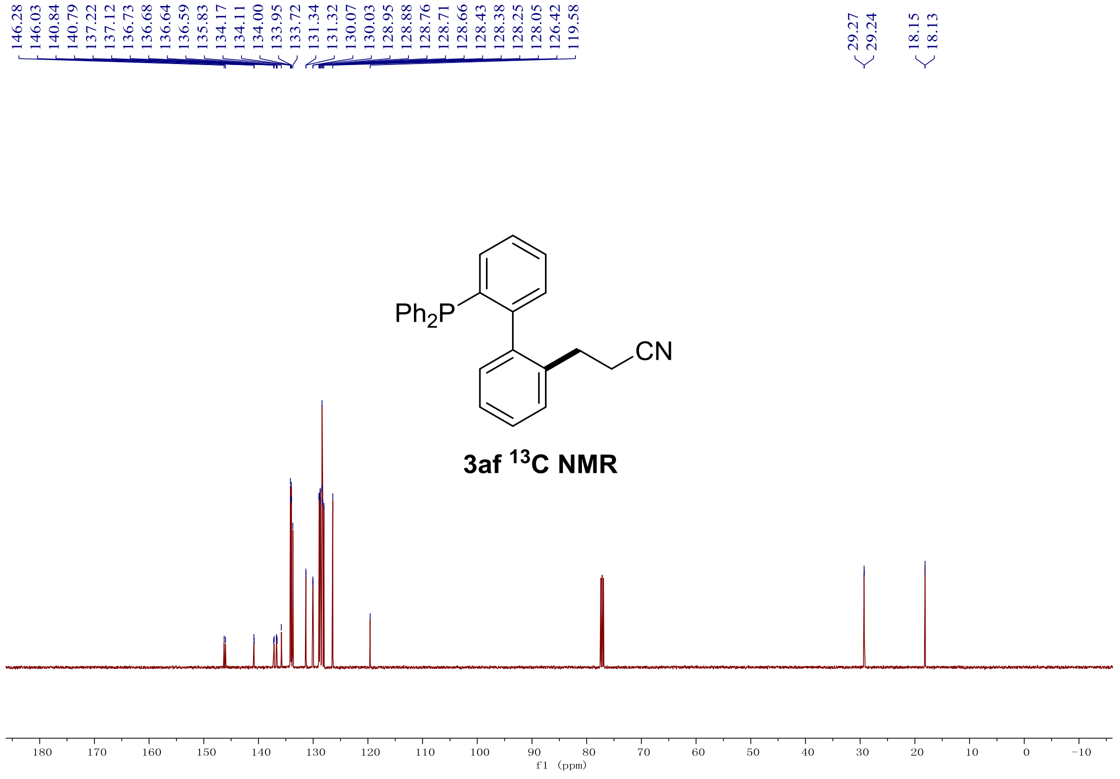
146.28 146.03 140.84 140.79 137.22 137.12 136.73 136.68 136.64 136.59 135.83 134.17 134.11 134.00 133.95 133.72 131.34 131.32 130.07 130.03 128.95 128.88 128.76 128.71 128.66 128.43 128.38 128.25 128.05 126.42 119.58
29.27 29.24 18.15 18.13
Ph2P
CN
3af 13C NMR
180 170 160 150 140 130 120 110 100 90 80 70 60 50 40 30 20 10 0 -10
f1 (ppm)

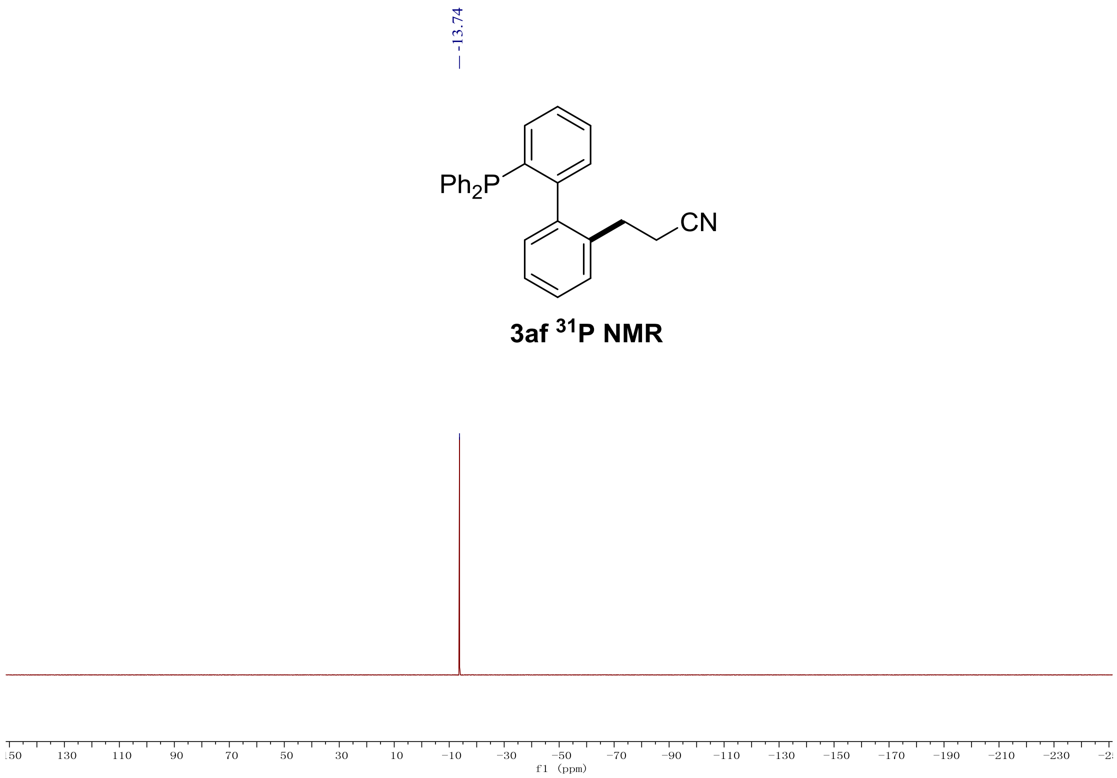

Supplementary Figure 27. ^{1}H, ^{13}C and ^{31}P NMR of compound **3af.**

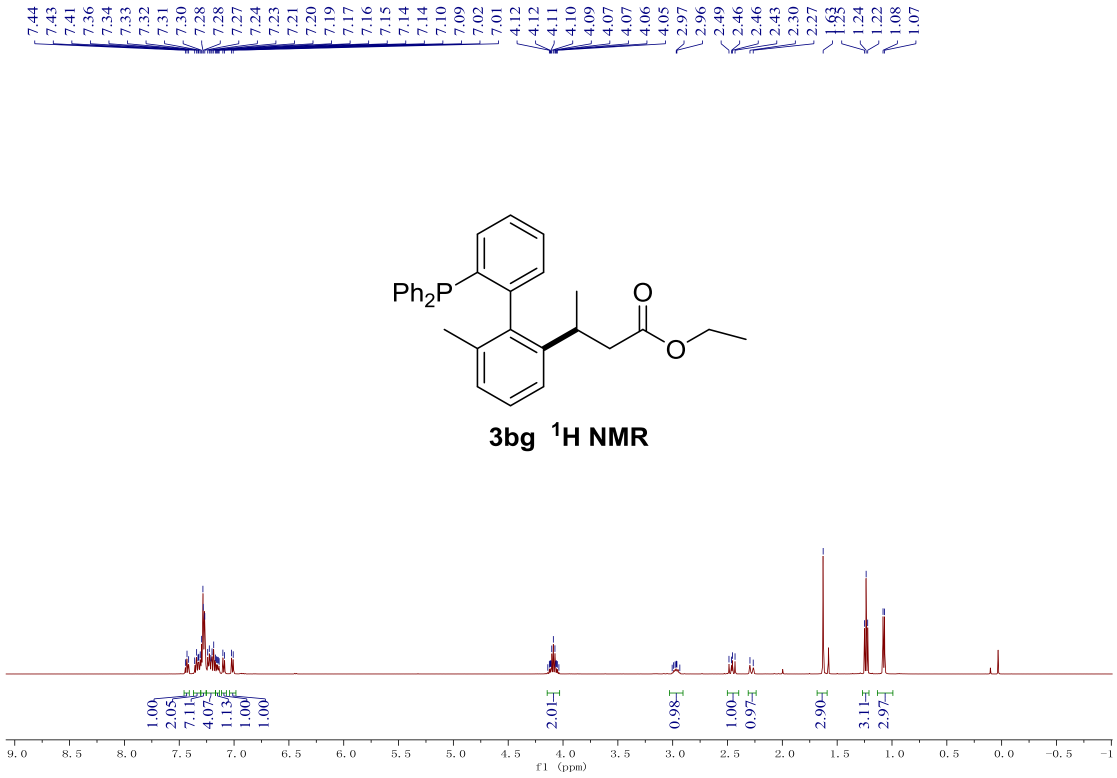

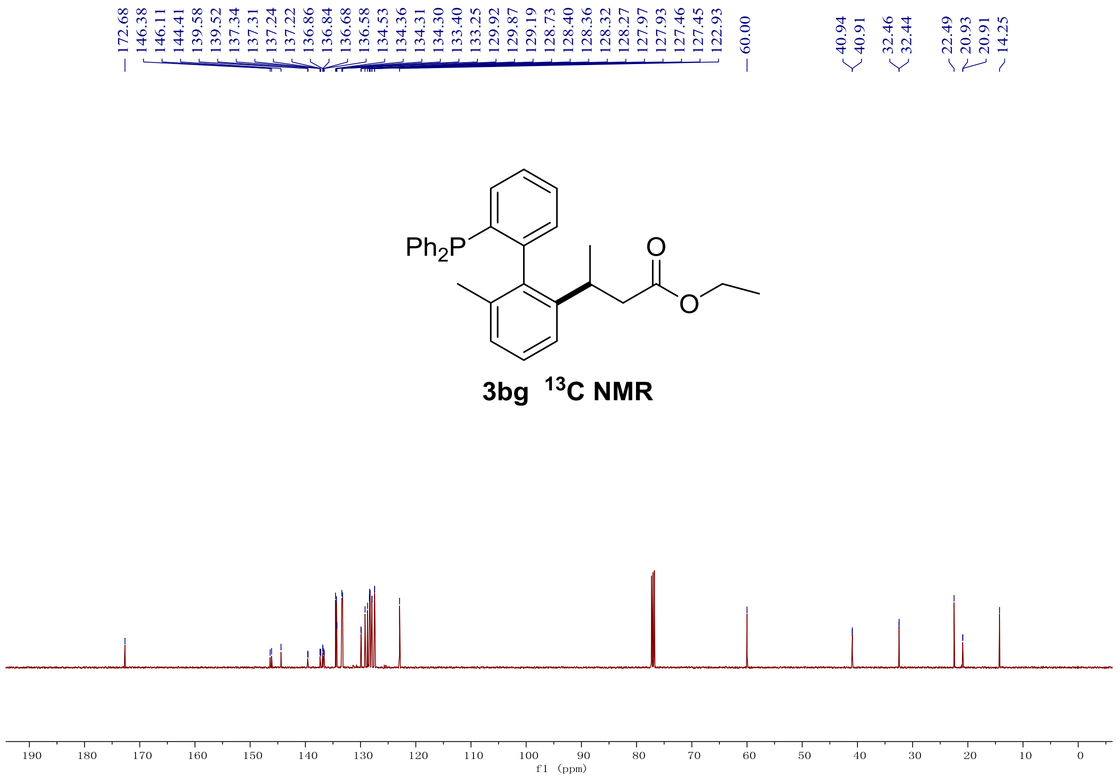

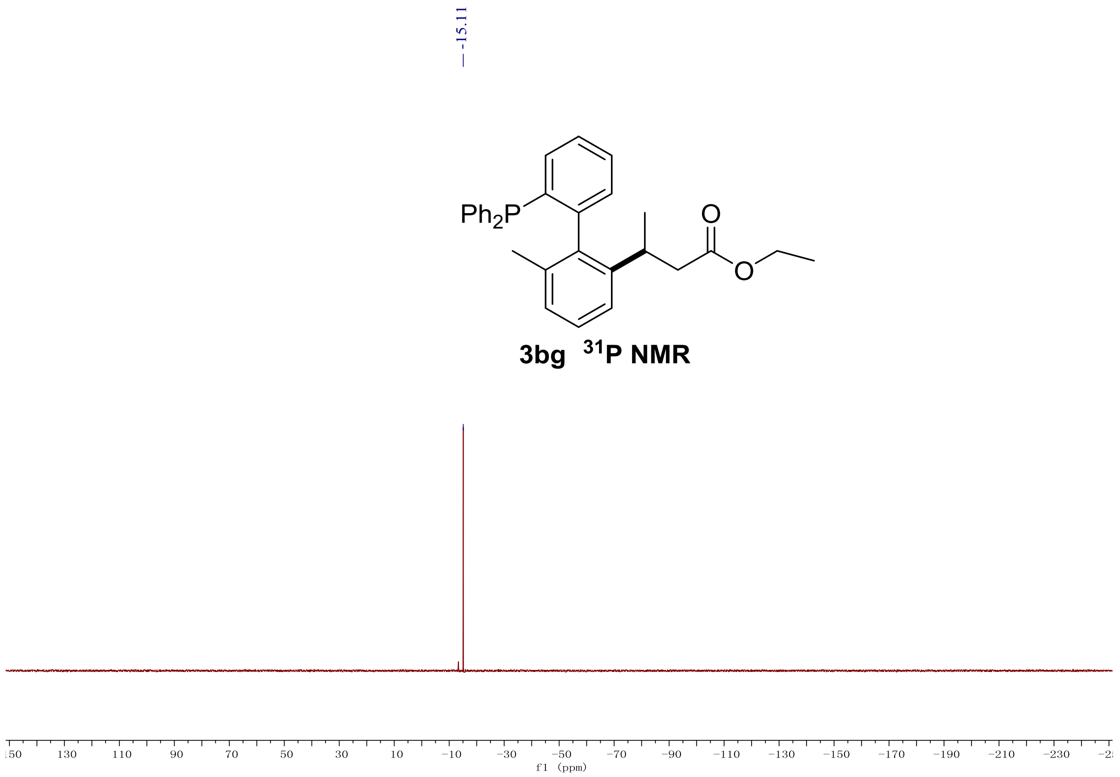

Supplementary Figure 28. ^{1}H, ^{13}C and ^{31}P NMR of compound **3bg.**

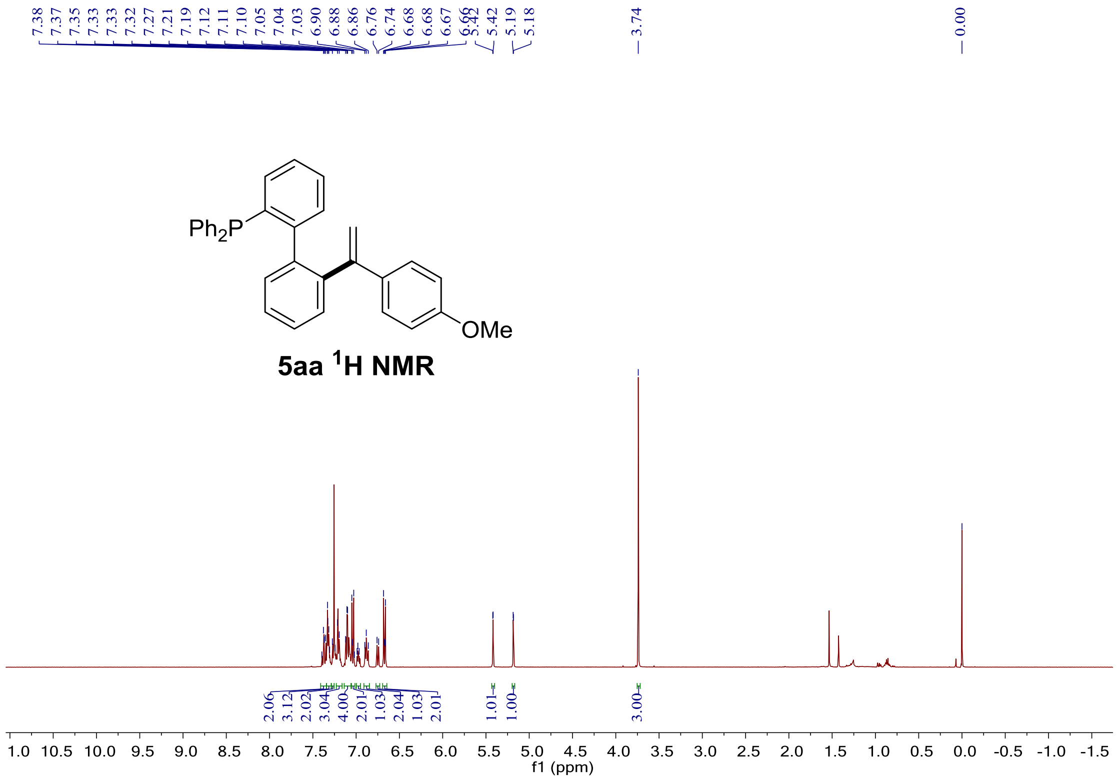

Ph2P
OMe
5aa 1H NMR
f1 (ppm)

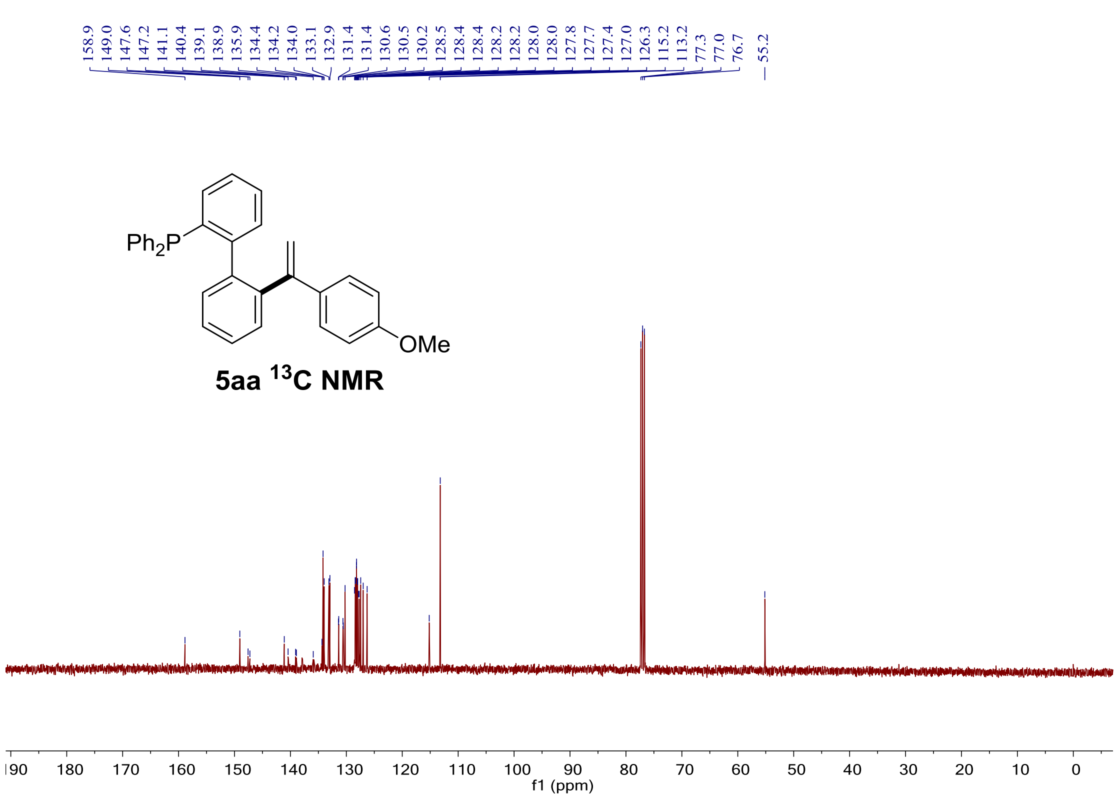

Ph2P
OMe
5aa 13C NMR
f1 (ppm)

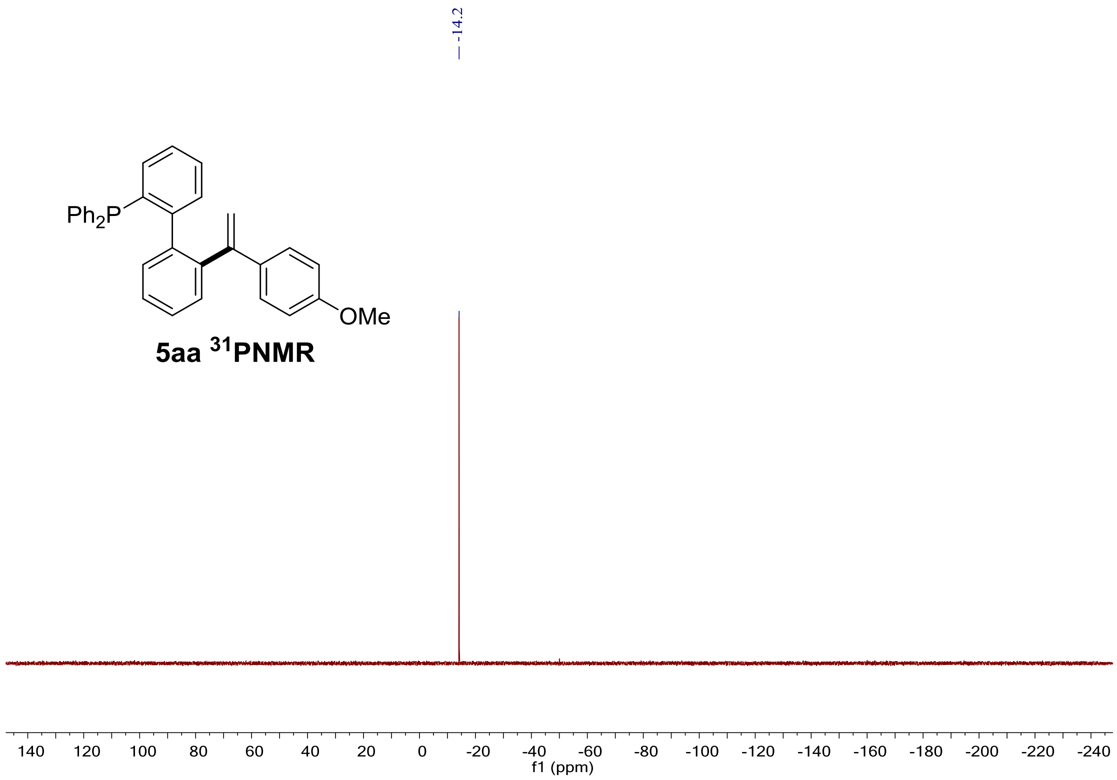

Supplementary Figure 29. ^{1}H, ^{13}C and ^{31}P NMR of compound **5aa.**

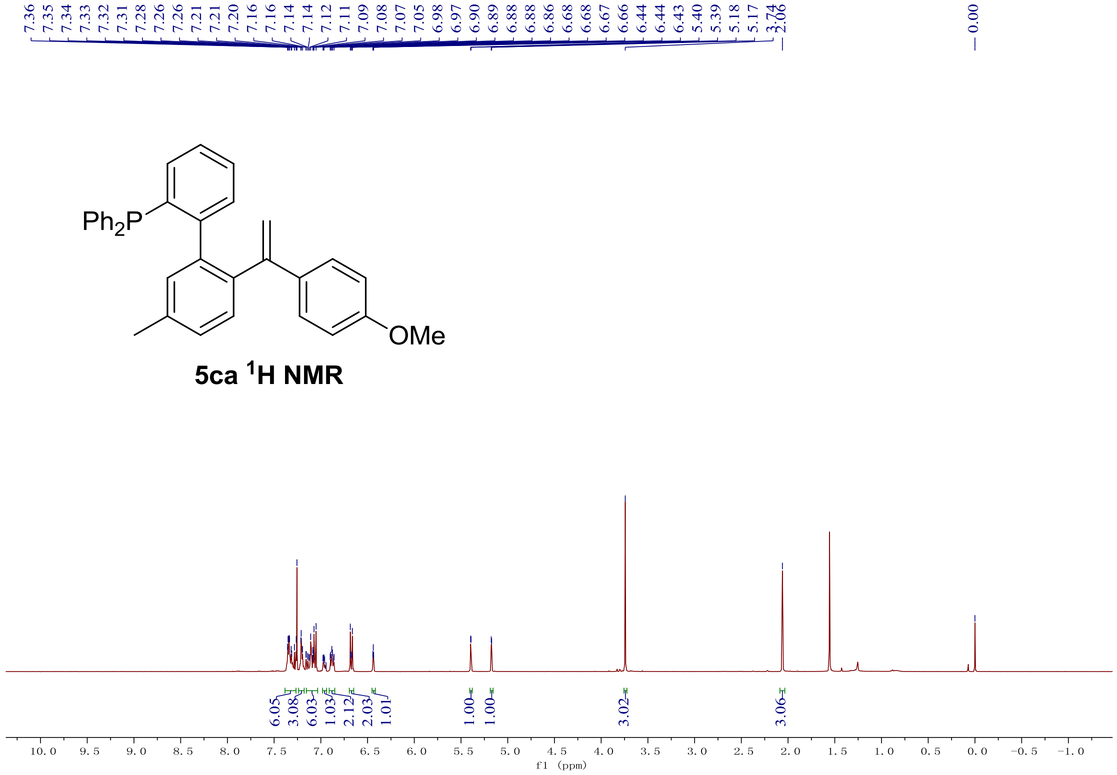

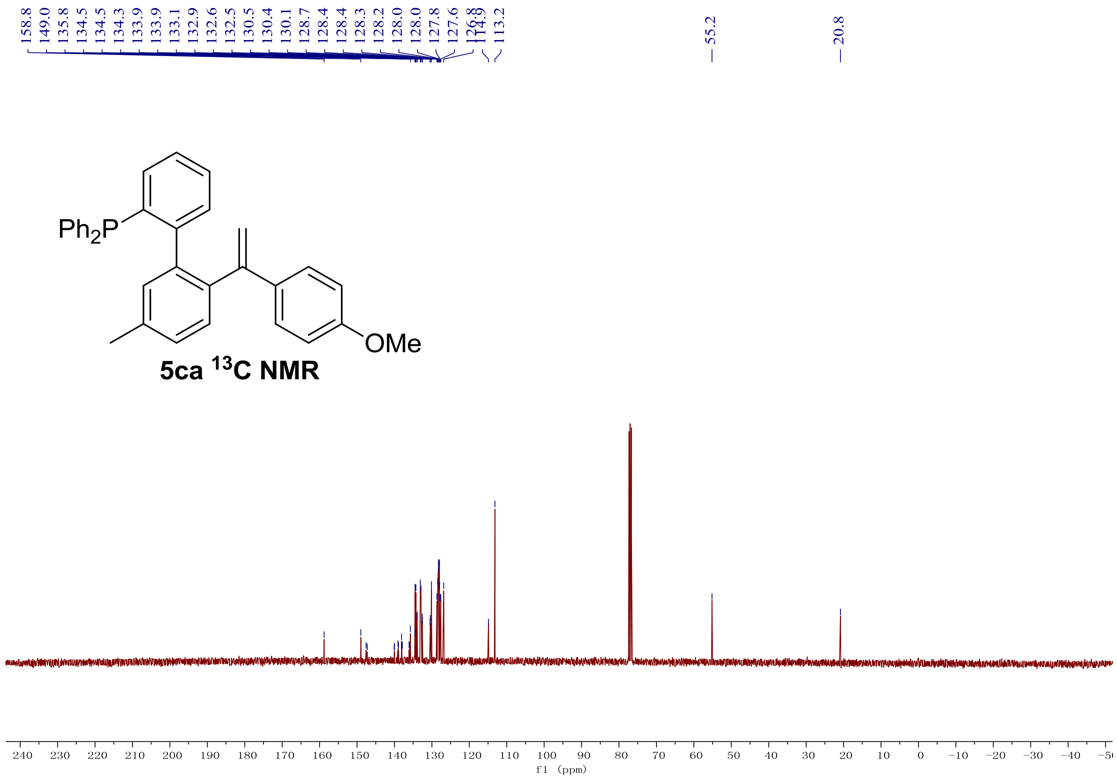

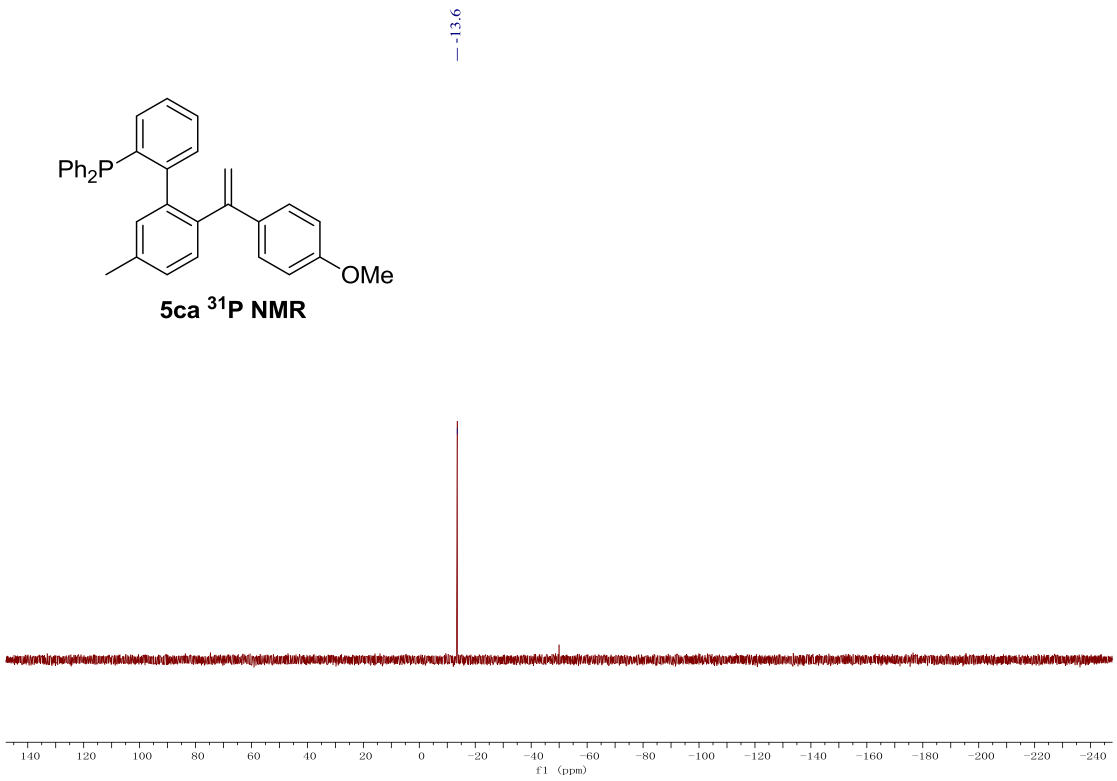

Supplementary Figure 30. ^{1}H, ^{13}C and ^{31}P NMR of compound **5ca.**

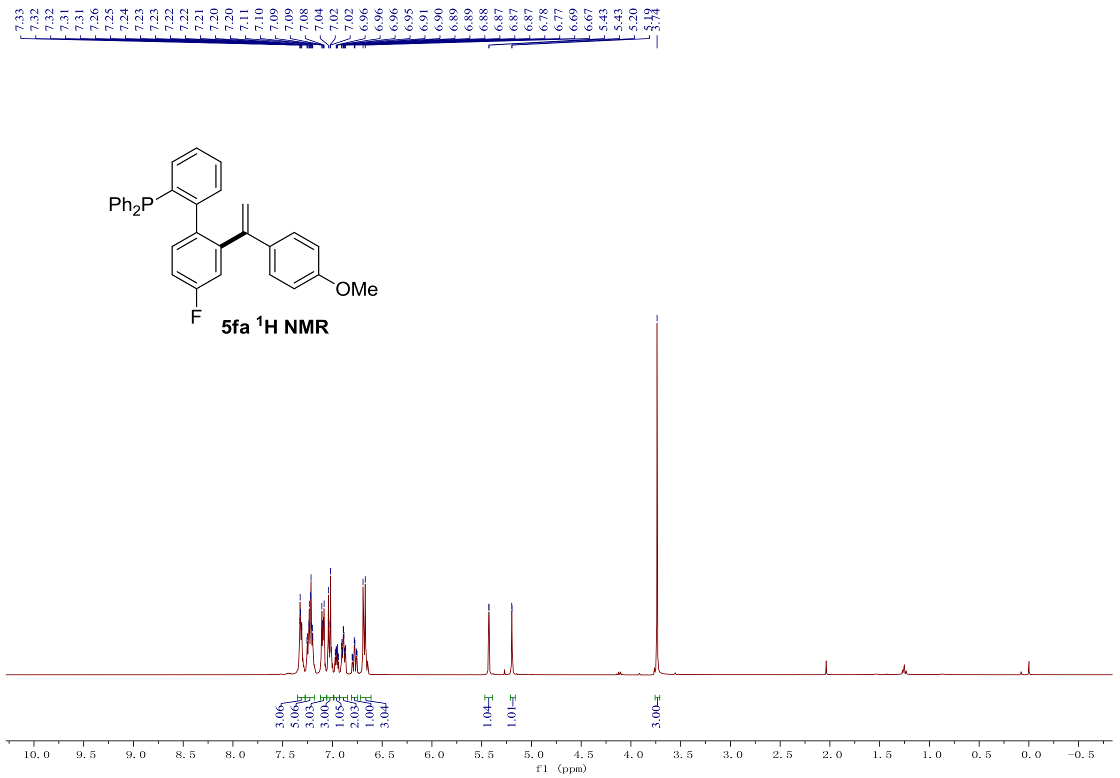

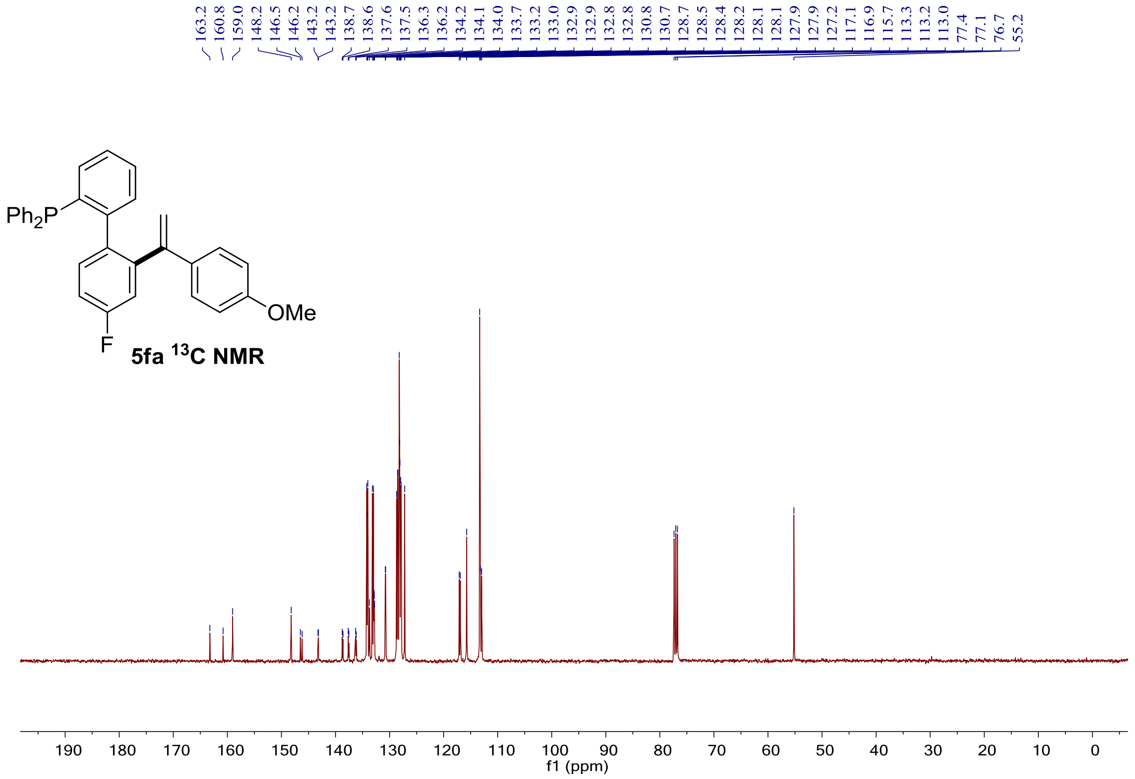

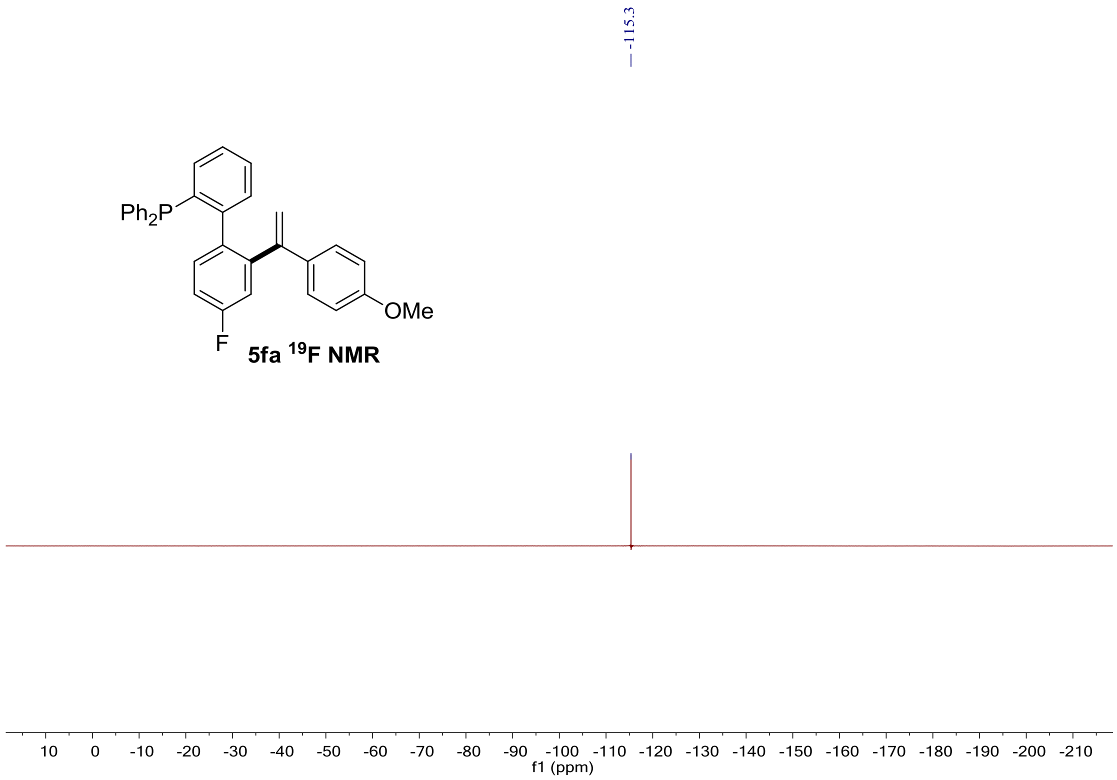

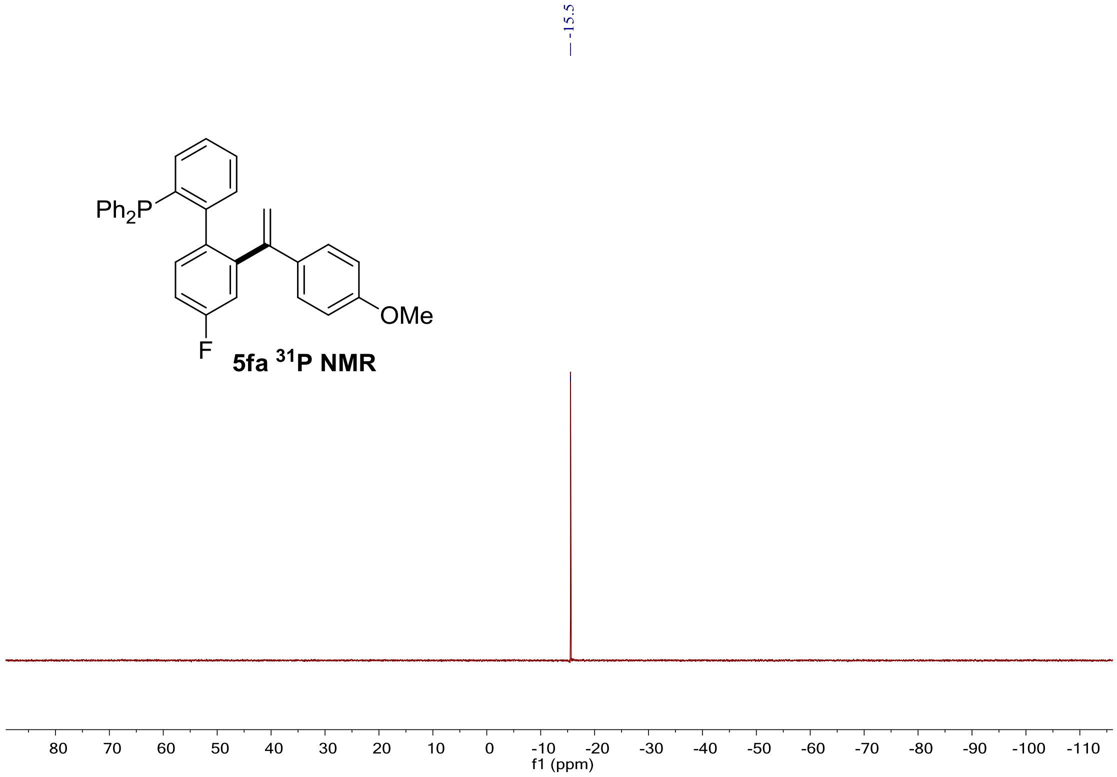

Supplementary Figure 31. ^{1}H, ^{13}C, ^{31}P and ^{19}F NMR of compound **5fa.**

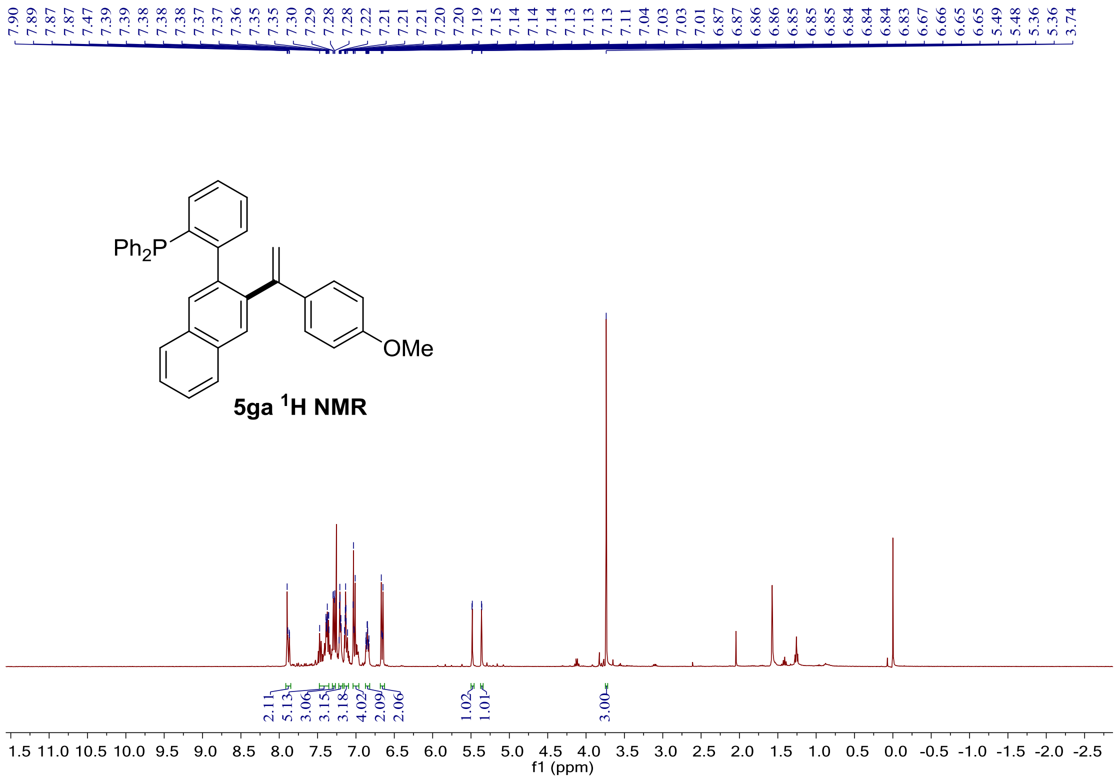
7.90 7.89 7.87 7.87 7.47 7.39 7.39 7.38 7.38 7.38 7.37 7.37 7.36 7.35 7.35 7.30 7.29 7.28 7.28 7.22 7.21 7.21 7.21 7.20 7.20 7.19 7.15 7.14 7.14 7.14 7.13 7.13 7.13 7.11 7.04 7.03 7.03 7.01 6.87 6.87 6.86 6.85 6.85 6.85 6.84 6.84 6.84 6.83 6.67 6.66 6.66 6.65 6.65 5.49 5.48 5.36 5.36 3.74
Ph2P
OMe
5ga 1H NMR
2.11 5.13 3.06 3.15 3.18 4.02 2.09 2.06 1.02 1.01 3.00
f1 (ppm)

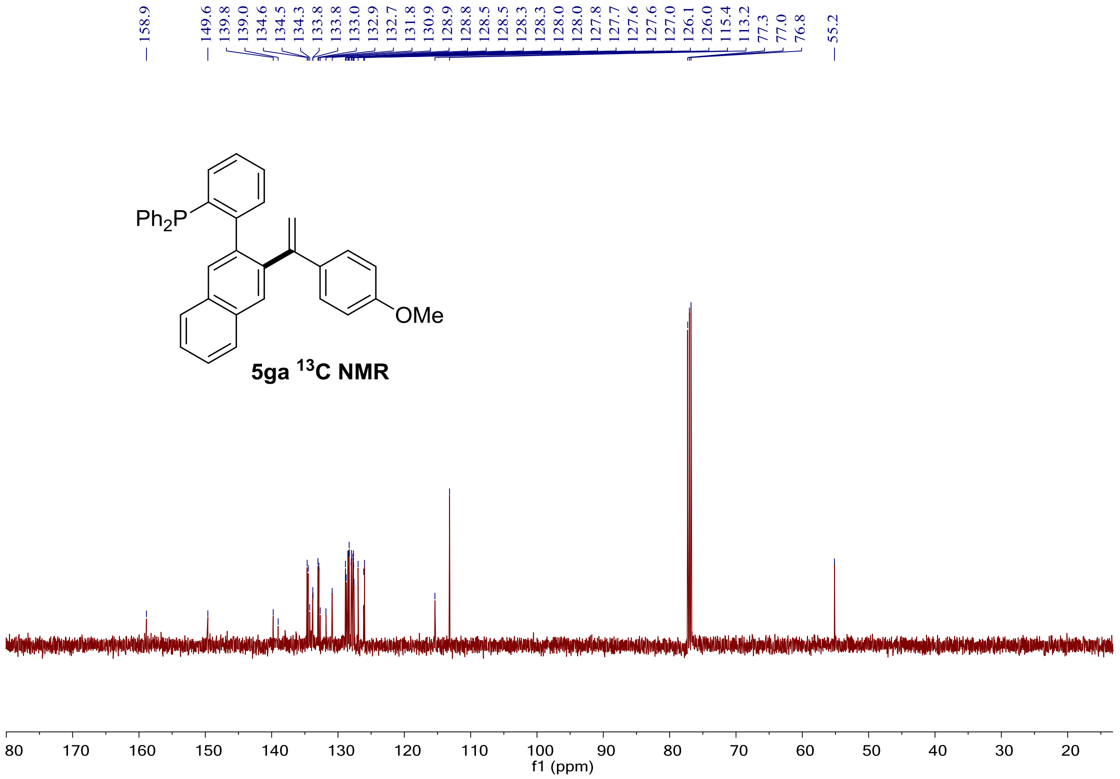
158.9 149.6 139.8 139.0 134.6 134.5 134.3 133.8 133.8 133.0 132.9 132.7 131.8 130.9 128.9 128.8 128.5 128.5 128.3 128.3 128.0 128.0 127.8 127.7 127.6 127.6 127.0 126.1 126.0 115.4 113.2 77.3 77.0 76.8 55.2
Ph2P
OMe
5ga 13C NMR
f1 (ppm)

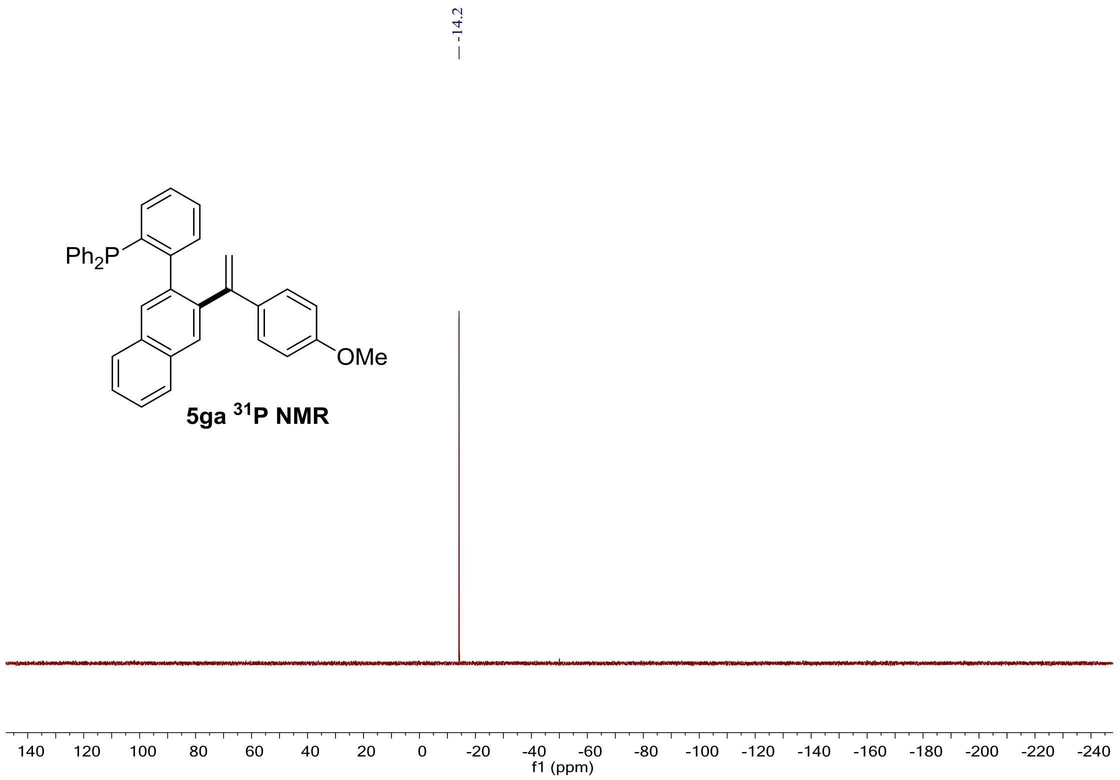

Supplementary Figure 32. ^{1}H, ^{13}C and ^{31}P NMR of compound **5ga.**

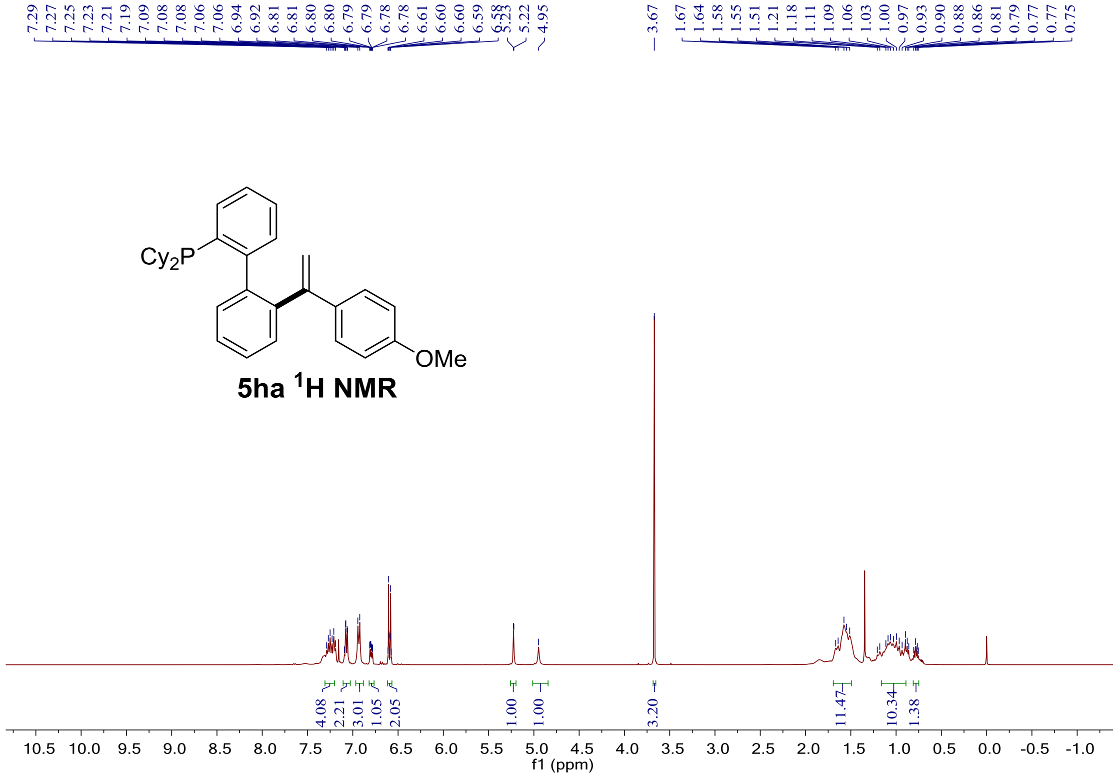

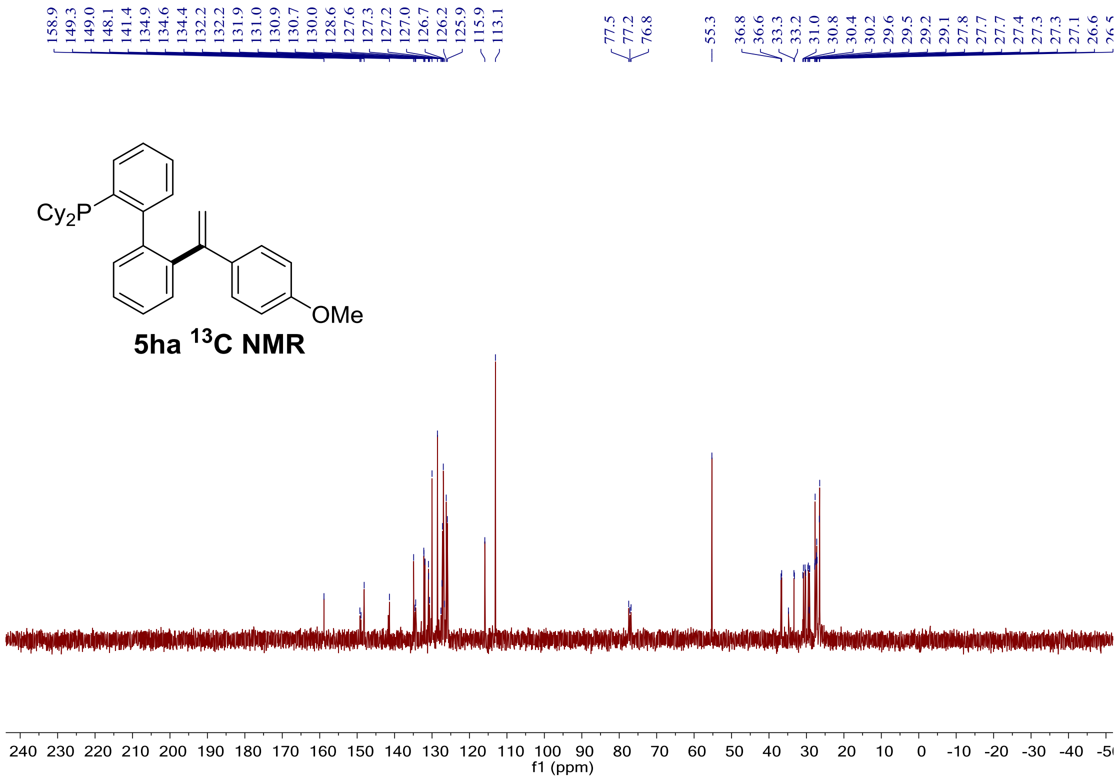

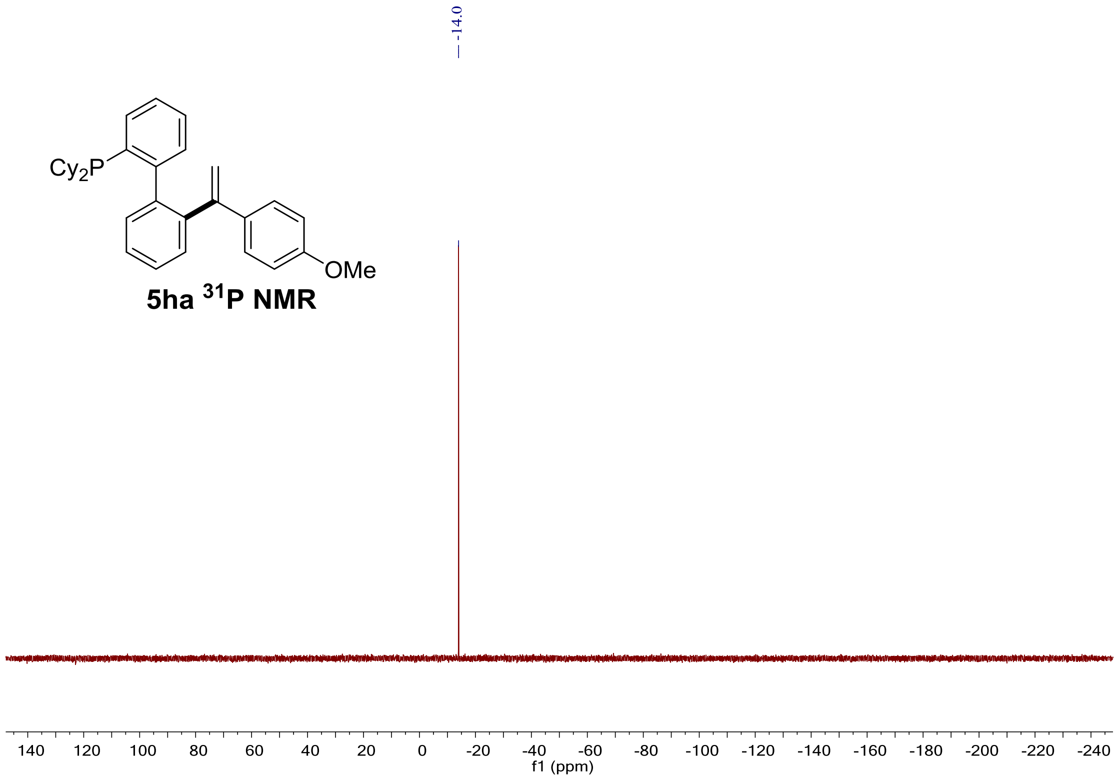

Supplementary Figure 33. ^{1}H, ^{13}C and ^{31}P NMR of compound **5ha.**

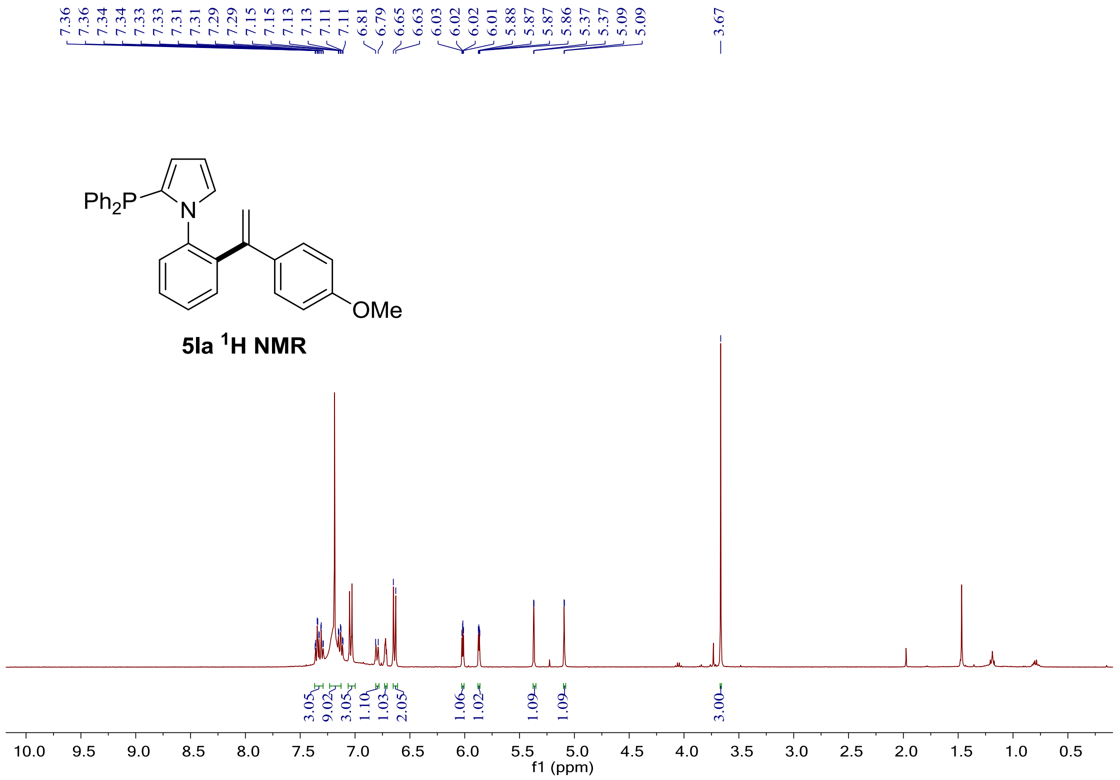
7.36 7.36 7.34 7.34 7.33 7.33 7.31 7.31 7.29 7.29 7.15 7.15 7.13 7.13 7.11 7.11 6.81 6.79 6.65 6.63 6.03 6.02 6.02 6.01 5.88 5.87 5.87 5.86 5.37 5.37 5.09 5.09
3.67
Ph2P
N
OMe
5la 1H NMR
3.05 9.02 3.05 1.10 1.03 2.05 1.06 1.02 1.09 1.09 3.00
10.0 9.5 9.0 8.5 8.0 7.5 7.0 6.5 6.0 5.5 5.0 4.5 4.0 3.5 3.0 2.5 2.0 1.5 1.0 0.5
f1 (ppm)

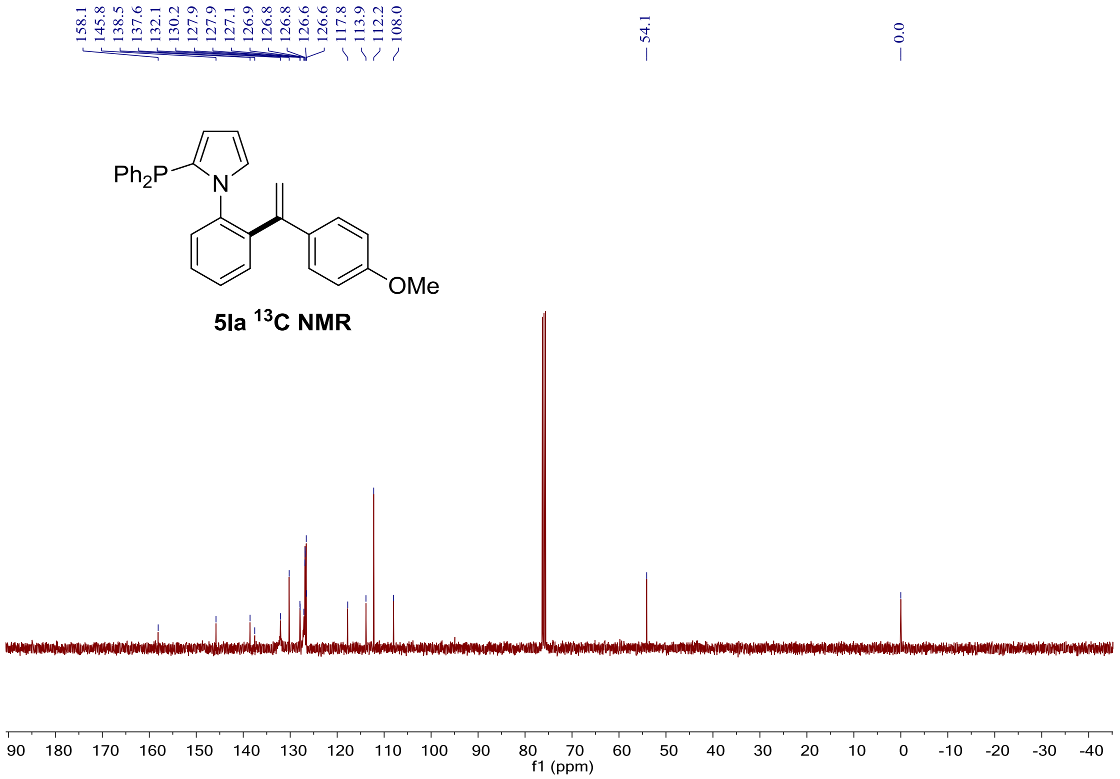
158.1 145.8 138.5 137.6 132.1 130.2 127.9 127.9 127.1 126.9 126.8 126.8 126.6 126.6 117.8 113.9 112.2 108.0
54.1
0.0
Ph2P
N
OMe
5la 13C NMR
90 180 170 160 150 140 130 120 110 100 90 80 70 60 50 40 30 20 10 0 -10 -20 -30 -40
f1 (ppm)

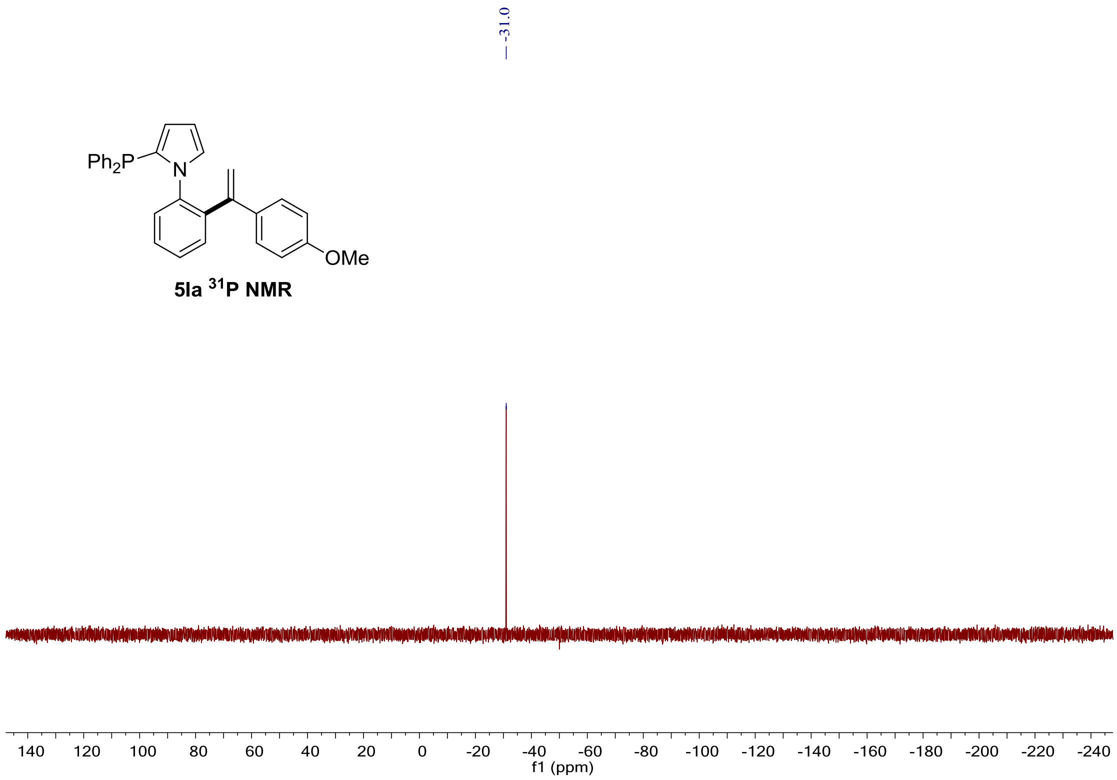

Supplementary Figure 34. ^{1}H, ^{13}C and ^{31}P NMR of compound **5la.**

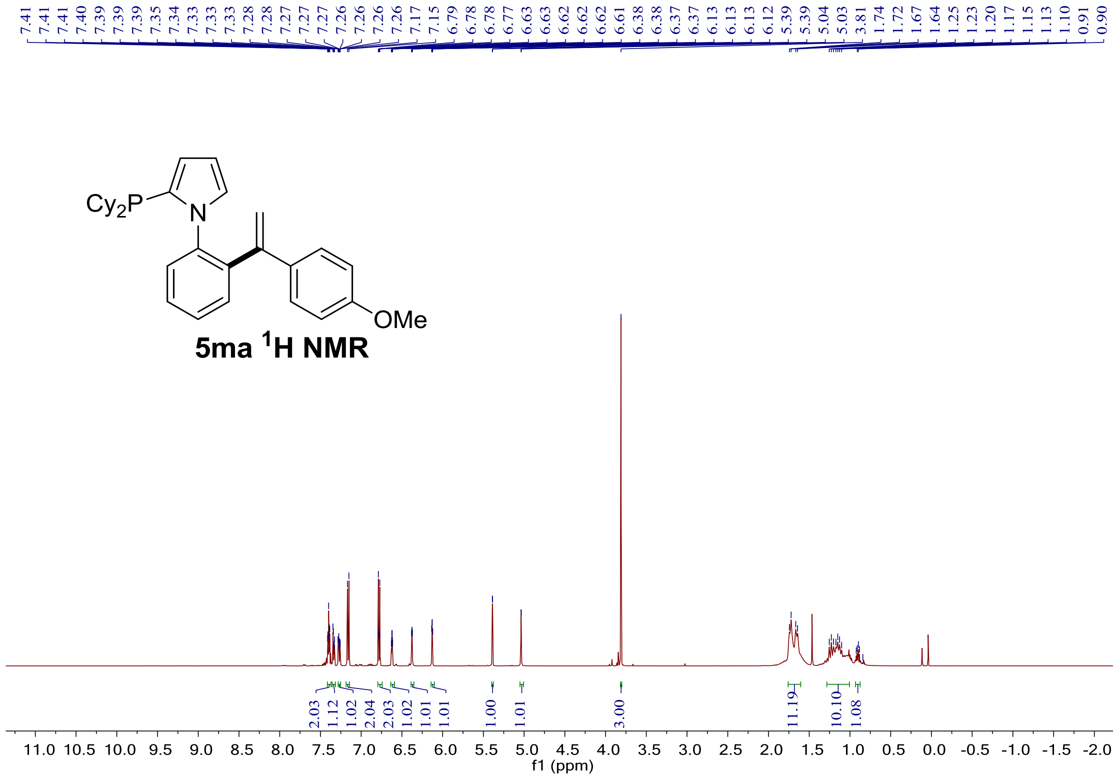

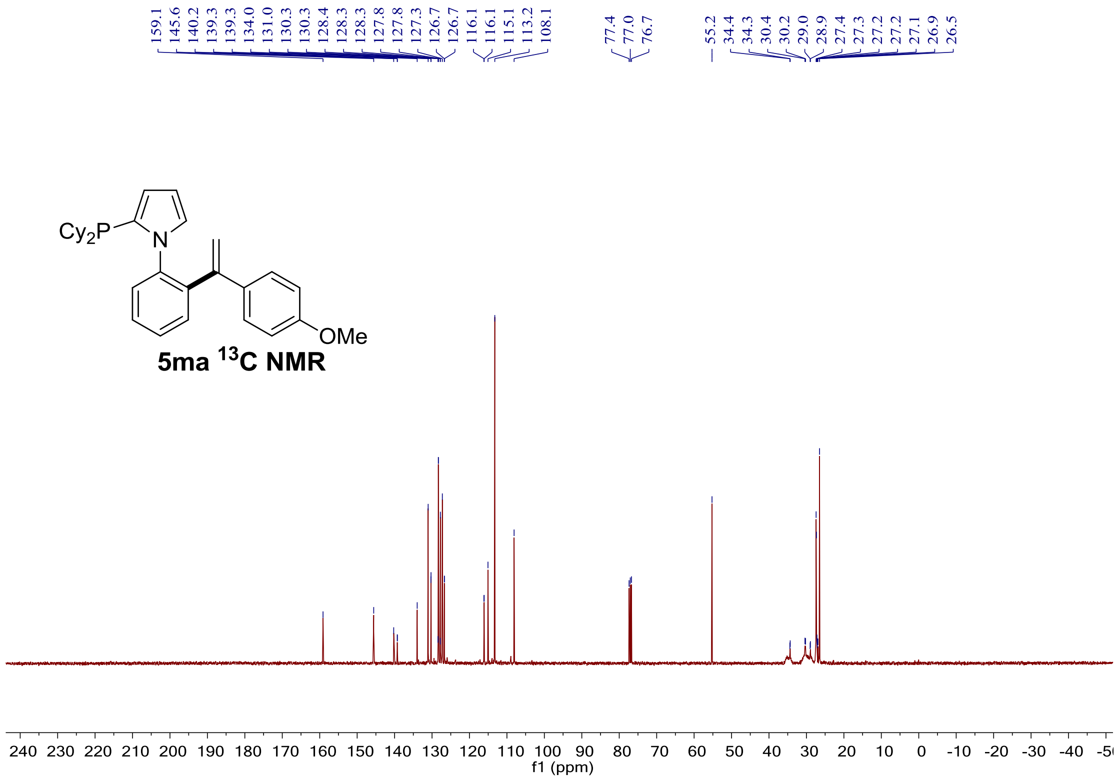

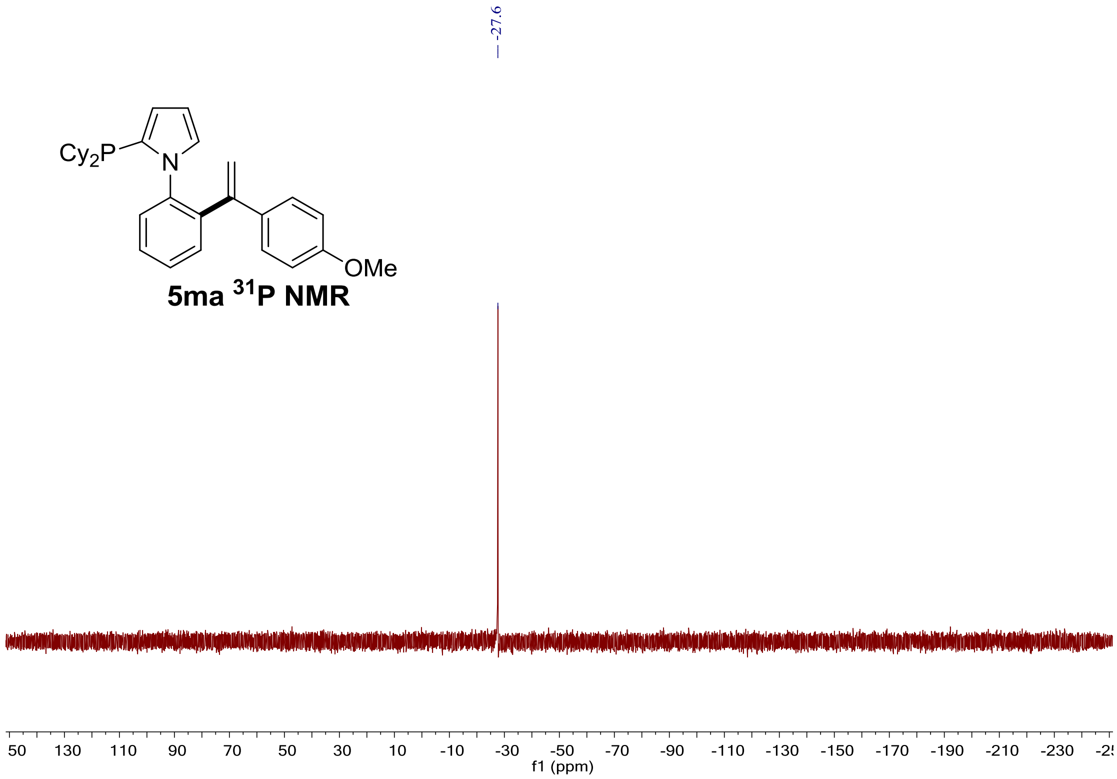

Supplementary Figure 35. ^{1}H, ^{13}C and ^{31}P NMR of compound **5ma.**

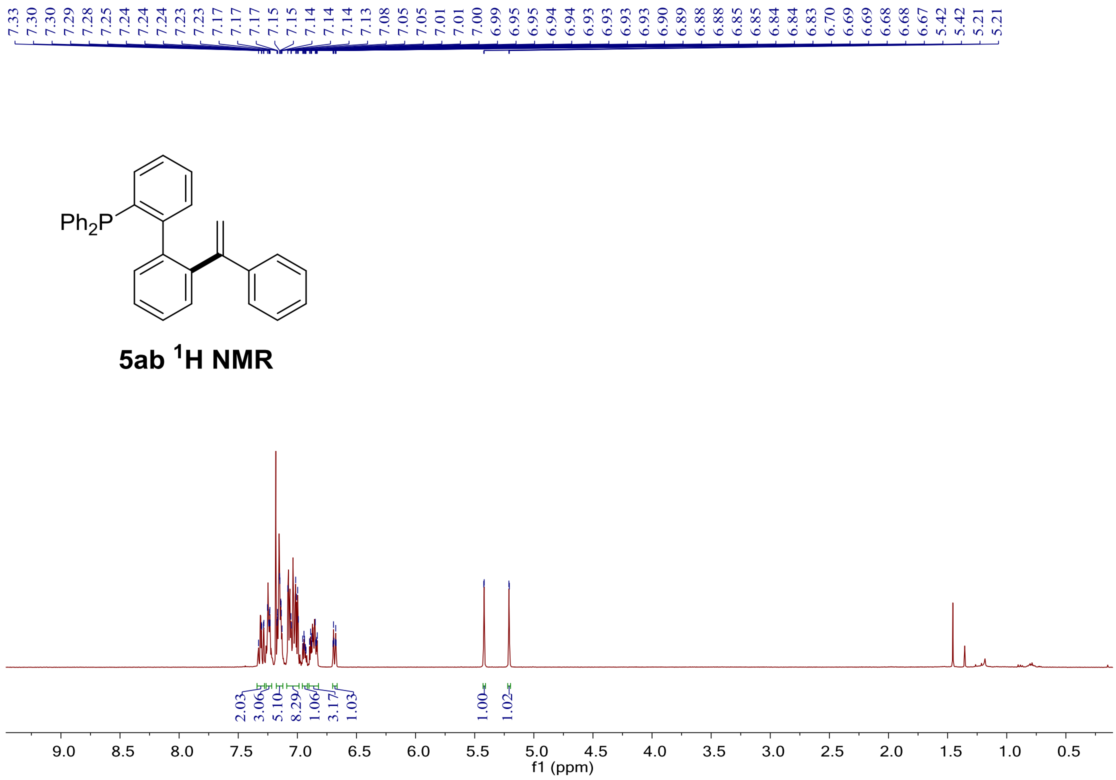

5ab ^{1}H NMR

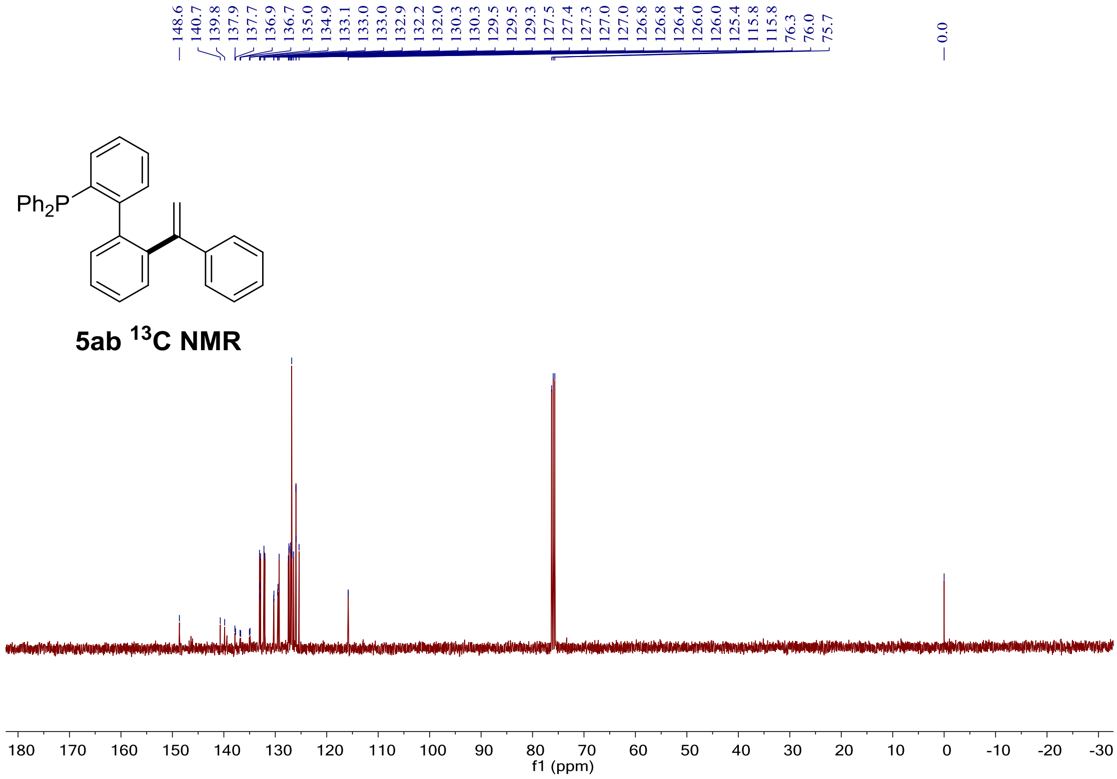

5ab ^{13}C NMR

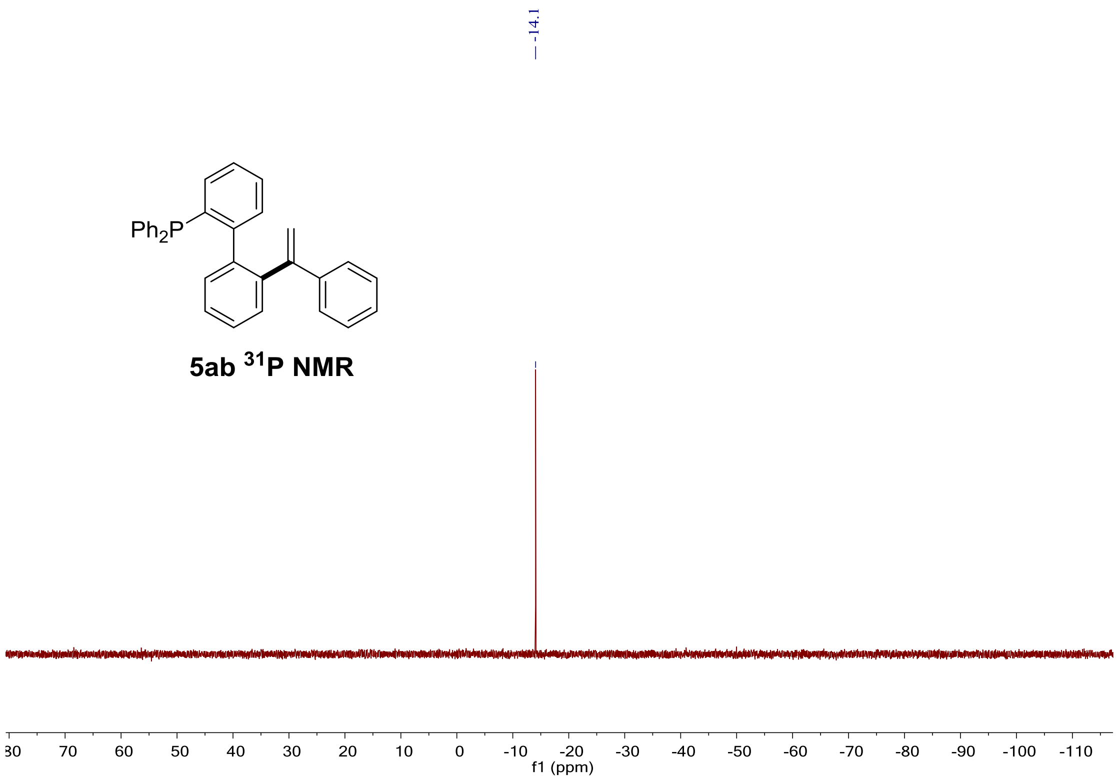

Supplementary Figure 36. ^{1}H, ^{13}C and ^{31}P NMR of compound **5ab.**

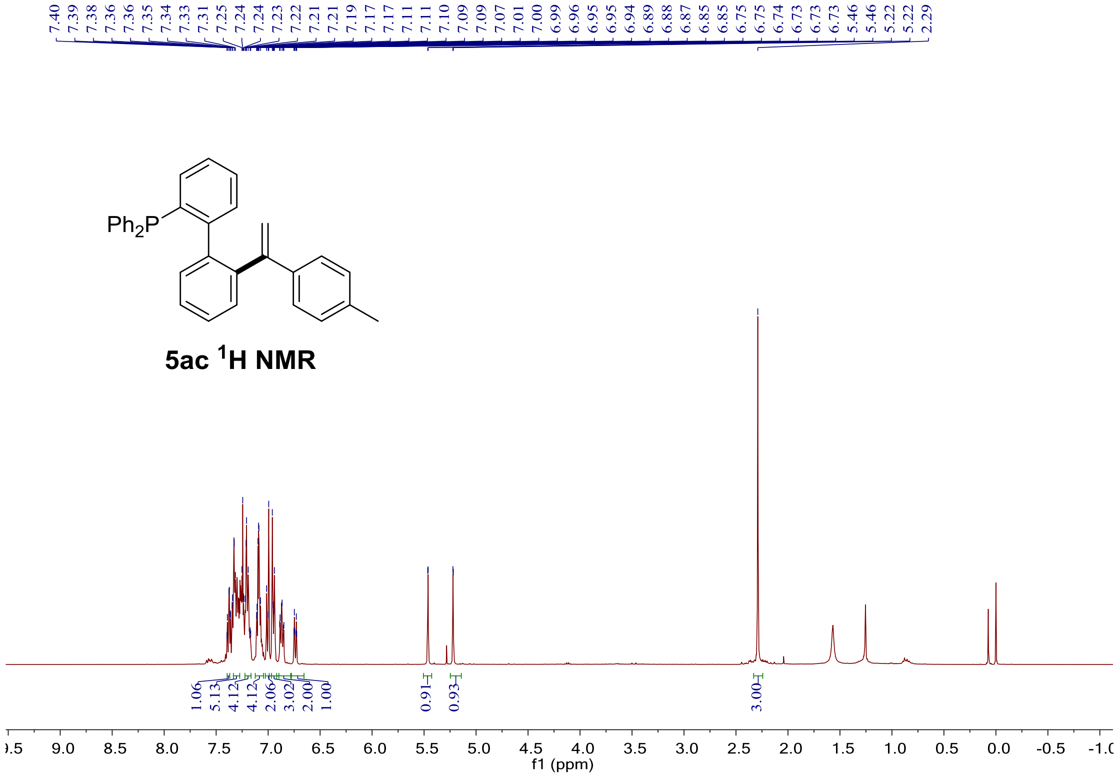

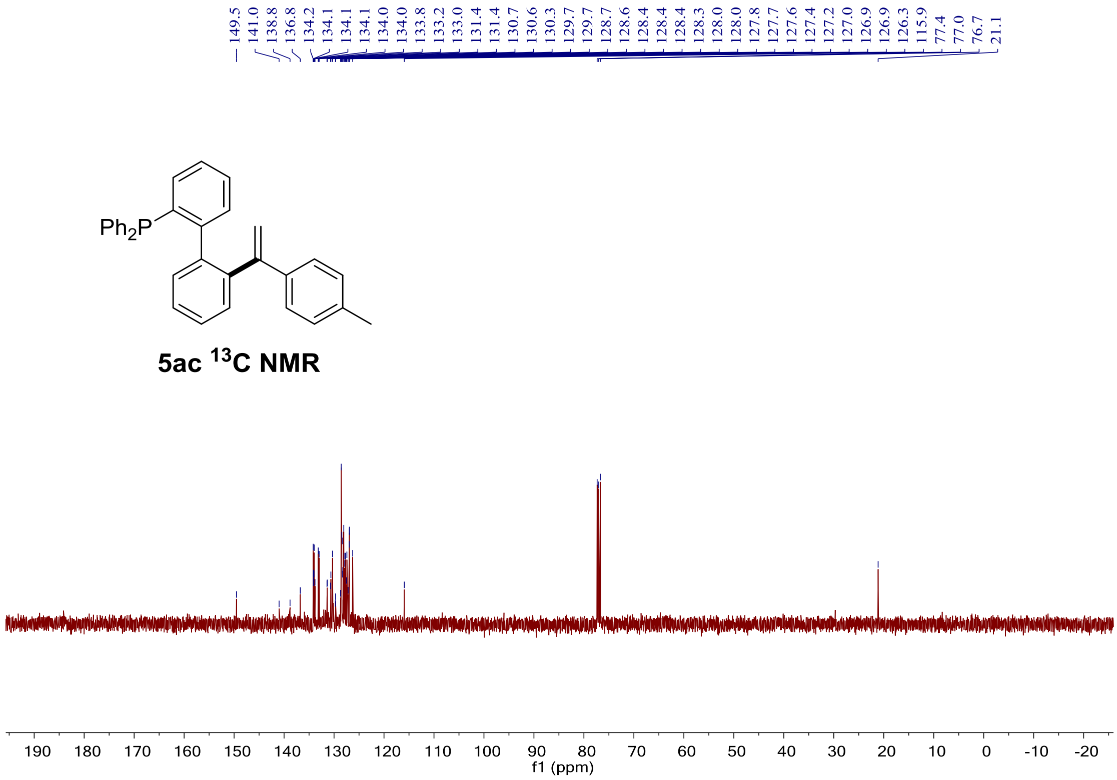

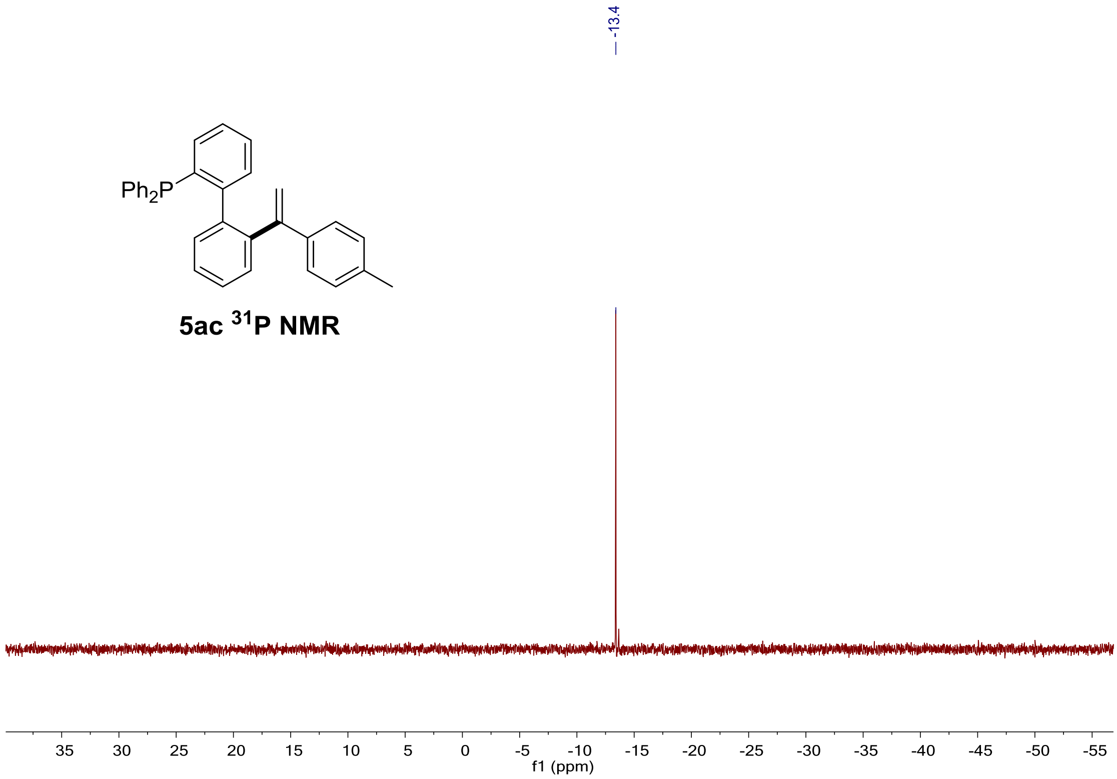

Supplementary Figure 37. ^{1}H, ^{13}C and ^{31}P NMR of compound **5ac.**

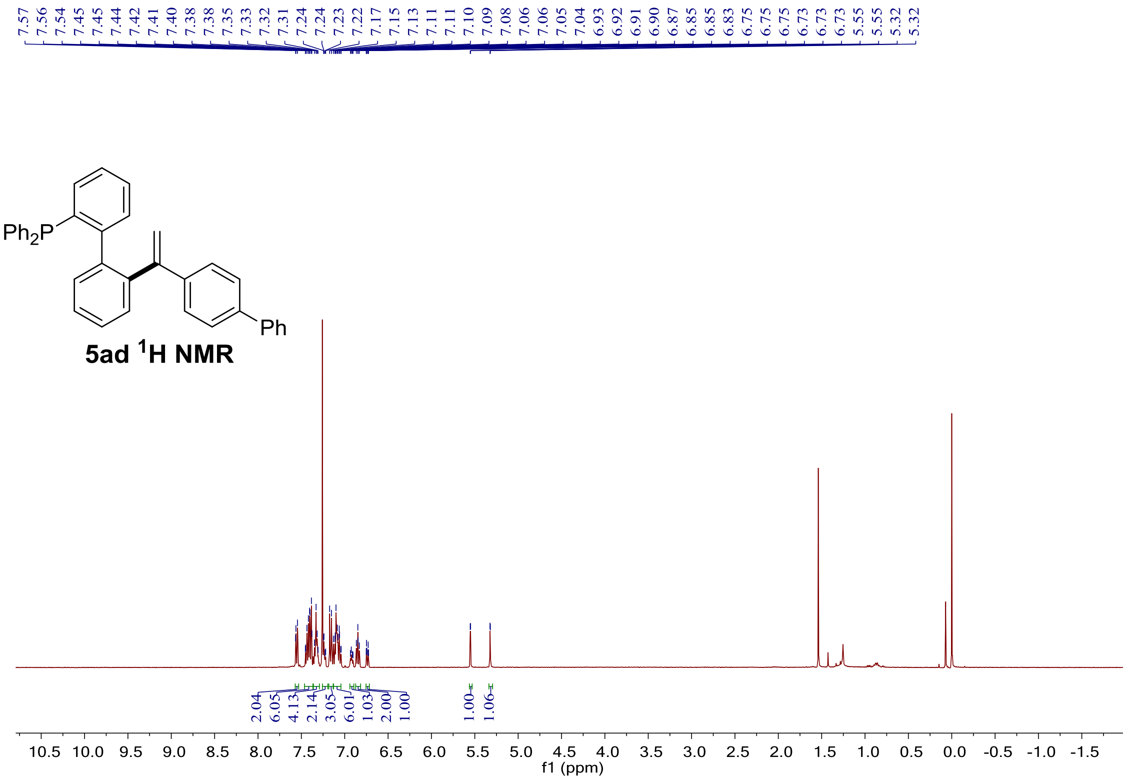
7.57 7.56 7.54 7.45 7.45 7.44 7.42 7.41 7.40 7.38 7.38 7.35 7.33 7.32 7.31 7.24 7.24 7.23 7.22 7.17 7.15 7.13 7.11 7.11 7.10 7.09 7.08 7.06 7.06 7.05 7.04 6.93 6.92 6.91 6.90 6.87 6.85 6.85 6.83 6.75 6.75 6.73 6.73 6.73 5.55 5.55 5.32 5.32
Ph2P
Ph
5ad 1H NMR
2.04 6.05 4.13 2.14 3.05 6.01 1.03 2.00 1.00 1.00 1.06
10.5 10.0 9.5 9.0 8.5 8.0 7.5 7.0 6.5 6.0 5.5 5.0 4.5 4.0 3.5 3.0 2.5 2.0 1.5 1.0 0.5 0.0 -0.5 -1.0 -1.5
f1 (ppm)

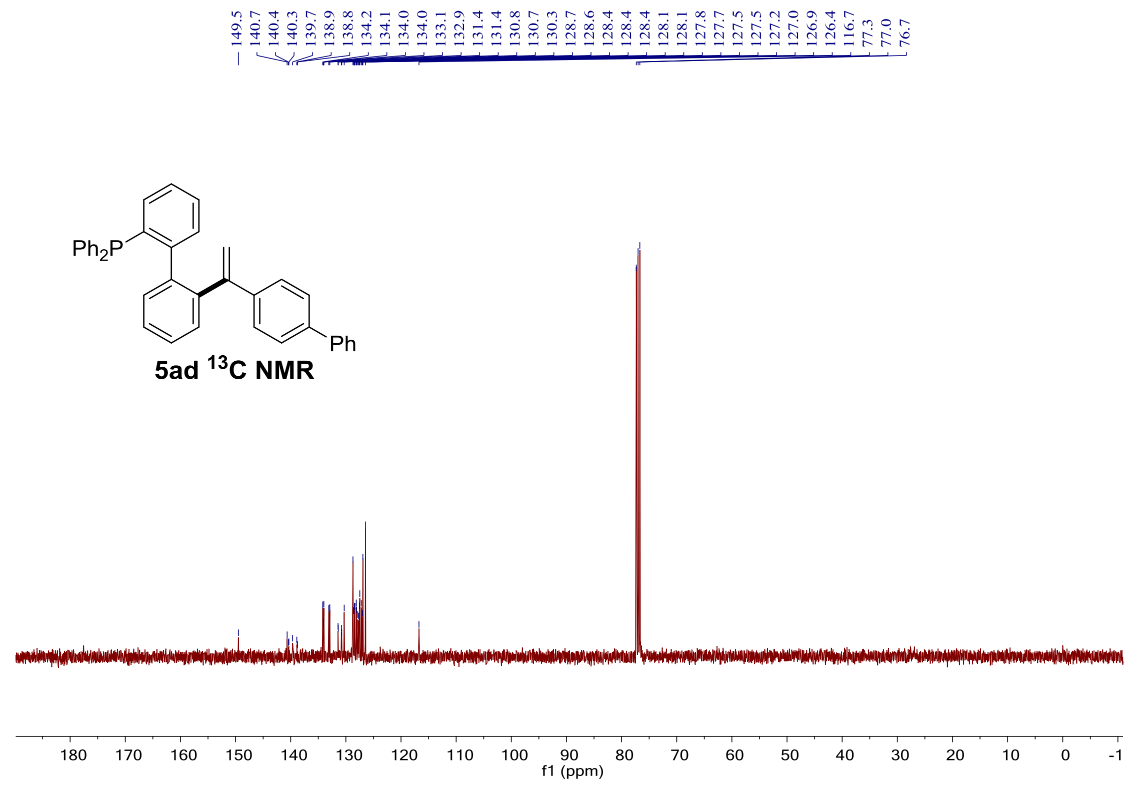
149.5 140.7 140.4 140.3 139.7 138.9 138.8 134.2 134.1 134.0 134.0 133.1 132.9 131.4 131.4 130.8 130.7 130.3 128.7 128.6 128.4 128.4 128.4 128.1 128.1 127.8 127.7 127.5 127.5 127.2 127.0 126.9 126.4 116.7 77.3 77.0 76.7
Ph2P
Ph
5ad 13C NMR
180 170 160 150 140 130 120 110 100 90 80 70 60 50 40 30 20 10 0 -1
f1 (ppm)

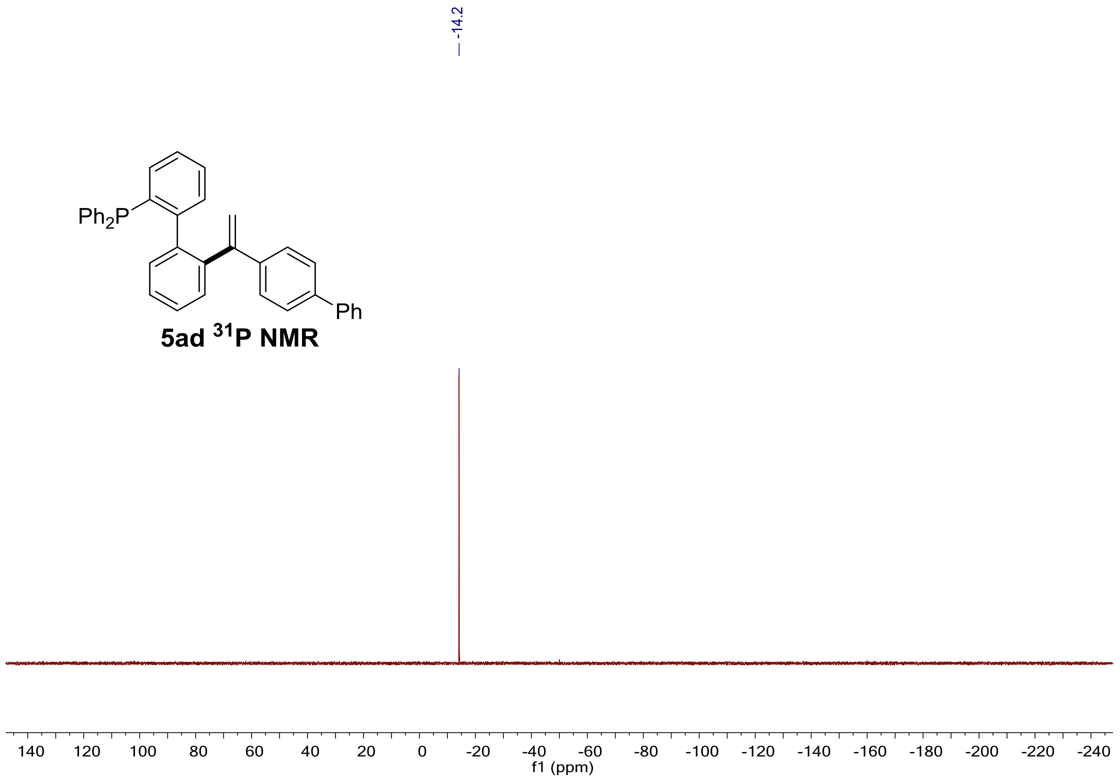

Supplementary Figure 38. ^{1}H, ^{13}C and ^{31}P NMR of compound **5ad.**

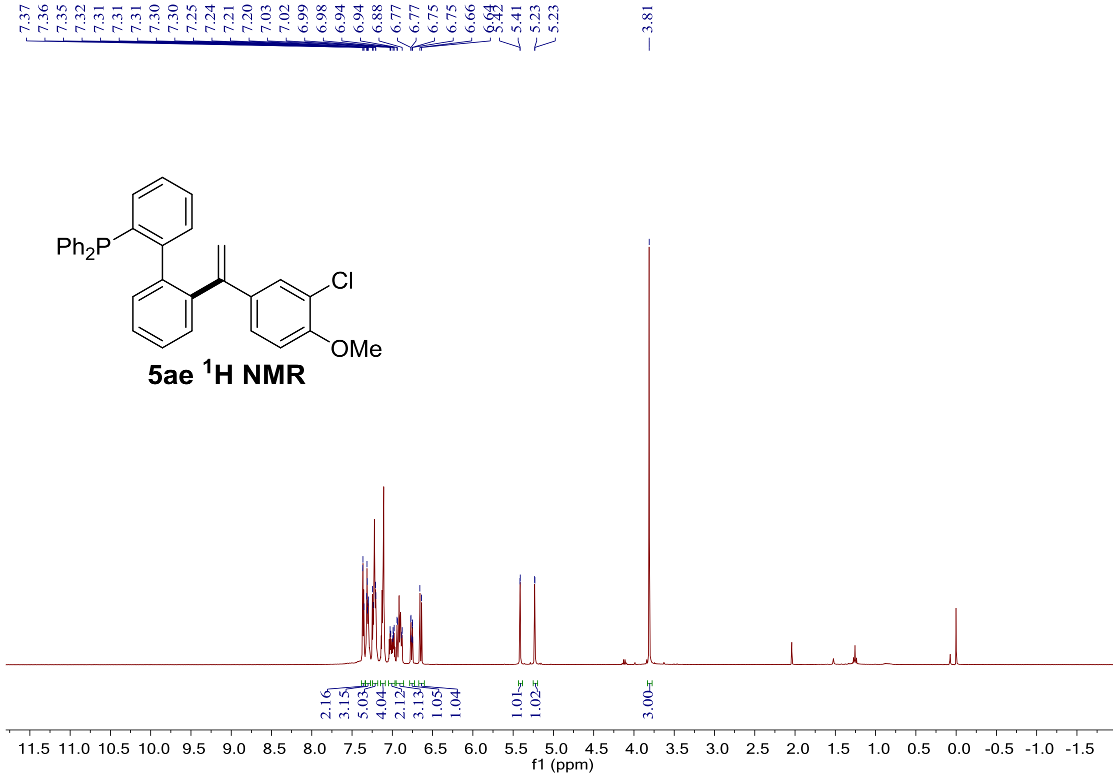

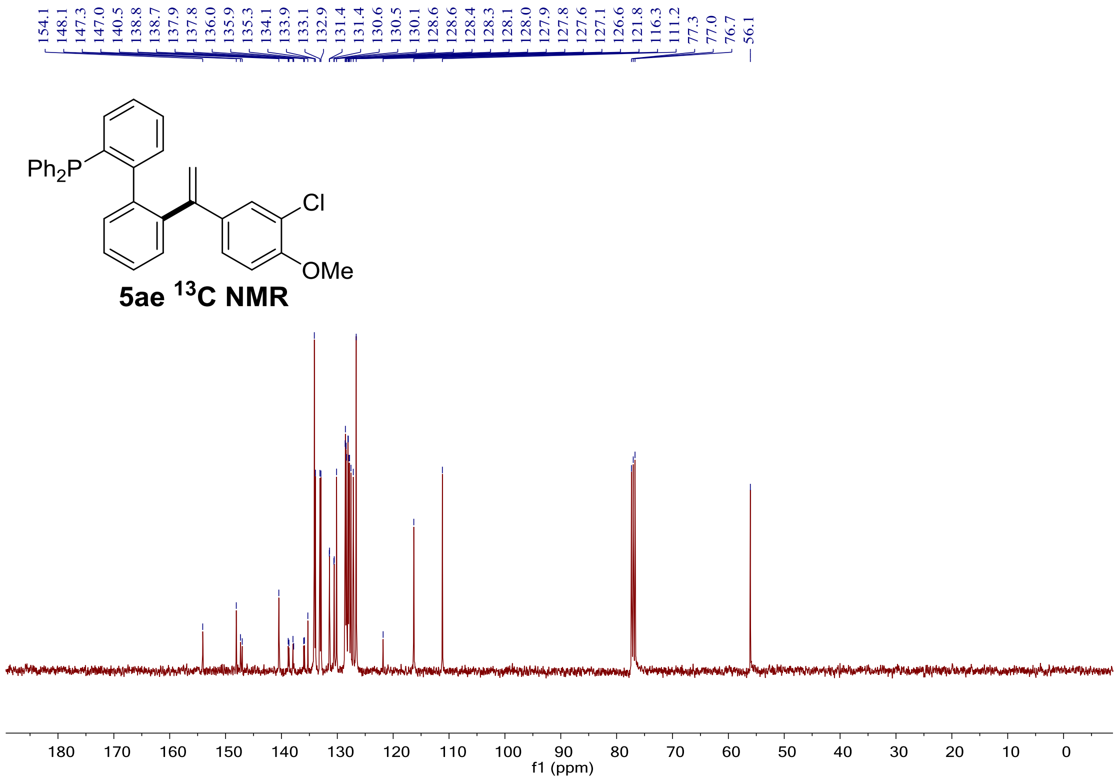

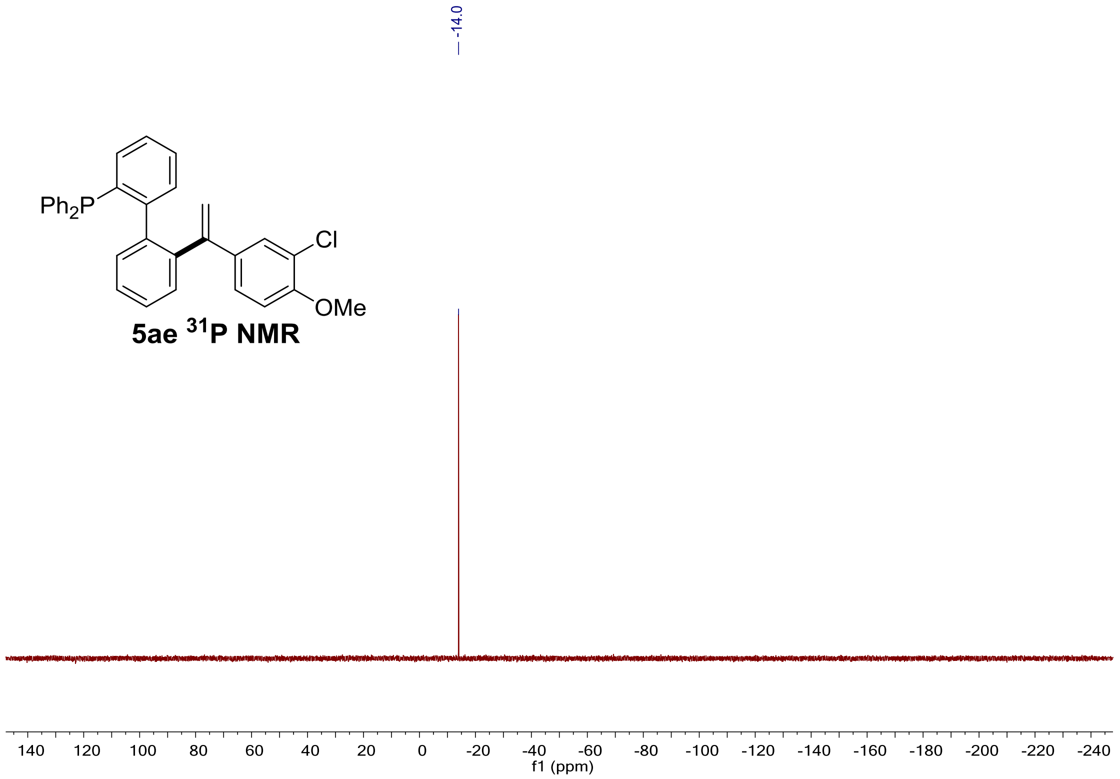

Supplementary Figure 39. ^{1}H, ^{13}C and ^{31}P NMR of compound **5ae.**

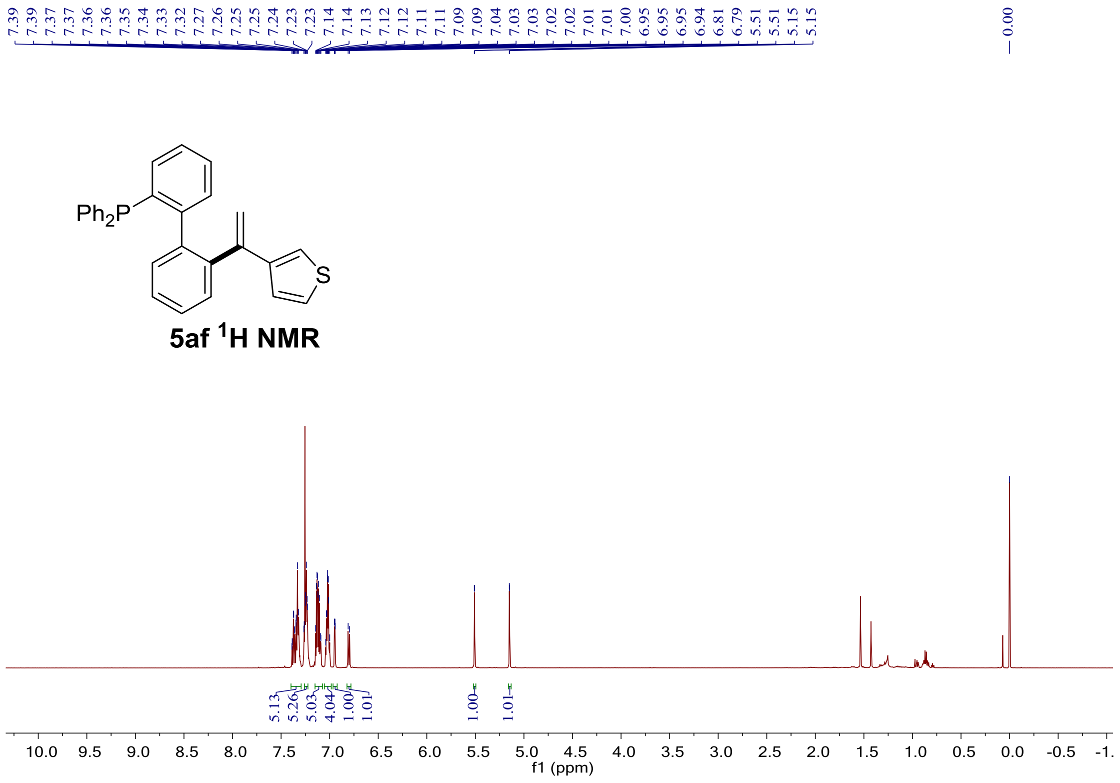
7.39 7.39 7.37 7.37 7.36 7.35 7.34 7.33 7.32 7.27 7.26 7.25 7.24 7.23 7.23 7.14 7.14 7.13 7.12 7.12 7.11 7.11 7.09 7.09 7.04 7.03 7.03 7.02 7.02 7.01 7.01 7.00 6.95 6.95 6.94 6.81 6.79 5.51 5.51 5.15 5.15
0.00
Ph2P
S
5af 1H NMR
5.13 5.26 5.03 4.04 1.00 1.01 1.00 1.01
10.0 9.5 9.0 8.5 8.0 7.5 7.0 6.5 6.0 5.5 5.0 4.5 4.0 3.5 3.0 2.5 2.0 1.5 1.0 0.5 0.0 -0.5
f1 (ppm)

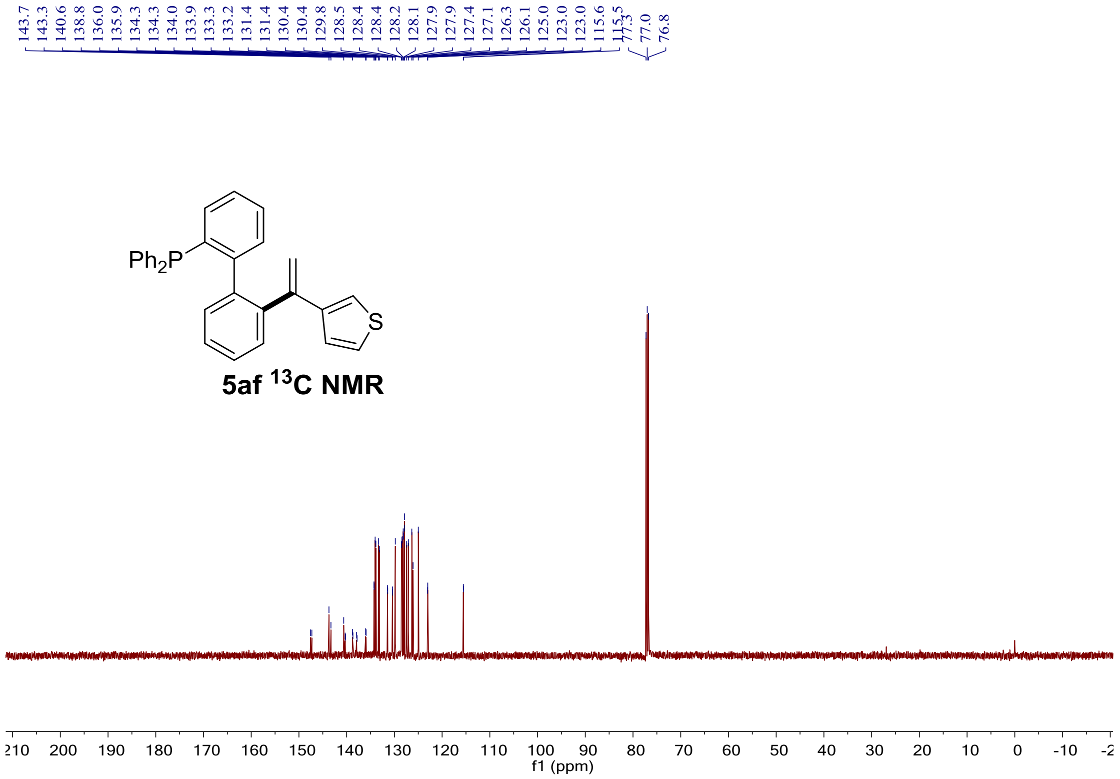
143.7 143.3 140.6 138.8 136.0 135.9 134.3 134.3 134.0 133.9 133.3 133.2 131.4 131.4 130.4 130.4 129.8 128.5 128.4 128.4 128.2 128.1 127.9 127.9 127.4 127.1 126.3 126.1 125.0 123.0 123.0 115.6 77.3 77.0 76.8
Ph2P
S
5af 13C NMR
200 190 180 170 160 150 140 130 120 110 100 90 80 70 60 50 40 30 20 10 0 -10
f1 (ppm)

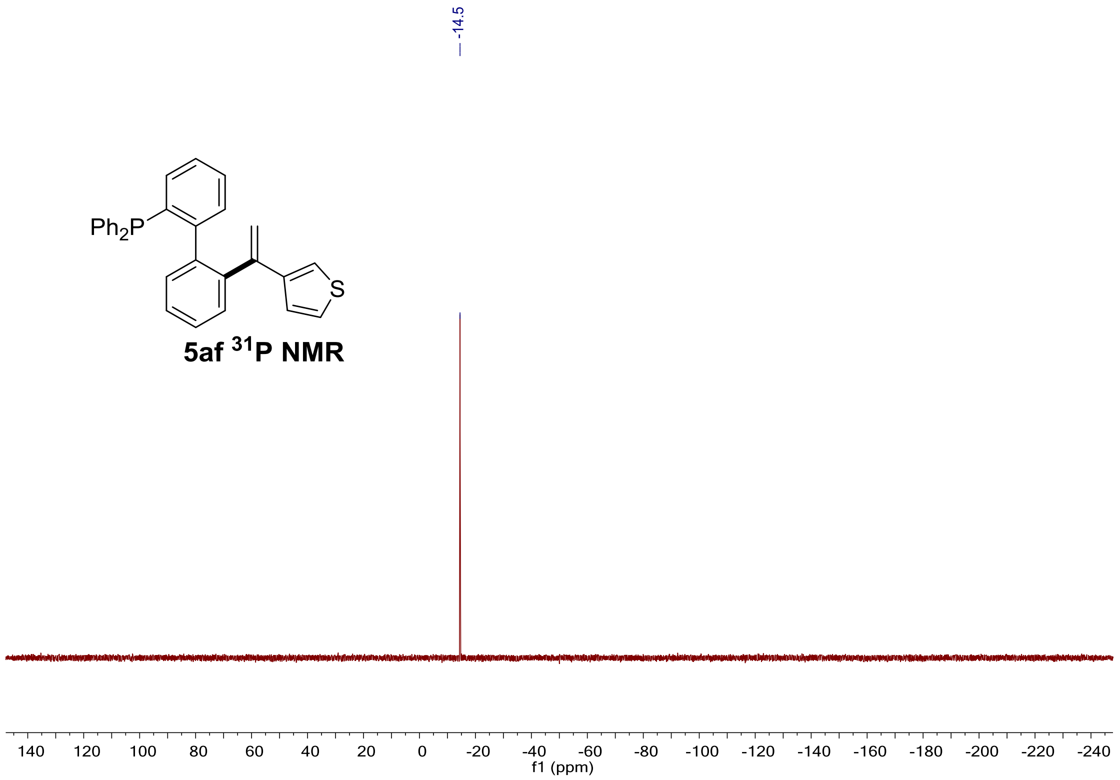

Supplementary Figure 40. ^{1}H, ^{13}C and ^{31}P NMR of compound **5af.**

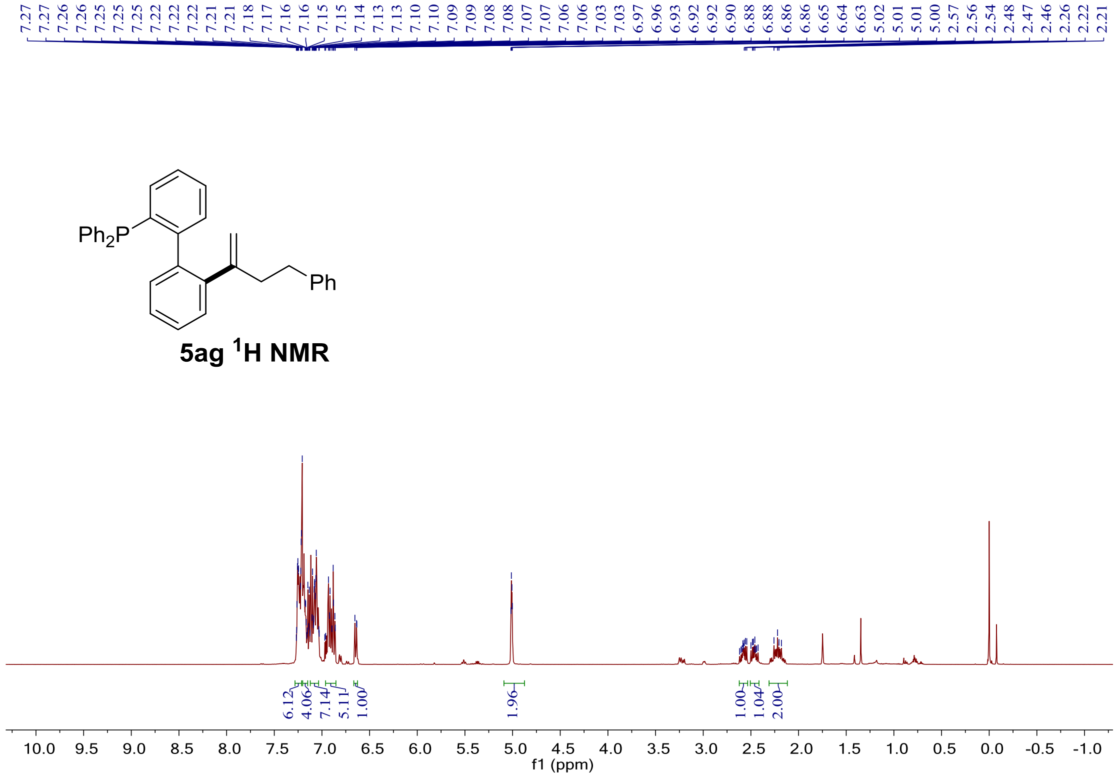

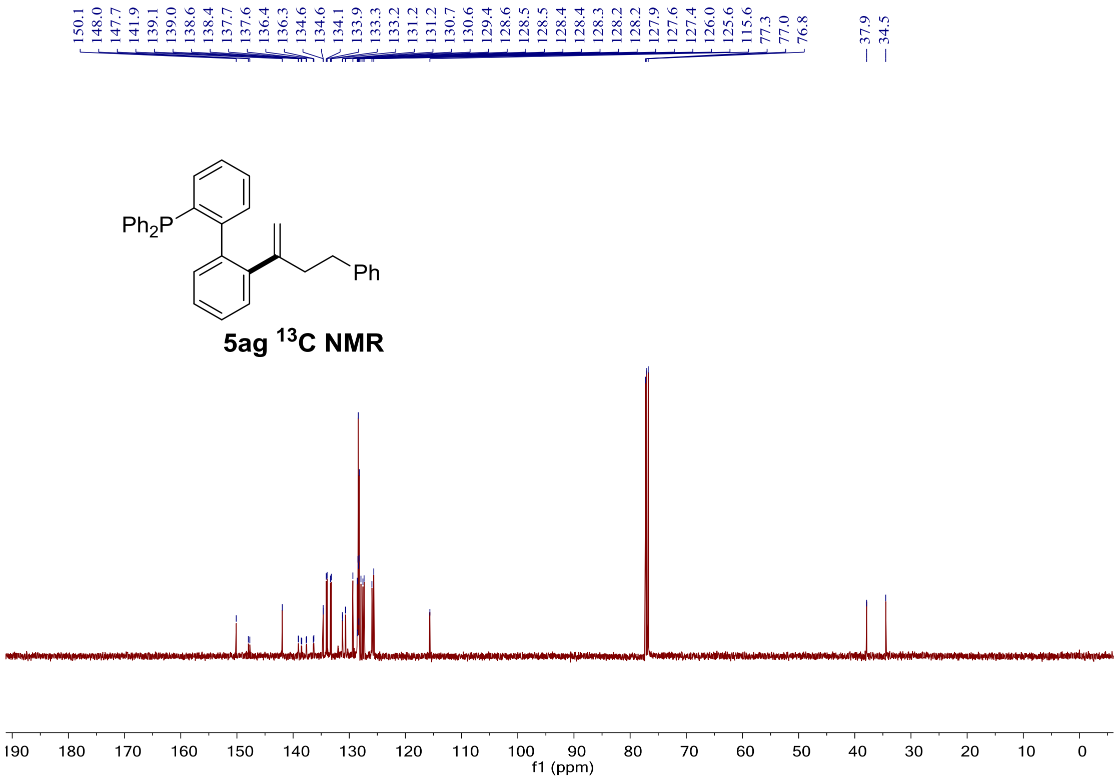

5ag ^{13}C NMR

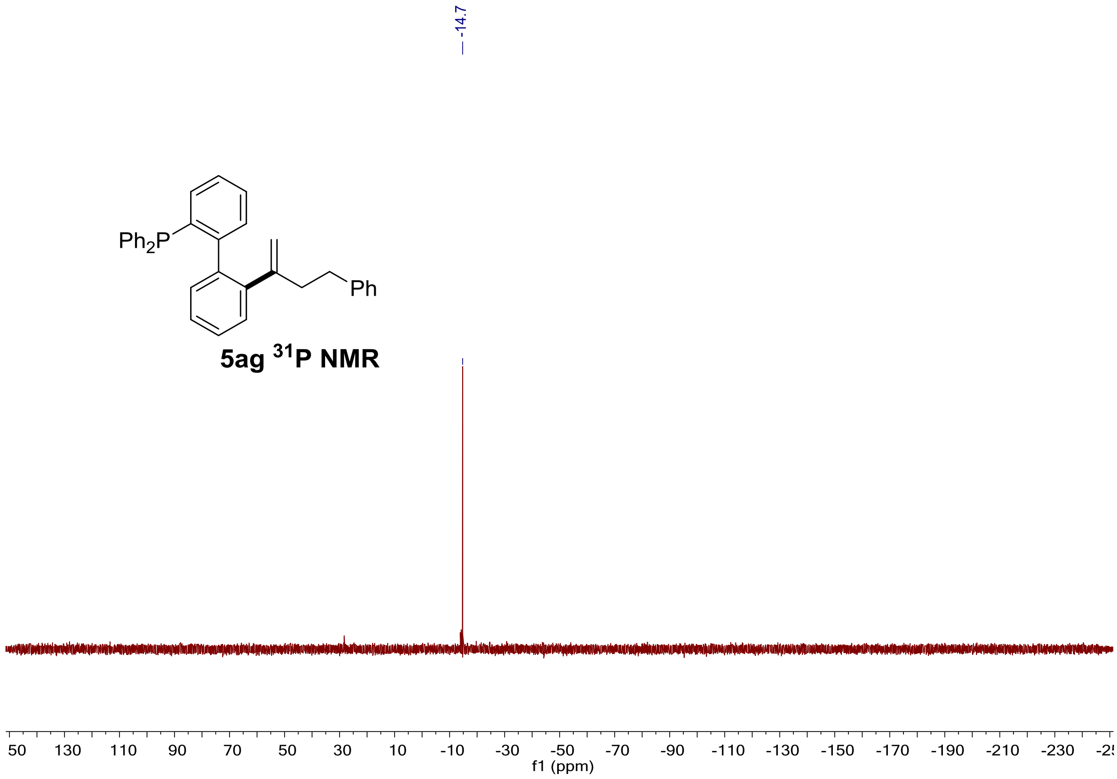

5ag ^{31}P NMR

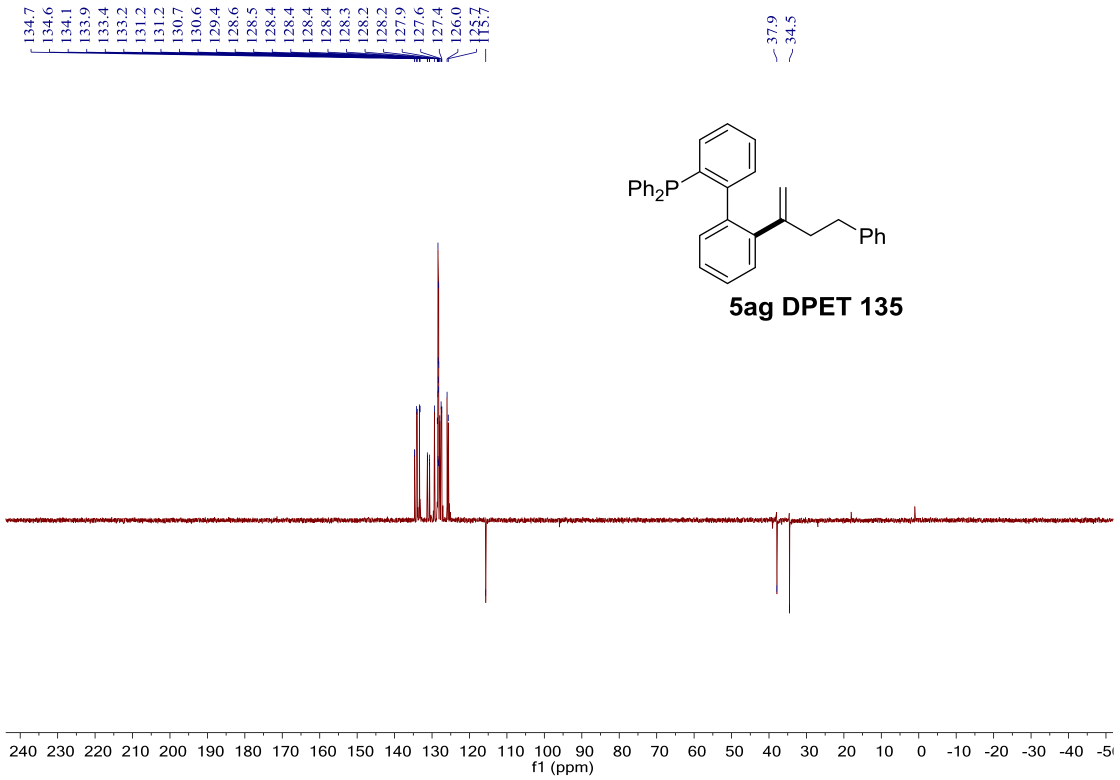
134.7
134.6
134.1
133.9
133.4
133.2
131.2
131.2
130.7
130.6
129.4
128.6
128.5
128.4
128.4
128.4
128.4
128.3
128.2
128.2
127.9
127.6
127.4
126.0
125.7
115.7
37.9
34.5
Ph2P
Ph
5ag DPET 135
f1 (ppm)

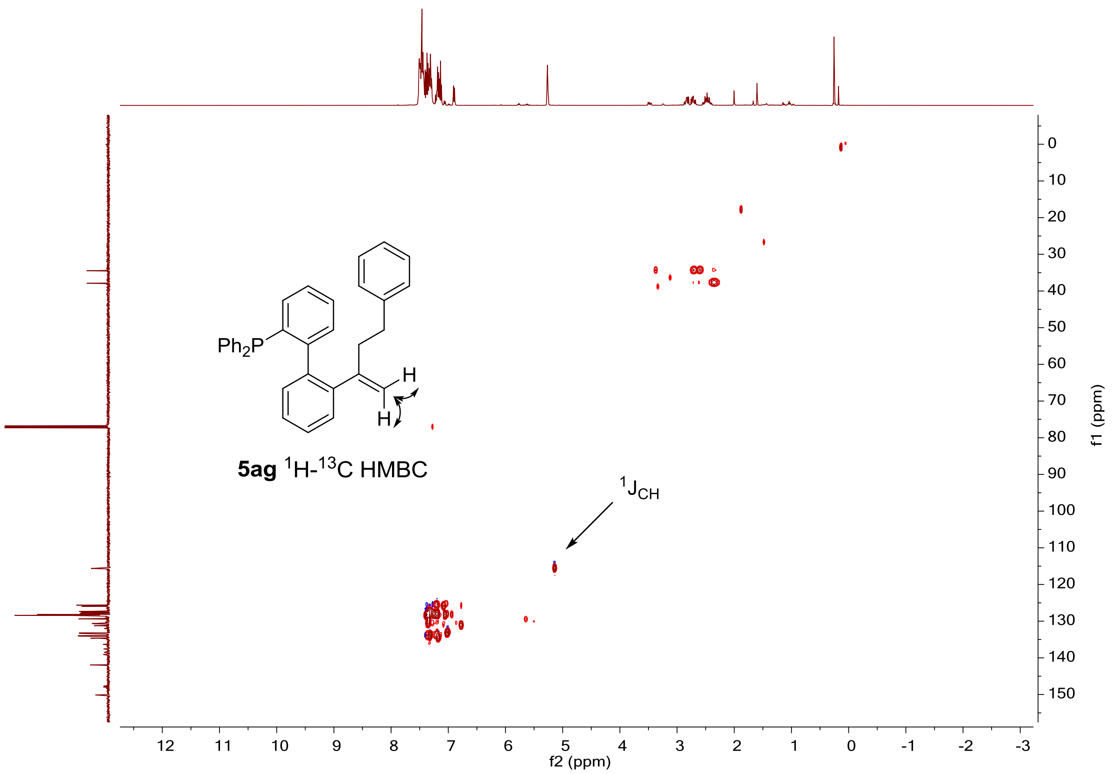
Ph2P
H
H
5ag 1H-13C HMBC
1JCH
f2 (ppm)
f1 (ppm)

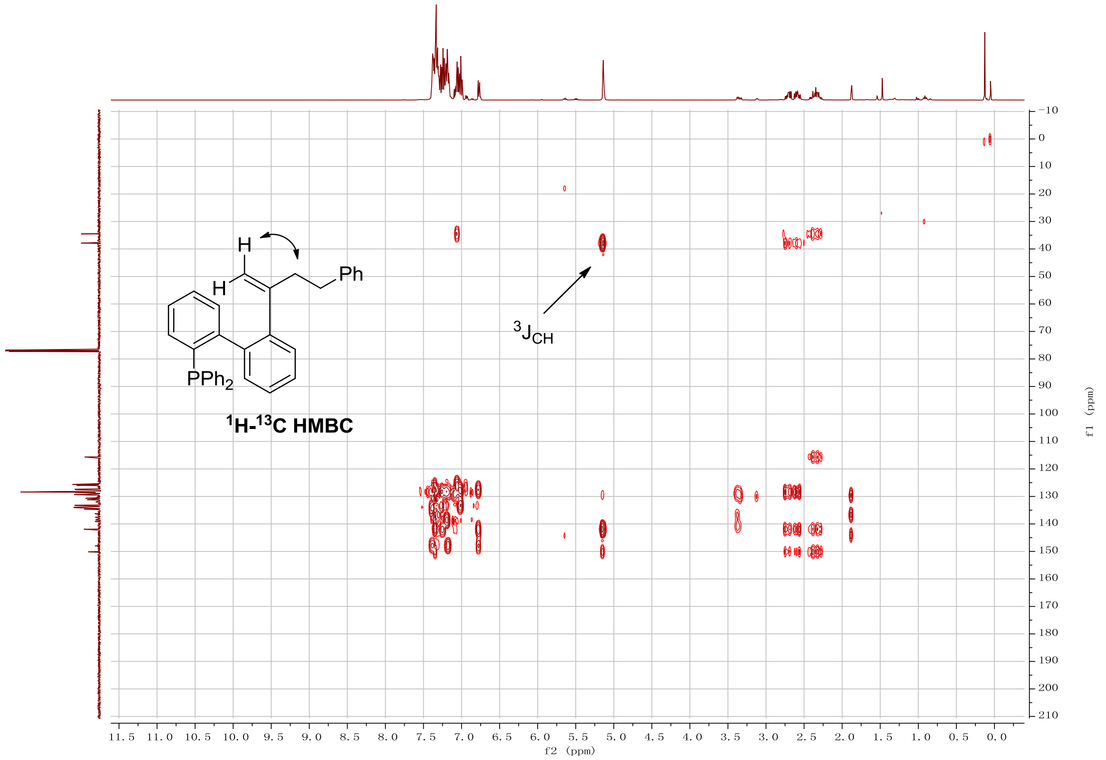

Supplementary Figure 41. ^{1}H, ^{13}C, ^{31}P NMR, DPET and 1H-13C HMBC of compound **5ag.**

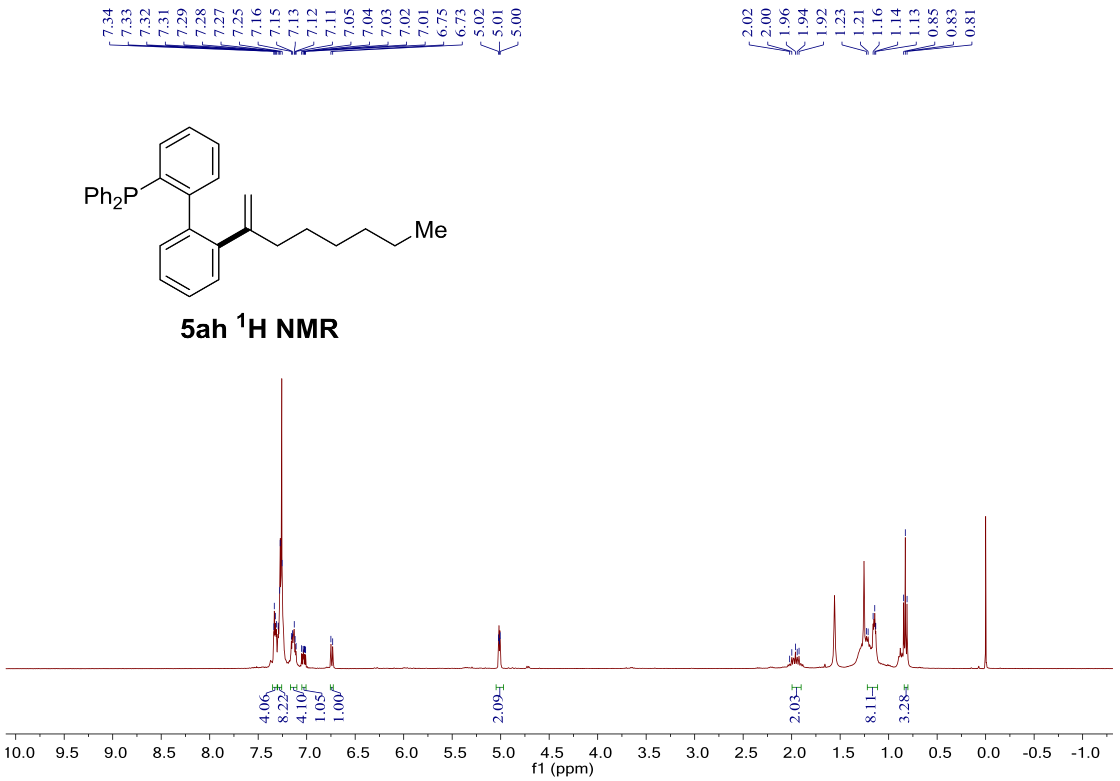

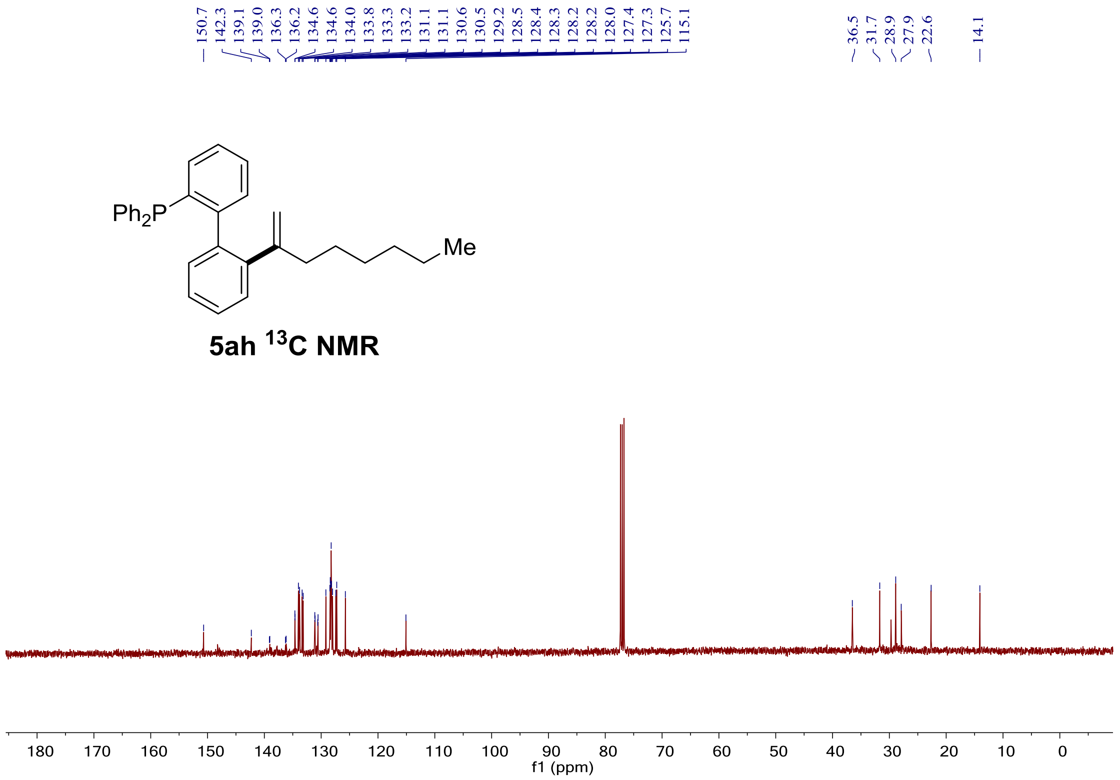

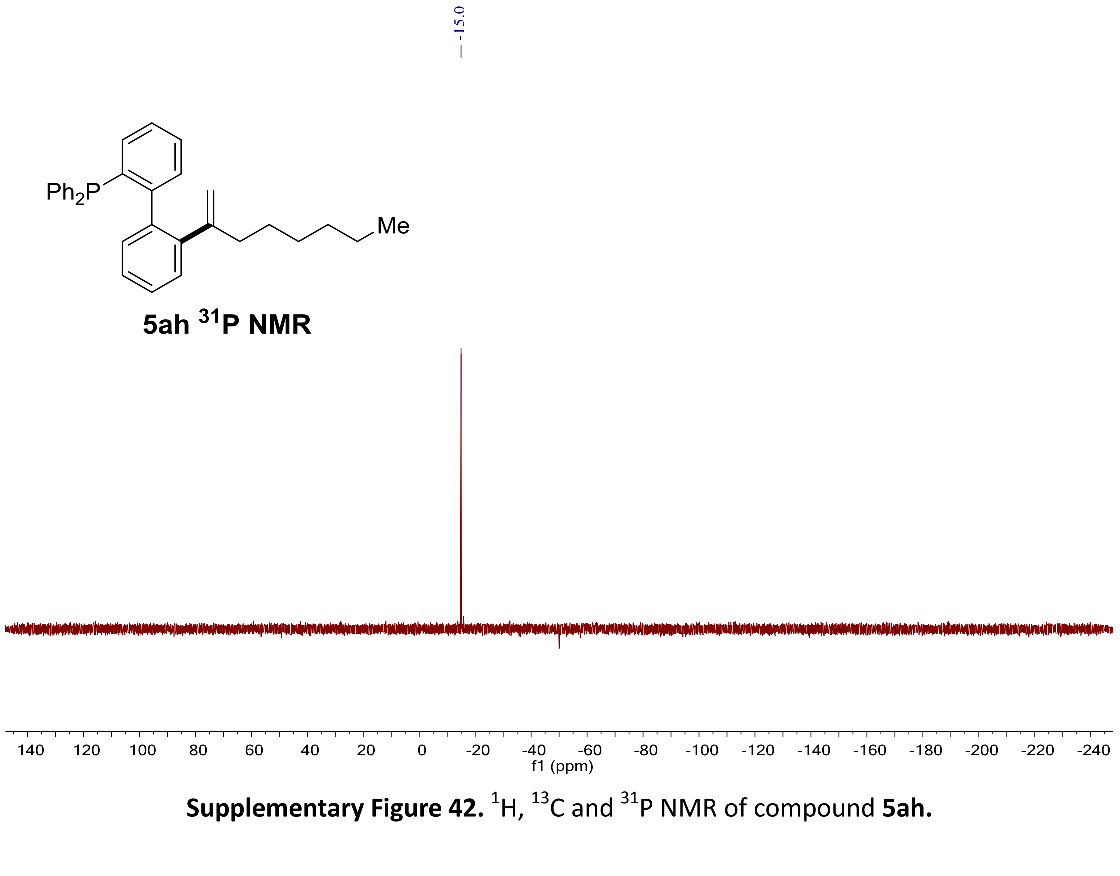

Supplementary Figure 42. ^{1}H, ^{13}C and ^{31}P NMR of compound **5ah.**

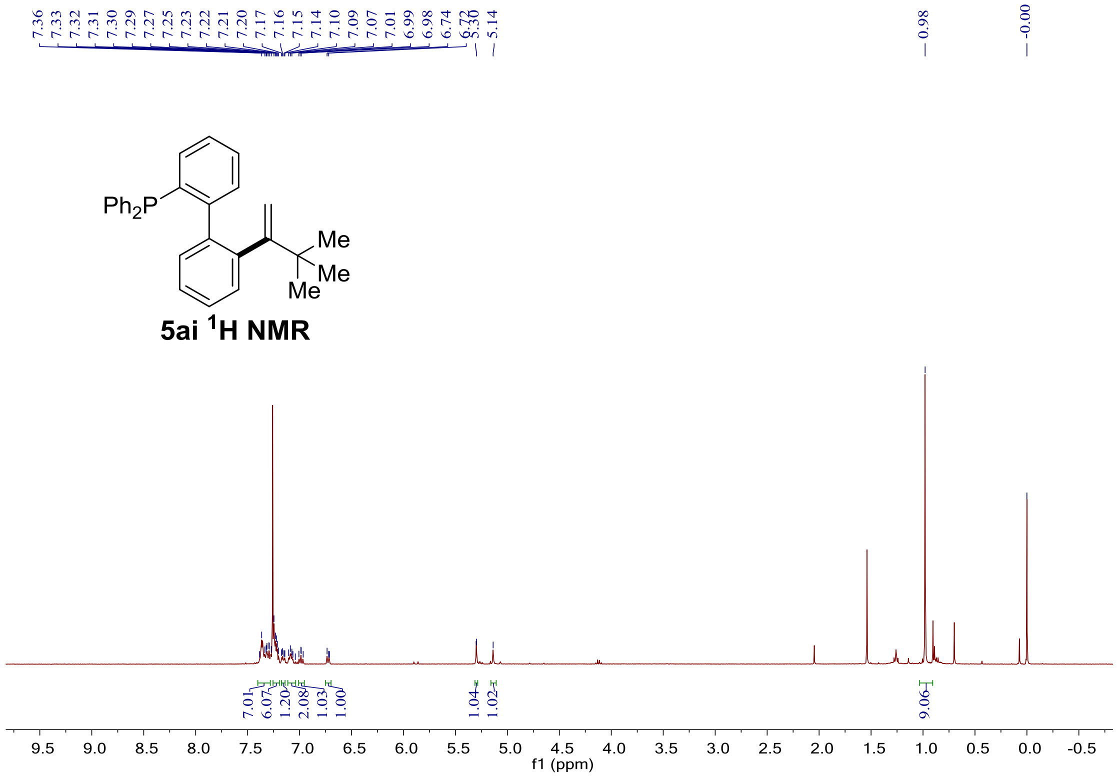
7.36
7.33
7.32
7.31
7.30
7.29
7.27
7.25
7.23
7.22
7.21
7.20
7.17
7.16
7.15
7.14
7.10
7.09
7.07
7.01
6.99
6.98
6.74
6.72
5.30
5.14
0.98
-0.00
Ph2P
Me
Me
Me
5ai 1H NMR
7.01
6.07
1.20
2.08
1.03
1.00
1.04
1.02
9.06
9.5
9.0
8.5
8.0
7.5
7.0
6.5
6.0
5.5
5.0
4.5
4.0
3.5
3.0
2.5
2.0
1.5
1.0
0.5
0.0
-0.5
f1 (ppm)

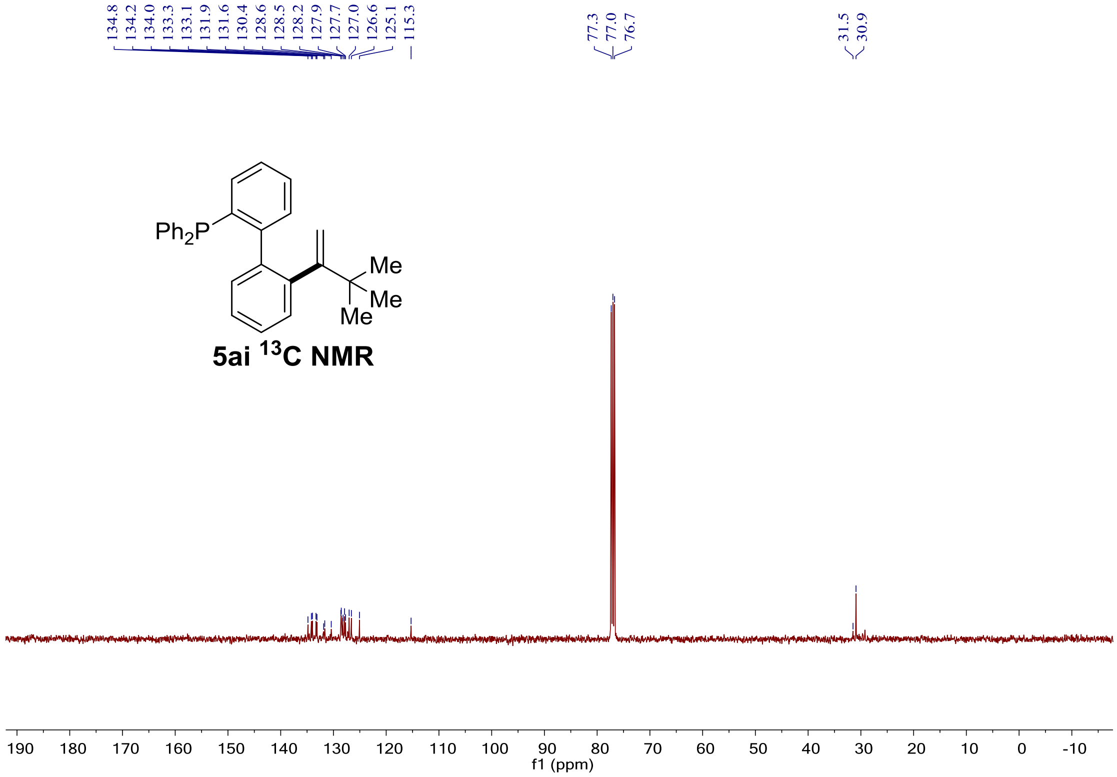
134.8
134.2
134.0
133.3
133.1
131.9
131.6
130.4
128.6
128.5
128.2
127.9
127.7
127.0
126.6
125.1
115.3
77.3
77.0
76.7
31.5
30.9
Ph2P
Me
Me
Me
5ai 13C NMR
190
180
170
160
150
140
130
120
110
100
90
80
70
60
50
40
30
20
10
0
-10
f1 (ppm)

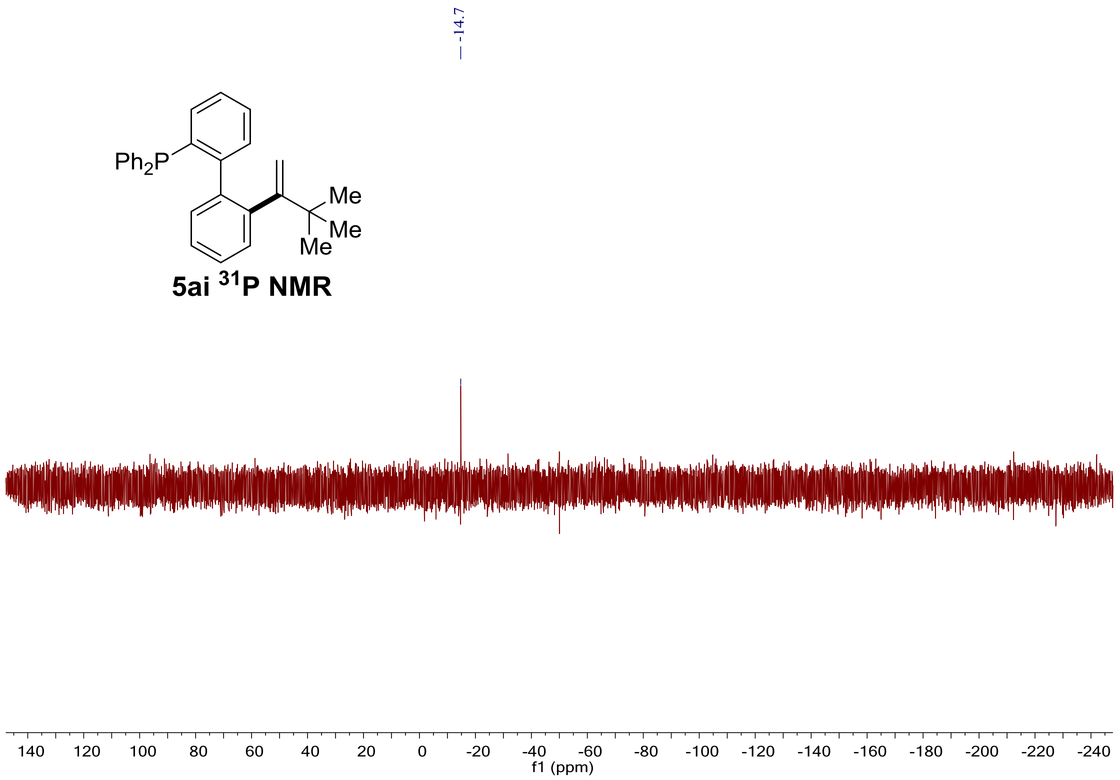

Supplementary Figure 43. ^{1}H, ^{13}C and ^{31}P NMR of compound **5ai.**

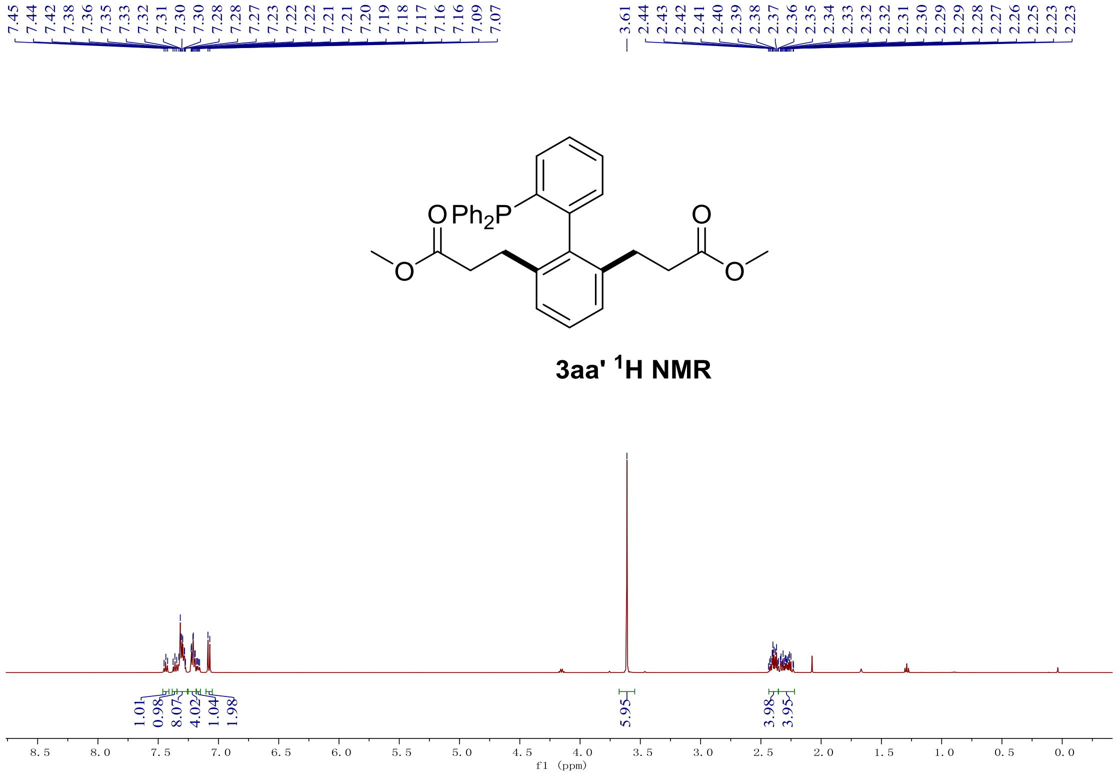

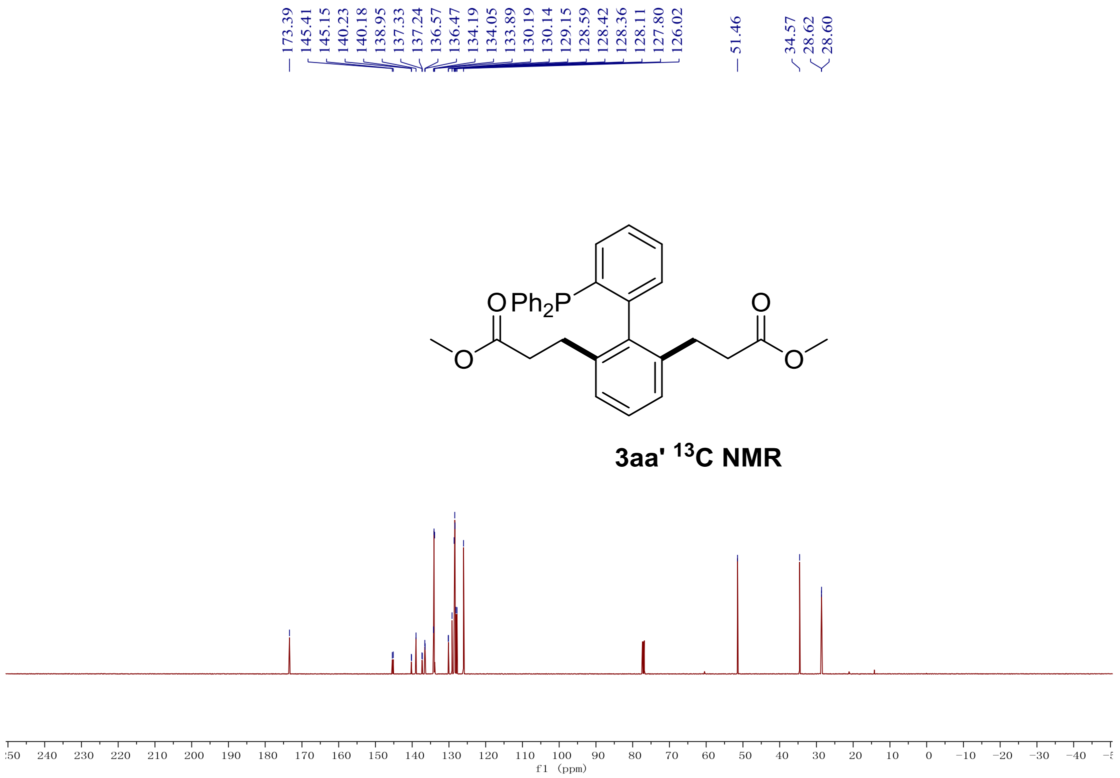

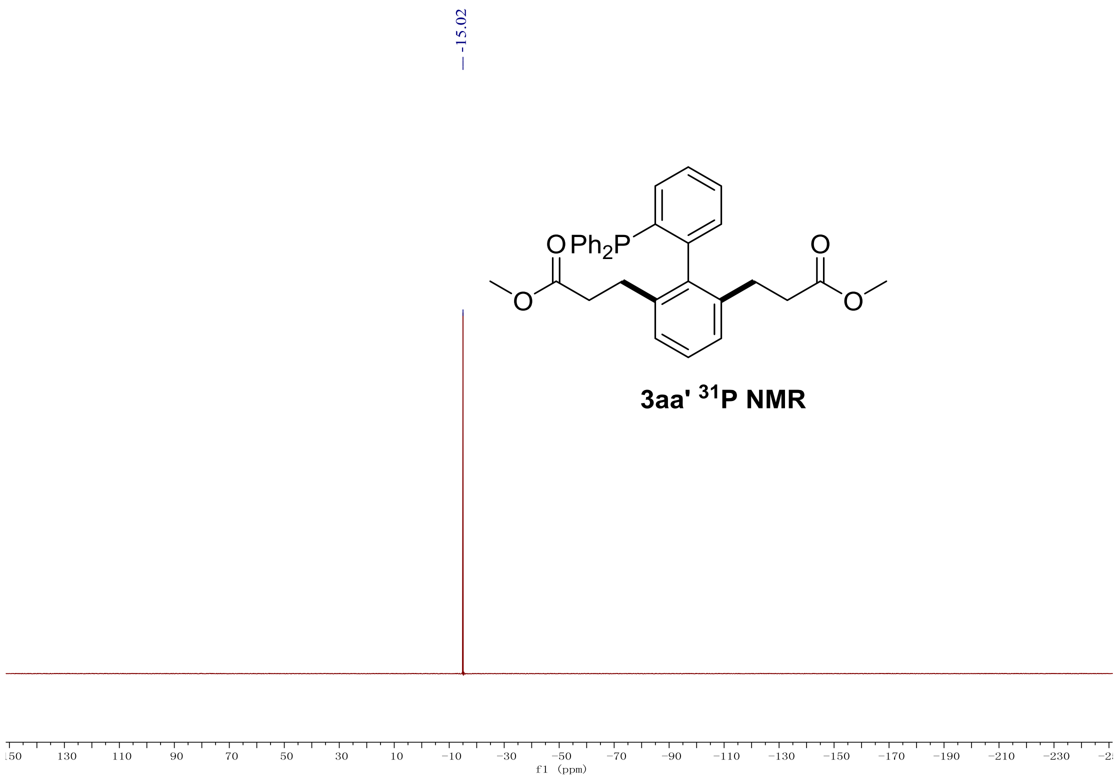

Supplementary Figure 44. ^{1}H, ^{13}C and ^{31}P NMR of compound **3aa'.**

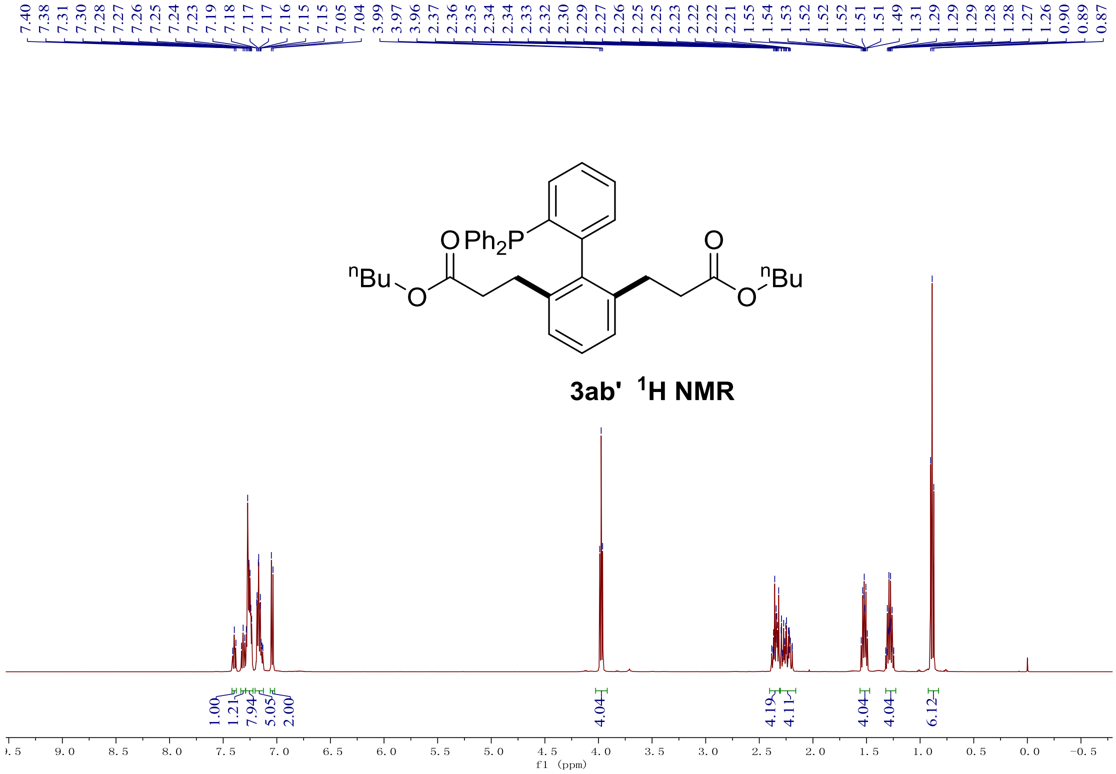
7.40 7.38 7.31 7.30 7.28 7.27 7.26 7.25 7.24 7.23 7.19 7.18 7.17 7.17 7.16 7.15 7.15 7.05 7.04 3.99 3.97 3.96 2.37 2.36 2.35 2.34 2.34 2.33 2.32 2.30 2.29 2.27 2.26 2.25 2.25 2.23 2.22 2.22 2.22 2.21 1.55 1.54 1.53 1.52 1.52 1.52 1.51 1.51 1.49 1.31 1.29 1.29 1.29 1.28 1.28 1.27 1.26 0.90 0.89 0.87
O Ph2P
O
nBu
O
O
nBu
3ab' 1H NMR
1.00 1.21 7.94 5.05 2.00 4.04 4.19 4.11 4.04 4.04 6.12
f1 (ppm)

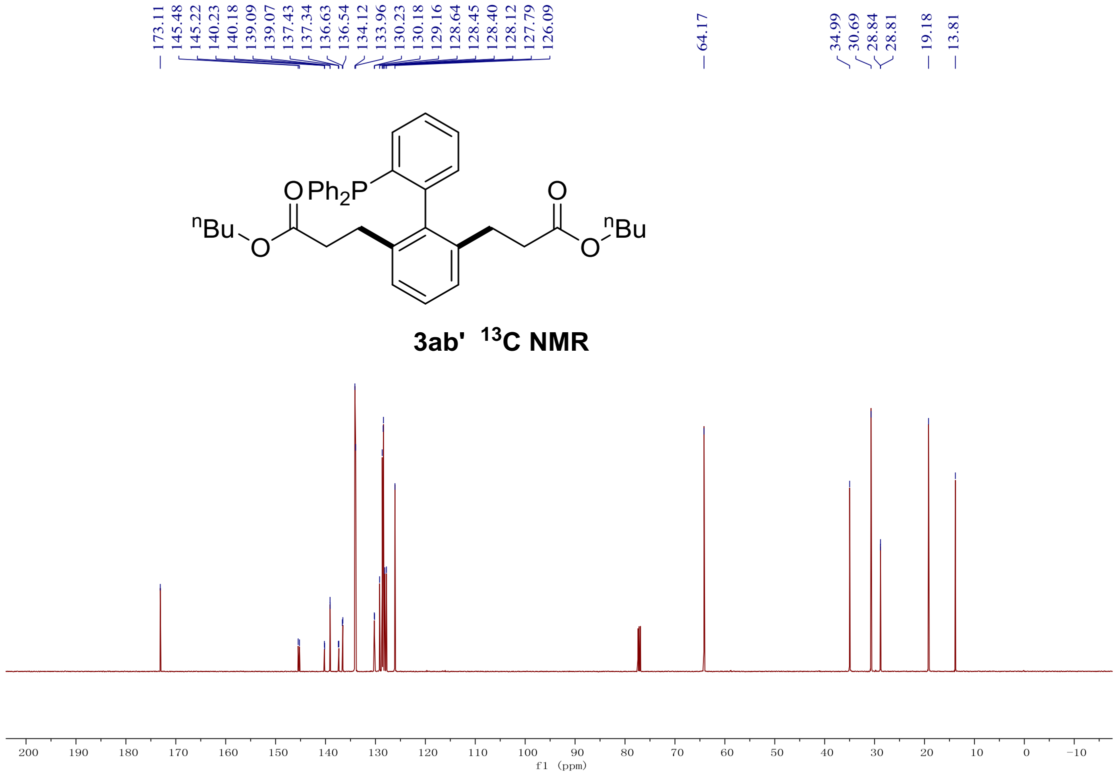
173.11 145.48 145.22 140.23 140.18 139.09 139.07 137.43 137.34 136.63 136.54 134.12 133.96 130.23 130.18 129.16 128.64 128.45 128.40 128.12 127.79 126.09 64.17 34.99 30.69 28.84 28.81 19.18 13.81
O Ph2P
O
nBu
O
O
nBu
3ab' 13C NMR
f1 (ppm)

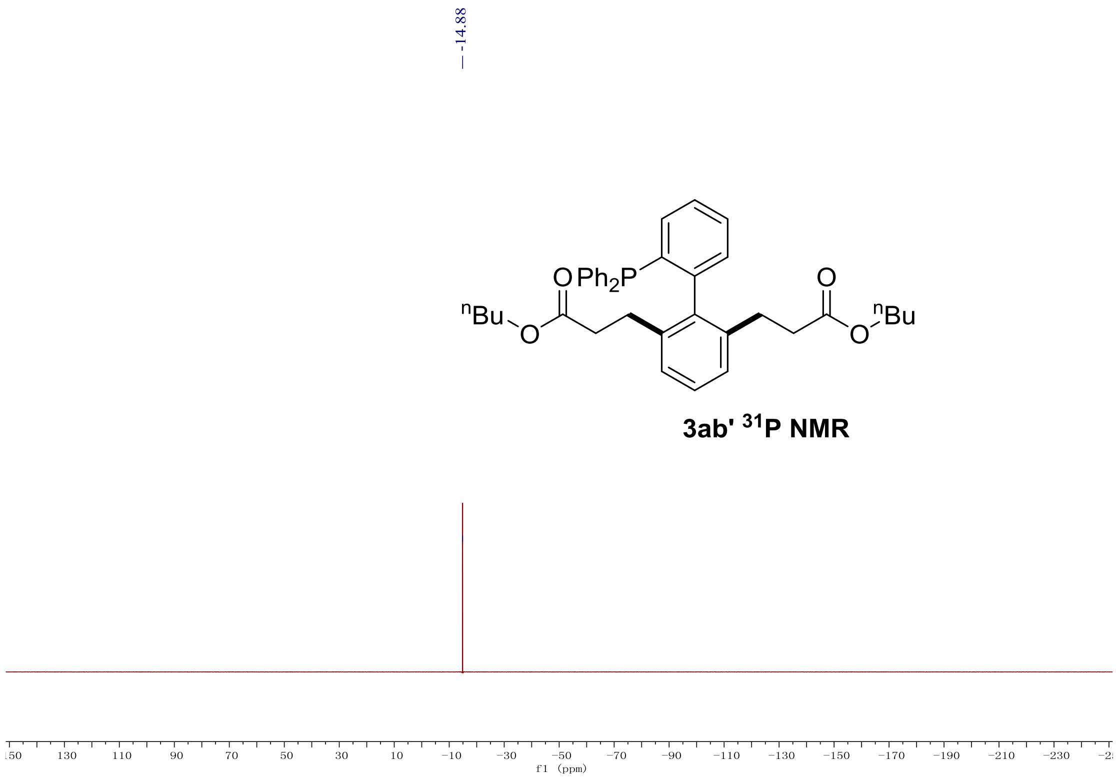

Supplementary Figure 45. ^{1}H, ^{13}C and ^{31}P NMR of compound **3ab.**

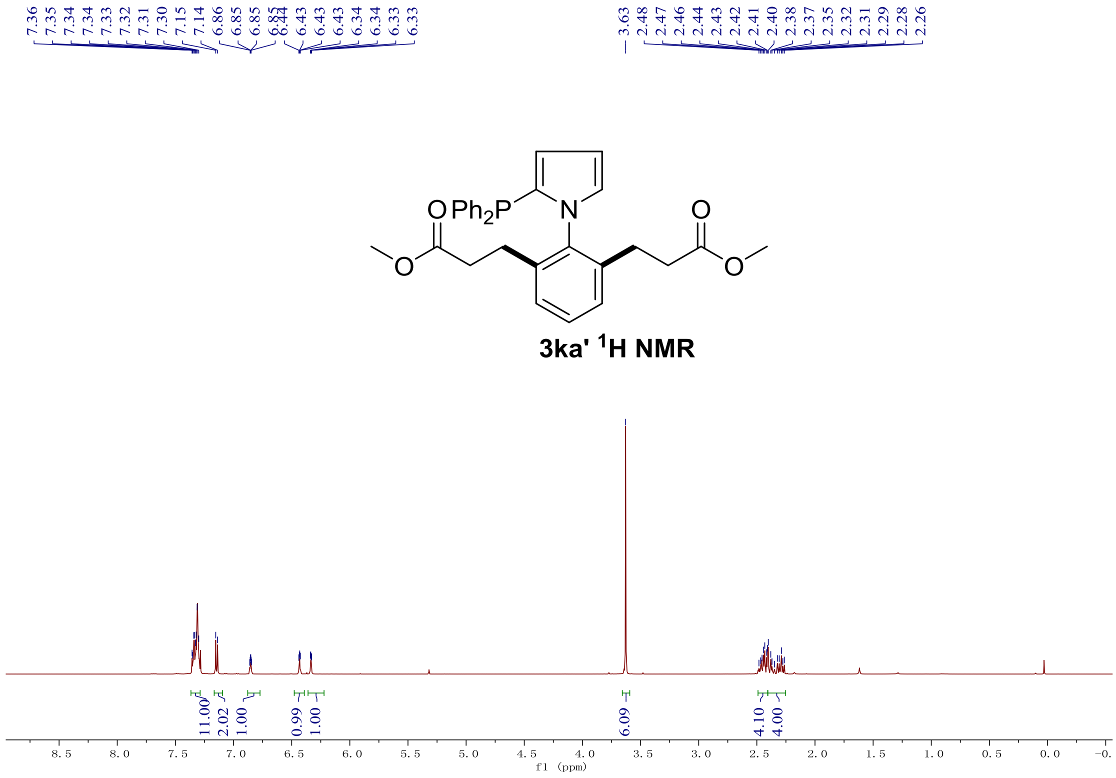

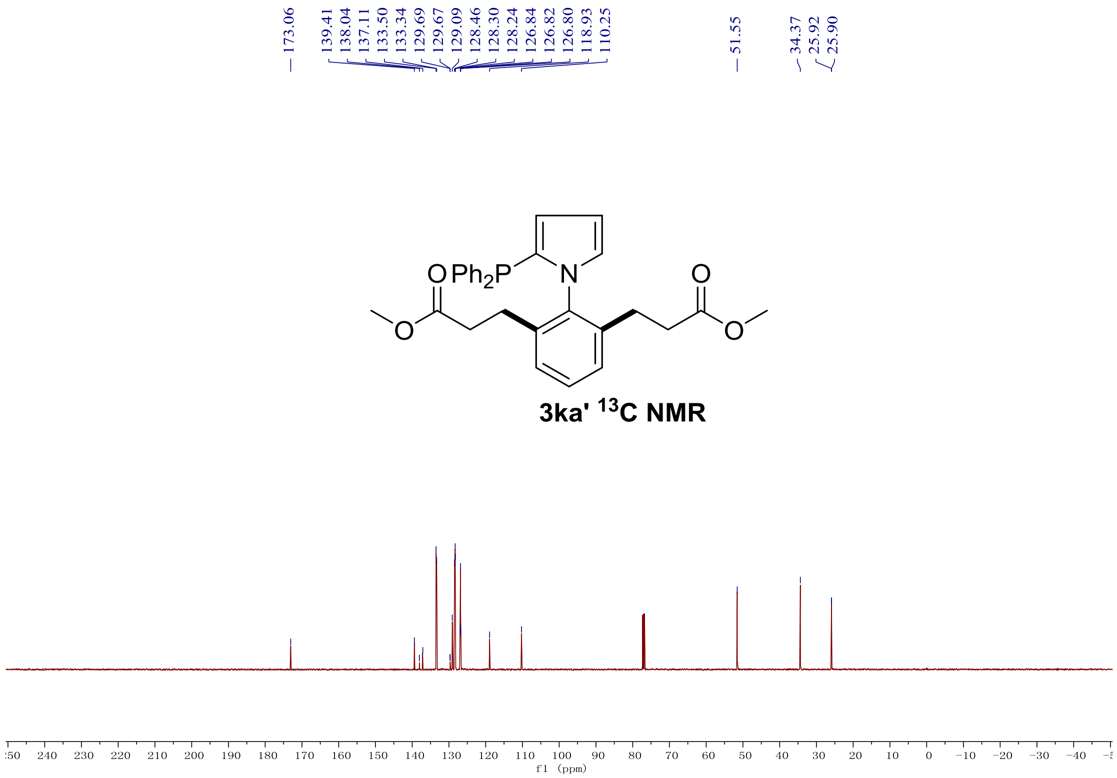

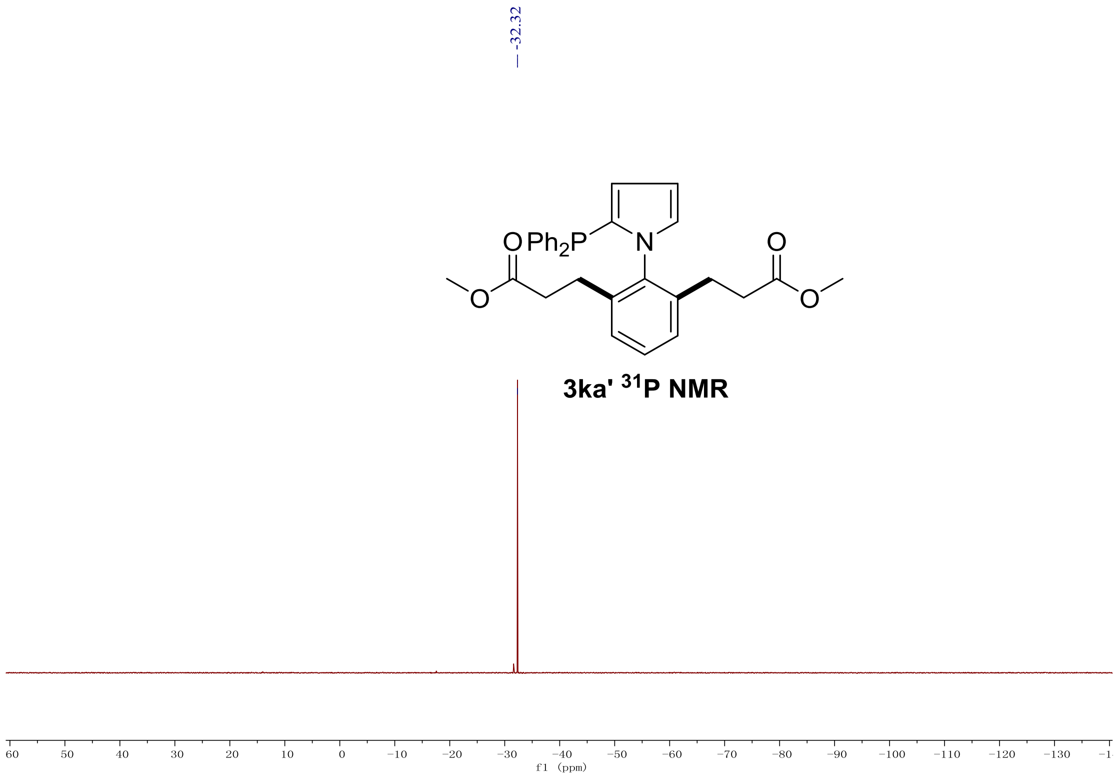

Supplementary Figure 46. ^{1}H, ^{13}C and ^{31}P NMR of compound **3ka.**

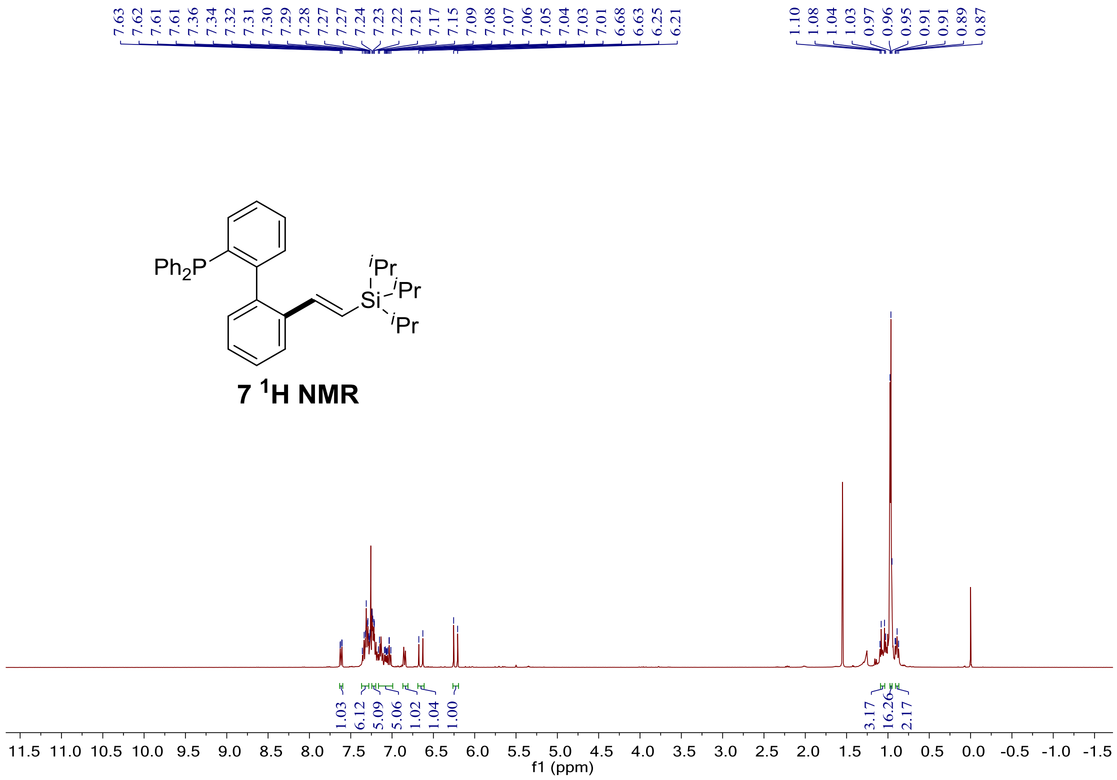

7 ^{1}H NMR

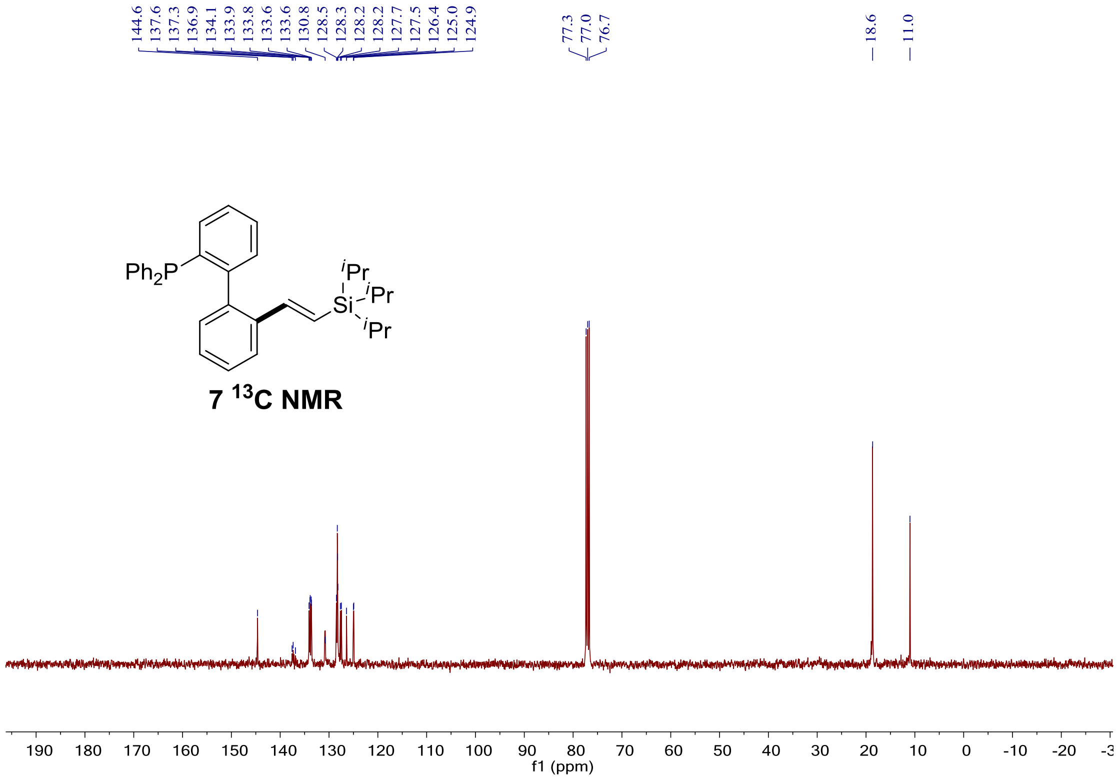

7 ^{13}C NMR

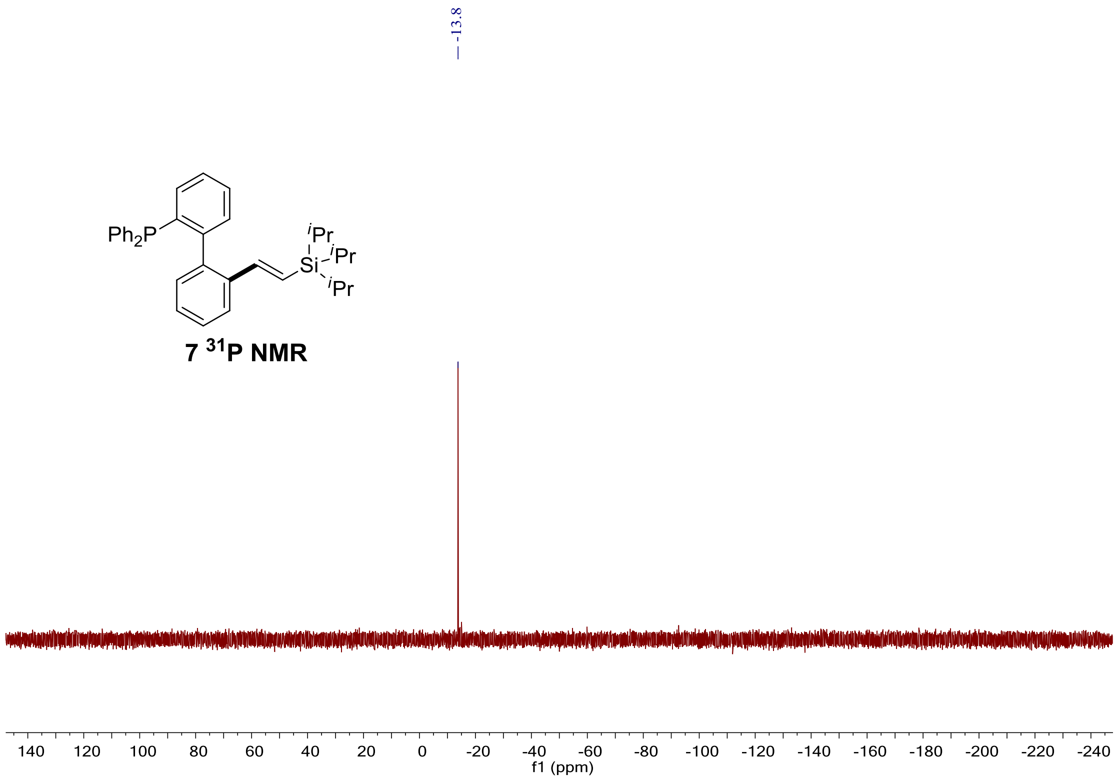

Supplementary Figure 47. ^{1}H, ^{13}C and ^{31}P NMR of compound **7.**

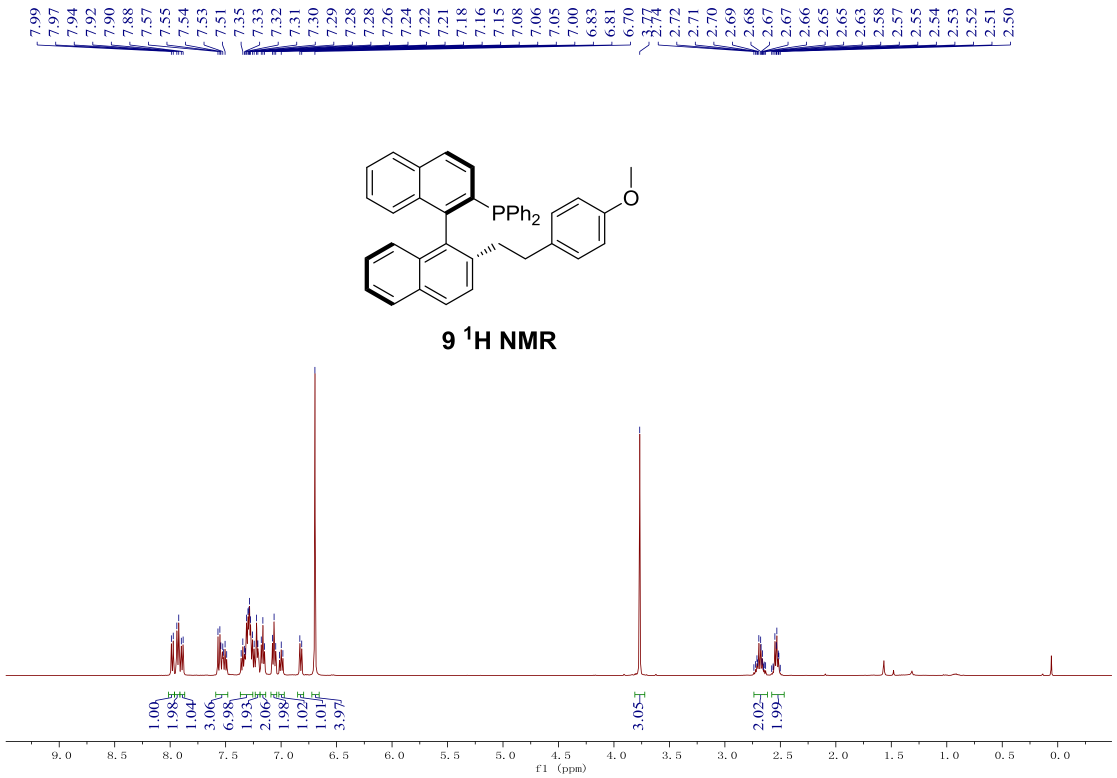
7.99
7.97
7.94
7.92
7.90
7.88
7.57
7.55
7.54
7.53
7.51
7.35
7.33
7.32
7.31
7.30
7.29
7.28
7.26
7.24
7.22
7.21
7.18
7.16
7.15
7.08
7.06
7.05
7.00
6.83
6.81
6.70
3.77
3.74
2.72
2.71
2.70
2.69
2.68
2.67
2.67
2.66
2.65
2.65
2.63
2.58
2.57
2.55
2.54
2.53
2.52
2.51
2.50
PPh2
O
9 1H NMR
1.00
1.98
1.04
3.06
6.98
1.93
2.06
1.98
1.02
1.01
3.97
3.05
2.02
1.99
f1 (ppm)

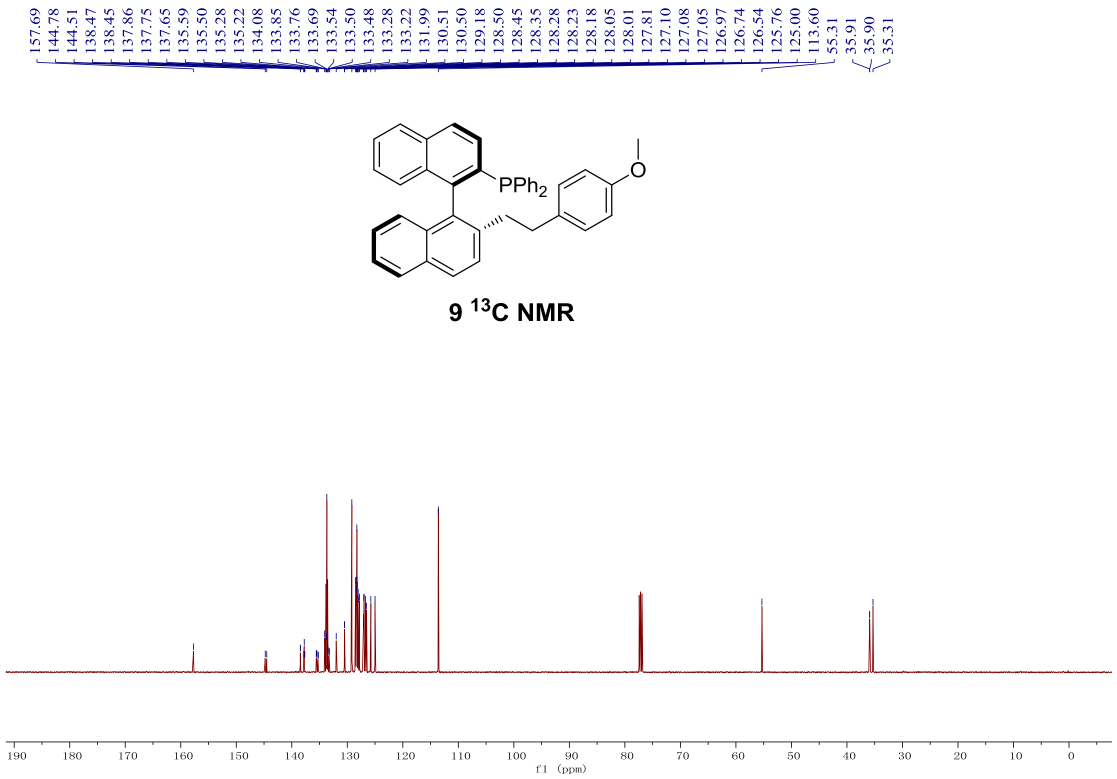
157.69
144.78
144.51
138.47
138.45
137.86
137.75
137.65
135.59
135.50
135.28
135.22
134.08
133.85
133.76
133.69
133.54
133.50
133.48
133.28
133.22
131.99
130.51
130.50
129.18
128.50
128.45
128.35
128.28
128.23
128.18
128.05
128.01
127.81
127.10
127.08
127.05
126.97
126.74
126.54
125.76
125.00
113.60
55.31
35.91
35.90
35.31
PPh2
O
9 13C NMR
f1 (ppm)

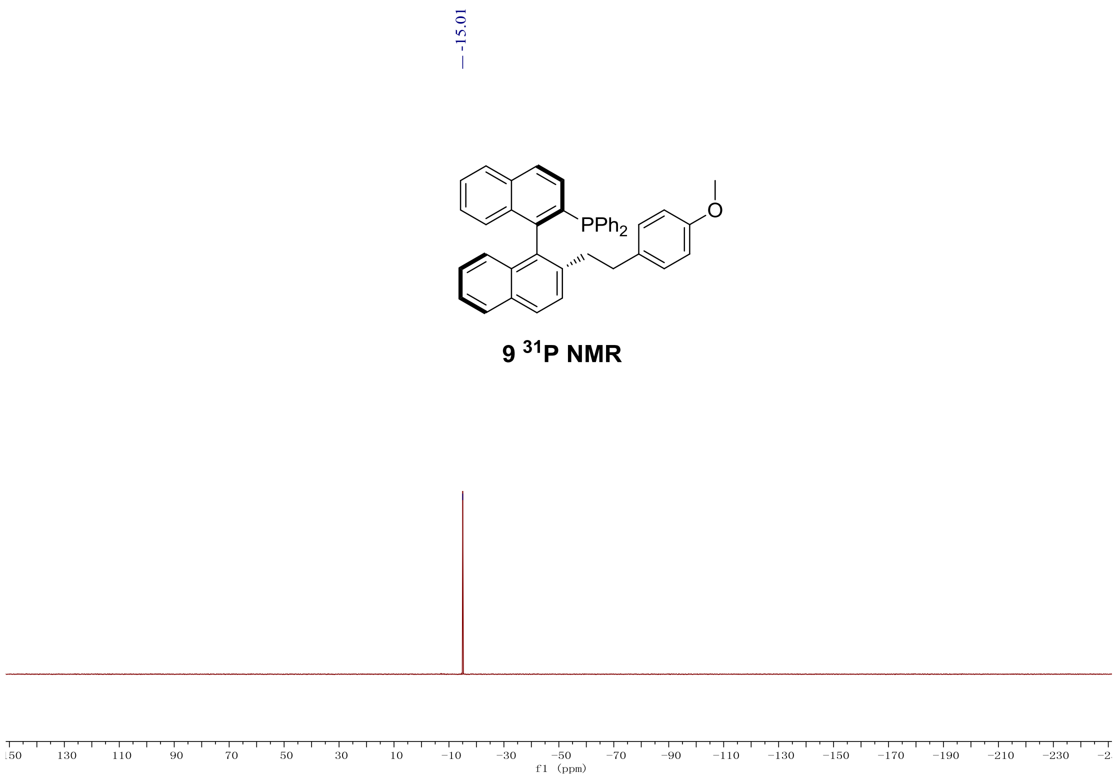

Supplementary Figure 48. ^{1}H, ^{13}C and ^{31}P NMR of compound **9.**

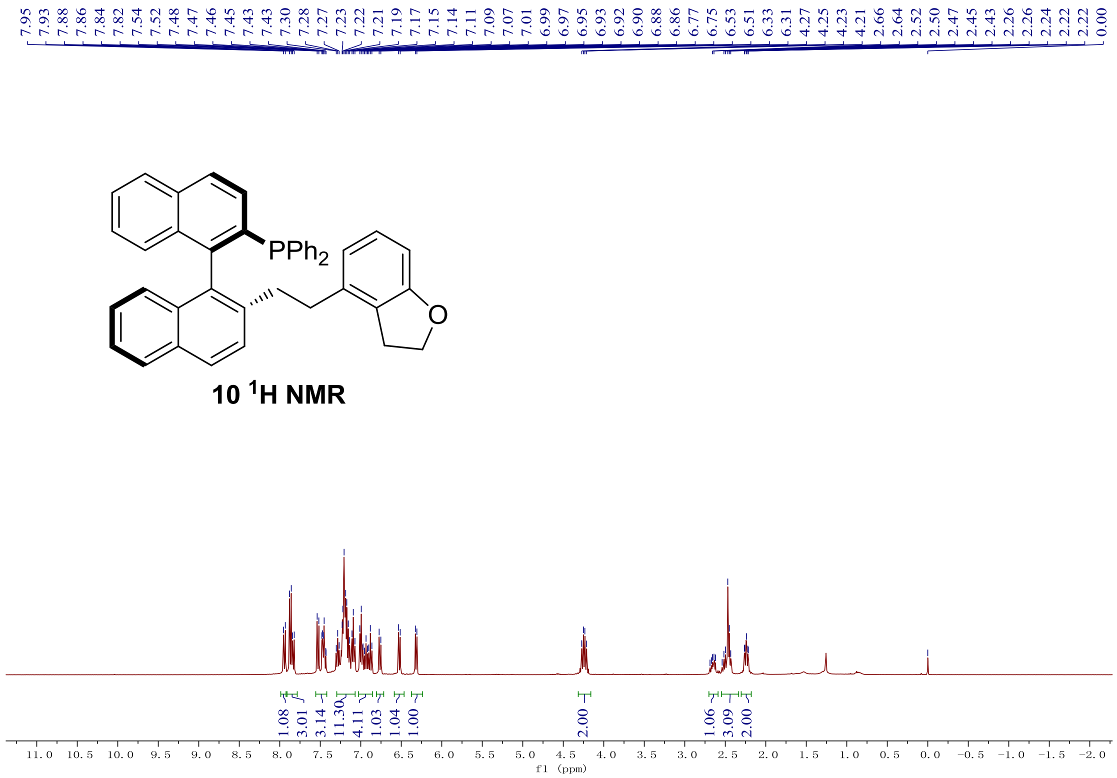

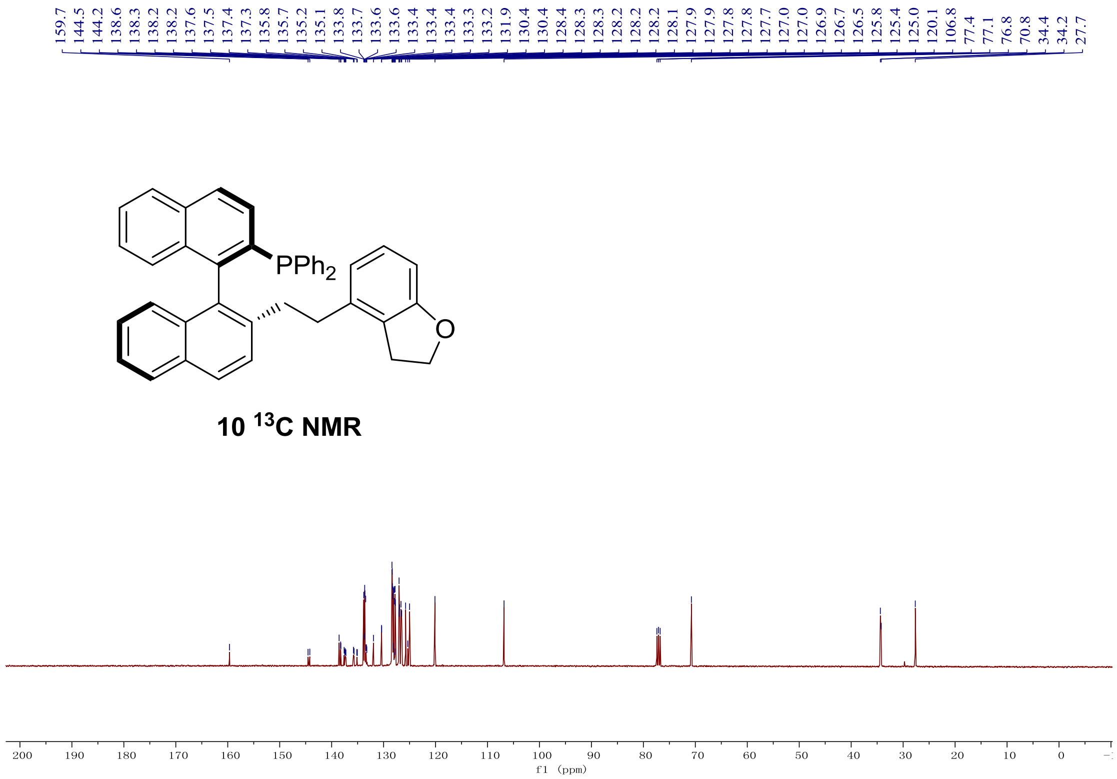

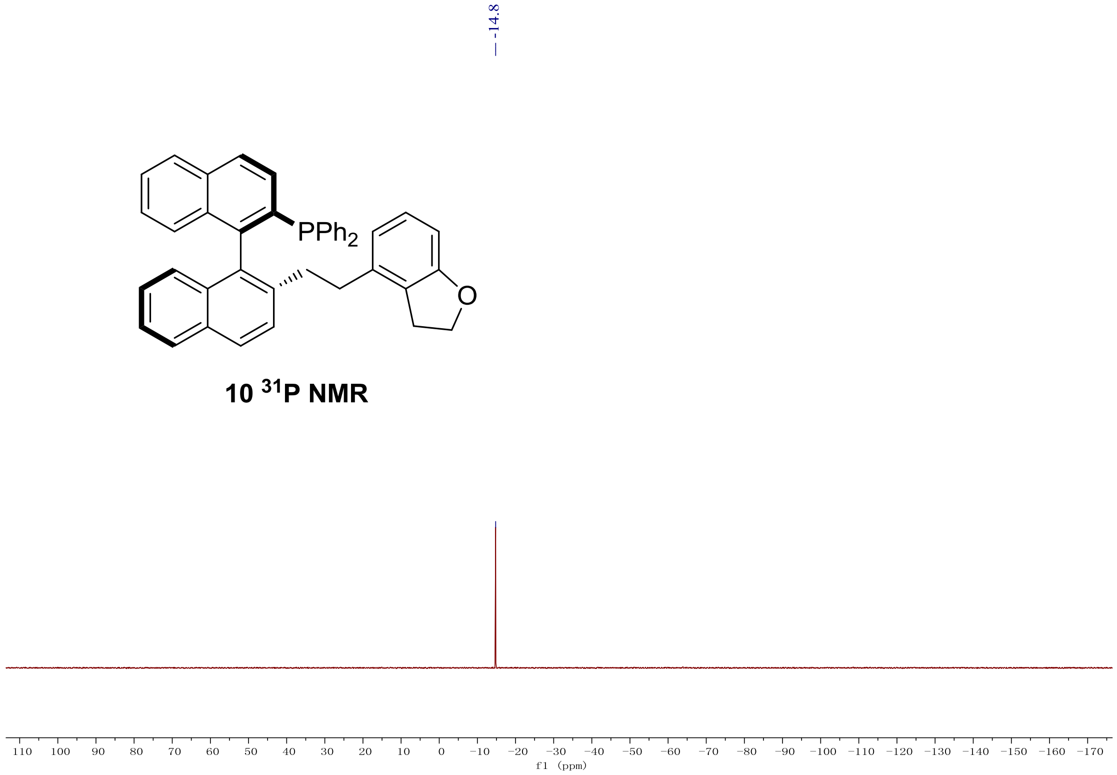

Supplementary Figure 49. ^{1}H, ^{13}C and ^{31}P NMR of compound **10.**

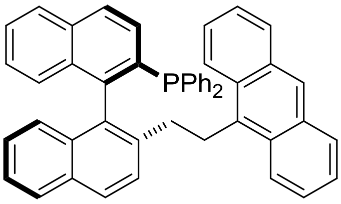

11 ^{1}H NMR

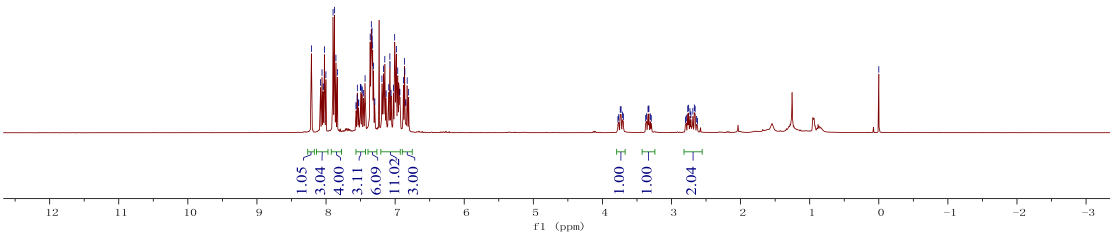

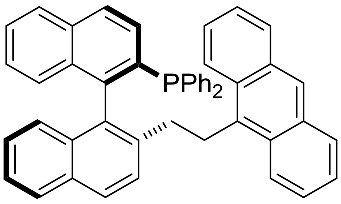

11 ^{13}C NMR

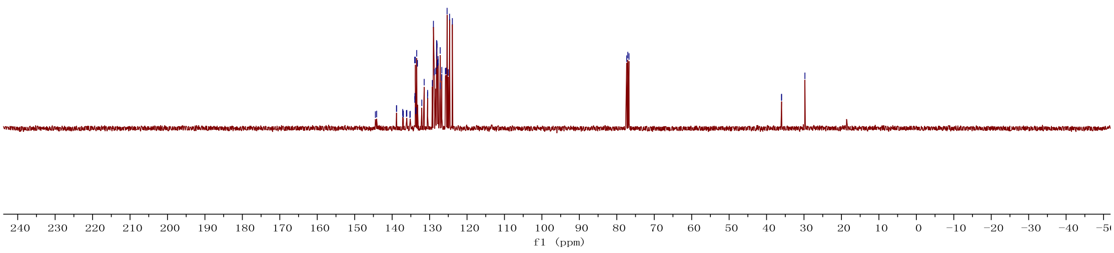

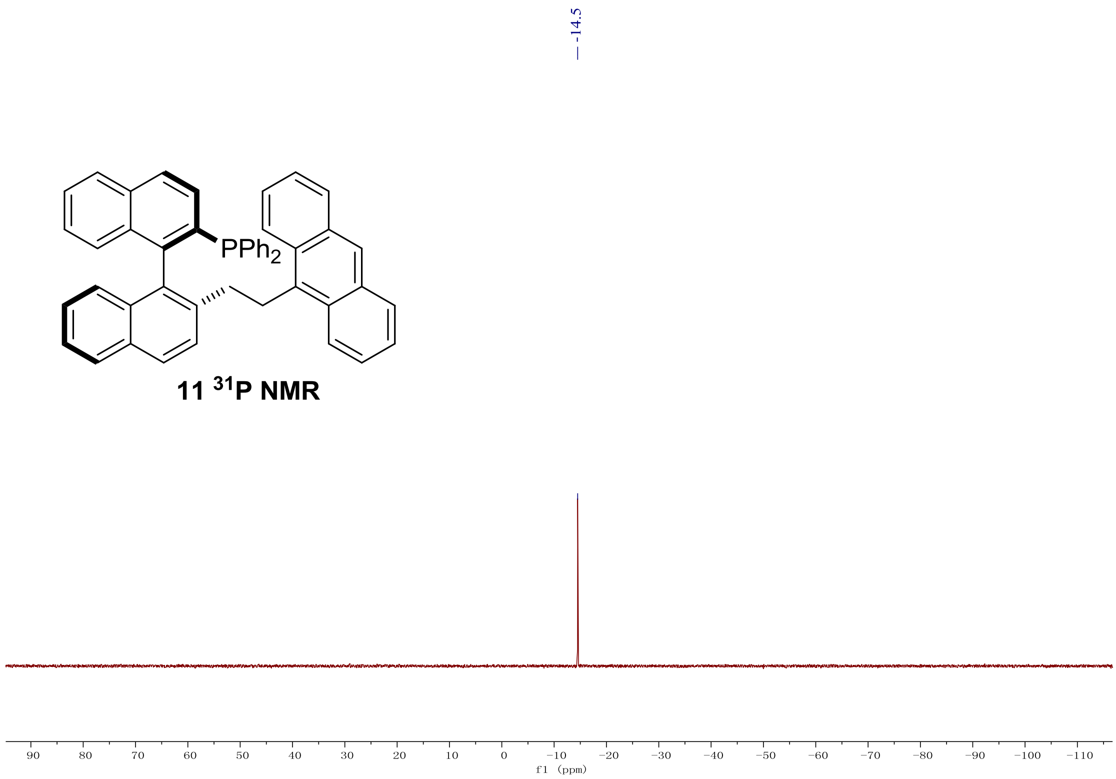

Supplementary Figure 50. ^{1}H, ^{13}C and ^{31}P NMR of compound **11.**

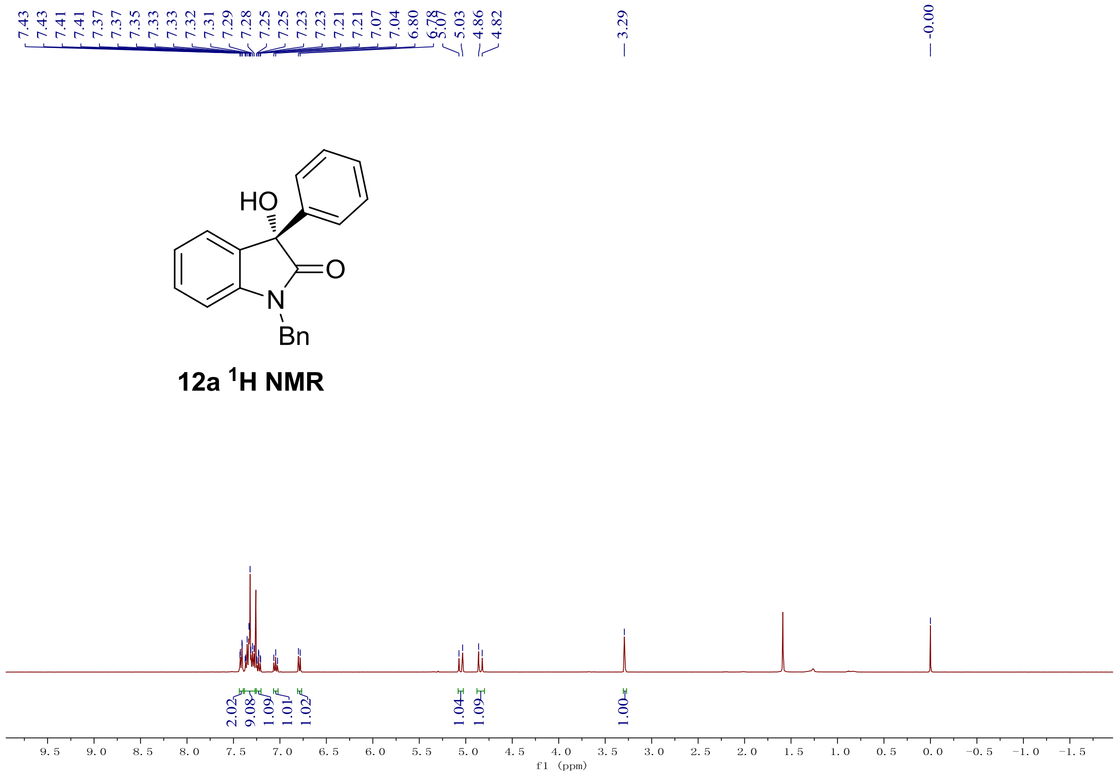

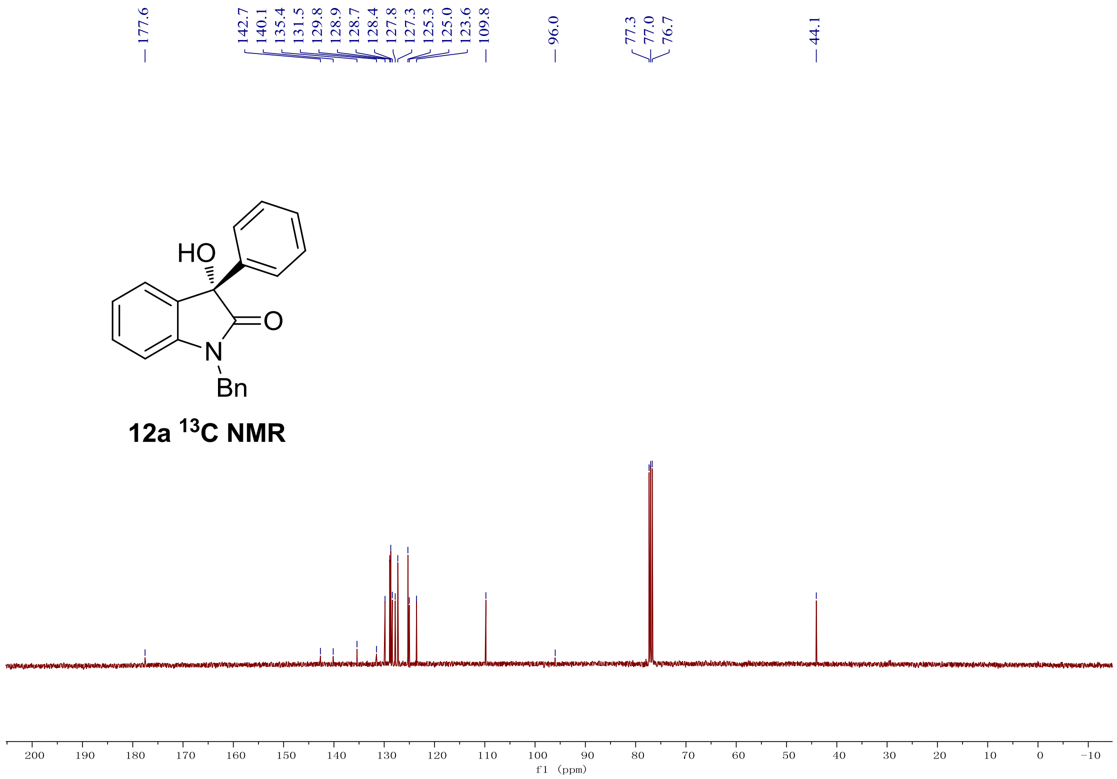

Supplementary Figure 51. ^{1}H and ^{13}C P NMR of compound **12a.**

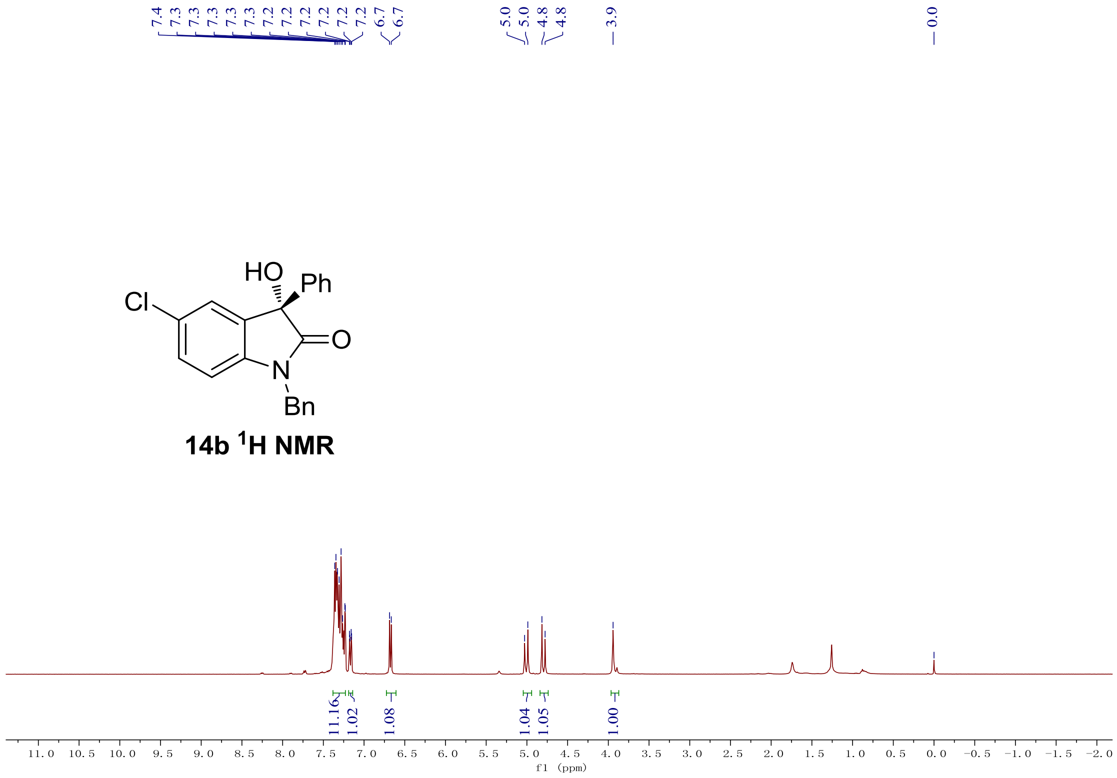

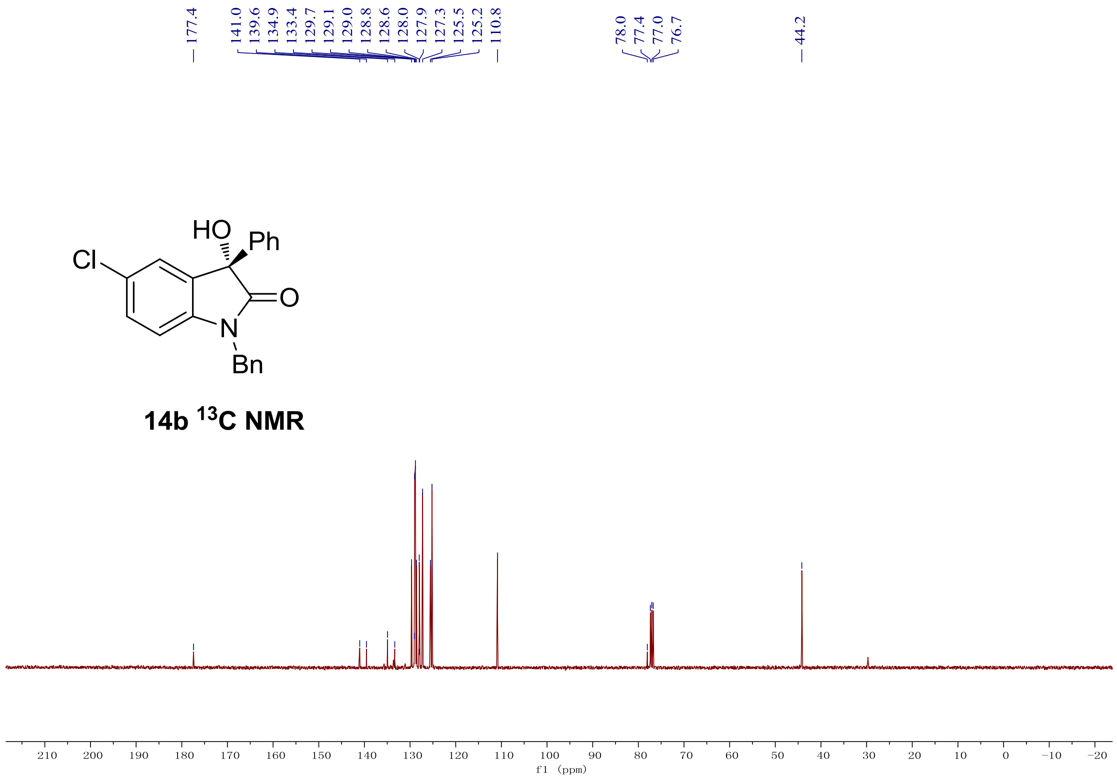

Supplementary Figure 52. ^{1}H and ^{13}C P NMR of compound **14b.**

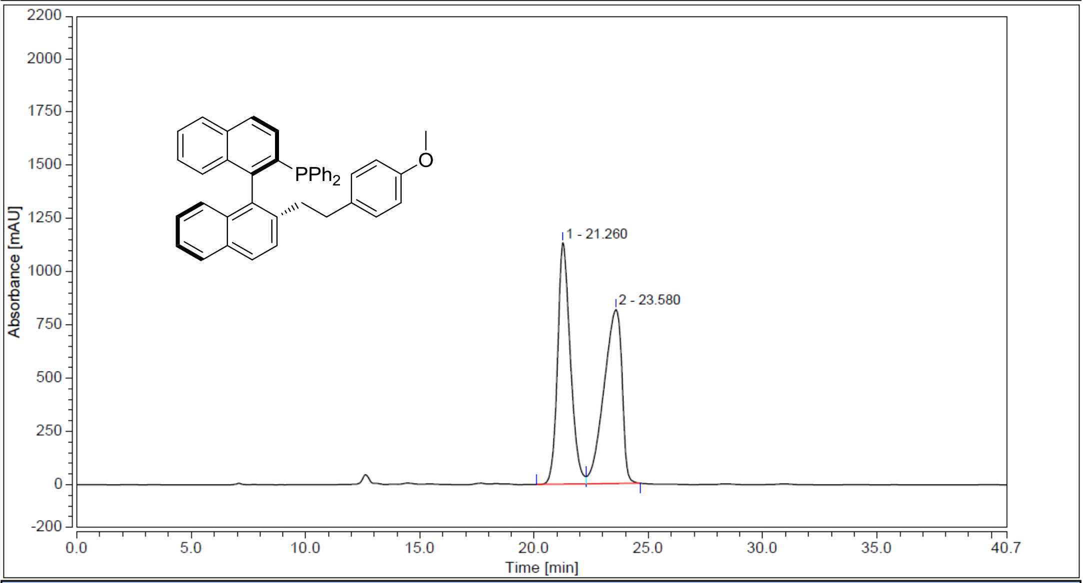

Integration Results							
No.	Peak Name	Retention Time<br>min	Area<br>mAU*min	Height<br>mAU	Relative Area<br>%	Relative Height<br>%	Amount<br>n.a.
1		21.260	734.386	1134.029	49.61	58.10	n.a.
2		23.580	745.969	817.699	50.39	41.90	n.a.
**Total:**			**1480.355**	**1951.728**	**100.00**	**100.00**	

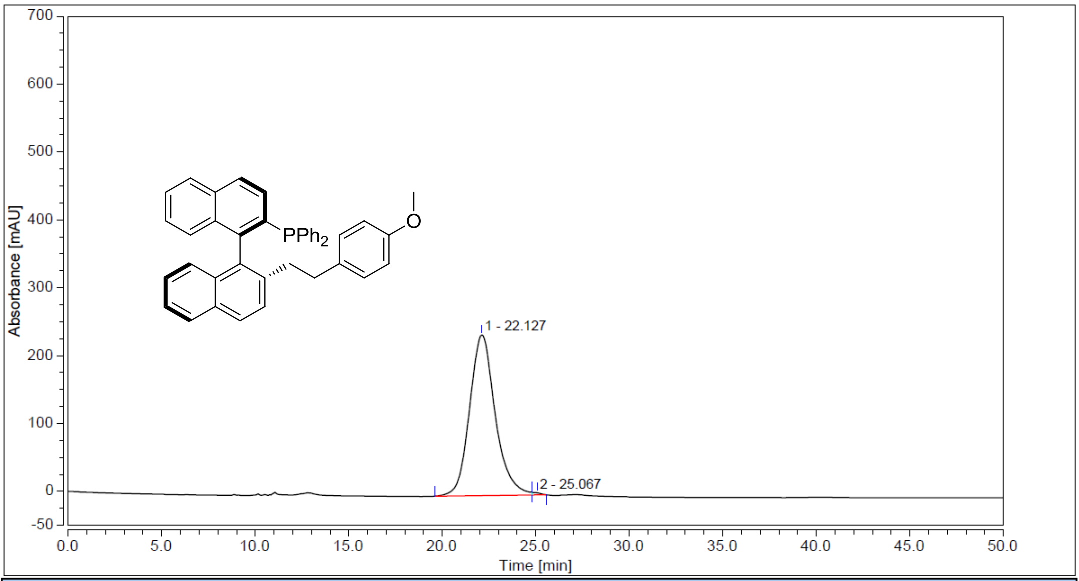

Integration Results							
No.	Peak Name	Retention Time min	Area mAU*min	Height mAU	Relative Area %	Relative Height %	Amount n.a.
1		22.127	376.518	236.799	99.54	98.72	n.a.
2		25.067	1.738	3.082	0.46	1.28	n.a.
**Total:**			**378.256**	**239.881**	**100.00**	**100.00**	

Supplementary Figure 53. HPLC of compound **9.**

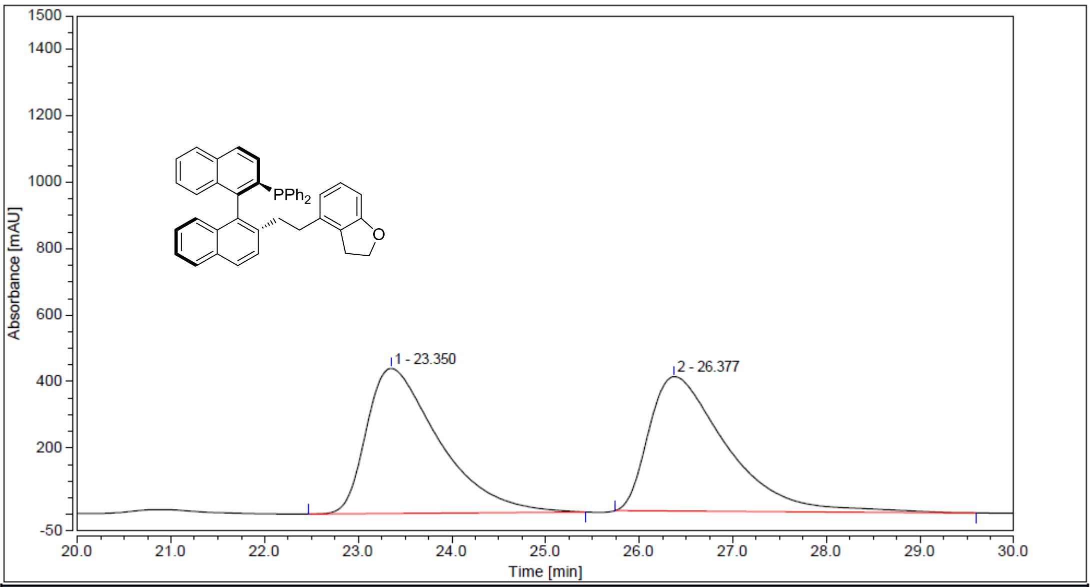

Integration Results							
No.	Peak Name	Retention Time min	Area mAU*min	Height mAU	Relative Area %	Relative Height %	Amount n.a.
1		23.350	391.573	436.816	50.12	51.92	n.a.
2		26.377	389.624	404.544	49.88	48.08	n.a.
**Total:**			**781.197**	**841.361**	**100.00**	**100.00**	

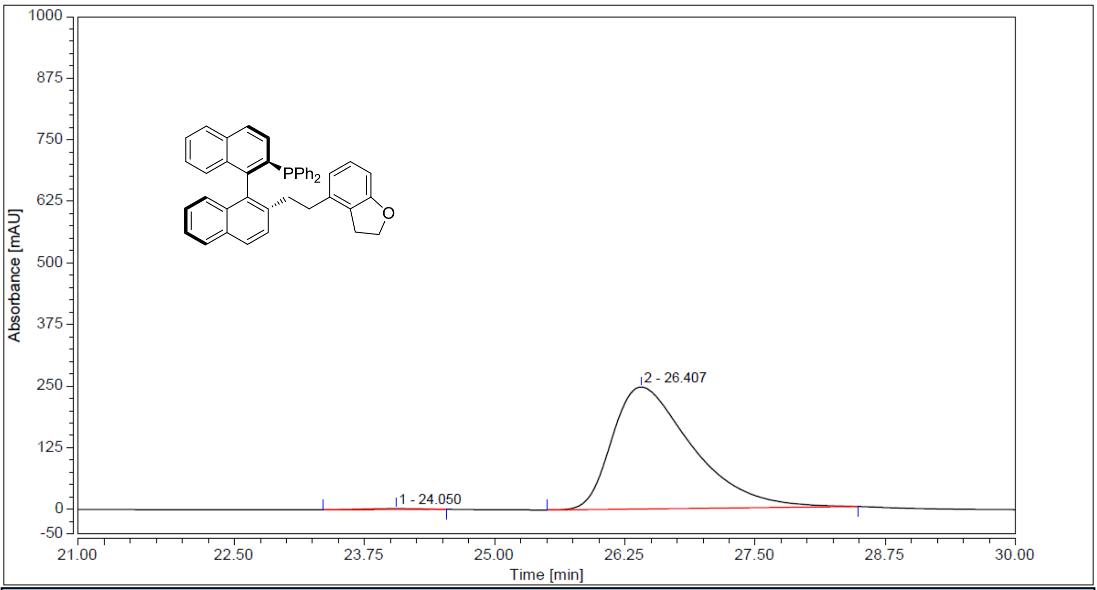

Integration Results							
No.	Peak Name	Retention Time<br>min	Area<br>mAU*min	Height<br>mAU	Relative Area<br>%	Relative Height<br>%	Amount<br>n.a.
1		24.050	1.163	1.859	0.52	0.75	n.a.
2		26.407	220.828	247.530	99.48	99.25	n.a.
**Total:**			**221.991**	**249.388**	**100.00**	**100.00**	

Supplementary Figure 54. HPLC of compound **10.**

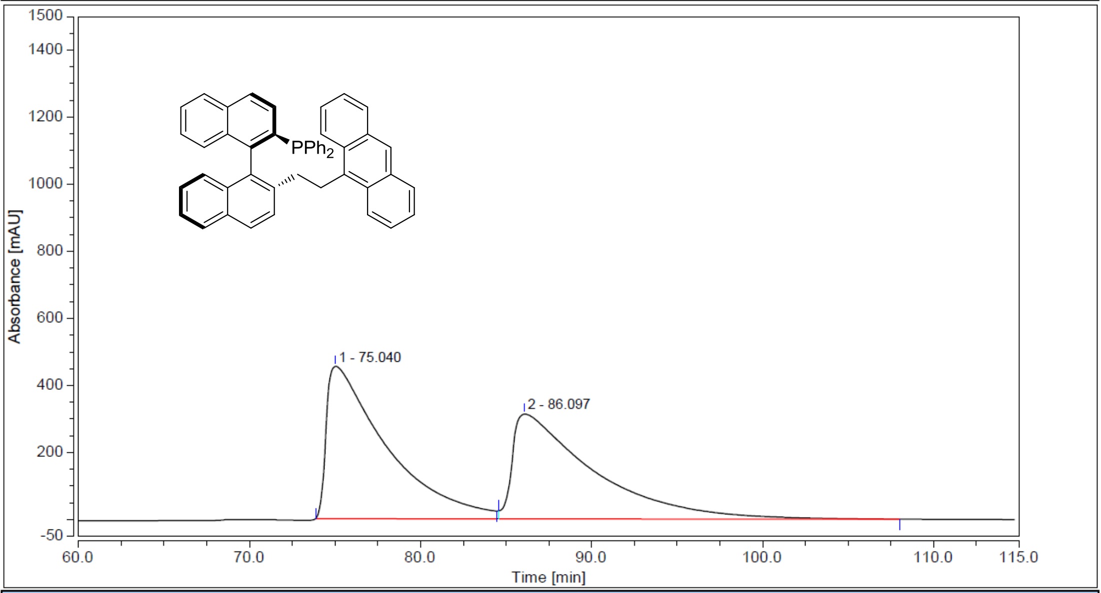

Integration Results							
No.	Peak Name	Retention Time<br>min	Area<br>mAU*min	Height<br>mAU	Relative Area<br>%	Relative Height<br>%	Amount<br>n.a.
1		75.040	1846.677	454.306	52.06	59.25	n.a.
2		86.097	1700.849	312.460	47.94	40.75	n.a.
**Total:**			**3547.527**	**766.766**	**100.00**	**100.00**	

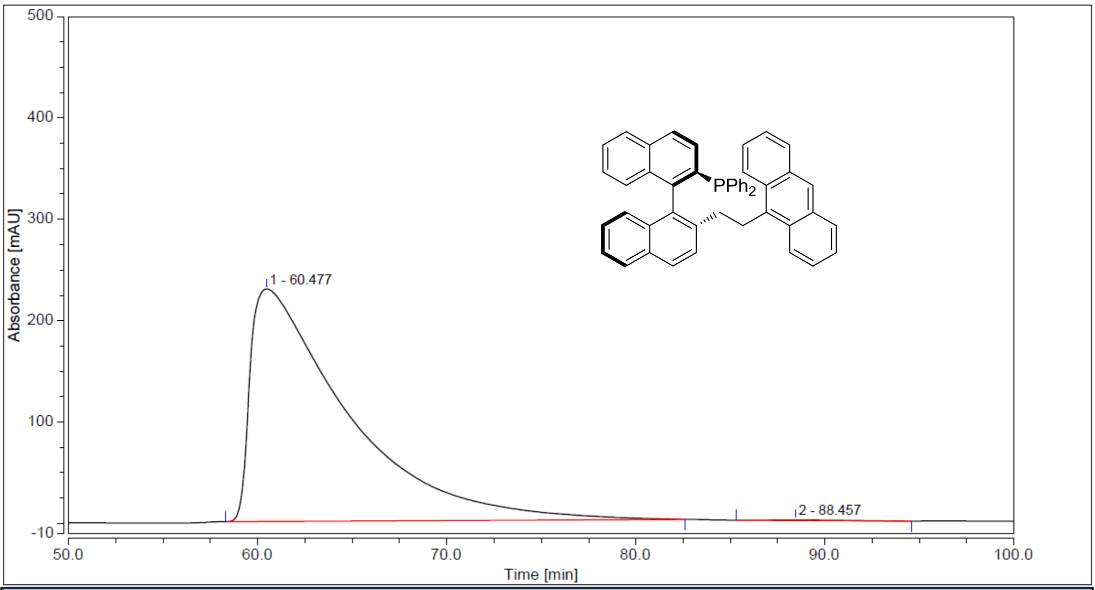

Integration Results							
No.	Peak Name	Retention Time min	Area mAU*min	Height mAU	Relative Area %	Relative Height %	Amount n.a.
1		60.477	1365.327	229.183	99.80	99.76	n.a.
2		88.457	2.743	0.553	0.20	0.24	n.a.
Total:			1368.070	229.736	100.00	100.00	

Supplementary Figure 55. HPLC of compound **11.**

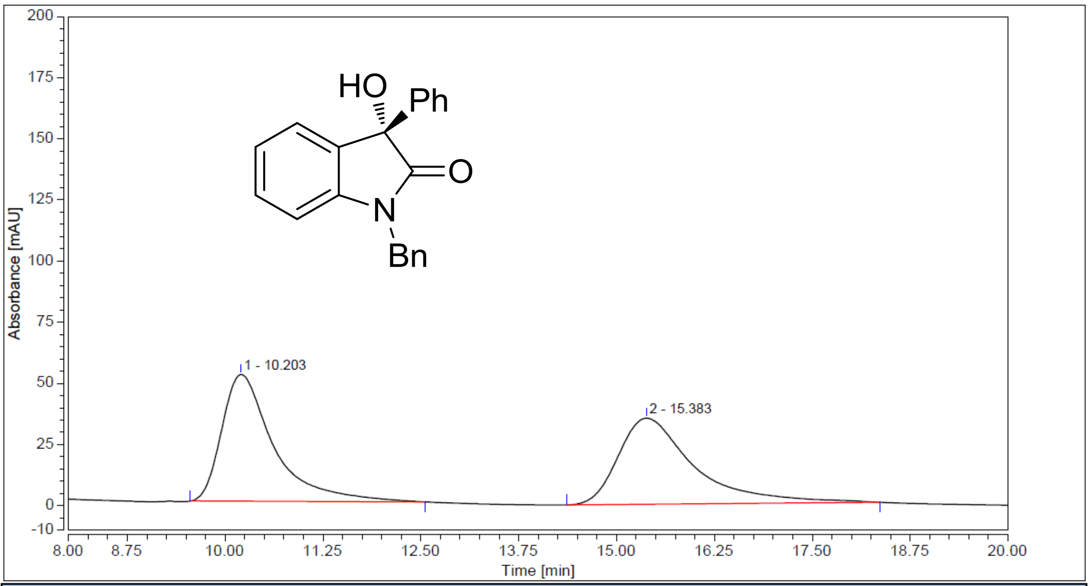

Integration Results							
No.	Peak Name	Retention Time min	Area mAU*min	Height mAU	Relative Area %	Relative Height %	Amount n.a.
1		10.203	39.885	51.835	50.33	59.56	n.a.
2		15.383	39.360	35.192	49.67	40.44	n.a.
Total:			79.245	87.027	100.00	100.00	

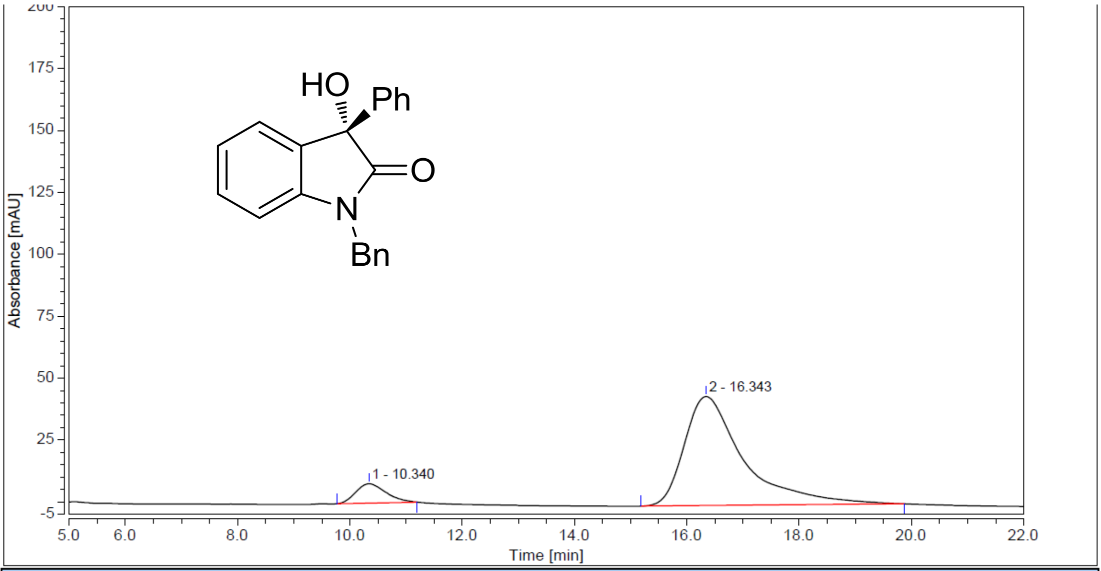

Integration Results							
No.	Peak Name	Retention Time min	Area mAU*min	Height mAU	Relative Area %	Relative Height %	Amount n.a.
1		10.340	4.831	7.779	8.24	15.01	n.a.
2		16.343	53.800	44.059	91.76	84.99	n.a.
**Total:**			**58.631**	**51.838**	**100.00**	**100.00**	

Supplementary Figure 56. HPLC of compound **12a.**

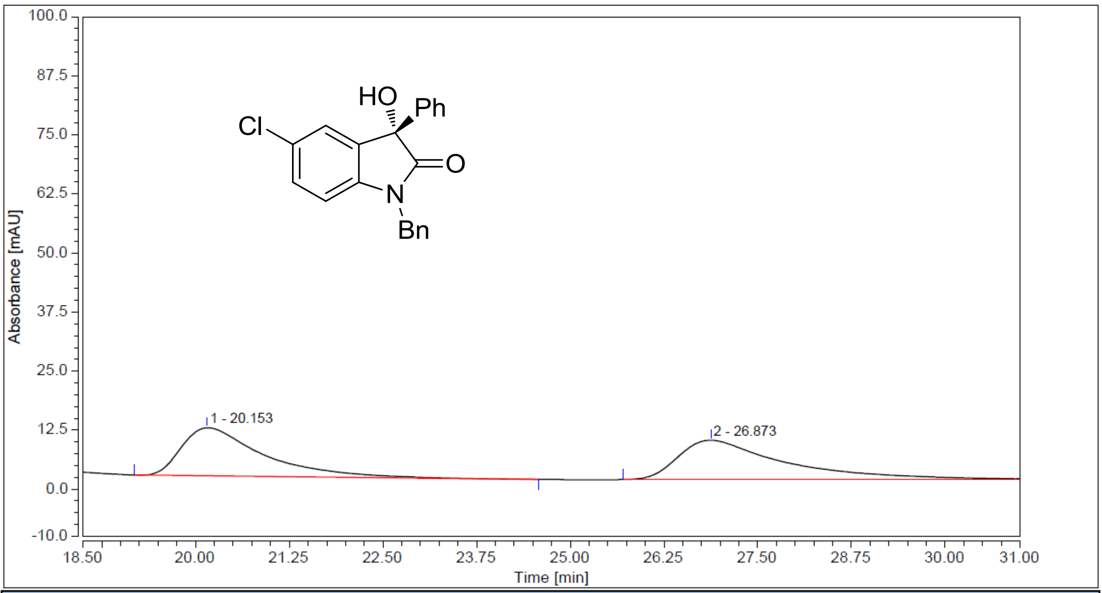

Integration Results							
No.	Peak Name	Retention Time min	Area mAU*min	Height mAU	Relative Area %	Relative Height %	Amount n.a.
1		20.153	12.811	10.159	48.22	54.95	n.a.
2		26.873	13.757	8.328	51.78	45.05	n.a.
**Total:**			**26.569**	**18.487**	**100.00**	**100.00**	

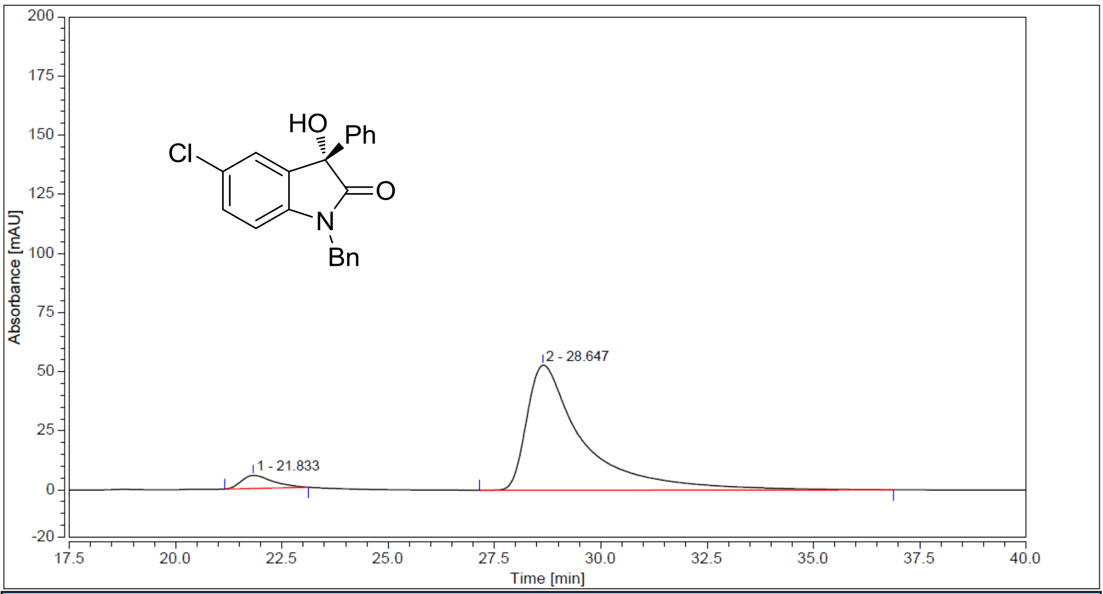

Integration Results

No.	Peak Name	Retention Time min	Area mAU*min	Height mAU	Relative Area %	Relative Height %	Amount n.a.
1		21.833	4.667	5.473	5.41	9.39	n.a.
2		28.647	81.597	52.824	94.59	90.61	n.a.
**Total:**			**86.264**	**58.297**	**100.00**	**100.00**	

Supplementary Figure 57. HPLC of compound **14b.**

Supplementary References

(1) Katsuaki, B.; Mamoru, T.; Naoto, C. Palladium‐Catalyzed Direct Synthesis of Phosphole Derivatives from Triarylphosphines through Cleavage of Carbon–Hydrogen and Carbon–Phosphorus Bonds. *Angew.Chem. Int. Ed*. **52**, 11892-11895 (2013).
