# Supplementary material for: Rhodium-catalysed direct hydroarylation of alkenes and alkynes with phosphines through phosphorous-assisted C−H activation
Source: Nat Commun. 2019 Aug 6;10:3539. doi: 10.1038/s41467-019-11420-5 (PMC6684548; doi:10.1038/s41467-019-11420-5)
Supplement: Supplementary file 3 — Description of Additional Supplementary Files [file 41467_2019_11420_MOESM3_ESM.pdf]

Description of Additional Supplementary Files

File Name: Supplementary Data 1
Description: The Crystallographic Data of CCDC 1893756, CCDC 1893758, CCDC 1893759 and CCDC 1893762.
