# Supplementary material for: Rhodium-catalysed direct hydroarylation of alkenes and alkynes with phosphines through phosphorous-assisted C−H activation
Source: Nat Commun. 2019 Aug 6;10:3539. doi: 10.1038/s41467-019-11420-5 (PMC6684548; doi:10.1038/s41467-019-11420-5)
Supplement: Supplementary file 4 — Supplementary Data 1 [file 41467_2019_11420_MOESM4_ESM.pdf]

Supplementary Data 1

Crystallographic Data

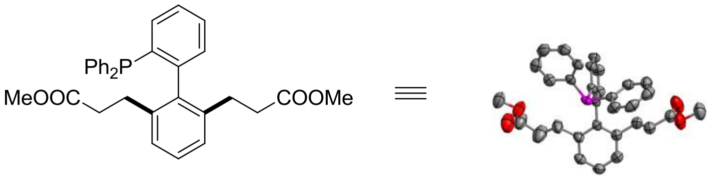

C(1)-C(2)	1.383(6)	C(15)-H(15)	0.9300
C(1)-C(6)	1.390(6)	C(16)-C(17)	1.392(5)
C(1)-H(1)	0.9300	C(16)-H(16)	0.9300
C(2)-C(3)	1.394(7)	C(17)-C(18)	1.392(5)
C(2)-H(2)	0.9300	C(17)-C(19)	1.501(5)
C(3)-C(4)	1.354(8)	C(18)-P(1)	1.838(4)
C(3)-H(3)	0.9300	C(19)-C(24)	1.390(5)
C(4)-C(5)	1.382(7)	C(19)-C(20)	1.406(5)
C(4)-H(4)	0.9300	C(20)-C(21)	1.392(6)
C(5)-C(6)	1.384(5)	C(20)-C(29)	1.505(6)
C(5)-H(5)	0.9300	C(21)-C(22)	1.366(6)
C(6)-P(1)	1.829(4)	C(21)-H(21)	0.9300
C(7)-C(12)	1.381(5)	C(22)-C(23)	1.362(6)
C(7)-C(8)	1.388(6)	C(22)-H(22)	0.9300
C(7)-H(7)	0.9300	C(23)-C(24)	1.402(6)
C(8)-C(9)	1.357(7)	C(23)-H(23)	0.9300
C(8)-H(8)	0.9300	C(24)-C(25)	1.513(6)
C(9)-C(10)	1.372(7)	C(25)-C(26)	1.494(6)
C(9)-H(9)	0.9300	C(25)-H(25A)	0.9700
C(10)-C(11)	1.386(6)	C(25)-H(25B)	0.9700
C(10)-H(10)	0.9300	C(26)-C(27)	1.524(7)
C(11)-C(12)	1.379(5)	C(26)-H(26A)	0.9700
C(11)-H(11)	0.9300	C(26)-H(26B)	0.9700
C(12)-P(1)	1.840(4)	C(27)-O(1)	1.183(6)
C(13)-C(14)	1.368(5)	C(27)-O(4)	1.297(6)
C(13)-C(18)	1.405(5)	C(28)-O(4)	1.422(6)
C(13)-H(13)	0.9300	C(28)-H(28A)	0.9600
C(14)-C(15)	1.376(5)	C(28)-H(28B)	0.9600
C(14)-H(14)	0.9300	C(28)-H(28C)	0.9600
C(15)-C(16)	1.376(6)	C(29)-C(30)	1.330(8)

C(29)-H(29)	0.9300	C(9)-C(10)-H(10)	120.0
C(30)-C(31)	1.457(8)	C(11)-C(10)-H(10)	120.0
C(30)-H(30)	0.9300	C(12)-C(11)-C(10)	120.8(4)
C(31)-O(2)	1.175(6)	C(12)-C(11)-H(11)	119.6
C(31)-O(3)	1.308(6)	C(10)-C(11)-H(11)	119.6
C(32)-O(3)	1.427(6)	C(11)-C(12)-C(7)	118.0(4)
C(32)-H(32A)	0.9600	C(11)-C(12)-P(1)	117.3(3)
C(32)-H(32B)	0.9600	C(7)-C(12)-P(1)	124.6(3)
C(32)-H(32C)	0.9600	C(14)-C(13)-C(18)	122.0(3)
		C(14)-C(13)-H(13)	119.0
C(2)-C(1)-C(6)	121.5(4)	C(18)-C(13)-H(13)	119.0
C(2)-C(1)-H(1)	119.3	C(13)-C(14)-C(15)	119.2(4)
C(6)-C(1)-H(1)	119.3	C(13)-C(14)-H(14)	120.4
C(1)-C(2)-C(3)	118.8(5)	C(15)-C(14)-H(14)	120.4
C(1)-C(2)-H(2)	120.6	C(14)-C(15)-C(16)	119.7(3)
C(3)-C(2)-H(2)	120.6	C(14)-C(15)-H(15)	120.2
C(4)-C(3)-C(2)	120.6(5)	C(16)-C(15)-H(15)	120.2
C(4)-C(3)-H(3)	119.7	C(15)-C(16)-C(17)	122.1(4)
C(2)-C(3)-H(3)	119.7	C(15)-C(16)-H(16)	118.9
C(3)-C(4)-C(5)	120.1(4)	C(17)-C(16)-H(16)	118.9
C(3)-C(4)-H(4)	120.0	C(16)-C(17)-C(18)	118.3(3)
C(5)-C(4)-H(4)	120.0	C(16)-C(17)-C(19)	117.3(3)
C(4)-C(5)-C(6)	121.4(5)	C(18)-C(17)-C(19)	124.3(3)
C(4)-C(5)-H(5)	119.3	C(17)-C(18)-C(13)	118.6(3)
C(6)-C(5)-H(5)	119.3	C(17)-C(18)-P(1)	119.8(3)
C(5)-C(6)-C(1)	117.7(4)	C(13)-C(18)-P(1)	121.6(3)
C(5)-C(6)-P(1)	117.5(3)	C(24)-C(19)-C(20)	120.1(4)
C(1)-C(6)-P(1)	124.9(3)	C(24)-C(19)-C(17)	119.7(3)
C(12)-C(7)-C(8)	121.1(4)	C(20)-C(19)-C(17)	119.9(3)
C(12)-C(7)-H(7)	119.4	C(21)-C(20)-C(19)	118.9(4)
C(8)-C(7)-H(7)	119.4	C(21)-C(20)-C(29)	120.2(4)
C(9)-C(8)-C(7)	119.9(4)	C(19)-C(20)-C(29)	121.0(4)
C(9)-C(8)-H(8)	120.1	C(22)-C(21)-C(20)	121.0(4)
C(7)-C(8)-H(8)	120.1	C(22)-C(21)-H(21)	119.5
C(8)-C(9)-C(10)	120.2(4)	C(20)-C(21)-H(21)	119.5
C(8)-C(9)-H(9)	119.9	C(23)-C(22)-C(21)	120.1(4)
C(10)-C(9)-H(9)	119.9	C(23)-C(22)-H(22)	119.9
C(9)-C(10)-C(11)	119.9(5)	C(21)-C(22)-H(22)	119.9

C(22)-C(23)-C(24)	121.3(4)	O(4)-C(28)-H(28C)	109.5
C(22)-C(23)-H(23)	119.4	H(28A)-C(28)-H(28C)	109.5
C(24)-C(23)-H(23)	119.4	H(28B)-C(28)-H(28C)	109.5
C(19)-C(24)-C(23)	118.6(4)	C(30)-C(29)-C(20)	125.8(5)
C(19)-C(24)-C(25)	122.5(4)	C(30)-C(29)-H(29)	117.1
C(23)-C(24)-C(25)	118.9(4)	C(20)-C(29)-H(29)	117.1
C(26)-C(25)-C(24)	111.2(4)	C(29)-C(30)-C(31)	121.6(6)
C(26)-C(25)-H(25A)	109.4	C(29)-C(30)-H(30)	119.2
C(24)-C(25)-H(25A)	109.4	C(31)-C(30)-H(30)	119.2
C(26)-C(25)-H(25B)	109.4	O(2)-C(31)-O(3)	124.0(5)
C(24)-C(25)-H(25B)	109.4	O(2)-C(31)-C(30)	123.3(6)
H(25A)-C(25)-H(25B)	108.0	O(3)-C(31)-C(30)	112.4(5)
C(25)-C(26)-C(27)	113.6(4)	O(3)-C(32)-H(32A)	109.5
C(25)-C(26)-H(26A)	108.9	O(3)-C(32)-H(32B)	109.5
C(27)-C(26)-H(26A)	108.9	H(32A)-C(32)-H(32B)	109.5
C(25)-C(26)-H(26B)	108.9	O(3)-C(32)-H(32C)	109.5
C(27)-C(26)-H(26B)	108.9	H(32A)-C(32)-H(32C)	109.5
H(26A)-C(26)-H(26B)	107.7	H(32B)-C(32)-H(32C)	109.5
O(1)-C(27)-O(4)	123.5(5)	C(31)-O(3)-C(32)	116.6(4)
O(1)-C(27)-C(26)	127.0(5)	C(27)-O(4)-C(28)	117.6(5)
O(4)-C(27)-C(26)	109.5(5)	C(6)-P(1)-C(18)	103.38(16)
O(4)-C(28)-H(28A)	109.5	C(6)-P(1)-C(12)	101.81(17)
O(4)-C(28)-H(28B)	109.5	C(18)-P(1)-C(12)	99.70(16)
H(28A)-C(28)-H(28B)	109.5		

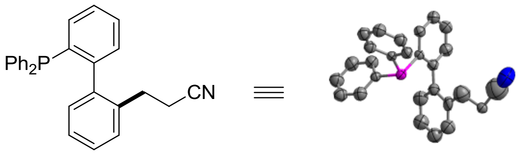

C(1)-C(6)	1.391(9)	C(5)-H(5A)	0.9500
C(1)-C(2)	1.425(10)	C(6)-P(1)	1.832(6)
C(1)-H(1)	0.9500	C(7)-C(12)	1.379(8)
C(2)-C(3)	1.318(11)	C(7)-C(8)	1.398(9)
C(2)-H(2A)	0.9500	C(7)-H(7)	0.9500
C(3)-C(4)	1.350(11)	C(8)-C(9)	1.372(9)
C(3)-H(3A)	0.9500	C(8)-H(8A)	0.9500
C(4)-C(5)	1.393(10)	C(9)-C(10)	1.377(10)
C(4)-H(4A)	0.9500	C(9)-H(9A)	0.9500
C(5)-C(6)	1.369(9)	C(10)-C(11)	1.368(9)

C(10)-H(10A)	0.9500	C(25')-H(25D)	0.9900
C(11)-C(12)	1.390(8)	C(26')-H(26C)	0.9900
C(11)-H(11A)	0.9500	C(26')-H(26D)	0.9900
C(12)-P(1)	1.835(6)		
C(13)-C(18)	1.393(8)	C(6)-C(1)-C(2)	118.4(7)
C(13)-C(14)	1.381(9)	C(6)-C(1)-H(1)	120.8
C(13)-H(13A)	0.9500	C(2)-C(1)-H(1)	120.8
C(14)-C(15)	1.375(10)	C(3)-C(2)-C(1)	120.9(8)
C(14)-H(14A)	0.9500	C(3)-C(2)-H(2A)	119.6
C(15)-C(16)	1.371(10)	C(1)-C(2)-H(2A)	119.6
C(15)-H(15A)	0.9500	C(2)-C(3)-C(4)	122.0(8)
C(16)-C(17)	1.397(9)	C(2)-C(3)-H(3A)	119.0
C(16)-H(16A)	0.9500	C(4)-C(3)-H(3A)	119.0
C(17)-C(18)	1.406(8)	C(3)-C(4)-C(5)	118.4(8)
C(17)-C(19)	1.491(9)	C(3)-C(4)-H(4A)	120.8
C(18)-P(1)	1.837(6)	C(5)-C(4)-H(4A)	120.8
C(19)-C(24)	1.394(10)	C(6)-C(5)-C(4)	122.2(7)
C(19)-C(20)	1.378(10)	C(6)-C(5)-H(5A)	118.9
C(20)-C(21)	1.414(12)	C(4)-C(5)-H(5A)	118.9
C(20)-H(20A)	0.9500	C(5)-C(6)-C(1)	118.0(6)
C(21)-C(22)	1.348(12)	C(5)-C(6)-P(1)	124.2(5)
C(21)-H(21A)	0.9500	C(1)-C(6)-P(1)	117.7(5)
C(22)-C(23)	1.341(11)	C(12)-C(7)-C(8)	120.4(6)
C(22)-H(22A)	0.9500	C(12)-C(7)-H(7)	119.8
C(23)-C(24)	1.392(11)	C(8)-C(7)-H(7)	119.8
C(23)-H(23A)	0.9500	C(9)-C(8)-C(7)	120.0(7)
C(24)-C(25')	1.463(11)	C(9)-C(8)-H(8A)	120.0
C(24)-C(25)	1.463(11)	C(7)-C(8)-H(8A)	120.0
C(25)-C(26)	1.39(2)	C(10)-C(9)-C(8)	119.8(7)
C(25)-H(25A)	0.9900	C(10)-C(9)-H(9A)	120.1
C(25)-H(25B)	0.9900	C(8)-C(9)-H(9A)	120.1
C(26)-C(27)	1.57(2)	C(11)-C(10)-C(9)	120.2(7)
C(26)-H(26A)	0.9900	C(11)-C(10)-H(10A)	119.9
C(26)-H(26B)	0.9900	C(9)-C(10)-H(10A)	119.9
C(27)-N(1)	1.149(16)	C(10)-C(11)-C(12)	121.2(6)
C(27)-C(26')	1.59(2)	C(10)-C(11)-H(11A)	119.4
C(25')-C(26')	1.479(18)	C(12)-C(11)-H(11A)	119.4
C(25')-H(25C)	0.9900	C(7)-C(12)-C(11)	118.4(5)

C(7)-C(12)-P(1)	124.7(4)
C(11)-C(12)-P(1)	116.8(4)
C(18)-C(13)-C(14)	121.2(6)
C(18)-C(13)-H(13A)	119.4
C(14)-C(13)-H(13A)	119.4
C(15)-C(14)-C(13)	119.2(7)
C(15)-C(14)-H(14A)	120.4
C(13)-C(14)-H(14A)	120.4
C(16)-C(15)-C(14)	120.5(7)
C(16)-C(15)-H(15A)	119.8
C(14)-C(15)-H(15A)	119.8
C(15)-C(16)-C(17)	121.7(7)
C(15)-C(16)-H(16A)	119.2
C(17)-C(16)-H(16A)	119.2
C(18)-C(17)-C(16)	117.8(6)
C(18)-C(17)-C(19)	122.2(5)
C(16)-C(17)-C(19)	120.0(6)
C(13)-C(18)-C(17)	119.6(5)
C(13)-C(18)-P(1)	123.4(4)
C(17)-C(18)-P(1)	116.9(4)
C(24)-C(19)-C(20)	119.5(7)
C(24)-C(19)-C(17)	121.0(6)
C(20)-C(19)-C(17)	119.5(6)
C(19)-C(20)-C(21)	119.8(8)
C(19)-C(20)-H(20A)	120.1
C(21)-C(20)-H(20A)	120.1
C(22)-C(21)-C(20)	119.1(9)
C(22)-C(21)-H(21A)	120.5
C(20)-C(21)-H(21A)	120.5
C(23)-C(22)-C(21)	122.0(9)
C(23)-C(22)-H(22A)	119.0
C(21)-C(22)-H(22A)	119.0
C(22)-C(23)-C(24)	120.7(8)
C(22)-C(23)-H(23A)	119.6
C(24)-C(23)-H(23A)	119.6
C(19)-C(24)-C(23)	119.0(7)
C(19)-C(24)-C(25')	119.8(7)
C(23)-C(24)-C(25')	121.2(7)
C(19)-C(24)-C(25)	119.8(7)
C(23)-C(24)-C(25)	121.2(7)
C(26)-C(25)-C(24)	120.1(12)
C(26)-C(25)-H(25A)	107.3
C(24)-C(25)-H(25A)	107.3
C(26)-C(25)-H(25B)	107.3
C(24)-C(25)-H(25B)	107.3
H(25A)-C(25)-H(25B)	106.9
C(25)-C(26)-C(27)	109.3(16)
C(25)-C(26)-H(26A)	109.8
C(27)-C(26)-H(26A)	109.8
C(25)-C(26)-H(26B)	109.8
C(27)-C(26)-H(26B)	109.8
H(26A)-C(26)-H(26B)	108.3
N(1)-C(27)-C(26')	166.3(17)
N(1)-C(27)-C(26)	151.7(18)
C(24)-C(25')-C(26')	118.3(10)
C(24)-C(25')-H(25C)	107.7
C(26')-C(25')-H(25C)	107.7
C(24)-C(25')-H(25D)	107.7
C(26')-C(25')-H(25D)	107.7
H(25C)-C(25')-H(25D)	107.1
C(25')-C(26')-C(27)	103.5(12)
C(25')-C(26')-H(26C)	111.1
C(27)-C(26')-H(26C)	111.1
C(25')-C(26')-H(26D)	111.1
C(27)-C(26')-H(26D)	111.1
H(26C)-C(26')-H(26D)	109.0
C(12)-P(1)-C(6)	102.2(3)
C(12)-P(1)-C(18)	101.8(2)
C(6)-P(1)-C(18)	101.0(3)

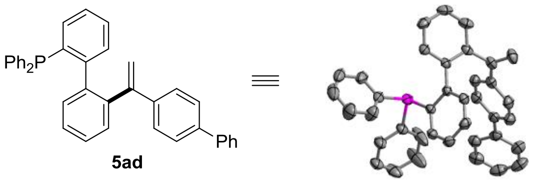

C(1)-C(2)	1.385(4)	C(18)-H(18)	0.9500
C(1)-C(6)	1.396(4)	C(19)-C(20)	1.391(4)
C(1)-H(1)	0.9500	C(19)-H(19)	0.9500
C(2)-C(3)	1.371(5)	C(20)-C(21)	1.494(3)
C(2)-H(2)	0.9500	C(21)-C(22)	1.396(3)
C(3)-C(4)	1.367(5)	C(21)-C(26)	1.412(3)
C(3)-H(3)	0.9500	C(22)-C(23)	1.381(4)
C(4)-C(5)	1.389(4)	C(22)-H(22)	0.9500
C(4)-H(4)	0.9500	C(23)-C(24)	1.384(4)
C(5)-C(6)	1.398(4)	C(23)-H(23)	0.9500
C(5)-H(5)	0.9500	C(24)-C(25)	1.378(4)
C(6)-C(7)	1.488(4)	C(24)-H(24)	0.9500
C(7)-C(8)	1.391(4)	C(25)-C(26)	1.389(3)
C(7)-C(12)	1.395(4)	C(25)-H(25)	0.9500
C(8)-C(9)	1.386(4)	C(26)-P(1)	1.840(2)
C(8)-H(8)	0.9500	C(27)-C(28)	1.365(5)
C(9)-C(10)	1.389(4)	C(27)-C(32)	1.374(4)
C(9)-H(9)	0.9500	C(27)-P(1)	1.836(3)
C(10)-C(11)	1.395(4)	C(28)-C(29)	1.383(5)
C(10)-C(13)	1.490(4)	C(28)-H(28)	0.9500
C(11)-C(12)	1.372(4)	C(29)-C(30)	1.373(5)
C(11)-H(11)	0.9500	C(29)-H(29)	0.9500
C(12)-H(12)	0.9500	C(30)-C(31)	1.333(5)
C(13)-C(14)	1.330(4)	C(30)-H(30)	0.9500
C(13)-C(15)	1.487(4)	C(31)-C(32)	1.389(4)
C(14)-H(14A)	0.9500	C(31)-H(31)	0.9500
C(14)-H(14B)	0.9500	C(32)-H(32)	0.9500
C(15)-C(16)	1.399(4)	C(33)-C(38)	1.380(5)
C(15)-C(20)	1.410(4)	C(33)-C(34)	1.390(4)
C(16)-C(17)	1.366(4)	C(33)-P(1)	1.832(3)
C(16)-H(16)	0.9500	C(34)-C(35)	1.386(5)
C(17)-C(18)	1.372(5)	C(34)-H(34)	0.9500
C(17)-H(17)	0.9500	C(35)-C(36)	1.356(7)
C(18)-C(19)	1.392(4)	C(35)-H(35)	0.9500

C(36)-C(37)	1.366(6)	C(11)-C(12)-C(7)	121.8(3)
C(36)-H(36)	0.9500	C(11)-C(12)-H(12)	119.1
C(37)-C(38)	1.374(5)	C(7)-C(12)-H(12)	119.1
C(37)-H(37)	0.9500	C(14)-C(13)-C(15)	119.8(3)
C(38)-H(38)	0.9500	C(14)-C(13)-C(10)	121.1(3)
C(2)-C(1)-C(6)	120.6(3)	C(15)-C(13)-C(10)	119.0(2)
C(2)-C(1)-H(1)	119.7	C(13)-C(14)-H(14A)	120.0
C(6)-C(1)-H(1)	119.7	C(13)-C(14)-H(14B)	120.0
C(3)-C(2)-C(1)	121.0(3)	H(14A)-C(14)-H(14B)	120.0
C(3)-C(2)-H(2)	119.5	C(16)-C(15)-C(20)	118.6(3)
C(1)-C(2)-H(2)	119.5	C(16)-C(15)-C(13)	118.9(2)
C(4)-C(3)-C(2)	119.3(3)	C(20)-C(15)-C(13)	122.4(2)
C(4)-C(3)-H(3)	120.3	C(17)-C(16)-C(15)	122.0(3)
C(2)-C(3)-H(3)	120.3	C(17)-C(16)-H(16)	119.0
C(3)-C(4)-C(5)	120.7(3)	C(15)-C(16)-H(16)	119.0
C(3)-C(4)-H(4)	119.6	C(16)-C(17)-C(18)	119.5(3)
C(5)-C(4)-H(4)	119.6	C(16)-C(17)-H(17)	120.2
C(4)-C(5)-C(6)	120.7(3)	C(18)-C(17)-H(17)	120.2
C(4)-C(5)-H(5)	119.7	C(17)-C(18)-C(19)	120.3(3)
C(6)-C(5)-H(5)	119.7	C(17)-C(18)-H(18)	119.9
C(1)-C(6)-C(5)	117.6(3)	C(19)-C(18)-H(18)	119.9
C(1)-C(6)-C(7)	121.4(3)	C(20)-C(19)-C(18)	121.0(3)
C(5)-C(6)-C(7)	121.0(3)	C(20)-C(19)-H(19)	119.5
C(8)-C(7)-C(12)	116.8(2)	C(18)-C(19)-H(19)	119.5
C(8)-C(7)-C(6)	122.1(2)	C(19)-C(20)-C(15)	118.7(2)
C(12)-C(7)-C(6)	121.1(2)	C(19)-C(20)-C(21)	119.6(2)
C(9)-C(8)-C(7)	121.5(2)	C(15)-C(20)-C(21)	121.4(2)
C(9)-C(8)-H(8)	119.2	C(22)-C(21)-C(26)	118.5(2)
C(7)-C(8)-H(8)	119.2	C(22)-C(21)-C(20)	117.4(2)
C(8)-C(9)-C(10)	121.2(2)	C(26)-C(21)-C(20)	124.0(2)
C(8)-C(9)-H(9)	119.4	C(23)-C(22)-C(21)	121.5(2)
C(10)-C(9)-H(9)	119.4	C(23)-C(22)-H(22)	119.2
C(9)-C(10)-C(11)	117.2(2)	C(21)-C(22)-H(22)	119.2
C(9)-C(10)-C(13)	121.5(2)	C(22)-C(23)-C(24)	119.8(2)
C(11)-C(10)-C(13)	121.2(2)	C(22)-C(23)-H(23)	120.1
C(12)-C(11)-C(10)	121.4(3)	C(24)-C(23)-H(23)	120.1
C(12)-C(11)-H(11)	119.3	C(25)-C(24)-C(23)	119.3(2)
C(10)-C(11)-H(11)	119.3	C(25)-C(24)-H(24)	120.4

C(23)-C(24)-H(24)	120.4	C(27)-C(32)-H(32)	119.2
C(24)-C(25)-C(26)	122.1(2)	C(31)-C(32)-H(32)	119.2
C(24)-C(25)-H(25)	119.0	C(38)-C(33)-C(34)	117.2(3)
C(26)-C(25)-H(25)	119.0	C(38)-C(33)-P(1)	124.9(2)
C(25)-C(26)-C(21)	118.7(2)	C(34)-C(33)-P(1)	117.9(3)
C(25)-C(26)-P(1)	121.35(18)	C(35)-C(34)-C(33)	120.4(4)
C(21)-C(26)-P(1)	119.91(18)	C(35)-C(34)-H(34)	119.8
C(28)-C(27)-C(32)	116.8(3)	C(33)-C(34)-H(34)	119.8
C(28)-C(27)-P(1)	118.2(2)	C(36)-C(35)-C(34)	120.9(4)
C(32)-C(27)-P(1)	124.7(2)	C(36)-C(35)-H(35)	119.6
C(27)-C(28)-C(29)	121.4(3)	C(34)-C(35)-H(35)	119.6
C(27)-C(28)-H(28)	119.3	C(35)-C(36)-C(37)	119.6(4)
C(29)-C(28)-H(28)	119.3	C(35)-C(36)-H(36)	120.2
C(30)-C(29)-C(28)	120.5(4)	C(37)-C(36)-H(36)	120.2
C(30)-C(29)-H(29)	119.8	C(36)-C(37)-C(38)	120.0(4)
C(28)-C(29)-H(29)	119.8	C(36)-C(37)-H(37)	120.0
C(31)-C(30)-C(29)	118.8(3)	C(38)-C(37)-H(37)	120.0
C(31)-C(30)-H(30)	120.6	C(37)-C(38)-C(33)	121.9(3)
C(29)-C(30)-H(30)	120.6	C(37)-C(38)-H(38)	119.1
C(30)-C(31)-C(32)	120.8(3)	C(33)-C(38)-H(38)	119.1
C(30)-C(31)-H(31)	119.6	C(33)-P(1)-C(27)	101.50(12)
C(32)-C(31)-H(31)	119.6	C(33)-P(1)-C(26)	101.69(12)
C(27)-C(32)-C(31)	121.6(3)	C(27)-P(1)-C(26)	102.62(11)

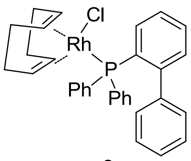

8

≡

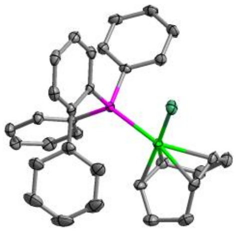

C(1)-C(2)	1.393(3)	C(5)-H(5)	0.9500
C(1)-C(6)	1.412(3)	C(6)-C(7)	1.494(3)
C(1)-P(1)	1.845(2)	C(7)-C(8)	1.390(3)
C(2)-C(3)	1.382(3)	C(7)-C(12)	1.391(4)
C(2)-H(2)	0.9500	C(8)-C(9)	1.379(4)
C(3)-C(4)	1.386(3)	C(8)-H(8)	0.9500
C(3)-H(3)	0.9500	C(9)-C(10)	1.385(5)
C(4)-C(5)	1.377(3)	C(9)-H(9)	0.9500
C(4)-H(4)	0.9500	C(10)-C(11)	1.375(5)
C(5)-C(6)	1.393(3)	C(10)-H(10)	0.9500

C(11)-C(12)	1.389(4)	C(28)-H(28A)	0.9900
C(11)-H(11)	0.9500	C(28)-H(28B)	0.9900
C(12)-H(12)	0.9500	C(29)-C(30)	1.375(4)
C(13)-C(14)	1.383(3)	C(29)-Rh(1)	2.188(2)
C(13)-C(18)	1.397(3)	C(29)-H(29)	1.0000
C(13)-H(13)	0.9500	C(30)-C(31)	1.508(4)
C(14)-C(15)	1.382(4)	C(30)-Rh(1)	2.179(2)
C(14)-H(14)	0.9500	C(30)-H(30)	1.0000
C(15)-C(16)	1.369(4)	C(31)-C(32)	1.527(4)
C(15)-H(15)	0.9500	C(31)-H(31A)	0.9900
C(16)-C(17)	1.393(3)	C(31)-H(31B)	0.9900
C(16)-H(16)	0.9500	C(32)-H(32A)	0.9900
C(17)-C(18)	1.386(3)	C(32)-H(32B)	0.9900
C(17)-H(17)	0.9500	Cl(1)-Rh(1)	2.3767(6)
C(18)-P(1)	1.839(2)	P(1)-Rh(1)	2.3452(6)
C(19)-C(20)	1.379(3)		
C(19)-C(24)	1.390(3)	C(2)-C(1)-C(6)	118.7(2)
C(19)-H(19)	0.9500	C(2)-C(1)-P(1)	119.38(17)
C(20)-C(21)	1.383(3)	C(6)-C(1)-P(1)	121.90(17)
C(20)-H(20)	0.9500	C(3)-C(2)-C(1)	121.7(2)
C(21)-C(22)	1.380(3)	C(3)-C(2)-H(2)	119.1
C(21)-H(21)	0.9500	C(1)-C(2)-H(2)	119.1
C(22)-C(23)	1.380(3)	C(2)-C(3)-C(4)	119.4(2)
C(22)-H(22)	0.9500	C(2)-C(3)-H(3)	120.3
C(23)-C(24)	1.395(3)	C(4)-C(3)-H(3)	120.3
C(23)-H(23)	0.9500	C(5)-C(4)-C(3)	119.7(2)
C(24)-P(1)	1.829(2)	C(5)-C(4)-H(4)	120.1
C(25)-C(26)	1.405(4)	C(3)-C(4)-H(4)	120.1
C(25)-C(32)	1.521(3)	C(4)-C(5)-C(6)	121.8(2)
C(25)-Rh(1)	2.131(2)	C(4)-C(5)-H(5)	119.1
C(25)-H(25)	1.0000	C(6)-C(5)-H(5)	119.1
C(26)-C(27)	1.513(4)	C(5)-C(6)-C(1)	118.5(2)
C(26)-Rh(1)	2.121(2)	C(5)-C(6)-C(7)	117.4(2)
C(26)-H(26)	1.0000	C(1)-C(6)-C(7)	124.1(2)
C(27)-C(28)	1.531(4)	C(8)-C(7)-C(12)	118.7(2)
C(27)-H(27A)	0.9900	C(8)-C(7)-C(6)	119.9(2)
C(27)-H(27B)	0.9900	C(12)-C(7)-C(6)	121.3(2)
C(28)-C(29)	1.523(4)	C(9)-C(8)-C(7)	120.6(3)

C(9)-C(8)-H(8)	119.7	C(22)-C(21)-C(20)	119.1(2)
C(7)-C(8)-H(8)	119.7	C(22)-C(21)-H(21)	120.4
C(8)-C(9)-C(10)	120.1(3)	C(20)-C(21)-H(21)	120.4
C(8)-C(9)-H(9)	119.9	C(23)-C(22)-C(21)	120.8(2)
C(10)-C(9)-H(9)	119.9	C(23)-C(22)-H(22)	119.6
C(11)-C(10)-C(9)	120.0(3)	C(21)-C(22)-H(22)	119.6
C(11)-C(10)-H(10)	120.0	C(22)-C(23)-C(24)	120.2(2)
C(9)-C(10)-H(10)	120.0	C(22)-C(23)-H(23)	119.9
C(10)-C(11)-C(12)	119.9(3)	C(24)-C(23)-H(23)	119.9
C(10)-C(11)-H(11)	120.1	C(19)-C(24)-C(23)	118.8(2)
C(12)-C(11)-H(11)	120.1	C(19)-C(24)-P(1)	122.23(17)
C(11)-C(12)-C(7)	120.6(3)	C(23)-C(24)-P(1)	118.95(17)
C(11)-C(12)-H(12)	119.7	C(26)-C(25)-C(32)	123.0(2)
C(7)-C(12)-H(12)	119.7	C(26)-C(25)-Rh(1)	70.32(14)
C(14)-C(13)-C(18)	121.0(2)	C(32)-C(25)-Rh(1)	114.00(16)
C(14)-C(13)-H(13)	119.5	C(26)-C(25)-H(25)	114.1
C(18)-C(13)-H(13)	119.5	C(32)-C(25)-H(25)	114.1
C(15)-C(14)-C(13)	120.1(2)	Rh(1)-C(25)-H(25)	114.1
C(15)-C(14)-H(14)	120.0	C(25)-C(26)-C(27)	123.7(2)
C(13)-C(14)-H(14)	120.0	C(25)-C(26)-Rh(1)	71.10(13)
C(16)-C(15)-C(14)	119.8(2)	C(27)-C(26)-Rh(1)	111.85(17)
C(16)-C(15)-H(15)	120.1	C(25)-C(26)-H(26)	114.2
C(14)-C(15)-H(15)	120.1	C(27)-C(26)-H(26)	114.2
C(15)-C(16)-C(17)	120.2(2)	Rh(1)-C(26)-H(26)	114.2
C(15)-C(16)-H(16)	119.9	C(26)-C(27)-C(28)	113.4(2)
C(17)-C(16)-H(16)	119.9	C(26)-C(27)-H(27A)	108.9
C(18)-C(17)-C(16)	120.9(2)	C(28)-C(27)-H(27A)	108.9
C(18)-C(17)-H(17)	119.5	C(26)-C(27)-H(27B)	108.9
C(16)-C(17)-H(17)	119.5	C(28)-C(27)-H(27B)	108.9
C(17)-C(18)-C(13)	117.9(2)	H(27A)-C(27)-H(27B)	107.7
C(17)-C(18)-P(1)	122.75(18)	C(29)-C(28)-C(27)	113.5(2)
C(13)-C(18)-P(1)	119.35(17)	C(29)-C(28)-H(28A)	108.9
C(20)-C(19)-C(24)	120.4(2)	C(27)-C(28)-H(28A)	108.9
C(20)-C(19)-H(19)	119.8	C(29)-C(28)-H(28B)	108.9
C(24)-C(19)-H(19)	119.8	C(27)-C(28)-H(28B)	108.9
C(19)-C(20)-C(21)	120.7(2)	H(28A)-C(28)-H(28B)	107.7
C(19)-C(20)-H(20)	119.7	C(30)-C(29)-C(28)	123.8(3)
C(21)-C(20)-H(20)	119.7	C(30)-C(29)-Rh(1)	71.30(14)

C(28)-C(29)-Rh(1)	111.96(17)	C(24)-P(1)-C(18)	101.42(10)
C(30)-C(29)-H(29)	114.1	C(24)-P(1)-C(1)	104.20(10)
C(28)-C(29)-H(29)	114.1	C(18)-P(1)-C(1)	102.58(10)
Rh(1)-C(29)-H(29)	114.1	C(24)-P(1)-Rh(1)	107.72(7)
C(29)-C(30)-C(31)	125.9(2)	C(18)-P(1)-Rh(1)	122.74(7)
C(29)-C(30)-Rh(1)	72.00(14)	C(1)-P(1)-Rh(1)	115.91(7)
C(31)-C(30)-Rh(1)	109.08(16)	C(26)-Rh(1)-C(25)	38.58(10)
C(29)-C(30)-H(30)	113.9	C(26)-Rh(1)-C(30)	96.98(10)
C(31)-C(30)-H(30)	113.9	C(25)-Rh(1)-C(30)	81.12(9)
Rh(1)-C(30)-H(30)	113.9	C(26)-Rh(1)-C(29)	81.51(10)
C(30)-C(31)-C(32)	113.4(2)	C(25)-Rh(1)-C(29)	89.30(10)
C(30)-C(31)-H(31A)	108.9	C(30)-Rh(1)-C(29)	36.70(10)
C(32)-C(31)-H(31A)	108.9	C(26)-Rh(1)-P(1)	95.85(7)
C(30)-C(31)-H(31B)	108.9	C(25)-Rh(1)-P(1)	97.16(7)
C(32)-C(31)-H(31B)	108.9	C(30)-Rh(1)-P(1)	156.60(7)
H(31A)-C(31)-H(31B)	107.7	C(29)-Rh(1)-P(1)	166.08(7)
C(25)-C(32)-C(31)	112.0(2)	C(26)-Rh(1)-Cl(1)	164.59(8)
C(25)-C(32)-H(32A)	109.2	C(25)-Rh(1)-Cl(1)	156.28(7)
C(31)-C(32)-H(32A)	109.2	C(30)-Rh(1)-Cl(1)	86.28(7)
C(25)-C(32)-H(32B)	109.2	C(29)-Rh(1)-Cl(1)	92.52(7)
C(31)-C(32)-H(32B)	109.2	P(1)-Rh(1)-Cl(1)	86.52(2)
H(32A)-C(32)-H(32B)	107.9		
